# STModule: identifying tissue modules to uncover spatial components and characteristics of transcriptomic landscapes

**DOI:** 10.1186/s13073-025-01441-9

**Published:** 2025-03-03

**Authors:** Ran Wang, Yan Qian, Xiaojing Guo, Fangda Song, Zhiqiang Xiong, Shirong Cai, Xiuwu Bian, Man Hon Wong, Qin Cao, Lixin Cheng, Gang Lu, Kwong Sak Leung

**Affiliations:** 1 CUHK-SDU Joint Laboratory on Reproductive Genetics, School of Biomedical Sciences, The Chinese University of Hong Kong, Shatin, New Territories, Hong Kong, 999077 China; 2 Center for Neuromusculoskeletal Restorative Medicine, Hong Kong Science Park, Shatin, New Territories, Hong Kong, 999077 China; 3 Department of Computer Science and Engineering, The Chinese University of Hong Kong, Shatin, New Territories, Hong Kong, 999077 China; 4 Department of Gastrointestinal Surgery Center, the First Affiliated Hospital, Sun Yat-Sen University, Guangzhou, 519082 China; 5 Health Data Science Center, Shenzhen People’s Hospital, First Affiliated Hospital of Southern University of Science and Technology, Shenzhen, 518020 China; 6 School of Data Science, The Chinese University of Hong Kong (Shenzhen), Shenzhen, 518172 China; 7 Jinfeng Laboratory, Chongqing, 401329 China; 8 School of Biomedical Sciences, The Chinese University of Hong Kong, Shatin, New Territories, Hong Kong, 999077 China; 9 Shenzhen Research Institute, the Chinese University of Hong Kong, Shenzhen, 518172 China; 10 Department of Applied Data Science, Hong Kong Shue Yan University, North Point, Hong Kong Island, Hong Kong, 999077 China

**Keywords:** Spatially resolved transcriptomics, Tissue module, Spatial expression components, Bayesian model

## Abstract

Here we present STModule, a Bayesian method developed to identify tissue modules from spatially resolved transcriptomics that reveal spatial components and essential characteristics of tissues. STModule uncovers diverse expression signals in transcriptomic landscapes such as cancer, intraepithelial neoplasia, immune infiltration, outcome-related molecular features and various cell types, which facilitate downstream analysis and provide insights into tumor microenvironments, disease mechanisms, treatment development, and histological organization of tissues. STModule captures a broader spectrum of biological signals compared to other methods and detects novel spatial components. The tissue modules characterized by gene sets demonstrate greater robustness and transferability across different biopsies. STModule: https://github.com/rwang-z/STModule.git.

**Supplementary Information:**

The online version contains supplementary material available at 10.1186/s13073-025-01441-9.

## Background

Recent advances in spatially resolved transcriptomics (SRT) technologies enable profiling of gene expression along with spatial localization information in the tissue context [1], which facilitates exploration of tissue architecture, cell–cell communications, tumor heterogeneity and tumor microenvironment (TME) [2–6]. The technologies can be primarily categorized into imaging-based and next-generation sequencing (NGS)-based approaches [3]. Most of the imaging-based approaches offer targeted gene profiling at a high resolution of single-cell or single-molecule level [7, 8], e.g., smFISH [9], seqFISH [10], MERFISH [11], and STARmap [12]. However, imaging-based technologies are not unbiased due to region selection, prob design to profile gene targets of interest, etc. [3] The NGS-based approaches allow transcriptome-wide profiling by capturing transcripts in situ with spatial barcoding and sequencing ex situ. The spatial resolution varies a lot across different NGS-based technologies. For instance, Spatial Transcriptomics (ST) and Visium of 10 × Genomics profile a tissue section with spatially distributed spots of 100/55 µm diameter and 200/100 µm center-to-center distance [13]. The resolution is further enhanced in subsequent technologies, e.g., improved to 10 µm in diameter in Slide-seq and Slide-seqV2 [14, 15], 2 µm in HDST [16] and Visium HD [17], and below 1 µm in Stereo-seq [18] and Seq-Scope [19]. Although capturing transcriptome at cellular or subcellular level, they may not provide single-cell resolution as the spots could span two or more cells [7].

The transcriptomic landscapes of tissues are complex biological contexts consisting of interacting functional units and structures, termed “tissue modules” which are constituted by recurrent cellular communities spatially organized to exert specific functions [20]. While gene modules of single cells uncover expression programs and recurrent cell states at the cellular level [21, 22], tissue modules additionally account for spatial correlations in the contexts and represent expression components at the tissue level. They provide insights into spatial organization and interactions of essential biological processes in the landscapes and reveal pathological characteristics and histological structures of tissues [20]. Exploring tissue modules facilitates detecting crucial components and characteristics for downstream analysis, linking spatial expression with histological and pathological features, and advancing our understanding of the tissue landscapes especially the TMEs [3]. SRT preserving the spatial contexts of tissues enables identification of tissue modules; however, there are some challenges. Firstly, it is hard to disentangle the tissue modules as they are likely to be convoluted with each other in the spatial profiles due to low sequencing resolutions and overlapping genes involved in different functions [20, 23]. Secondly, biological processes and tissue architectures are arranged in multiple scales encompassing both higher-order structures and finer components, leading to modules with varying granularities in the same tissue [24, 25]. Thirdly, although the expression variances between cells are dominated by cell types, there are also properties shared across cell types revealing biological processes such as metabolism, immune response, differentiation, and growth, which are not detected by cell-type-centric methods [23]. Furthermore, as joint optimization of related tasks contributes to more accurate results [26, 27], methods that capturing tissue modules by jointly estimating their spatial distributions and associated genes are required.

Computational methods have been developed for different purposes of SRT analysis, including spatially variable gene (SVG) detection [1, 28], spatial domain clustering [6, 29–31], and cell type deconvolution [32–34], which differ from tissue module detection. SVG detection methods identify genes that exhibit statistically significant spatial expression patterns across the tissue, serving as feature selection tools in data pre-processing. They may additionally summarize representative expression patterns of the SVGs which suggest potential tissue modules. However, the SVGs are not guaranteed with spatial patterns [29] and the resulting expression patterns substantially rely on the success of SVG detection. Spatial domain clustering methods group the profiled spots/cells within a tissue section into domains with high coherence in both gene expression and spatial distribution. While they have demonstrated efficacy in dissecting histological and anatomical structure of tissues [29] and tissue annotation [6], they struggle to disentangle convoluted and multi-scale structures [20]. Cell type deconvolution methods have been developed to estimate the composition of distinct cell types/subtypes for spots based on single-cell references or cell-type markers, which facilitate investigation of spatial distributions and interactions of particular cell types/subtypes in the tissue context and thereby play important roles in understanding tumor heterogeneity and TME [35, 36]. As they focus on cell types/subtypes present in the single-cell references, the biological signals shared across cell types and unique characteristics of tissues might be overlooked in the analysis. In addition, there are also some other tissue-centric approaches tailored for SRT data, including those investigating spatial cell–cell communications [37, 38], integrating or aligning multiple slides for clustering or three-dimensional reconstruction [39, 40], etc. Although the methods have demonstrated promising results in respective tasks, they are not well-suited for tissue module detection that seeks to explore the constitutional components and expression signals within transcriptomic landscapes.

In this study, we developed STModule, a computational method for detecting tissue modules. Using a Bayesian model, STModule identifies spatial expression components within tissue transcriptomic landscapes by simultaneously estimating their spatial maps (i.e., spatial distributions over tissues) and associated genes. It is tissue-centric rather than cell-type-centric, promising to detect diverse expression signals including novel molecular features and offer unique pictures of the tissue contexts. As tissue modules are detected in an unsupervised and data-driven manner according to underlying spatial expression patterns, the granularities of modules are automatically determined, so that the modules reveal biological processes of multiple scales. STModule also accounts for spatial expression correlations among genes and groups related genes into tissue modules, instead of analyzing individual genes as in previous studies. In addition, STModule allows overlapping spatial maps and associated genes across distinct modules, addressing the issue of convoluted biological processes and signals, and is applicable to SRT data with various spatial resolutions. We evaluated the effectiveness of STModule with both simulated data and real-world datasets of a variety of tissue types and cancer subtypes profiled by different SRT technologies, including ST datasets of human pancreatic ductal adenocarcinoma (PDAC), breast cancer (BC), prostate cancer (PC) and melanoma, 10 × Visium data of human dorsolateral prefrontal cortex (DLPFC), Slide-seqV2 data of mouse hippocampus, and mouse olfactory bulb (MOB) datasets profiled by ST, Slide-seqV2, and Stereo-seq respectively. The results indicate that STModule is able to dissect spatial organization and interactions of essential components in the transcriptomic landscapes and uncover histological and pathological characteristics of tissues, providing insights into tumor microenvironments, disease mechanisms, and treatment development.

## Methods

### STModule

**Fig. 1:**
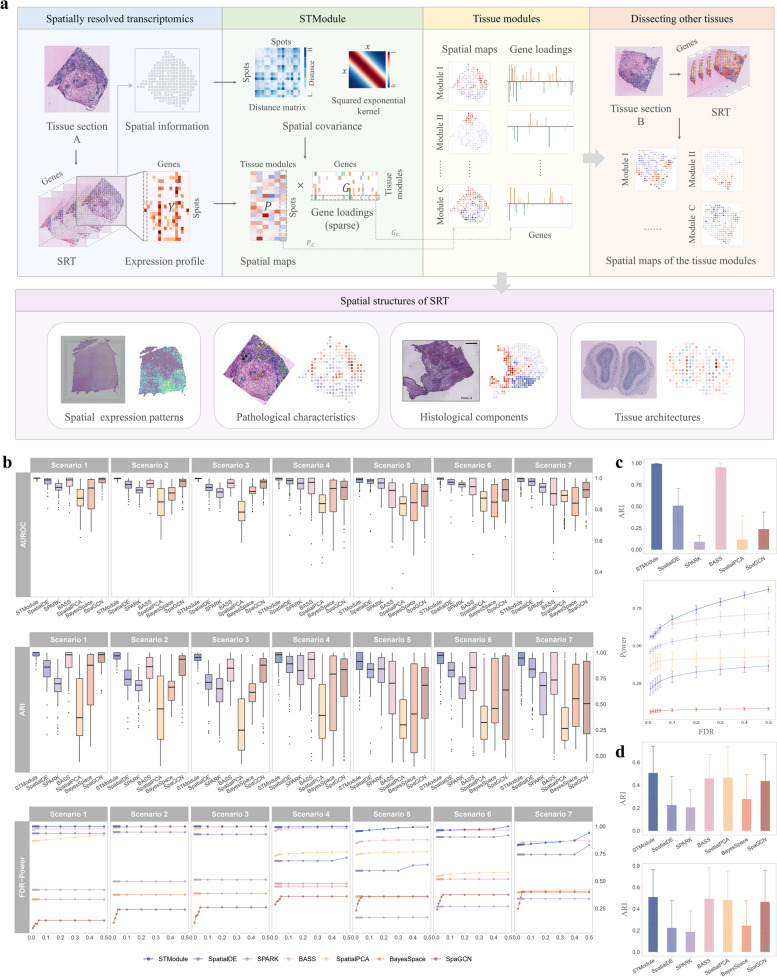
Overview of STModule and simulation results.** a** STModule identifies spatially distributed tissue modules with the gene expression profile ($Y$) and spatial information of the profiled spots. **b** Evaluation and comparison of different methods in seven scenarios of the first set of simulations, including AUROC (top) and ARI (middle) for spatial pattern identification and FDR-Power curve for associated gene detection (bottom). The *x* and *y* axes of the FDR-Power curve represent FDR and Power, respectively. **c** Evaluation and comparison of the second set of simulations for layer-wise DLPFC data. Top, ARI of layer identification. Bottom, FDR-Power curve of layer-associated gene detection. **d** Comparison of ARI for layer identification in the third set of simulations for tissue-wise DLPFC data. Top, tissue-based simulations. Bottom, domain-based simulations

STModule identifies tissue modules that represent functional units and structures by dissecting underlying spatial components of a single slice/sample. To cope with SRT data of various spatial resolutions and to detect general as well as tissue-specific spatial components, the problem is modeled as spatial Bayesian factorization, which enables uncovering underlying structures and latent factors from noisy data [41, 42]. For a tissue section profiled by a SRT technology, gene expression levels at different locations (spots/cells) are available, together with the coordinates of the locations. The expression profile of $L$ genes at $S$ spots is represented as a matrix $Y\in {\mathbb{R}}^{S\times L}$ (Fig. 1a). STModule factorizes the profile into $C$ tissue modules by simultaneously grouping co-expressed genes and estimating their spatial patterns, which indicate the associated genes ($G\in {\mathbb{R}}^{C\times L}$) and spatial maps ($P\in {\mathbb{R}}^{S\times C}$) of the modules:

1 $$P\left(\left.{Y}_{sl}\right|\theta \right)=\mathcal{N}\left({Y}_{sl}|{\sum }_{c}{P}_{sc}{G}_{cl},{{\lambda }_{s}}^{-1}\right)$$

where $l\in \left\{1,\cdots ,L\right\}$, $s\in \left\{1,\cdots ,S\right\}$, $c\in \left\{1,\cdots ,C\right\}$, and $\theta$ represents the set of all variables in the model. ${P}_{sc}$ represents the spatial activity of module $c$ at spot $s$. ${G}_{cl}$ indicates the loading of gene $l$ in module $c$. ${\lambda }_{s} \sim Gamma\left(u, v\right)$ models the noise of gene expression in spot $s$.

As neighboring spots are likely to express more similar transcriptomic signals than spots that are far away, and the spatial maps should be continuous and smooth, STModule accounts for spatial covariance of the spots based on squared exponential (SE) kernel and Euclidean distance between the spots:

2 $$P\left(\left.{P}_{.c}\right|{\sigma }_{c},{\Sigma }^{\left(c\right)}\right)=\text{MVN}\left({P}_{.c}|0,{{\sigma }_{c}}^{-1}{\Sigma }^{\left(c\right)}\right)$$

3 $${\Sigma }_{s,{s}{\prime}}^{\left(c\right)}=exp\left(-\frac{{d\left(s,{s}{\prime}\right)}^{2}}{2{l}_{c}^{2}}\right)=exp\left(-\frac{1}{2}{d\left(s,{s}{\prime}\right)}^{2}{r}_{c}\right)$$

where ${\Sigma }^{\left(c\right)}\in {\mathbb{R}}^{S\times S}$ represents the covariance matrix of module $c$. ${\sigma }_{c}\sim Gamma(m,n)$ is the scaling factor of module $c$ to control the degree of spatial covariance the module follows, allowing tissue modules with either smooth and continuous spatial distributions or discrete spatial maps highly activated at individual locations. Covariance between spots $s$ and ${s}{\prime}$ in module $c$ (i.e., ${\Sigma }_{s,{s}{\prime}}^{\left(c\right)}$) is determined by their Euclidean distance $d\left(s,{s}{\prime}\right)$ calculated based on their $x$ and $y$ coordinates in the tissue and the length scale ${l}_{c}$ of the module. The length scale represents the decay rate between the covariance and distance. Rather than using empirically determined constant values as in previous studies, the length scale of each module is automatically inferred by the algorithm according to the latent spatial structures. For convenience, ${l}_{c}^{2}=1/{r}_{c}$ is used in the model and ${r}_{c}\sim Gamma(a,b)$.

To identify associated genes for each module, spike and slab prior [43] is used to induce a sparse structure for matrix $G$, grouping genes with similar underlying patterns into the same module:

4 $${G}_{cl}\sim {p}_{cl}\mathcal{N}\left({G}_{cl}|0,{{\beta }_{c}}^{-1}\right)+\left(1-{p}_{cl}\right){\delta }_{0}\left({G}_{cl}\right)$$

where ${p}_{cl}$ is the mixing weight between the Gaussian and the point mass, ${\beta }_{c}\sim Gamma(e,f)$ is the precision of the Gaussian distribution and ${\delta }_{0}(\cdot )$ denotes the Dirac delta function centered at zero. For efficient inference of the model, we followed Hore et al. [44] and re-parameterized it as follows:

5 $${G}_{cl}={\omega }_{cl}{s}_{cl}$$

6 $${\omega }_{cl}\sim \mathcal{N}\left({\omega }_{cl}|0,{{\beta }_{c}}^{-1}\right)$$

7 $${s}_{cl}\sim Bernoulli\left({s}_{cl}|{\psi }_{cl}{\phi }_{cl}\right)$$

where ${\psi }_{cl}\sim Beta\left(g,h\right)$, ${\phi }_{cl}\sim Bernoulli\left({\rho }_{c}\right)$, and ${\rho }_{c}\sim Beta\left(t,z\right)$. The gene loadings in $G$ indicate the contributions of the genes in the tissue modules. In addition, the spike and slab prior allows automatically selection of the model complexity, i.e., the number of tissue modules, by generating zero vectors in the factor matrices when $C$ is large enough.

The model was inferred using Variational Bayes (VB) [45], which finds the setting of parameters for a selected family of distributions $Q\left(\Theta \right)$ that is closest to the posterior distribution by minimizing the Kullback–Leibler (KL) divergence between $Q\left(\Theta \right)$ and $P\left(\left.\theta \right|Y\right)$. The full model and updates of the variables are introduced in Additional file 1: Supplementary notes. The membership of a gene in a tissue module was determined by posterior inclusion probabilities (PIP), calculated as ${E}_{Q}\left({s}_{cl}\right)$, which indicates the probability of that a variable is included in the true model [44]. Gene $l$ is associated to module $c$ if ${E}_{Q}\left({s}_{cl}\right)>0.5$. To visualize spatial maps of the tissue modules, the estimated activities ${E}_{Q}\left({P}_{.c}\right)$ were scaled to a vector with zero-mean and variance of one for ST and 10 × Visium data, and log-transformed while keeping the sign of activities for Slide-seqV2 and Stereo-seq data.

### Simulations

To quantitatively and comprehensively evaluate STModule, we conducted four sets of simulations based on spatial patterns, DLPFC layouts and real data, respectively.

In the first set of simulations, we generated expression profiles to simulate spatial patterns of seven different scenarios following the data generative schemes in [1, 46, 47] (Additional file 1: Supplementary notes and Additional file 2: Fig. S1). In scenarios 1–3, we simulated one gene set expressing a simple spatial pattern (*scenario 1*) or a pattern generated with two (*scenario 2*) or three (*scenario 3*) basic patterns to investigate spatial patterns of simple and complex shapes. In scenarios 4 and 5, we simulated two gene sets each expressing a spatial pattern without (*scenario 4*) or with (*scenario 5*) overlapping genes to imitate independent and related biological signals, respectively. In scenarios 6 and 7, we simulated gene sets expressing multi-scale spatial patterns to represent biological concepts in different levels or granularities. The expression profiles were simulated based on the layout of ST data, each containing the expression of 1,000 genes at 256 spots equally distributed on a $16\times 16$ square grid. The spatial patterns were generated using basic spot and linear patterns following the strategy of each scenario.

In the second set of simulations, we generated layer-wise patterns based on the layout of 10 × Visium DLPFC following SRTsim [48], simulating the spatial expression of layer-specific genes. For each of the seven layers, we generated 10 simulated profiles, each including 100 spatially expressed genes that were highly expressed in the layer spots and 900 non-spatially expressed genes with random expression across the tissue. The number of spots for each profile was 3,611. The expression of genes was simulated using negative binomial distributions with the mean and dispersion parameters recommended by SRTsim (Additional file 1: Supplementary notes).

In the third set of simulations, we used SRTsim to generate whole-tissue expression profiles of 10 × Visium DLPFC based on real data with two settings—tissue-based and domain-specific scenarios. The expression profiles were directly generated across all spots of the tissue in the tissue-based scenario, while in the domain-specific scenario, the expression data was first simulated for each domain separately and then combined to generate the whole profile. Each profile consisted of expressions of 80 genes on 3,611 spots.

In the fourth set of simulations, we simulated real sagittal mouse brain data profiled by 10 × Visium using scDesign3 [49] to evaluate the performance of SVG detection. We followed the tutorial of the method for benchmarking differential expression analysis in SRT data and generated 10 expression profiles along with the labelled SVGs as ground truth. In particular, we identified SVGs from the raw data using FindSpatiallyVariableFeatures function of Seurat [50] package with Moran’s I method and set the mean expression of the non-spatially variable genes (NSVGs) in the simulated profiles to that of non-variable features of the raw data. Each simulated profile consisted of expressions of 995 genes including 50 SVGs and 945 NSVGs at 2,696 spots. The simulations are summarized in Additional file 3: Table S1.

### SRT data collection and preprocessing

The count matrices and respective hematoxylin and eosin (H&E) images of human BC [13], PC [5], melanoma [51], and MOB [13] profiled by Spatial Transcriptomics were downloaded from the data repository provided by Spatial Research (https://www.spatialresearch.org/resources-published-datasets/) [52–54]. The BC dataset [52] consists of SRT data of four sections (layers 1–4) collected from the same biopsy, profiling 14,789 ~ 14,929 genes at 251 ~ 264 spots. For the PC dataset [53], we selected two representative samples with cancer areas (P1.2; 17,678 genes, 406 spots) and inflammation regions (P4.2; 17,781 genes, 324 spots), respectively. For the melanoma dataset [54], replicate 2 of biopsy 1 (16,148 genes, 293 spots) and replicate 1 of biopsy 2 (16,831 genes, 383 spots) were included in the analysis. Replicate 11 (16,218 genes, 262 spots) of the ST MOB dataset [52] was used following previous studies [1, 28, 47]. ST data of human PDAC [4] was downloaded from Gene Expression Omnibus (GEO) with accession number GSE111672 [55]. We analyzed samples PDAC-A and PDAC-B, which quantified 19,738 genes at 428 and 224 spots respectively. The human DLPFC dataset [56] profiled by 10 × Visium was downloaded using R package spatialLIBD [57]. It contains SRT data of 12 sections collected from 3 individuals, profiling 33,538 genes at 3,460 ~ 4,789 spots. The SRT data of mouse hippocampus and MOB profiled by Slide-seqV2 [15] were downloaded from Single Cell Portal [58]. The mouse hippocampus data quantified 19,653 genes at 20,143 locations, while the MOB data profiled 21,220 genes at 21,724 locations. The processed count matrix of Stereo-seq MOB data [18] was downloaded from SEDR [59], comprising expression of 27,106 genes at 19,527 locations. The datasets used in this study are summarized in Additional file 3: Table S2.

Genes expressed in less than 10% spots were removed for BC, PDAC, melanoma, ST MOB, and DLPFC data. Due to more sparse count matrix compared to other ST datasets, genes expressed in less than 5% spots were removed for the PC samples. For high-resolution datasets profiled by Slide-seqV2 and Stereo-seq, genes expressed in less than 50 locations were filtered out and cells expressing less than 100 and 200 genes were removed, respectively. The count matrices were normalized using NormalizeData function of Seurat with the method of relative abundance and a scaling factor of $1e4$. The top 2,000 highly variable genes (HVGs) were selected using FindVariableFeatures function of Seurat with the “vst” method, except the Stereo-seq MOB dataset, for which we used the combination of top 1,000 HVGs selected by Seurat and genes included in the tissue modules identified from MOB profiled by ST and Slide-seqV2.

### Application of different methods

We compared STModule with two SVG detection methods SpatialDE [28] and SPARK [1, 60], which are capable of identifying spatial patterns, as well as four representative domain clustering methods BASS [30], SpatialPCA [31], BayesSpace [6], and SpaGCN [29], each with distinct advantages. BASS is a Bayesian method and able to perform multi-scale transcriptomic analysis. SpatialPCA extends conventional principal component analysis (PCA) to the SRT data taking into account the spatial correlations of the spots/cells. BayesSpace is able to enhance the resolution of clustering which might dissect convoluted signals. SpaGCN is based on graph convolutional neural network and integrates histological image with SRT data to improve the performance. For SpatialDE and SPARK, we followed the methods specified in the original studies to identify spatial patterns after SVG detection. For domain clustering methods, differentially expressed genes of each cluster were identified following the corresponding studies.

For simulated data, in the first set of simulations, the number of modules/patterns/clusters for the methods was set to the number of spatial patterns in each profile + 2 to allow flexibility, i.e., 3 modules/patterns/clusters for scenarios 1, 2 and 3, 4 modules/patterns/clusters for scenarios 4 and 5, and 4 and 5 modules/patterns/clusters for scenarios 6 and 7, respectively. In the second set of simulations for layer-wise DLPFC data, as the spots of each simulated profile were labelled as a particular layer or others, we set the number of modules/patterns/clusters to 2. In the third set of simulations for whole-tissue DLPFC data, the number of modules/patterns/clusters was set to the number of layers. In the fourth set of simulations for SVG detection, the number of modules/patterns for STModule, SpatialDE, and SPARK was set to 10, while the number of clusters for the domain clustering methods was set to 15 as demonstrated in Seurat example for the same dataset. BayesSpace failed to run on the second set of simulations, thus not compared to the other.

For real datasets, the number of tissue modules for STModule, SpatialDE, and SPARK was set to 10 in the main analysis for all datasets as 10 modules could reveal major expression components [1, 28]. SpatialDE was only applied to ST and 10 × Visium data due to the considerable computational time for high-resolution data. For SPARK, we applied spark.test for ST and 10 × Visium data and sparkx for high-resolution data. For the domain clustering methods, we used the number of clusters determined by BayesSpace qtune for the ST datasets. The number of clusters for the DLPFC dataset was set to the number of layers of the tissue sections. As for high-resolution datasets, we set the number of clusters to that used in the corresponding study or online tutorial of the methods if available, otherwise using the number of clusters recommended by BayesSpace qtune.

### Evaluation of the methods

For simulated data, the ability to identify spatial patterns was assessed by area under the receiver operating characteristic (AUROC) curve and area under the precision-recall (AUPR) curve using R package PRROC [61], as well as adjusted rand index (ARI) using R package mclust [62], where ARI for STModule, SpatialDE, and SPARK was calculated with a sequence of thresholds and the highest ARI was demonstrated to indicate their best discriminative ability. For the simulated DLPFC data, methods were evaluated in a layer-wise manner. The performance of identifying associated genes or SVGs was evaluated by FDR-Power curve.

For the real DLPFC dataset [56] with spot annotations indicating their respective layers, we mapped the detected tissue modules, spatial patterns, and clusters with the annotated structures. In the case of SpatialDE and SPARK, spatial pattern with the highest AUROC was selected as the aligned pattern for each layer. As for the domain clustering methods, i.e., BASS, SpatialPCA, BayesSpace, and SpaGCN, the one-to-one mapping of clusters to layers with the highest average AUROC over all permutations was selected for each sample. AUPR and ARI were calculated afterwards for each aligned pair of module/pattern/cluster and layer. In addition, we identified differentially expressed genes (DEGs) for each layer of each sample using FindVariableFeatures of Seurat R package and performed gene set enrichment analysis (GSEA) of the associated genes detected by the methods towards the layer-specific DEGs.

For the other real datasets without spot-/cell-level annotations, we performed cell type deconvolution using CARD [33] to estimate spatial distributions of different cell types as references for evaluation. Cell types related to the histological domains or structures annotated in the original studies of the datasets were included for further evaluation so that they were cross validated by both sources. Moreover, to reduce the effects of noise and focus on more reliable domains estimated by CARD, cell types with less than 0.5 proportions across all spots/cells were excluded. We evaluated Pearson correlations of the cell type proportions and spatial patterns identified by STModule, SpatialDE, and SPARK. Compared with domain clustering methods, for each cell type, the spots/cells with higher than 0.5 proportions were labelled as 1 while the others as 0. We evaluated AUROC, AUPR, and ARI for all methods on these binarized spatial domains. GSEA comparing the associated genes detected by the methods and the cell-type markers identified from single-cell references using Seurat was also conducted for these datasets.

### Cell type deconvolution

Cell type deconvolution for the real datasets was conducted using CARD [33] with default settings. The single-cell references for PDAC [4], BC [35], PC [63], melanoma [64], and MOB [65] were collected from GEO with accession number GSE111672 [55], GSE176078 [66], GSE181294 [67], GSE115978 [68], and GSE121891 [69], respectively. For the single-cell reference for mouse hippocampus [70], we used the processed data provided by CARD [71].

### Gene set enrichment analysis

GSEA was performed using gost function of R package gprofiler2 [72]. Layer-associated genes or cell-type markers were used to construct Gene Matrix Transposed (GMT) files as the annotation data for GSEA. Ordered queries were conducted based on the gene loadings, memberships, or adjusted *p*-values. As for functional enrichment analysis of tissue modules identified by STModule, the top 200 associated genes of each module demonstrating the highest loadings with the same direction as the annotated spatial map were included. Enrichment of Gene Oncology (GO) terms, including biological process (BP), molecular function (MF) and cellular component (CC), Kyoto Encyclopedia of Genes and Genomes (KEGG) pathways, and Reactome pathways were analyzed.

### Comparison of tissue modules

For sample A in the PDAC dataset, we investigated the results of STModule for identifying different numbers of tissue modules. The modules were compared in terms of their associate genes by hypergeometric test to estimate the similarity between them using phyper function in R. The top 50 associated genes demonstrating the highest loadings with the same direction as the annotated spatial map were included for comparison. The top 2,000 HVGs of the sample used as input of STModule were utilized as background genes.

### Application of tissue modules to other sections

Suppose tissue modules were identified from section A with STModule, i.e., $P\left({Y}_{sl}^{A}\left|\theta \right.\right)=\mathcal{N}\left({Y}_{sl}^{A}|{\sum }_{c}{P}_{sc}^{A}{G}_{cl}^{A},{{\lambda }_{s}^{A}}^{-1}\right)$, where ${Y}^{A}$ represents the processed expression profile of A. When applying the modules to another section B, the spatial expression profile of section B was first normalized as mentioned above. The HVGs in section A were used to filter the normalized expression matrix of section B so that the resulting profile ${Y}^{B}$ included the spatial expression of a gene set same as ${Y}^{A}$. The factor matrix $G={E}_{Q}\left({G}^{A}\right)$ derived from section A indicating gene loadings in the modules was used and fixed in the factorization of ${Y}^{B}$, i.e., $P\left({Y}_{sl}^{B}\left|\theta \right.\right)=\mathcal{N}\left({Y}_{sl}^{B}|{\sum }_{c}{P}_{sc}^{B}{G}_{cl},{{\lambda }_{s}^{B}}^{-1}\right)$, to estimate spatial maps of the tissue modules for section B.

## Results

### STModule recovers simulated spatial components

As most public real datasets lack comprehensive and precise annotations, it is challenging to thoroughly evaluate and compare different methods for dissecting spatial components. Therefore, we conducted four sets of simulations to simulate spatial expression in various situations (Methods).

In the first set of simulations for spatially expressed patterns, STModule outperforms other methods in spatial pattern identification and is superior or comparable to BASS in detecting associated genes (Fig. 1b and Additional file 2: Fig. S2a, b). Specifically, in scenarios 1–3, most methods exhibit a decline in both AUROC and ARI as the patterns become more complex, while STModule remains relatively stable. In scenarios 4 and 5, the methods demonstrate better performance in scenario 4 for both spatial pattern and associated genes, suggesting that disentangling related or convoluted biological signals is more challenging than identifying independent ones. In scenarios 6 and 7, the methods also perform better in the simpler case of scenario 6, which features two multi-scale patterns, compared to scenario 7 with three patterns, particularly regarding associated gene detection. Examples of simulated spatial patterns in different scenarios and corresponding patterns/clusters identified by different methods are illustrated in Additional file 2: Fig. S3. In the second set of simulations focused on DLPFC layer-wise patterns, STModule and BASS far surpass the other methods in distinguishing the layers and their associated genes (Fig. 1c and Additional file 2: Fig. S2c). In addition, most methods struggle to perform well in both tasks across the two simulation sets, demonstrating a trade-off between them. For instance, SpatialPCA achieves better performance in detecting associated genes compared to BayesSpace and SpaGCN but is less effective in pattern identification.

In the third and fourth sets of simulations, we evaluated the methods on DLPFC and mouse brain profiles simulated based on real data for pattern identification and SVG detection, respectively. STModule also better discriminates the layers of DLPFC compared to the others and ranks just behind SpatialDE in SVG detection even though it is not tailored for this task (Fig. 1d and Additional file 2: Fig. S2d, e). As in the first set of simulations, it is more difficult to differentiate layers in whole-tissue profiles with more intricate structures (STModule, avg. ARI 0.49) than layer-wise profiles (STModule, avg. ARI 0.99). The superior performance of STModule across the sets of simulations in both spatial pattern and associated gene detection highlights the benefits of integrating these tasks to better unravel the intricate structures and characteristics of tissues, where the spatial patterns guide the algorithm in retrieving the corresponding genes while the detected genes, in turn, help refine the patterns iteratively.

### STModule reveals pathological and histological characteristics of PDAC

**Fig. 2:**
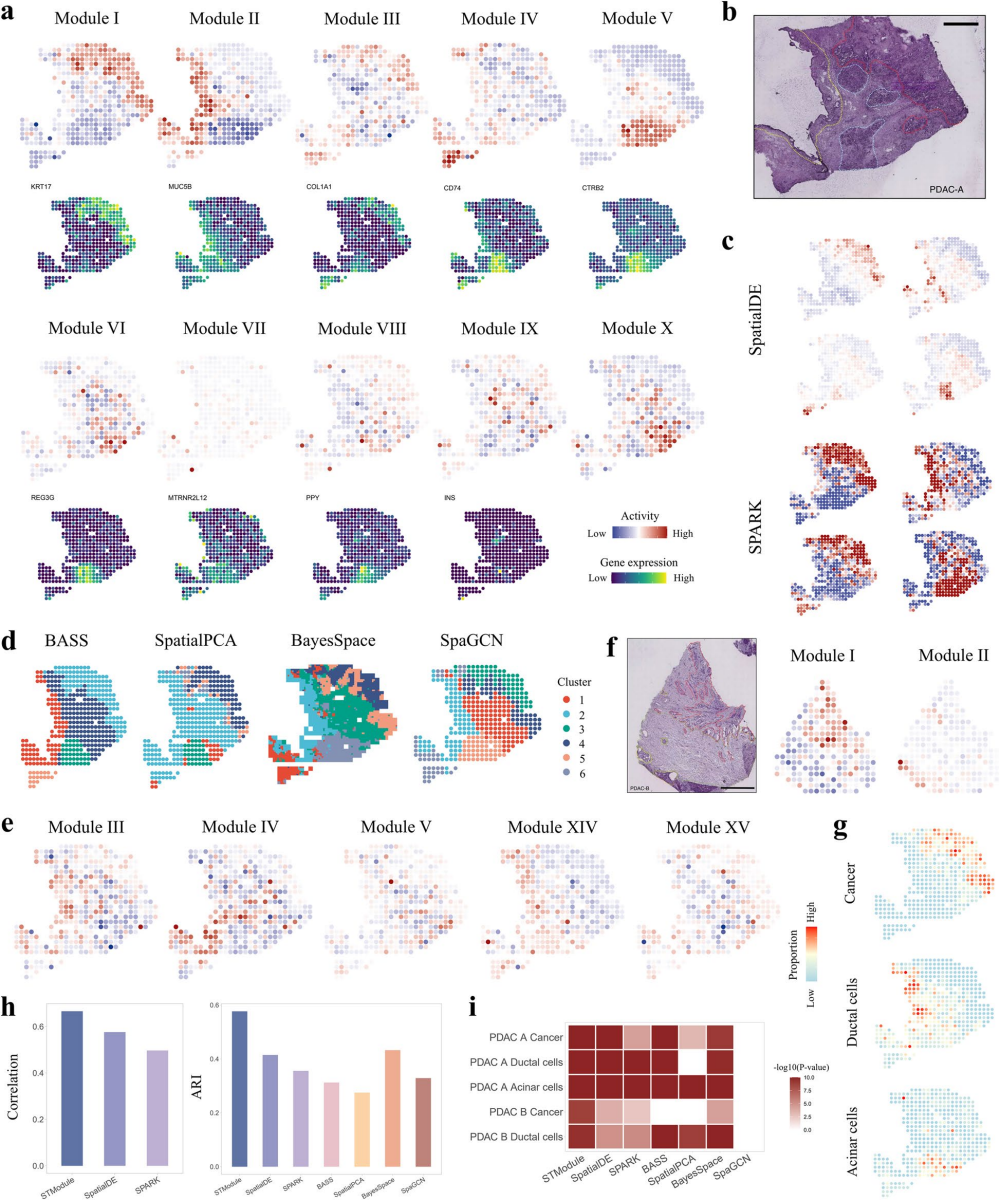
Results of the human pancreatic ductal adenocarcinoma dataset.** a** Spatial maps of tissue modules identified by STModule and spatial expression of representative associated genes. Colors indicate module activities or gene expression levels at different spots. **b** Histological annotation of sample A in the original study [4]. Red, cancer cells and desmoplasia. Yellow, duct epithelium. Blue, normal pancreatic tissue. **c** Representative spatial patterns identified by SpatialDE and SPARK. **d** Results of domain clustering methods. Colors indicate different clusters. **e** Additional spatial patterns identified by STModule with 15 modules. **f** Histological annotation of sample B in the original study [4] (left) and two tissue modules identified by STModule. Red, cancer cells and desmoplasia. Yellow, duct epithelium. Green, interstitium. **g** Spatial distributions of major cell types of sample A estimated by CARD. **h** Comparison of the methods in terms of correlation and ARI of identifying domains. **i** GSEA of associated genes detected by the methods. White color indicates adjusted *P*-value $\ge$ 0.05

To investigate the efficacy of STModule in dissecting tissue modules in real SRT datasets, we first applied it to a human PDAC sample (PDAC-A) profiled by ST [4] (Fig. 2). The tissue modules were interpreted according to the annotations in the original study, signatures identified in previous studies [36, 73, 74] and examination of the H&E-stained image by experienced pathologists (Fig. 2a, b and Additional file 2: Fig. S4). The spatial map of module I aligns well with the cancer regions, as well as the spatial expression of module-associated genes such as KRT17, TM4SF1, and S100A4, which have been reported as PDAC markers [36, 73, 74] (Additional file 2: Fig. S5). Module II represents duct epithelium, expressing high levels of ductal cell markers including MUC5B, DMBT1, and CRP [33, 36]. Module III is enriched with genes of cancer-associated fibroblasts (CAFs) like COL1A1, COL1A2, and COL3A1, distributing spatially around cancer cells for tumorigenesis promotion [75]. Module X is annotated as stroma based on the H&E-stained image (Additional file 2: Fig. S6a).

Furthermore, STModule detects tissue modules indicative of cell subtypes with particular biological signals, including antigen-presenting CAFs (apCAFs) (module IV) expressing both CAF markers and major histocompatibility complex class II (MHC II) genes such as CD74 [76], and subtypes of endocrine, including alpha cells (VIII) and beta cells (IX) located in line with islets in the H&E-stained image (Additional file 2: Fig. S6a). Module V represents acinar cells distinguished by high expression of CTRB1, CTRB2, and PRSS2 [36], while module VI identifies a subset (acinar REG −) with low expression of REG gene family, e.g., REG1A and REG3G, leaving the others as acinar REG + that promote acinar-to-ductal metaplasia (ADM) and pancreatic intraepithelial neoplasia (PanIN) in PDAC [36] (Additional file 2: Fig. S4 and Fig. S5). STModule also highlights another spatial pattern (VII) presented by a set of MT-RNR2-like genes, including MTRNR2L12, MTRNR2L1, and MTRNR2L8, which encode a mitochondrial-derived peptide humanin that suppresses cell apoptosis, acts as a cytoprotective factor, and improves function of pancreatic beta cells [77, 78]. Specifically, as an intracellular signal, humanin exerts its cytoprotective effects by interacting with proteins of the Bcl-2 family to inhibit the intrinsic or mitochondrial apoptotic pathway [79]. It has also been reported to act as a therapeutic target of cancers and degenerative diseases [79, 80]. Functional analysis of the tissue modules in terms of GO terms, KEGG pathways, and Reactome pathways shows agreement with the above annotations (Additional file 2: Fig. S7 and Fig. S8).

We applied SpatialDE, SPARK, BASS, SpatialPCA, BayesSpace, and SpaGCN to the same tissue for comparison (Fig. 2c, d, and Additional file 2: Fig. S9), as well as CARD for cell type deconvolution (Additional file 2: Fig. S6b). SpatialDE and SPARK detect spatial patterns of cancer cells, duct epithelium, acinar cells, and CAFs, while spatial domain clustering methods outline the basic structure organization of the tissue. It is challenging for the clustering methods to distinguish between the convoluted signals caused by the low resolution of ST data, for instance, the co-occurrence and overlapping distributions of cancer cells and CAFs. BayesSpace performs better as it offers enhanced clustering with improved resolution. The spatial distributions of tissue modules identified by STModule, which represent cell types such as acinar cells, fibroblasts, ductal cells, and endocrines, are concordant with the results of CARD, indicating that although not educated with prior knowledge, STModule is able to uncover them through spatial expression patterns.

We further explored the outcomes of STModule by factorizing the tissue into 5 and 15 modules respectively and compared them to the 10-module results. With 5 modules, STModule detects major components of the tissue including cancer cells, duct epithelium, CAFs, acinar cells, and spatial expression of MT-RNR2-like genes (Additional file 2: Fig. S10), which comprise a subset of the 10-module results. Expanding to 15 modules, STModule identifies additional spatial structures representing subtypes of ductal cells including centroacinar (III; AQP3), terminal (IV; TFF1, TFF2, TFF3), and antigen-presenting (V; CD74) [4], pancreatic glands (XIV), and macrophages (XV) (Fig. 2e and Additional file 2: Figs. S11-S14). Overlapping modules aligned to the same structures in the three settings show significantly high consensus of associated genes (Additional file 2: Fig. S15). Modules linked to the same cell type also demonstrate higher similarity due to shared basic functions and biological pathways, e.g., CAFs vs. apCAFs, acinar cells vs. acinar cells (REG −), and subtypes of ductal cells.

In addition, applied to another sample (PDAC-B) in this dataset, STModule detects cancer cells, fibroblasts and acinar cells, and subtypes of ductal cells, as well as atypical ductal cells which suggest a progression from ductal cells to malignancy [73] (Fig. 2f and Additional file 2: Figs. S16-S20). Results of comparison methods are illustrated in Additional file 2: Fig. S21a, b. Based on the spatial distributions of cell types related to the histology features of the two samples estimated by CARD (Fig. 2g and Additional file 2: Fig. S21c), we evaluated the methods in identifying these features as well as associated genes (Methods). STModule demonstrates the highest correlation, ARI, AUROC, and AUPR in identifying the domains and the detected associated genes of the domains are enriched with corresponding cell type markers (Fig. 2h, i and Additional file 2: Fig. S21d). Particularly, higher correlations between the tissue modules and domain-related cell types indicate that STModule not only identifies the spatial locations of the domains, but also better preserves their strength across the landscape. The results suggest that STModule effectively identifies tissue modules from SRT data, capturing histological structures and pathological signals of tissues.

### STModule detects modules applicable across different sections of breast cancer

**Fig. 3:**
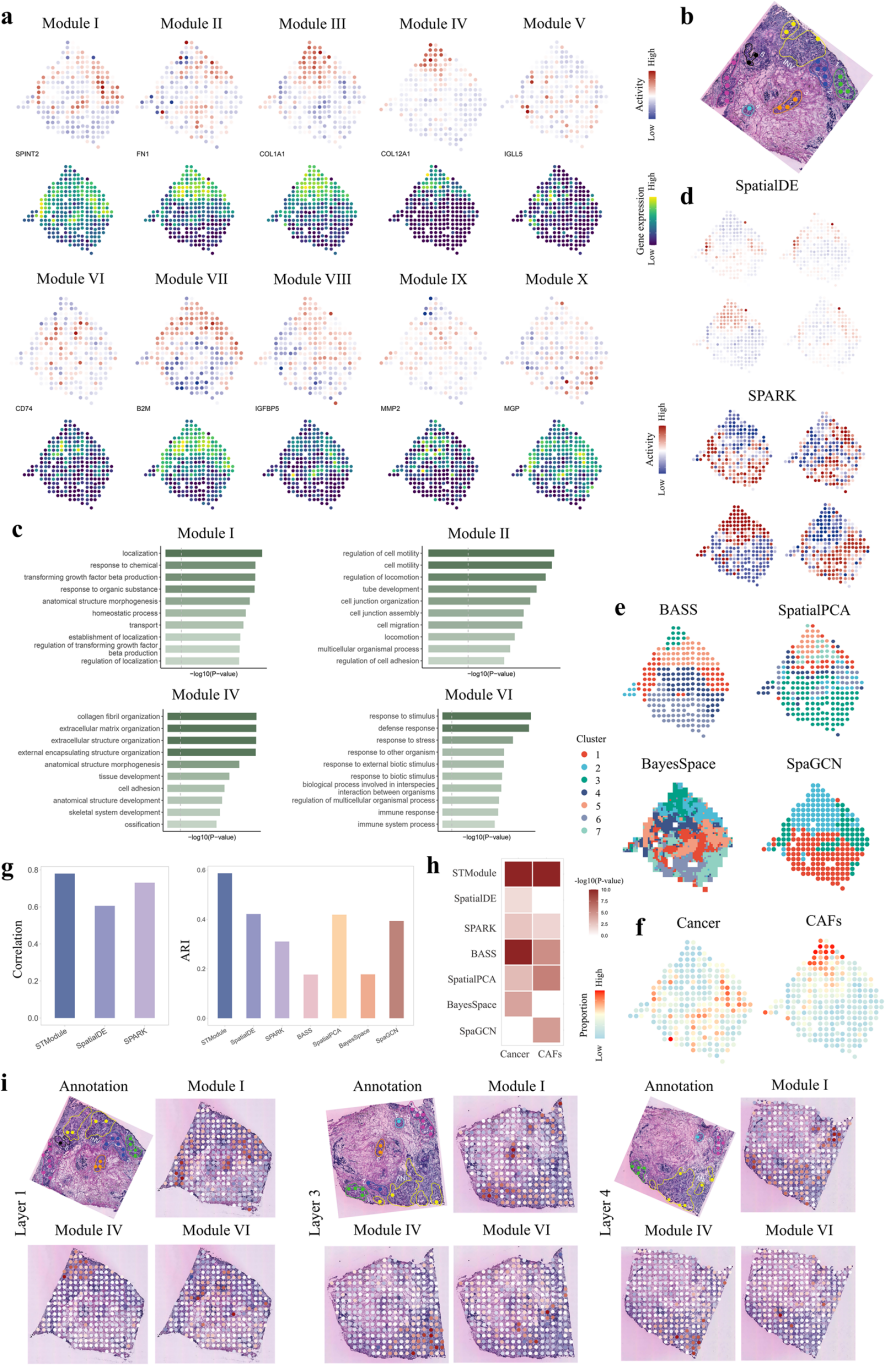
Results of the human breast cancer dataset.** a** Spatial maps of tissue modules identified by STModule and spatial expression of representative associated genes. Colors indicate module activities or gene expression levels at different spots. **b** Histological annotation of the tissue section in the original study [13], containing invasive ductal cancer (INV) and six areas of ductal cancer in situ (DCIS) (1 to 6). **c** Illustration of most enriched Gene Oncology terms of representative modules. Color indicates the value of − log_10_(*P*-value). Dashed grey lines represent the threshold of *P*-value = 0.05. **d** Representative spatial patterns identified by SpatialDE and SPARK. **e** Results of domain clustering methods. Colors indicate different clusters. **f** Spatial distributions of cancer and CAFs estimated by CARD. **g** Comparison of the methods in terms of correlation and ARI of identifying domains in **f**. **h** GSEA of associated genes detected by the methods. White color indicates adjusted *P*-value $\ge$ 0.05. **i** Application of the tissue modules to the other three sections collected from the same biopsy. Spatial maps of modules I, IV, and VI in **a** are illustrated with manual alignment to the corresponding H&E-stained images

We applied STModule to a breast cancer dataset profiled by ST [13]. The original biopsy was sliced at 16 $um$ thickness, and every fourth slice was selected for SRT profiling, resulting in four quantified sections labelled as layers 1–4. Applied to layer 2, STModule identifies 10 modules annotated as ductal carcinoma in situ (DCIS) (I), invasive ductal cancer (INV) (II), CAFs (III and IV), immune-related signals (V, VI and VII), perivascular cells (VIII), endothelial cells (IX), and normal breast glands (X), respectively (Fig. 3a–c and Additional file 2: Figs. S22-S25).

Specifically, module I is highly activated in the six DCIS areas identified in the original study, representing the spatial expression of breast cancer markers such as SPINT2, FXYD3, and ERBB2 [35]. Module II (INV) is associated with high expression of FN1, a marker gene of epithelial-mesenchymal transition (EMT) [22], which is closely related to tumorigenesis, invasion and metastasis, and predicts poor outcome of breast cancer[81–83]. Overexpression of another member gene SERPINA3 is also reported to promote tumor invasion and EMT in breast cancer[84]. Module III represents CAFs expressing COL1A1, COL1A2, and COL3A1 [35] enriched in the cancer regions, whereas module IV identifies a subset known as myofibroblasts (myCAFs) (COL12A1, THBS2) that mainly contribute to extracellular matrix remodeling and growth and metastasis of cancer cells [85, 86]. Distinct signals of the immune landscape are detected, including B cells (V; IGLL5, JCHAIN), myeloid cells (VI; CD74, CST3, APOC1), and spatial expression of B2M (VII), an essential component of MHC class I antigen presentation [87]. The loss of B2M has been linked to immune escape and resistance of immune checkpoint blockade therapies, making it a potential biomarker of immunotherapy[88–90]. Compared to the results of other methods (Fig. 3d, e and Additional file 2: Fig. S26a, b), STModule better detects the cancer and CAF domains as well as associated genes (Fig. 3f–h and Additional file 2: Fig. 26c), especially the cancer region with ARI 0.57 compared to ARI $\le$ 0.46 of other methods, producing more accurate spatial maps of histological and pathological characteristics of the tissue.

As the tissue modules reveal recurrent communities that exert specific biological functions, they are expected to generalize to other regions of the same tissue. Therefore, we further investigated whether these modules could decode the spatial components of corresponding biological signals in other sections of the biopsy. We inferred spatial maps of the modules for layers 1, 3, and 4, respectively (Methods). The spatial maps align well with respective histological and pathological structures as well as spatial expression of associated genes, despite variations of the sections in structure organization and rotations due to changes of the biopsy in 3D space and arbitrary placement on the barcoded array in SRT profiling (Fig. 3i and Additional file 2: Figs. S27-S32). The results indicate that the modules, although initially identified in layer 2, characterize general biological processes across the tissue and act as crucial components in other areas as well.

### STModule identifies diverse biological signals from other cancers

**Fig. 4:**
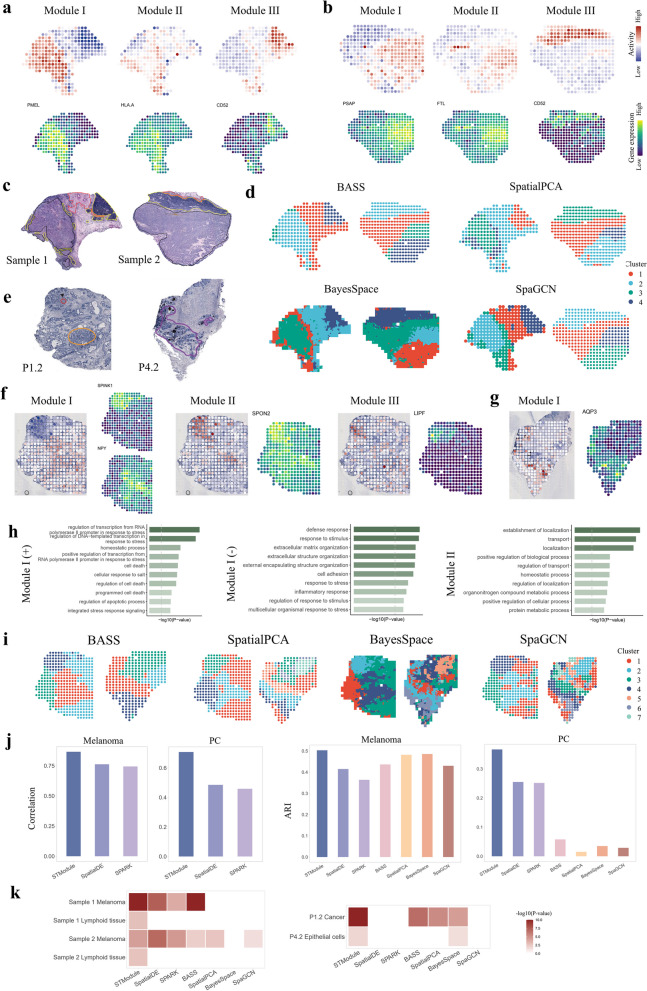
Application of STModule to other cancers.** a,b** Spatial maps of tissue modules identified by STModule from sample 1 (**a**) and sample 2 (**b**) of a melanoma dataset, including melanoma (I), transition area (II), and lymphoid tissue (III), along with spatial expression of representative associated genes. Colors indicate module activities or gene expression levels at different spots. **c** Histological annotations of the melanoma samples in the original study [51], including areas of melanoma (black), lymphoid (yellow), and stroma (red). **d** Results of domain clustering methods. **e** Histological annotations of samples P1.2 and P4.2 of prostate cancer in the original study [5] demonstrating regions of cancer Gs 3 + 3 (red), prostatic intraepithelial neoplasia (PIN) (orange), and chronic inflammation in stroma (purple). **f** Spatial maps of tissue modules identified by STModule from prostate sample P1.2 along with spatial expression of representative associated genes, including cancer (I −), PIN (I +), shared features of cancer and PIN (II), and center of cancer (III). **g** Spatial map of module I identified by STModule from prostate sample P4.2 representing inflammation along with spatial expression of associated gene AQP3. **h** The most highly enriched Gene Ontology terms of modules I and II in **f**. Color indicates the value of − log_10_(*P*-value). Dashed grey lines represent the threshold of *P*-value = 0.05. **i** Results of domain clustering methods. Colors indicate different clusters. **j** Comparison of the methods in terms of correlation and ARI of identifying major domains. **k** GSEA of associated genes detected by the methods. White color indicates adjusted *P*-value $\ge$ 0.05

As biological signals in tumors are intricate and heterogeneous, we applied STModule to another two datasets of human melanoma [51] and prostate cancer [5] profiled by ST to demonstrate tissue modules captured from various tissue types and cancer subtypes. In the melanoma dataset, STModule identifies similar components from the two samples, including cancer cells (I), transition areas (II), lymphoid tissue (III; CD74, CD52, MS4A1), immune activities (IV-VI), CAFs, and melanocytes (GAPDH) (Fig. 4a–c and Additional file 2: Figs. S33-S39). The cancer cell modules (I) of the two samples are characterized by distinct marker genes, i.e., PMEL, ATP1A1, and SPP1 in sample 1 whereas S100B and PSAP in sample 2, in line with previous studies [6, 51, 91]. The transition areas (II) that encompass both cancer cells and immune cells as defined in the original study [51] exhibit high expression of FTL, CTSB, and HLA-associated genes (e.g., HLA-A, HLA-B, HLA-C), indicating immune infiltration in the TME.

We included two samples from the prostate cancer dataset for their distinct pathological features, i.e., cancer (Gs 3 + 3) and prostatic intraepithelial neoplasia (PIN) in sample P1.2 and chronic inflammation in sample P4.2 (Fig. 4e). PIN is characterized by neoplastic growth of epithelial cells within pre-existing benign prostatic acini or ducts, and high-grade PIN is considered as the putative precursor of prostatic carcinoma [92, 93]. STModule identifies three tissue modules related to cancer cells from sample P1.2, among which module I discriminates between cancer cells (SPINK1, FMOD, AGR2) and PIN (NPY, ACPP, DBI), module II reveals shared features of cancer and PIN (SPON2, TFF3), and module III aligns with the center of the cancer region as annotated in the original study [5] (Fig. 4f, h). The modules potentially imply different pathological signals in prostate cancer progression, including early tumorigenesis of PIN expressing high levels of NPY [94], proliferation, metastasis, and invasion of tumor with overexpression of SPON2 and TFF3 in PIN and cancer cells compared to normal glands [95, 96], and unique alterations in cancer cells such as elevated expression of SPINK1 [97]. The center of the cancer (III) demonstrates high expression of LIPF, which can generate free fatty acids for cancer cells to uptake [98]. For sample P4.2, STModule identifies signal of inflammation (I; AQP3) and spatial expression of ATF3 (II), which plays an important role in regulating immune responses and exhibits dual functions as an oncogene or tumor suppressor [99] (Fig. 4g and Additional file 2: Fig. S40). Other modules are illustrated in Additional file 2: Figs. S40-S46 and discussed in Additional file 1: Supplementary notes.

STModule detects diverse tissue modules from melanoma and prostate cancer, capturing both tissue-/cancer-specific signals and fundamental components of tumors. It uncovers a greater variety of spatial structures with biological significance in comparison to other methods (Fig. 4d, i, Additional file 2: Fig. S47a, b and Fig. S48a, b), especially intricate signals associated with cancer progression, distinct activities of the immune landscape and convoluted structures of epithelial cells. In these two datasets, STModule also outperforms other methods in revealing the corresponding components and genes (Fig. 3j, k, Additional file 2: Fig. S47c-e and Fig. S48c, d).

### STModule dissects spatial structure organization of brain tissues

**Fig. 5:**
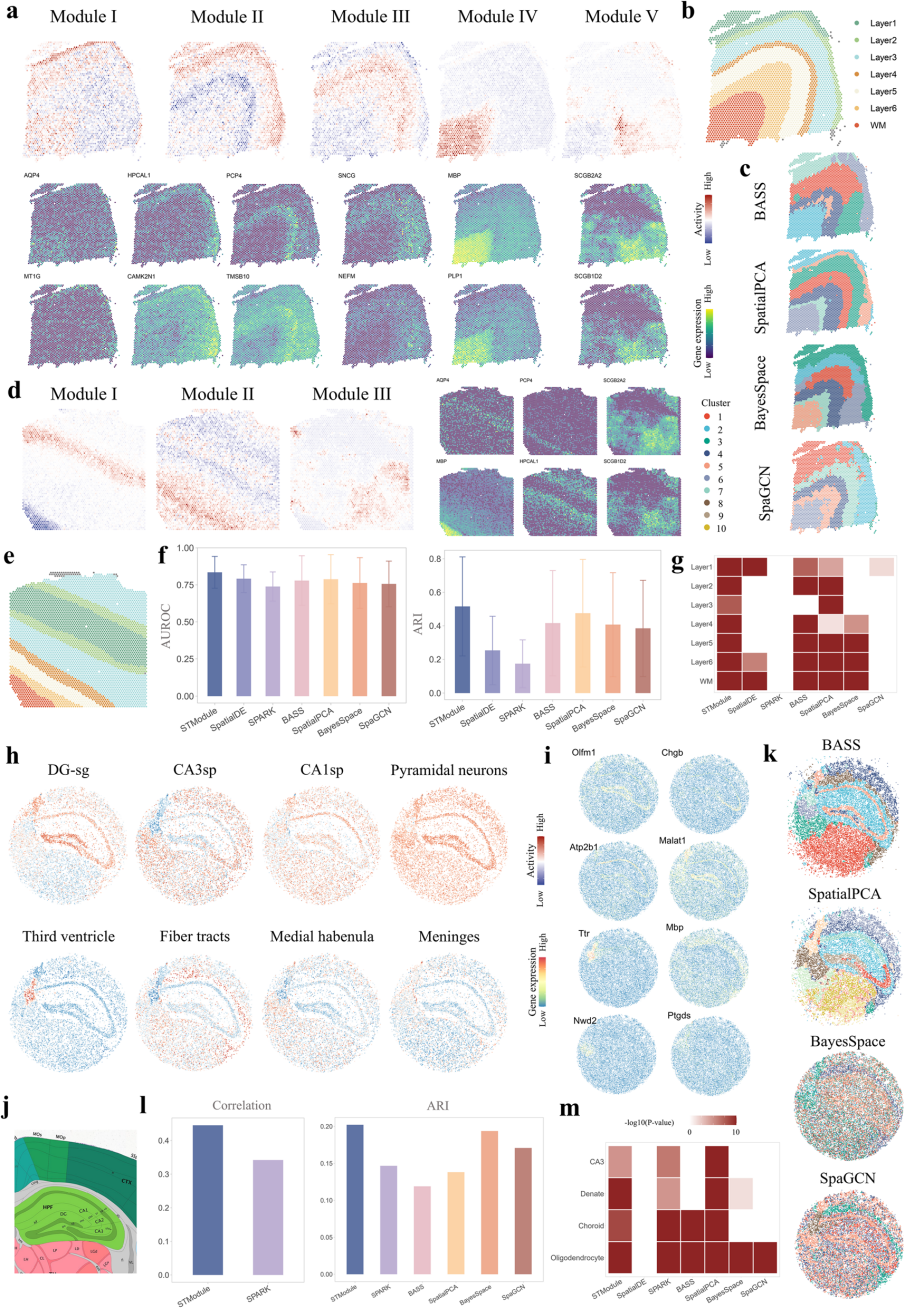
Results of the human dorsolateral prefrontal cortex (DLPFC) (a-g) and mouse hippocampus (h-m) datasets.** a** Spatial maps of tissue modules identified by STModule from DLPFC sample 151676 along with spatial expression of representative associated genes. **b** Illustration of cortical layers (1 to 6) and white matter (WM) of DLPFC sample 151676 annotated by the original study [56]. **c** Results of domain clustering methods. Colors indicate different clusters. **d** Spatial maps of tissue modules identified by STModule from DLPFC sample 151510 (left) and spatial expression of representative associated genes (right). **e** Illustration of spatial structure of DLPFC sample 151510 annotated by the original study. **f** Comparison of the methods in layer identification for all twelve samples in the DLPFC dataset, evaluated by AUROC (left) and ARI (right). **g** GSEA of associated genes detected by the methods for DLPFC sample 151676. White color indicates adjusted *P*-value $\ge$ 0.05. **h** Spatial maps of tissue modules identified by STModule from the mouse hippocampus dataset profiled by Slide-seqV2. DG-sg, granule cell layer of dentate gyrus. CA3sp, pyramidal layer of cornu ammonis 3. CA1sp, pyramidal layer of cornu ammonis 1. **i** Spatial expression of representative associated genes of the tissue modules in **h**. **j** Annotation of mouse hippocampus structure from Allen Brain Atlas. **k** Results of domain clustering methods for the hippocampus sample. Colors indicate different clusters. **l** Comparison of the methods in terms of correlation and ARI of identifying major components of the hippocampus sample. **m** GSEA of associated genes detected by the methods for the hippocampus sample. White color indicates adjusted *P*-value $\ge$ 0.05 or not applicable

The brain has a unique and complex spatial structure that corresponds to its functions. We applied STModule to a human DLPFC dataset (10 × Visium) [56] and a mouse hippocampus dataset (Slide-seqV2) [15] respectively to demonstrate tissue modules identified from different components of the brain. The human DLPFC dataset comprises 12 tissue sections collected from three donors along with manually annotated structures including six cortical layers and a white matter (WM) region (Additional file 2: Fig. S49). STModule captures structural layers of DLPFC samples through tissue modules induced by differential spatial expression patterns of gene sets among the layers. For instance, tissue modules identified from section 151676 reveal structures of layer 1 (I; AQP4, MT1G), layer 2 (II + ; HPCAL1, CAMK2N1), layer 4 (III + ; SNCG, NEFM), layer 5 (II − ; PCP4, TMSB10), WM (IV + ; MBP, PLP1), and neurons (IV − ; SNAP25), as well as spatial expression of the astrocytic marker GFAP (III −) (Fig. 5a, b, Additional file 2: Fig. S50 and Fig. S51). Layer 3 and layer 6 are not explicitly represented by the tissue modules, likely because they are not well depicted by maker genes including those identified in previous studies [56, 100] (Additional file 2: Fig. S52). Nevertheless, the gaps between the modules of other layers may offer insights into the overall architecture of the tissue section and assist experts in structural dissection. Additionally, STModule recognizes another module signifying the spatial expression of a gene set comprising SCGB2A2, SCGB1D2, MUC1, TFF1, and TFF3 (Fig. 5a, module V). While SCGB2A2 and SCGB1D2 are reported as markers of breast cancer [101], their roles in human brain have not been exhaustively investigated.

For a representative sample 151510 collected from another individual showing a different layout, STModule also identifies tissue modules indicating layers 1 (I +), 2 (II −), 5 (II +), WM (I −), and neurons (IV +) characterized by gene sets concordant with sample 151676, as well as spatial expression of SCGB2A2 (III) and GFAP (V) (Fig. 5d, e and Additional file 2: Fig. S53). Genes associated with layer 4 in sample 151676, such as NEFH, NEFL, and NEFM (Additional file 2: Fig. S51), do not distinguish the layer in sample 151510 (Additional file 2: Fig. S54), leading to the absence of the module in this sample. However, it is successfully recovered by applying the tissue modules of sample 151676 to sample 151510 as the spatial maps are summarized from the associated genes, as well as the other tissue modules (Additional file 2: Fig. S55). Individual markers may not act as reliable indicators of layers. In contrast, tissue modules based on common expression patterns of gene sets are less susceptible to variability and heterogeneity across individuals. As another example, AQP4 is a representative gene associated with layer 1 (Fig. 5a, d) and reported as a marker gene in the original study [56], but not able to differentiate layer 1 in all samples (Additional file 2: Fig. S56).

Certain clusters identified by spatial domain clustering methods appear to be tangled together rather than neatly arranged layer by layer, and some layers are not recognized, especially thin layers such as 1, 2, and 4 (Fig. 5c and Additional file 2: Fig. S49). One possible reason is that some layers are differentiated by small gene sets whose expression patterns are not discerned with current clustering granularity in the methods. Another reason is that the clustering process is likely confused by spatial expression of genes that are not layer-specific markers, e.g., SCGB2A2 and SCGB1D2. SpatialDE and SPARK only identify structures of layer 1 and WM, probably hindered by the detected SVGs (Additional file 2: Fig. S57). We quantitatively evaluated and compared the performance of the methods in identifying each layer for all twelve samples in the dataset (Methods). STModule is superior to the other methods with higher ARI and AUROC in layer identification (Fig. 5f) while detecting associated genes enriched of layer-specific genes in most cases (Fig. 5g and Additional file 2: Fig. S58).

Mouse hippocampus exhibits an “arrow-like” structure instead of stacked layers in DLPFC (Fig. 5j). STModule detects components of hippocampus including granule cell layer of dentate gyrus (DG-sg) (Olfm1), pyramidal layers of cornu ammonis 1 (CA1sp) (Chgb) and 3 (CA3sp) (Atp2b1), pyramidal neurons (Malat1), third ventricle (V3) (Ttr), fiber tracts (Mbp), medial habenula (MH) (Nwd2), and meninges (Ptgds) (Fig. 5h, i, Additional file 2: Fig. S59 and Fig. S60). STModule and SpatialPCA capture more components than other methods, dissecting the basic structure of mouse hippocampus (Fig. 5k and Additional file 2: Fig. S61a). STModule not only recognizes CA1sp, CA3sp, and DG-sg as distinct substructures by their respective differential genes but also characterizes them as pyramidal neurons by highly expressed genes in common. Moreover, STModule detects meninges which cover and protect the brain and spinal cord and are not distinguished by other methods. By comparing the results of the methods to the structures estimated by CARD, STModule better identifies the structural components than other methods and links them with their associated genes (Fig. 5l, m and Additional file 2: Fig. S61b, c). The results of human DLPFC and mouse hippocampus indicate that STModule is able to reveal the spatial organization of tissues with intricate structures and uncover components with various granularities.

### STModule recognizes the same structures from MOB datasets profiled by different technologies with various spatial resolutions

**Fig. 6:**
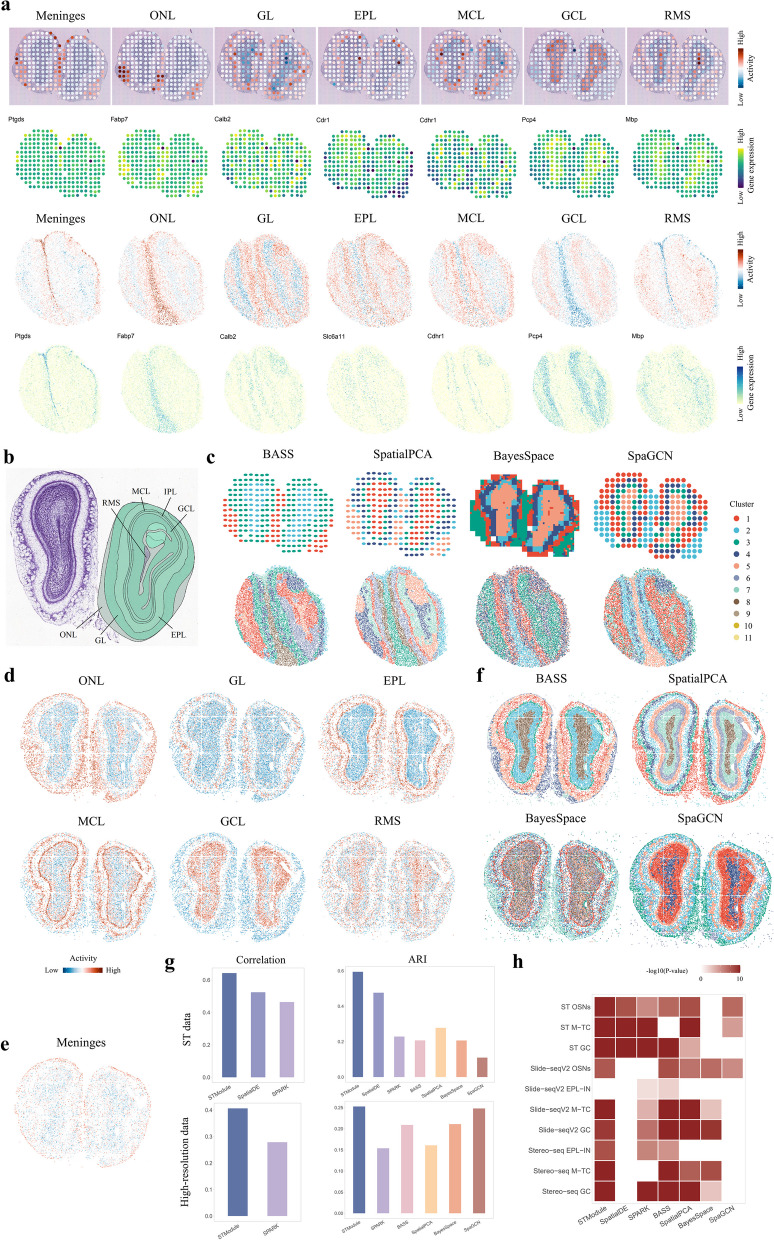
Results of the mouse olfactory bulb (MOB) datasets profiled by different technologies with various resolutions.** a** Spatial maps of tissue modules identified by STModule from MOB datasets profiled by ST (top) and Slide-seqV2 (bottom), along with spatial expression of representative associated genes. **b** Annotation of MOB structure from Allen Brain Atlas. ONL, olfactory nerve layer. GL, glomerular layer. EPL, external plexiform layer. MCL, mitral cell layer. IPL, internal plexiform layer. GCL, granule cell layer. RMS, rostral migratory stream. **c** Results of domain clustering methods for the MOB datasets profiled by ST (top) and Slide-seqV2 (bottom). **d** Spatial maps of tissue modules identified by STModule from MOB profiled by Stereo-seq. **e** Meninges of Stereo-seq MOB uncovered by the corresponding tissue module of ST MOB. **f** Results of domain clustering methods for MOB profiled by Stereo-seq. **g** Comparison of the methods in terms of correlation and ARI of identifying major components of the MOB samples profiled by ST (top), Slide-seqV2 and Stereo-seq (bottom). **h** GSEA of associated genes detected by the methods. White color indicates adjusted *P*-value $\ge$ 0.05 or not applicable

We further examined tissue modules detected by STModule from MOB profiled by different technologies with various spatial resolutions. We applied STModule to two MOB datasets profiled by ST [13] and Slide-seqV2 [15] with spot diameters of 100 and 10 µm, respectively. STModule successfully identifies MOB layers along with consistent associated genes, including olfactory nerve layer (ONL) (Fabp7), glomerular layer (GL) (Calb2), external plexiform layer (EPL) (Cdr1, Slc6a11), mitral cell layer (MCL) (Cdhr1), granule cell layer (GCL) (Pcp4), and rostral migratory stream (RMS) (Mbp), in line with markers reported in previous studies [102] (Fig. 6a, b and Additional file 2: Figs. S62-S65). SpatialDE and SPARK also detect the layers of MOB for the ST data, and SPARK detects ONL, MCL, GCL, and RMS for the Slide-seqV2 data (Additional file 2: Fig. S66). The spatial domain clustering methods outline the basic structure of MOB in high-resolution Slide-seqV2 data but fail to recover the same structure in the ST data of lower resolution (Fig. 6c), potentially due to the aggregated gene expression and convoluted signals. Furthermore, STModule detects meninges (Ptgds) of MOB in both datasets in accordance with mouse hippocampus, which are not captured by other methods (Fig. 6a, c). STModule also reveals neurons located in GL and GCL and discovers a substructure depicting the interface between ONL and GL characterized by Ptn, Npy, and Kctd12 (Additional file 2: Fig. S63 and Fig. S65).

Next, we applied STModule to another MOB dataset profiled by Stereo-seq [18]. Considering that the ultra-high resolution of Stereo-seq might lead to more sparse and noisy data and some critical genes are likely to be excluded from the top HVGs, the top 1,000 HVGs of the Stereo-seq data and all associated genes (2,695) of the modules identified from the ST and Slide-seqV2 MOB data were included in the analysis. STModule detects clear spatial structures of ONL, GL, EPL, MCL, GCL, and RMS as well as concordant associated genes with the other two MOB datasets (Fig. 6d, Additional file 2: Fig. S67 and Fig. S68). The meninges are not recognized by the tissue modules, possibly as a result of the noisy expression of the associated genes (Additional file 2: Fig. S69). We then applied the tissue modules of ST and Slide-seqV2 MOB to the Stereo-seq data respectively to investigate whether they generalize across SRT data of distinct technologies. The modules effectively transfer to the Stereo-seq data and additionally uncover the spatial structures of meninges, neurons, and interface between ONL and GL (Fig. 6e and Additional file 2: Fig. S70). The comparison methods also dissect the layers of the MOB sample (Fig. 6f and Additional file 2: Fig. S71).

According to the results of single-cell clustering and layer-enriched clusters of a MOB study [65], we estimated the spatial domains for the three datasets with CARD (Additional file 2: Fig. S72a-c). To reduce effects of noise, only domains with the highest proportion $\ge$ 0.5 were included. Single-cell clusters of OSNs, EPL-IN, M-TC, and GC indicate the layers of ONL, EPL, MCL, and GCL, respectively. Based on the domains, STModule achieves the best performance in both ST and high-resolution (i.e., Slide-seqV2 and Stereo-seq) data (Fig. 6g and Additional file 2: Fig. S72d). The associated genes of the modules are also enriched of corresponding cell type markers except the EPL of the Slide-seqV2 data (Fig. 6h).

## Discussion

Spatially resolved transcriptomics enables profiling of gene expression with spatial information for intact tissue, which facilitates investigating spatial organization of transcriptomic landscapes of tissues. Here, we have presented a computational method STModule which identifies tissue modules from SRT data. Applied to SRT data of various cancer subtypes including human PDAC, breast cancer, prostate cancer, and melanoma, STModule identifies tissue modules revealing biological signals of cancer, PIN, immune activities and immune infiltration in TME, molecular features related to clinical outcomes, distinct cell types/subtypes, etc. It also detects tissue modules from brain sections that dissect spatial architectures of human DLPFC (10 × Visium), mouse hippocampus (Slide-seqV2), and MOB (ST, Slide-seqV2 and Stereo-seq). Moreover, STModule detects some spatial expression patterns of gene groups without comprehensive investigations in previous studies, which may imply important molecular signals in the biological contexts. The results demonstrate that STModule is able to uncover spatial components of transcriptomic landscapes which facilitates capturing functional and structural components as well as other essential molecular features of tissues for downstream analysis. The tissue modules provide insights into pathological and histological characteristics of tissues, disease mechanisms, and treatment development [73, 103].

STModule differs from existing computational methods for SRT analysis. SVG detection methods aim to select informative genes in the spatial context for downstream analysis. While they can recognize representative spatial expression patterns by clustering the SVGs, the patterns substantially rely on the results of SVG detection, revealing limited expression components in the biological context as demonstrated in our results. In addition, the methods examine each gene independently without accounting for the expression correlations between them, rendering them inappropriate to study co-expression modules. Spatial domain clustering methods group the profiled spots/cells into domains with high coherence of gene expression and spatial distribution, primarily investigating the similarities between the spots/cells in the spatial transcriptomic landscape. They are promising in generating rough segmentations of tissues for further annotations, rather than exploring spatial components of gene expression and uncovering biological signals in the tissue context. They encounter challenges in dealing with convoluted biological processes and structures, e.g., the co-occurrence of cancer cells and CAFs in the PDAC samples and the architecture of MOB profiled by ST. Cell type deconvolution methods are used to estimate the spatial distributions of interested cell types/subtypes based on single-cell references, focusing on spatial communications and interactions among distinct cell types/subtypes. In contrast, STModule explores tissue sections from the perspective of tissue modules, investigating inherent biological signals revealed by underlying spatial patterns.

STModule identifies underlying expression components as tissue modules that collectively comprise the overall expression of a tissue section, capable of disentangling convoluted signals in SRT with lower resolution (e.g., ST) and dissecting spatial structures from data with cellular/subcellular resolution (e.g., Slide-seqV2 and Stereo-seq). It infers the associated genes and spatial maps of tissue modules jointly and simultaneously, addressing the issue of impaired performance resulting from individually optimizing the two tasks. Induced by spatial expression patterns of the transcriptomic landscape, the tissue modules reflect both fundamental functional components such as CAFs, ductal cells and immune cells, and tissue-specific biological signals such as spatial pattern of MT-RNR2-like genes in the PDAC sample, expression of interferon-induced genes in the melanoma sample and various biological processes related to tumor progression in prostate cancer. STModule additionally uncovers some spatial structures and molecular features not revealed by other methods, such as the spatial expression of SCGB2A2 and SCGB1D2 commonly found in the DLPFC samples, meninges of mouse hippocampus and olfactory bulb, interface between ONL and GL in MOB. The granularities of tissue modules are flexible and automatically determined, ranging from higher-order spatial structures to finer components, thereby uncovering biological processes of multiple scales, including immune signal of B2M and different subtypes of immune cells, the overall layout and substructures of pancreatic/prostatic glands, as well as neurons and distinct components in MOB and hippocampus. Furthermore, the spatial maps of tissue modules are estimated based on the whole sets of associated genes which characterize the components more reliably compared to individual markers, e.g., AQP4 of layer 1 in DLPFC. The modules also generalize to other sections, not only those collected from the same biopsy, but also sections from other individuals or data profiled by a distinct technology, suggesting that they capture general functional components of the tissues and align with the biological processes in unseen data.

Beyond the datasets and simulated cases in the study, STModule is also applicable to data with more complex spatial patterns such as mouse colon with curved shapes [104] (Additional file 2: Fig. S73). It effectively dissects the structures of colon Swiss roll in both steady (d0) and healing (d14) states. As sequencing-based technologies are more unbiased, we focused on datasets profiled by this branch of technologies in the study. However, STModule is also compatible with SRT data profiled by imaging-based technologies. Applied to a MERFISH dataset of mouse primary motor cortex [105], STModule successfully identified different layers composed of distinct cell types (Additional file 2: Fig. S74). Moreover, we utilized SE kernel in the method to model spatial covariances among the spots/cells. We additionally investigated the performance of periodic kernels using the first set of simulations with different scenarios (Additional file 1: Supplementary notes). The performance of spatial pattern identification with periodic kernels is slightly worse than the SE kernel, while that of associated gene detection with periodic kernel is significantly poorer (Additional file 2: Fig. S75). One possible reason is that the fixed values of parameter p in the periodic kernel hinder the optimization process of the algorithm. In addition, we summarized the computational time and maximum memory usage of the methods in Additional file 3: Table S3.

There are also some limitations of the method. Since STModule detects tissue modules based on spatial expression patterns, it can only recognize spatial components highlighted by differentially expressed genes. Although captured in other biopsies, a component might still remain hidden in a particular tissue section if the associated genes exhibit relatively random expression rather than the respective spatial structure, e.g., layer 4 identified in sample 151676 but missed in sample 151510 in the DLPFC dataset. However, it could be tackled by leveraging tissue modules identified from other sections to recover the component for this one as the algorithm would be directed to infer the shared expression pattern of the associated genes. Moreover, while the tissue modules can be generalized across tissue sections, individuals, and profiling technologies, it might be more robust to identify tissue modules from multiple sections of a biopsy collectively, as discussed in previous studies that analyze tissue architecture or characteristics across multiple slices[106–108], which poses a potential direction for enhancing STModule in the future.

## Conclusions

In this study, we have developed STModule, a Bayesian method to identify tissue modules from SRT data to uncover spatial components and characteristics that underlie the intricate tissue contexts. It is able to detect crucial molecular properties of tissues for downstream analysis, capturing a broader spectrum of biological signals compared to other methods. The tissue modules reveal spatial organization and interactions of essential components in the transcriptomic landscapes and provide insights into histological architecture, tumor microenvironment, disease mechanisms, and treatment development.

## Supplementary Information

Additional file 1. Supplementary notes, including details of STModule and simulations, additional discussion of modules for melanoma and prostate cancer, and investigation of periodic kernels.

Additional file 2. Supplementary figures S1-S75.

Additional file 3. Supplementary tables S1-S3. Table S1. Summary of simulations in the study. Table S2: Summary of datasets used in the study. Table S3: Comparison of computational time and maximum memory usage.

## Data Availability

The datasets of human BC, PC, melanoma and MOB profiled by ST are available at Spatial Research (https://www.spatialresearch.org/resources-published-datasets/). The PDAC dataset is available at GEO with accession number GSE111672. The human DLPFC dataset is accessible using the spatialLIBD package (http://research.libd.org/spatialLIBD/). The Slide-seqV2 datasets of mouse hippocampus and MOB are available at Single Cell Portal (https://singlecell.broadinstitute.org/single_cell/study/SCP815/highly-sensitive-spatial-transcriptomics-at-near-cellular-resolution-with-slide-seqv2#study-summary). The Stereo-seq MOB dataset is available at GitHub, https://github.com/JinmiaoChenLab/SEDR_analyses. The single-cell references used in this study during cell-type deconvolution for PDAC, BC, PC, melanoma and MOB analysis are available at GEO with accession number GSE111672, GSE176078, GSE181294, GSE115978 and GSE121891, respectively. The single-cell reference for mouse hippocampus is available at Google Drive, https://drive.google.com/drive/folders/1uXIPb-ifkiy-3sG5cuMtees8H_wnMqbT.

## Abbreviations

SRT: Spatially resolved transcriptomics
TME: Tumor microenvironment
NGS: Next-generation sequencing
ST: Spatial Transcriptomics
SVG: Spatially variable gene
NSVG: Non-spatially variable gene
PDAC: Pancreatic ductal adenocarcinoma
BC: Breast cancer
PC: Prostate cancer
DLPFC: Dorsolateral prefrontal cortex
MOB: Mouse olfactory bulb
SE: Squared exponential
VB: Variational Bayes
KL: Kullback–Leibler
PIP: Posterior inclusion probabilities
H&E: Hematoxylin and eosin
GEO: Gene Expression Omnibus
HVG: Highly variable gene
PCA: Principal component analysis
AUROC: Area under the receiver operating characteristic
AUPR: Area under the precision-recall
ARI: Adjusted rand index
DEG: Differentially expressed gene
GSEA: Gene set enrichment analysis
GMT: Gene Matrix Transposed
GO: Gene Oncology
BP: Biological process
MF: Molecular function
CC: Cellular component
KEGG: Kyoto Encyclopedia of Genes and Genomes
CAF: Cancer-associated fibroblast
apCAF: Antigen-presenting CAF
myCAF: Myofibroblast
ADM: Acinar-to-ductal metaplasia
PanIN: Pancreatic intraepithelial neoplasia
DCIS: Ductal carcinoma in situ
INV: Invasive ductal cancer
EMT: Epithelial-mesenchymal transition
PIN: Prostatic intraepithelial neoplasia
WM: White matter
DG-sg: Granule cell layer of dentate gyrus
CA1sp: Pyramidal layers of cornu ammonis 1
CA3sp: Pyramidal layers of cornu ammonis 3
V3: Third ventricle
MH: Medial habenula
ONL: Olfactory nerve layer
GL: Glomerular layer
EPL: External plexiform layer
MCL: Mitral cell layer
GCL: Granule cell layer
RMS: Rostral migratory stream
