# Supplementary material for: STModule: identifying tissue modules to uncover spatial components and characteristics of transcriptomic landscapes
Source: Genome Med. 2025 Mar 3;17:18. doi: 10.1186/s13073-025-01441-9 (PMC11874447; doi:10.1186/s13073-025-01441-9)
Supplement: Supplementary file 1 — Additional file 1. Supplementary notes, including details of STModule and simulations, additional discussion of modules for melanoma and prostate cancer, and investigation of periodic kernels. [file 13073_2025_1441_MOESM1_ESM.pdf]

Additional file 1: Supplementary notes

Details of STModule

The Bayesian model of STModule

The full model of STModule is as follows:

$$P(Y|\theta) = \prod_{sl} \mathcal{N}\left(Y_{sl} | \sum_c P_{sc} G_{cl}, {\lambda_s}^{-1}\right)$$
$$P(\lambda_s) = Gamma(u, v)$$
$$P\left(P_{.c} | \sigma_c, \Sigma^{(c)}\right) = \mathrm{MVN}\left(P_{.c} | 0, {\sigma_c}^{-1} \Sigma^{(c)}\right)$$
$$P(\sigma_c) = Gamma(m, n)$$
$$P(r_c) = Gamma(a, b)$$
$$l_c^2 = \frac{1}{r_c}$$
$$\Sigma_{s,s'}^{(c)} = exp\left(-\frac{d(s, s')^2}{2l_c^2}\right) = exp\left(-\frac{1}{2} d(s, s')^2 r_c\right)$$
$$G_{cl} = \omega_{cl} s_{cl}$$
$$P(\omega_{cl} | \beta_c) = \mathcal{N}\left(\omega_{cl} | 0, {\beta_c}^{-1}\right)$$
$$P(\beta_c) = Gamma(\beta_c | e, f\ )$$
$$P(s_{cl} | \psi_{cl}, \phi_{cl}) = Bernoulli(s_{cl} | \psi_{cl} \phi_{cl})$$
$$P(\psi_{cl}) = Beta(\psi_{cl} | g, h\ )$$
$$P(\phi_{cl} | \rho_c) = Bernoulli(\phi_{cl} | \rho_c)$$
$$P(\rho_c) = Beta(\rho_c | t, z\ )$$

The model is fitted using Variational Bayes (VB). The distribution of $Q(\theta)$ is in the form of

$$Q(\theta) = \prod_{s,c} Q(P_{sc}) \prod_c Q(\sigma_c) \prod_c \delta_{r_c^*}(r_c) \prod_{c,l} Q(\omega_{cl} | s_{cl}) Q(s_{cl}) \prod_c Q(\beta_c)$$
$$\prod_{c,l} \delta_{\psi_{cl}^*}(\psi_{cl}) \prod_{c,l} \delta_{\phi_{cl}^*}(\phi_{cl}) \prod_c \delta_{\rho_c^*}(\rho_c) \prod_s Q(\lambda_s)$$

where $\delta(\cdot)$ is the Dirac delta function.

Updates of the variables

- Factor matrix P

$$Q(P_{.c}) = MVN\left(P_{.c} \middle| \mu_{.c}^*, {\Omega^{(c)}}^{*-1}\right)$$
$${\Omega^{(c)}}^* = \langle \sigma_c \rangle \langle {\Sigma^{(c)}}^{-1} \rangle + \langle \lambda \rangle \sum_l \langle {s_{cl}}^2 {w_{cl}}^2 \rangle I_S$$
$$\mu_{.c}^* = \left(\sum_l Y_{.l} \langle \lambda \rangle \langle s_{cl} w_{cl} \rangle - \sum_l \langle \lambda \rangle \langle s_{cl} w_{cl} \rangle \sum_{k \neq c} \langle P_{.k} \rangle \langle s_{kl} w_{kl} \rangle\right) {\Omega^{(c)}}^{*-1}$$

- Scaling factor σ

$$Q(\sigma_c) = Gamma(\sigma_c | m_c^*, n_c^*)$$
$$m_c^* = m + \frac{1}{2} S$$

$$n_c^* = \left(\frac{1}{n} + \frac{1}{2}\sum\nolimits_s \langle P_{.c}^T \rangle \langle \Sigma^{(c)^{-1}} \rangle \langle P_{.c} \rangle \right)^{-1}$$

- Length scale r

Since the prior of length scale is not conjugate, r is updated by maximizing the relevant evidence lower bound (ELBO) using Newton's optimization method:

$$f(r_c) = \log P(P_{.c}|r_c) + \log P(r_c)$$

$$= -\frac{1}{2}\langle P_{.c}^T \rangle \langle \sigma_c \rangle \langle \Sigma^{(c)^{-1}} \rangle \langle P_{.c} \rangle + (a-1)\log r_c - \frac{1}{b} r_c$$

$$r_c^* = r_c - \frac{f'(r_c)}{f''(r_c)}$$

where

$$f'(r_c) = -\frac{1}{2}\langle P_{.c}^T \rangle \langle \sigma_c \rangle (K^{-1})' \langle P_{.c} \rangle + \frac{a-1}{r_c} - \frac{1}{b}$$

$$f''(r_c) = -\frac{1}{2}\langle P_{.c}^T \rangle \langle \sigma_c \rangle (K^{-1})'' \langle P_{.c} \rangle - \frac{a-1}{r_c^2}$$

$$K = \Sigma^{(c)} = exp\left(-\frac{1}{2} D r_c\right)$$

$$(K^{-1})' = -K^{-1} K' K^{-1}$$

$$(K^{-1})'' = 2K^{-1} K' K^{-1} K' K^{-1} - K^{-1} K'' K^{-1}$$

$$K' = -\frac{1}{2} D \cdot exp\left(-\frac{1}{2} D r_c\right)$$

$$K'' = \frac{1}{4} D^2 \cdot exp\left(-\frac{1}{2} D r_c\right)$$

D is the matrix of squared Euclidean distance of the spots/cells.

- Sparsity parameters of factor matrix G:

$$Q(w_{cl}|s_{cl}) = \mathcal{N}(w_{cl}|s_{cl} o_{cl}^*, (s_{cl}\eta_{cl}^* + (1-s_{cl})\langle \beta_c \rangle)^{-1})$$
$$\eta_{cl}^* = \langle \beta_c \rangle + \sum\nolimits_s \langle \lambda_s \rangle \langle {P_{sc}}^2 \rangle$$
$$o_{cl}^* = {\eta_{cl}^*}^{-1} \left(\sum\nolimits_s Y_{sl} \langle \lambda_s \rangle \langle P_{sc} \rangle - \sum\nolimits_s \langle \lambda_s \rangle \langle P_{sc} \rangle \sum\nolimits_{k \neq c} \langle P_{sk} \rangle \langle s_{kl} w_{kl} \rangle \right)$$

$$Q(s_{cl}) = Bernoulli(s_{cl}|\gamma_{cl}^*)$$
$$\gamma_{cl}^* = 1/\left(1 + e^{-\vartheta_{cl}^*}\right)$$
$$\vartheta_{cl}^* = log(\psi_{cl}^* \phi_{cl}^*) - \frac{1}{2} log(\eta_{cl}^*) + \frac{1}{2}\eta_{cl}^* {o_{cl}^*}^2 - log(1 - \psi_{cl}^* \phi_{cl}^*) + \frac{1}{2} log\langle \beta_c \rangle$$

$$Q(\beta_c) = Gamma(\beta_c|e_c^*, f_c^*)$$
$$e_c^* = e + \frac{1}{2} L$$
$$f_c^* = \left(\frac{1}{f} + \frac{1}{2}\sum\nolimits_l \langle {w_{cl}}^2 \rangle \right)^{-1}$$

ψ_{cl}, ϕ_{cl} and ρ_c are updated by point estimation[1].

- Noise term λ:

$$Q(\lambda_s) = Gamma(\lambda_s|u_s^*, v_s^*)$$
$$u_s^* = u + \frac{1}{2}L$$
$$v_s^* = \left(\frac{1}{v} + \frac{1}{2}\sum\nolimits_l \langle\left(Y_{sl} - \sum\nolimits_c \langle P_{sc}\rangle\langle s_{cl}w_{cl}\rangle\right)^2\rangle\right)^{-1}$$

ELBO

Minimizing the KL-divergence between $Q(\theta)$ and $P(\theta|Y)$ was equivalent to maximizing ELBO:

$$ELBO = E_Q[\log P(Y,\theta)] - E_Q[\log Q(\theta)]$$

$$= \frac{L}{2}\sum\nolimits_s \langle\log\lambda_s\rangle - \frac{1}{2}\sum\nolimits_{sl}\langle\lambda_s\rangle\langle\left(Y_{sl} - \sum\nolimits_c \langle P_{sc}\rangle\langle s_{cl}w_{cl}\rangle\right)^2\rangle$$

$$+\frac{S}{2}\sum\nolimits_c\langle\log\sigma_c\rangle - \frac{1}{2}\sum\nolimits_c\langle P_{.c}^T\rangle\langle\sigma_c\rangle\langle{\Sigma^{(c)}}^{-1}\rangle\langle P_{.c}\rangle - \frac{1}{2}\sum\nolimits_c \log\left(det\left({\Omega^{(c)}}^*\right)\right)$$

$$+\sum\nolimits_c\left[(m-1)\langle\log\sigma_c\rangle - \frac{1}{n}\langle\sigma_c\rangle\right]$$

$$-\sum\nolimits_c[(m_c^*-1)\langle\log\sigma_c\rangle - m_c^*(1+\log|n_c^*|) - \log\Gamma(m_c^*)]$$

$$+\sum\nolimits_c\left[(a-1)\log r_c^* - \frac{1}{b}\langle r_c^*\rangle\right]$$

$$+\sum\nolimits_s\left[(u-1)\langle\log\lambda_s\rangle - \frac{1}{v}\langle\lambda_s\rangle\right]$$

$$-\sum\nolimits_s[(u_s^*-1)\langle\log\lambda_s\rangle - u_s^*(1+\log|v_s^*|) - \log\Gamma(u_s^*)]$$

$$+\frac{1}{2}\sum\nolimits_c\langle\log\beta_c\rangle - \frac{1}{2}\sum\nolimits_{c,l}\langle\beta_c\rangle\langle {w_{cl}}^2\rangle$$

$$-\frac{1}{2}\sum\nolimits_{c,l}\gamma_{cl}^* log(\eta_{cl}^*) + \frac{1}{2}\sum\nolimits_{c,l}(1-\gamma_{cl}^*)\langle\log\beta_c\rangle$$

$$+\sum\nolimits_c(e-1)\langle\log\beta_c\rangle - \sum\nolimits_c\frac{1}{f}\langle\beta_c\rangle - \sum\nolimits_c[(e_c^*-1)\langle\log\beta_c\rangle - e_c^* - \log\Gamma(e_c^*)]$$

$$+\sum\nolimits_{c,l}[\gamma_{cl}^* log(\psi_{cl}^*\phi_{cl}^*) + (1-\gamma_{cl}^*)\log(1-\psi_{cl}^*\phi_{cl}^*) - \gamma_{cl}^*\log\gamma_{cl}^* - (1-\gamma_{cl}^*)\log(1-\gamma_{cl}^*)]$$

$$+\sum\nolimits_{c,l}[(g-1)\log\psi_{cl}^* + (h-1)\log(1-\psi_{cl}^*)]$$

$$+\sum\nolimits_{c,l}[\phi_{cl}^*\log\rho_c^* + (1-\phi_{cl}^*)\log(1-\rho_c^*)]$$

$$+\sum\nolimits_c[(t-1)\log\rho_c^* + (z-1)\log(1-\rho_c^*)]$$

Simulations

Simulated spatial patterns

In the first set of simulations, the spatial patterns were generated following the data generative schemes in[2-4] based on spot and linear patterns (Additional file 1: Fig. S1). In scenarios 1-5, a spot pattern was generated by randomly selecting a location on the lattice as its center and sampling a radius from 2 to 5. For a linear pattern, a slope and a location were randomly sampled to establish the central line of the pattern, while the line width was sampled from 4 to 10. In scenarios 6 and 7, spatial patterns were generated using spot patterns. The radius of higher-level patterns was sampled from 5 to 7, while the radius of sub-patterns was sampled from 2 to 3 and the centers were sampled in the area of the higher-level patterns. Within each spot/linear pattern, values were set to 10 at the center spot or central line and decreased linearly to zero to the boundary. The values of other locations on the lattice beyond the pattern were set to zero. The overall expression of genes were determined based on the simulated spatial patterns, baseline expression and random noise, that is, for each gene, its expression was calculated as $y = \sigma y_{spatial} + y_{base} + \varepsilon$, where $y_{spatial}$ represents the spatial pattern it follows, σ is a scaling factor randomly sampled from 1 to 5 to control the magnitude of its expression, y_{base} is the baseline expression set to 2 for all genes, and $\varepsilon \sim \mathcal{N}(0,1)$ represents the random noise. In addition to the spatially expressed genes (SEGs), we also simulated non-spatially expressed genes (NSEGs) for each expression profile. The spatial component of expression was set to a zero vector for the NSEGs, thereby, they only exhibited baseline expression with random noise. The final simulated profiles were generated combining the expression of SEGs and NSEGs.

In scenarios 1, 2 and 3, we simulated the spatial expression of a set of 100 genes sharing a similar expression pattern which were generated by 1, 2 and 3 basic patterns respectively to investigate spatial patterns of simple and complex shapes. In scenario 4, we simulated the spatial expression of two individual gene sets, each containing 100 genes with different spatial patterns, representing two independent biological signals. In scenario 5, we simulated the situation where two biological processes involved the same subset of genes and affected their spatial expression accordingly. We generated two gene sets with 50 overlapping genes, each comprising 100 co-expressing genes in total. The non-overlapping genes of each set (subsets 1 and 2) followed the respective basic pattern, while the overlapping genes expressed the merged pattern. In scenarios 6 and 7, we investigated multi-scale patterns to simulate biological concepts in different granularities. We first generated a spatial pattern for a gene set (GS1) as the higher-level concept and then generated one (scenario 6) or two (scenario 7) sub-patterns included in the previous pattern as the sub-concepts with their own associated genes (GS2 and GS3).

Simulated layer-wise DLPFC data

In the second set of simulations, we simulated layer-wise expression based on the layout of real DLPFC samples including seven layers following SRTsim[5]. For each layer, we simulated 10 expression profiles containing 100 SEGs and 900 NSEGs. Specifically, for each simulated profile, the SEGs are highly expressed in the spots of the corresponding layer, following a negative binomial distribution with the dispersion parameter set to 0.3, which is recommended by SRTsim. For the NSEGs, the mean expression was set to 0.03. For the SEGs, we simulated genes with both increased and decreased expression compared to NSEGs, by setting their mean expression to 10 times or 1/10 of that of NSEGs.

Additional discussion of tissue modules for the melanoma and prostate cancer datasets

Melanoma

Highly concordant with the results of breast cancer, immune signals of B cells (IV; IGLL5, IGJ (JCHAIN)), spatial expression of B2M (V) and macrophages (VI) are also detected in the melanoma

samples. In addition, STModule identifies another module (X) in sample 2 related to two interferon-induced genes, IFI6 and IFI27, that regulate apoptosis and immune responses[6] (Additional file 2: Fig. S34b). They play critical roles in oncogenic NRAS-induced melanocyte transformation and tumor growth and can serve as targets of ATF3 to suppress growth of melanoma as well as predictors of clinical outcome of immunotherapies[7-9].

Prostate cancer

STModule disentangles distinct subtypes of epithelial cells in both samples differentiated by respective markers, including luminal epithelial cells (KLK3, MSMB, ACPP), basal epithelial cells (CYR61, FOSB, KRT17), club cells (SCGB1A1, S100P, GDF15) and hillock cells (KRT13)[10-12], as well as other tissue components such as fibroblasts, B cells, smooth muscle and prostatic glands (Additional file 2: Figs. S40-S46).

Investigation of periodic kernels

We used SE kernel in STModule to model spatial covariances among the spots/cells. Here we further investigated the performance of using periodic kernels defined as follows:

$$\Sigma_{s,s'}^{(c)} = \sigma^2 exp\left(-\frac{2}{l_c^2} sin^2\left(\pi\frac{d(s,s')}{p}\right)\right) = \sigma^2 exp\left(-2sin^2\left(\pi\frac{d(s,s')}{p}\right) r_c\right),$$

where σ is amplitude, l_c is the length scale of module c, p is the period of the covariance. We embedded the periodic kernel into our algorithm by using 5 different values of p determined following [2] and replaced the squared Euclidean distance in the SE kernel with $4sin^2\left(\pi\frac{d(s,s')}{p}\right)$, $l_c^2 = 1/r_c$. As the SE kernel, r_c was modeled as a variable in the algorithm and estimated by Variational Bayes.

References

1. Hore V, Vinuela A, Buil A, et al. Tensor decomposition for multiple-tissue gene expression experiments. *Nat Genet*. 2016;48(9):1094–1100.
2. Sun S, Zhu J, Zhou X. Statistical analysis of spatial expression patterns for spatially resolved transcriptomic studies. *Nature methods*. 2020;17(2):193–200.
3. Li Q, Zhang M, Xie Y, Xiao G. Bayesian modeling of spatial molecular profiling data via gaussian process. *Bioinformatics*. 2021;37(22):4129–4136.
4. Edsgärd D, Johnsson P, Sandberg R. Identification of spatial expression trends in single-cell gene expression data. *Nature methods*. 2018;15(5):339–342.
5. Zhu J, Shang L, Zhou X. SRTsim: Spatial pattern preserving simulations for spatially resolved transcriptomics. *Genome Biol*. 2023;24(1):39.
6. Hao J, Sun M, Li D, Zhang T, Li J, Zhou D. An IFI6-based hydrogel promotes the healing of radiation-induced skin injury through regulation of the HSF1 activity. *Journal of Nanobiotechnology*. 2022;20(1):1–14.
7. Gupta R, Forloni M, Bisserier M, Dogra SK, Yang Q, Wajapeyee N. Interferon alpha-inducible protein 6 regulates NRASQ61K-induced melanomagenesis and growth. *Elife*. 2016;5:e16432.
8. Xu L, Zu T, Li T, et al. ATF3 downmodulates its new targets IFI6 and IFI27 to suppress the growth and migration of tongue squamous cell carcinoma cells. *PLoS Genetics*. 2021;17(2):e1009283.
9. Bustos MA, Mizuno S, Ryu S, Hoon DS. Transcriptomic signatures in melanoma lymph node metastasis that relate to immune oncology treatment. *Cancer Res*. 2023;83(7):6375.
10. Henry GH, Malewska A, Joseph DB, et al. A cellular anatomy of the normal adult human prostate and prostatic urethra. *Cell reports*. 2018;25(12):3530–3542.
11. Chen S, Zhu G, Yang Y, et al. Single-cell analysis reveals transcriptomic remodellings in distinct cell types that contribute to human prostate cancer progression. *Nat Cell Biol*. 2021;23(1):87–98.
12. Song H, Weinstein HNW, Allegakoen P, et al. Single-cell analysis of human primary prostate cancer reveals the heterogeneity of tumor-associated epithelial cell states. *Nature communications*. 2022;13(1):141.
