# Supplementary material for: STModule: identifying tissue modules to uncover spatial components and characteristics of transcriptomic landscapes
Source: Genome Med. 2025 Mar 3;17:18. doi: 10.1186/s13073-025-01441-9 (PMC11874447; doi:10.1186/s13073-025-01441-9)
Supplement: Supplementary file 3 — Additional file 3. Supplementary tables S1-S3. Table S1. Summary of simulations in the study. Table S2: Summary of datasets used in the study. Table S3: Comparison of computational time and maximum memory usage. [file 13073_2025_1441_MOESM3_ESM.pdf]

Additional file 3: Supplementary tables

Table S1: Summary of simulations in the study

	Data generation tool/scheme	Purpose	Number of scenarios	Number of simulations for each scenario
Set 1: simulated spatial patterns	Following [1, 46, 47]	Spatial pattern	7	100
Set 2: simulated layer-wise DLPFC data	Following SRTsim	Spatial pattern	7 (layers)	10
Set 3: simulated whole-tissue DLPFC data	SRTsim	Spatial pattern	2	10
Set 4: simulated mouse brain data	scDesign3	SVG	1	10

Table S2: Summary of datasets used in the study

Dataset	Technology	Number of slides/samples used	Number of genes	Number of spots/cells
PDAC	ST	2<br>(Samples A & B)	19,738	428 & 224
BC	ST	4<br>(Layers 1- 4)	14,789 ~ 14,929	251~264
Melanoma	ST	2<br>(Samples 1 & 2)	16,148 & 16,831	293 & 383
PC	ST	2<br>(P1.2 & P4.2)	17,678 & 17,781	406 & 324
DLPFC	10x Visium	12	33,538	3,460 ~ 4,789
Hippocampus	Slide-seqV2	1	19,653	20,143
MOB	ST	1	16,218	262
MOB	Slide-seqV2	1	21,220	21,724
MOB	Stereo-seq	1	27,106	19,527

Table S3: Comparison of computational time and maximum memory usage*

	ST data (MOB)	10x Visium data (DLPFC)	Slide-seqV2 data (MOB)	Stereo-seq data (MOB)
STModule	14min<br>140M	1.8h<br>770M	5h<br>27G	4h<br>32G
SpatialDE	14min<br>177M	34h<br>3G	-	-
SPARK/SPARK-X	27min<br>408M	4h<br>3G	41min<br>9G	25min<br>12G
BASS	2min<br>852M	48min<br>5G	1.3h<br>18G	1.4h<br>26G
SpatialPCA	9min<br>759M	1h<br>5.5G	16h<br>29G	19h<br>43G
BayesSpace	30min<br>1G	1h<br>10G	20h<br>52G	12h<br>53G
SpaGCN	1min<br>500M	10min<br>3.6G	44min<br>16G	1h<br>20G

*Computational time and maximum memory usage of the methods for spatial pattern/domain identification and associated/differential gene detection. Using a Linux server with NVIDIA A100 40GB.
