# Supplementary material for: STModule: identifying tissue modules to uncover spatial components and characteristics of transcriptomic landscapes
Source: Genome Med. 2025 Mar 3;17:18. doi: 10.1186/s13073-025-01441-9 (PMC11874447; doi:10.1186/s13073-025-01441-9)
Supplement: Supplementary file 2 — Additional file 2. Supplementary figures S1-S75. [file 13073_2025_1441_MOESM2_ESM.pdf]

Additional file 2: Supplementary figures

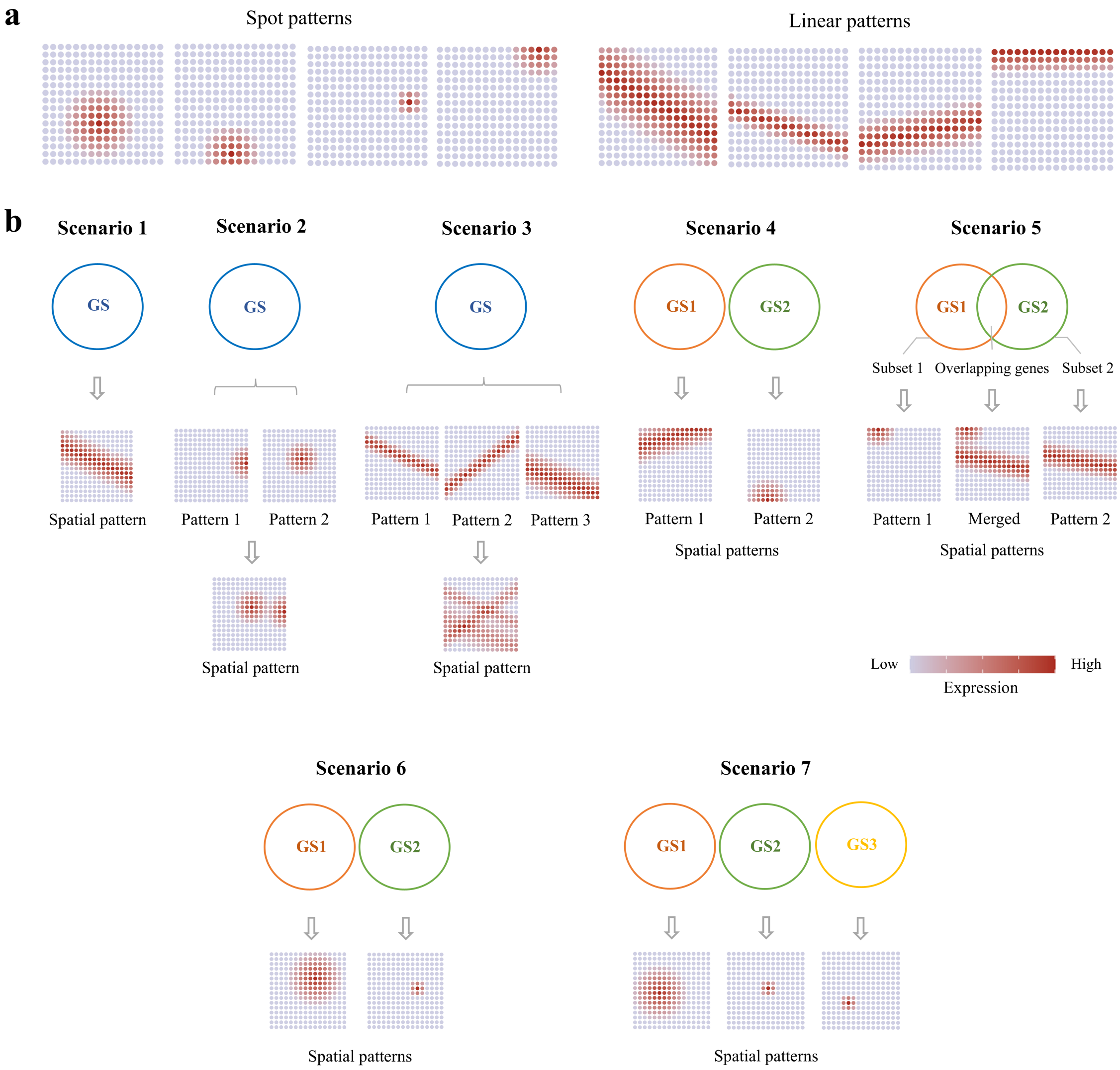

Fig. S1. Simulation strategy of the first set of simulations. a Examples of simulated spot and linear patterns on the 16 × 16 grid. The simulated spot and linear patterns are used to generate simulated spatial profiles in the scenarios. **b** Illustration of simulation strategies of the scenarios. *Scenario 1*: a gene set (GS) expressing a randomly simulated spot/linear pattern. *Scenario 2*: a gene set expressing the spatial pattern generated by merging two randomly simulated spot/linear patterns. *Scenario 3*: a gene set expressing the spatial pattern generated by merging three randomly simulated spot/linear patterns. *Scenario 4*: two individual gene sets with no overlapping genes, each expressing a randomly simulated spot/linear pattern. *Scenario 5*: two gene sets with 50 overlapping genes, each expressing a randomly simulated spot/linear pattern. The overlapping genes express the merged spatial pattern. *Scenario 6*: gene set 1 expressing a more general pattern while gene set 2 expressing a sub-pattern. *Scenario 7*: gene set 1 expressing a more general pattern while gene sets 2 and 3 each expressing a sub-pattern. For all scenarios, each gene set consists of 100 genes. Each simulated spatial expression profile is composed of 1,000 genes, including the spatially expressed genes and genes that randomly express across the locations on the grid.

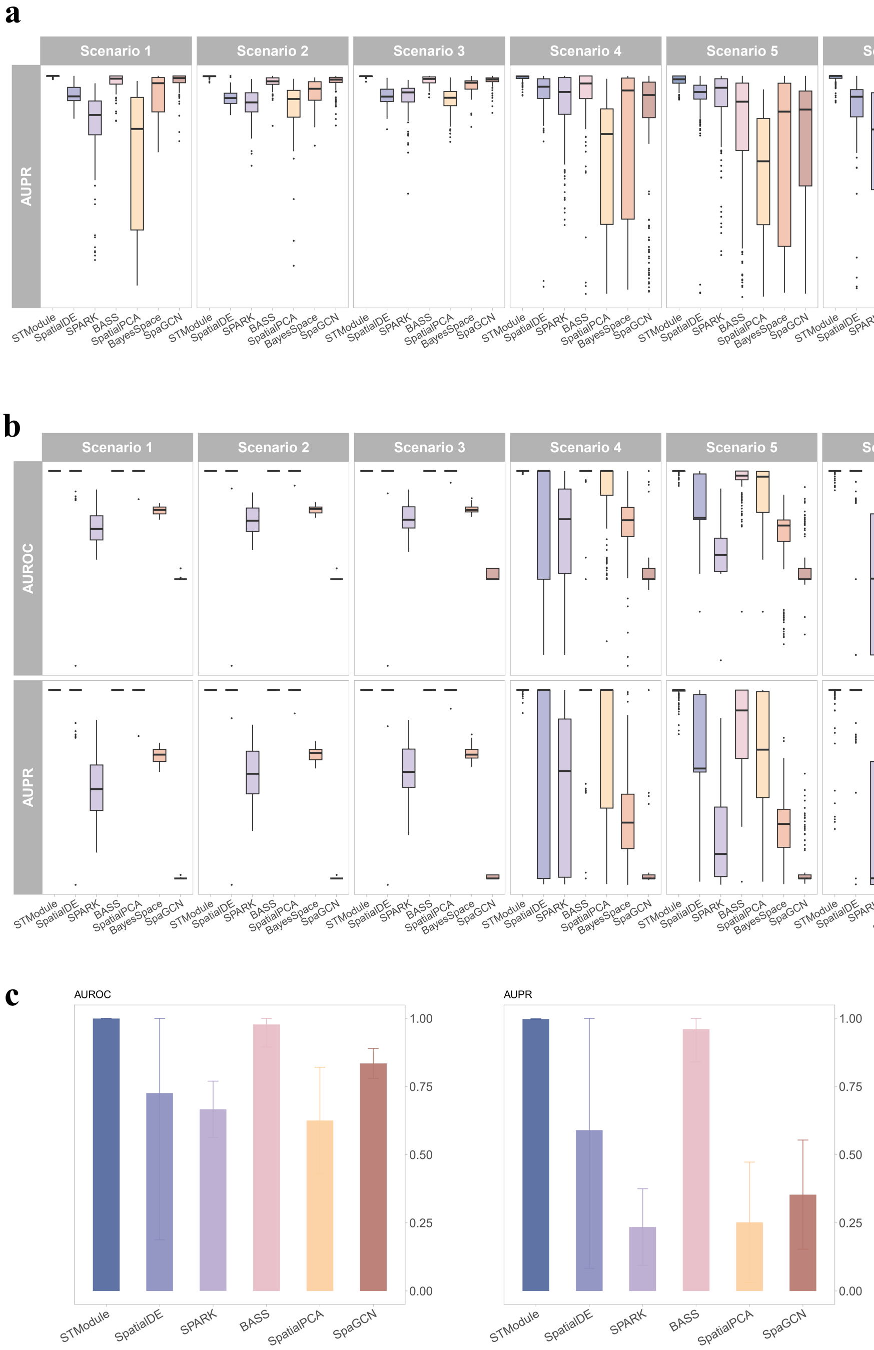
a
b
c
Scenario 1
Scenario 2
Scenario 3
Scenario 4
Scenario 5
Scenario 6
Scenario 7
AUPR
AUROC
1.00
0.75
0.50
0.25
0.00
STModule
SpatialDE
SPARK
BASS
SpatialPCA
BayesSpace
SpaGCN

d

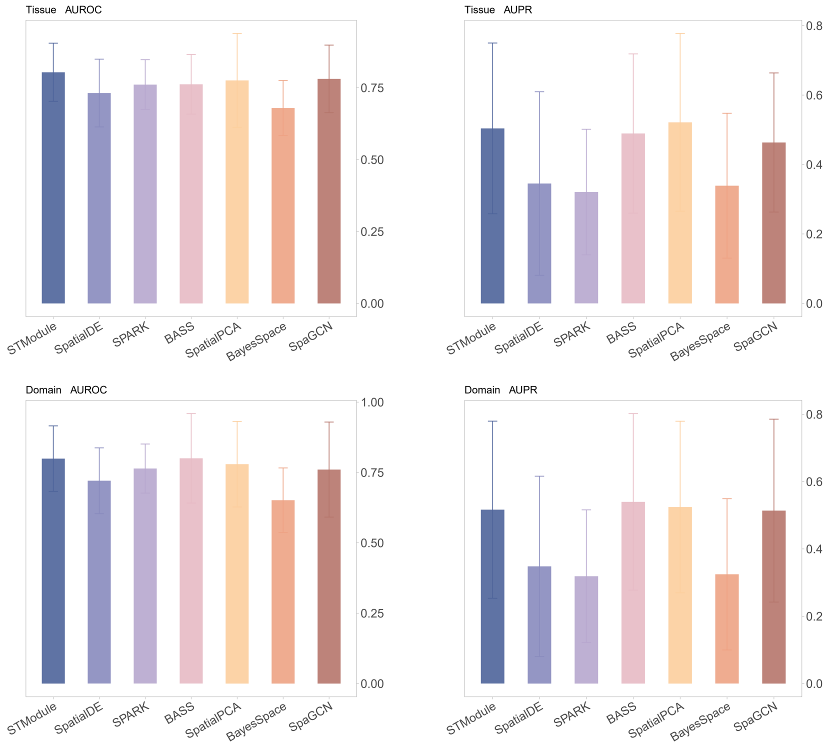

e

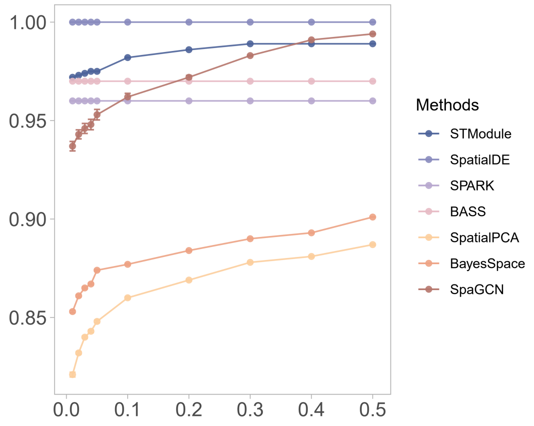

Fig. S2. Additional comparison for simulations. a Comparison of AUPR of the methods in identifying spatial patterns in the first set of simulations. **b** Comparison of AUROC and AUPR of the methods in identifying associated genes in the first set of simulations. **c** Comparison of average AUROC and AUPR of the methods in identifying the layers in the second set of simulations. **d** Comparison of average AUROC and AUPR of the methods in identifying the layers in the third set of simulations, including the tissue-based scenario (top) and the domain-based scenario (bottom). **e** Comparison of FDR-Power curve of the methods in identifying SVGs in the fourth set of simulations.

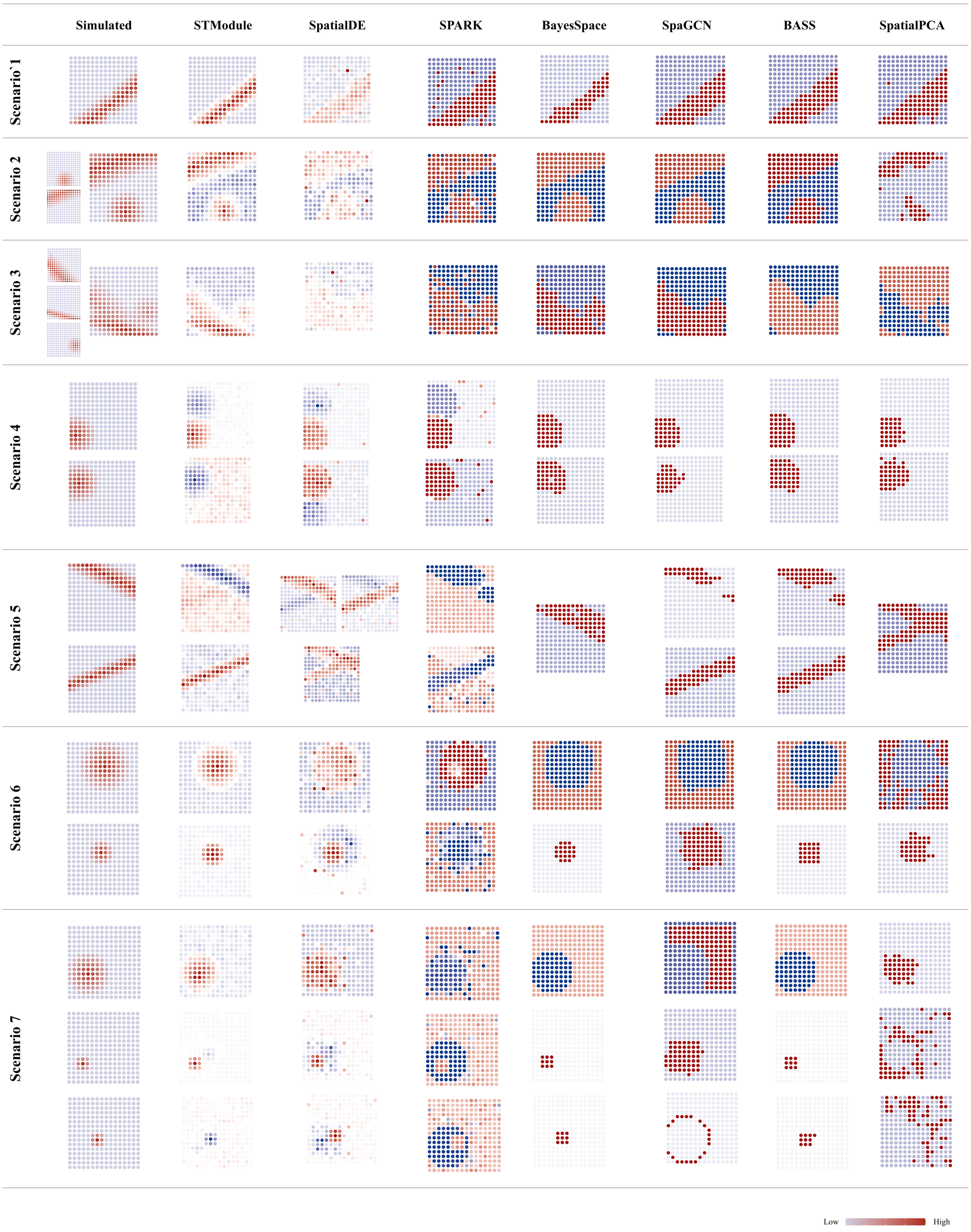

Fig. S3. Examples of simulated spatial patterns in the first set of simulations and corresponding patterns/clusters identified by different methods.

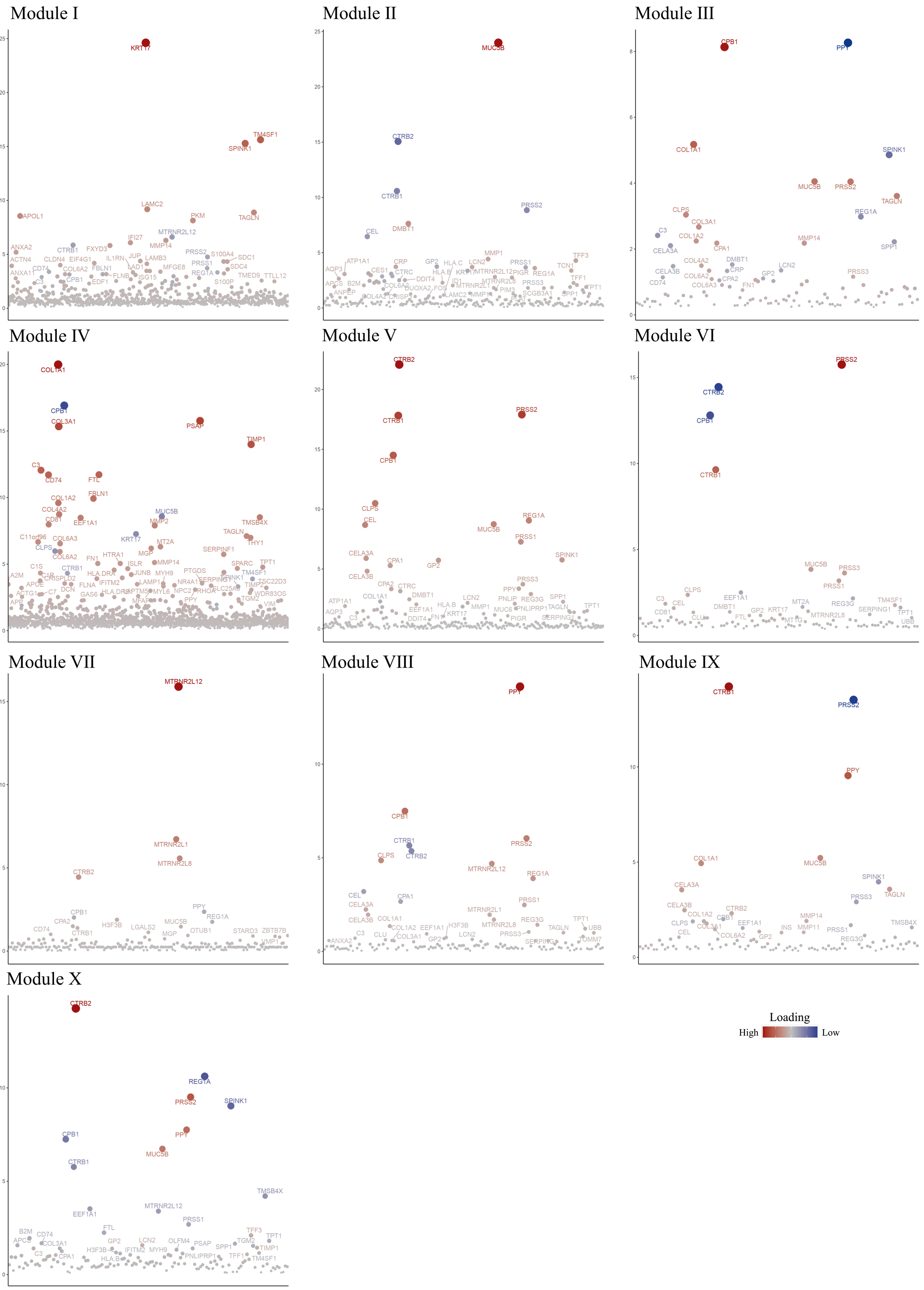

Fig. S4. Gene loadings of the tissue modules identified by STModule from PDAC-A. Y-axis represents the absolute value of gene loading and color indicates the sign and value of loading. X-axis represents the alphabetical order of the gene names.

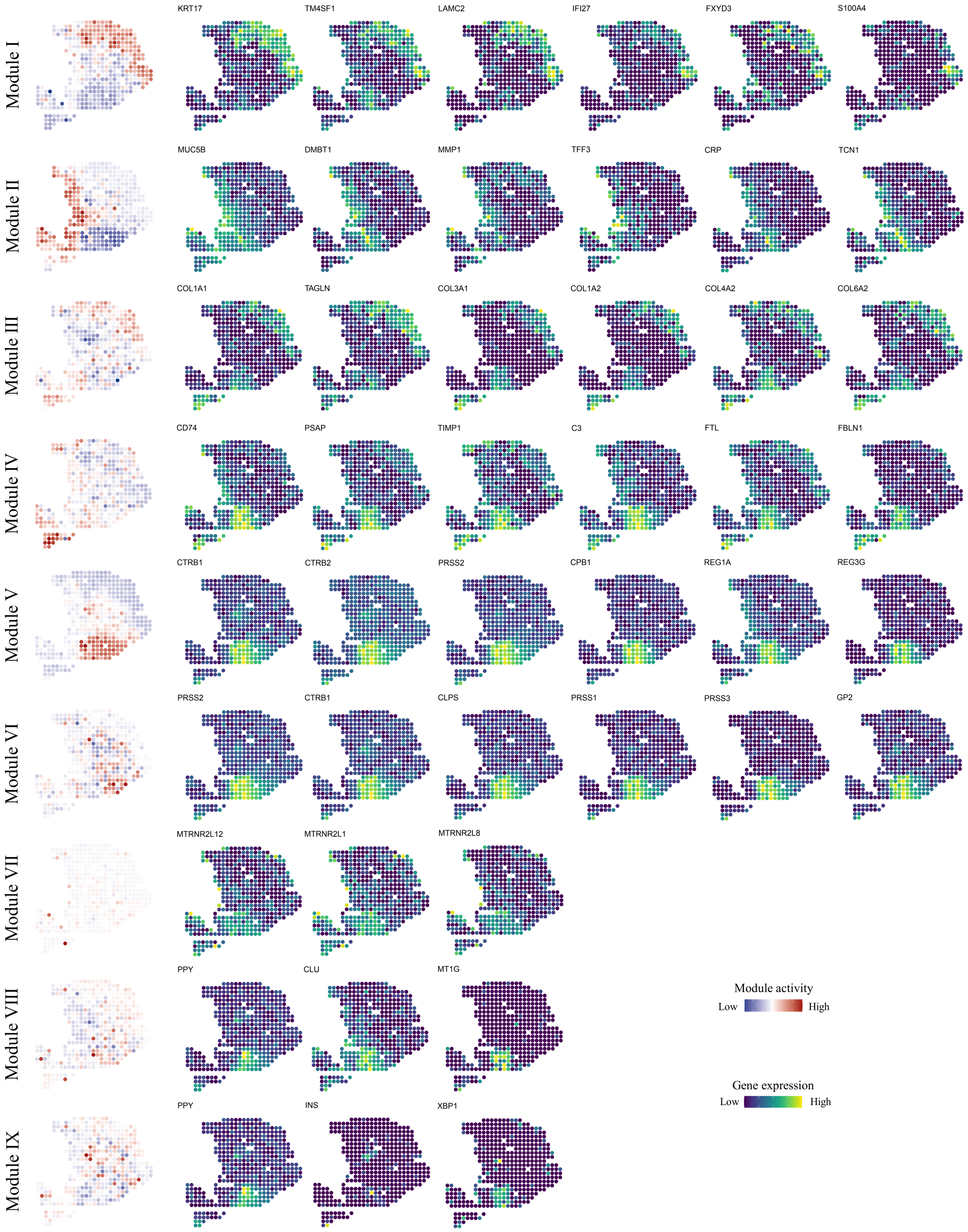

Fig. S5. Spatial expression of representative associated genes of the tissue modules identified by STModule from PDAC-A. Module X is not demonstrated as there is no marker gene of stroma with same spatial pattern as the module.

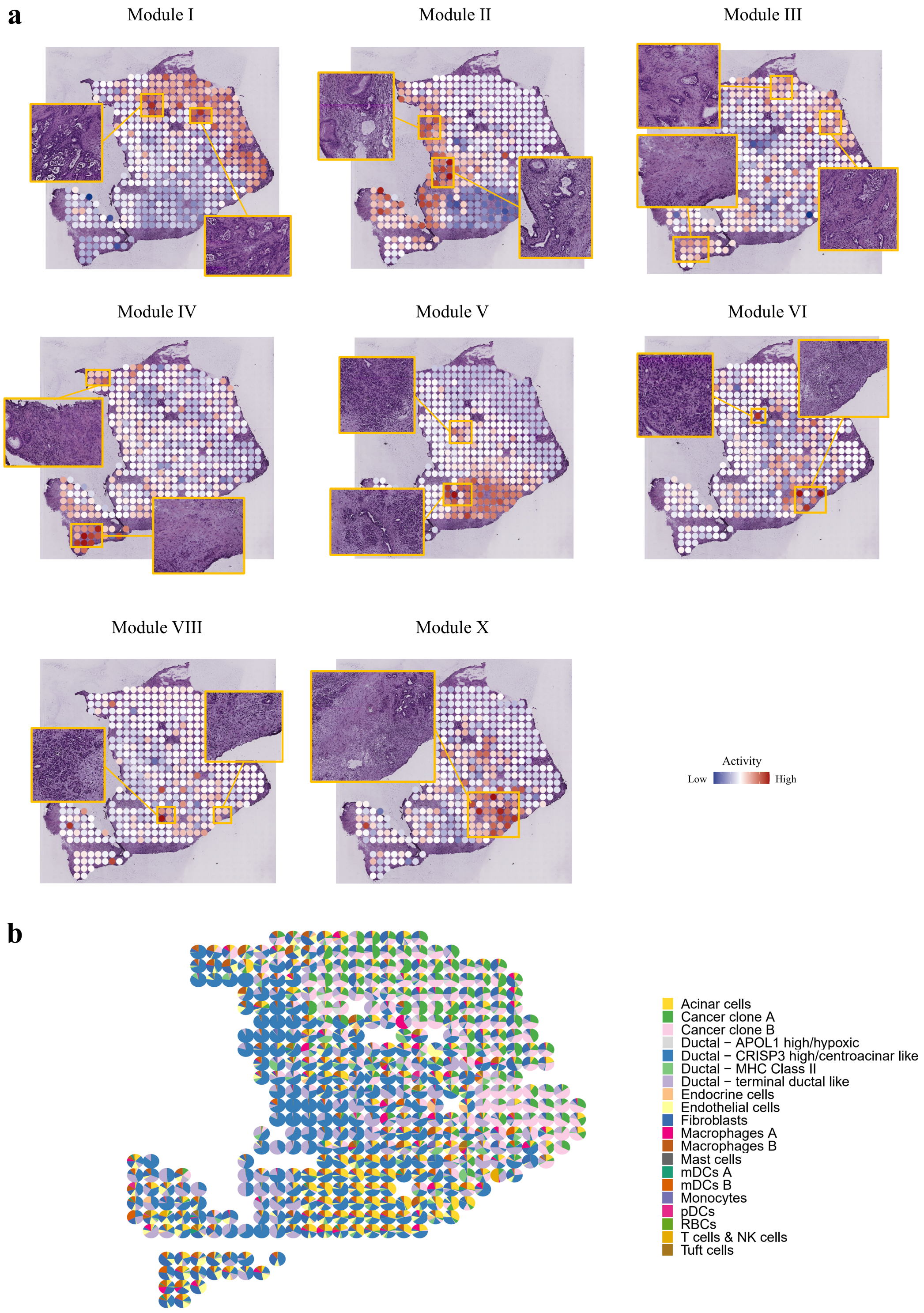

Fig. S6. Additional histological information of PDAC-A. a Illustration of areas in the H&E-stained image with relatively high activities of the modules identified by STModule from PDAC-A. **b** Cell type deconvolution results of PDAC-A.

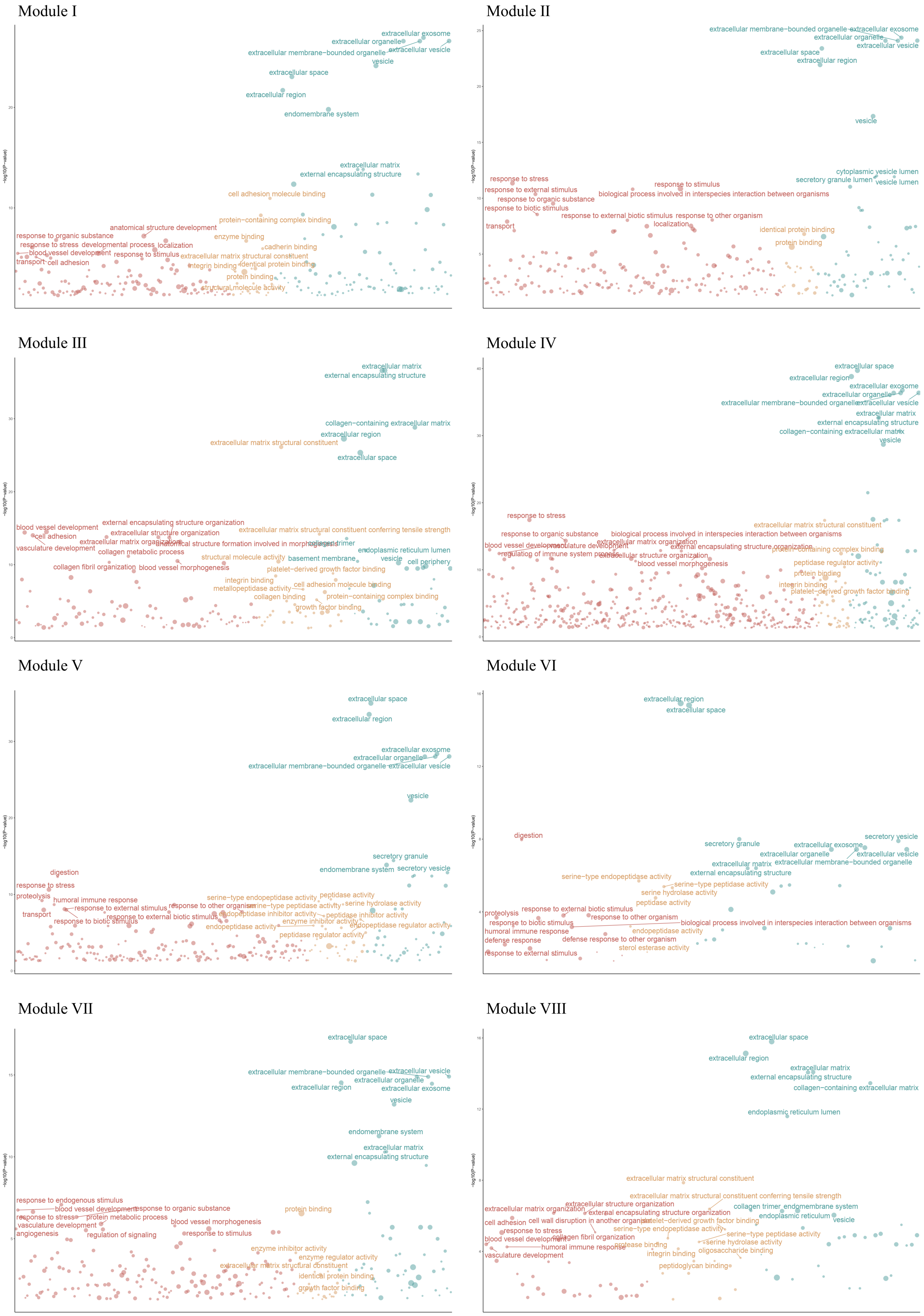
Module I
extracellular exosome
extracellular organelle
extracellular membrane−bounded organelle
extracellular vesicle
vesicle
extracellular space
extracellular region
endomembrane system
extracellular matrix
external encapsulating structure
cell adhesion molecule binding
protein−containing complex binding
anatomical structure development
enzyme binding
response to organic substance
response to stress
developmental process
localization
cadherin binding
blood vessel development
response to stimulus
extracellular matrix structural constituent
transport
cell adhesion
integrin binding
identical protein binding
protein binding
structural molecule activity
-log10(P-value)
Module II
extracellular membrane−bounded organelle
extracellular exosome
extracellular organelle
extracellular vesicle
extracellular space
extracellular region
vesicle
cytoplasmic vesicle lumen
secretory granule lumen
vesicle lumen
response to stress
response to stimulus
response to external stimulus
response to organic substance
biological process involved in interspecies interaction between organisms
response to biotic stimulus
response to external biotic stimulus
response to other organism
localization
transport
identical protein binding
protein binding
Module III
extracellular matrix
external encapsulating structure
collagen−containing extracellular matrix
extracellular region
extracellular matrix structural constituent
extracellular space
blood vessel development
external encapsulating structure organization
cell adhesion
extracellular structure organization
extracellular matrix structural constituent conferring tensile strength
extracellular matrix organization
anatomical structure formation involved in morphogenesis
collagen trimer
vasculature development
collagen metabolic process
endoplasmic reticulum lumen
structural molecule activity
basement membrane
vesicle
cell periphery
collagen fibril organization
blood vessel morphogenesis
platelet−derived growth factor binding
integrin binding
cell adhesion molecule binding
metallopeptidase activity
collagen binding
protein−containing complex binding
growth factor binding
Module IV
extracellular space
extracellular region
extracellular exosome
extracellular organelle
extracellular membrane−bounded organelle
extracellular vesicle
extracellular matrix
external encapsulating structure
collagen−containing extracellular matrix
vesicle
response to stress
extracellular matrix structural constituent
response to organic substance
biological process involved in interspecies interaction between organisms
extracellular matrix organization
blood vessel development
vasculature development
external encapsulating structure organization
regulation of immune system process
extracellular structure organization
protein−containing complex binding
blood vessel morphogenesis
peptidase regulator activity
protein binding
integrin binding
platelet−derived growth factor binding
Module V
extracellular space
extracellular region
extracellular exosome
extracellular organelle
extracellular membrane−bounded organelle
extracellular vesicle
vesicle
secretory granule
endomembrane system
secretory vesicle
digestion
response to stress
proteolysis
serine−type endopeptidase activity
peptidase activity
humoral immune response
serine−type peptidase activity
serine hydrolase activity
response to external stimulus
response to other organism
transport
response to external biotic stimulus
endopeptidase inhibitor activity
peptidase inhibitor activity
response to biotic stimulus
enzyme inhibitor activity
endopeptidase activity
endopeptidase regulator activity
peptidase regulator activity
Module VI
extracellular region
extracellular space
digestion
secretory vesicle
secretory granule
extracellular exosome
extracellular organelle
extracellular vesicle
extracellular matrix
extracellular membrane−bounded organelle
external encapsulating structure
serine−type endopeptidase activity
serine−type peptidase activity
serine hydrolase activity
peptidase activity
proteolysis
response to external biotic stimulus
response to other organism
response to biotic stimulus
biological process involved in interspecies interaction between organisms
humoral immune response
endopeptidase activity
defense response
defense response to other organism
response to external stimulus
sterol esterase activity
Module VII
extracellular space
extracellular membrane−bounded organelle
extracellular vesicle
extracellular organelle
extracellular region
extracellular exosome
vesicle
endomembrane system
extracellular matrix
external encapsulating structure
response to endogenous stimulus
blood vessel development
response to organic substance
protein binding
response to stress
protein metabolic process
vasculature development
blood vessel morphogenesis
angiogenesis
regulation of signaling
response to stimulus
enzyme inhibitor activity
enzyme regulator activity
extracellular matrix structural constituent
identical protein binding
growth factor binding
Module VIII
extracellular space
extracellular region
extracellular matrix
external encapsulating structure
collagen−containing extracellular matrix
endoplasmic reticulum lumen
extracellular matrix structural constituent
extracellular matrix structural constituent conferring tensile strength
extracellular structure organization
collagen trimer
endomembrane system
extracellular matrix organization
external encapsulating structure organization
endoplasmic reticulum
cell adhesion
cell wall disruption in another organism
platelet−derived growth factor binding
vesicle
response to stress
serine−type endopeptidase activity
serine−type peptidase activity
blood vessel development
collagen fibril organization
serine hydrolase activity
humoral immune response
protease binding
integrin binding
oligosaccharide binding
vasculature development
peptidoglycan binding

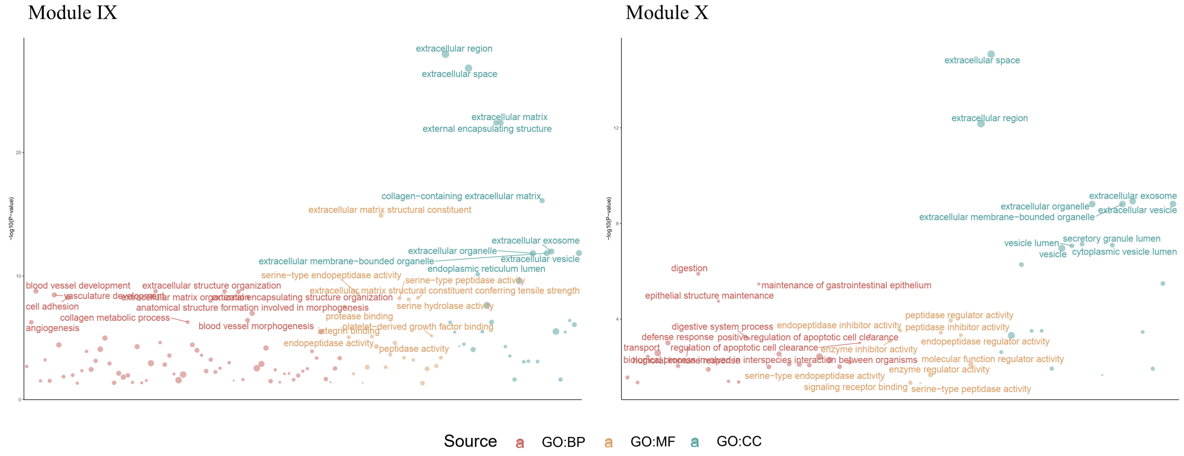

Fig. S7. Gene Ontology (GO) enrichment analysis of the tissue modules identified by STModule from PDAC-A. Y-axis represents -$\log_{10}$(P-value). Color indicates the type of GO terms, including biological process (BP), molecular function (MF) and cellular component (CC). Point size indicates the number of overlapping genes of the modules and the GO terms. X-axis represents the id and type of the GO terms.

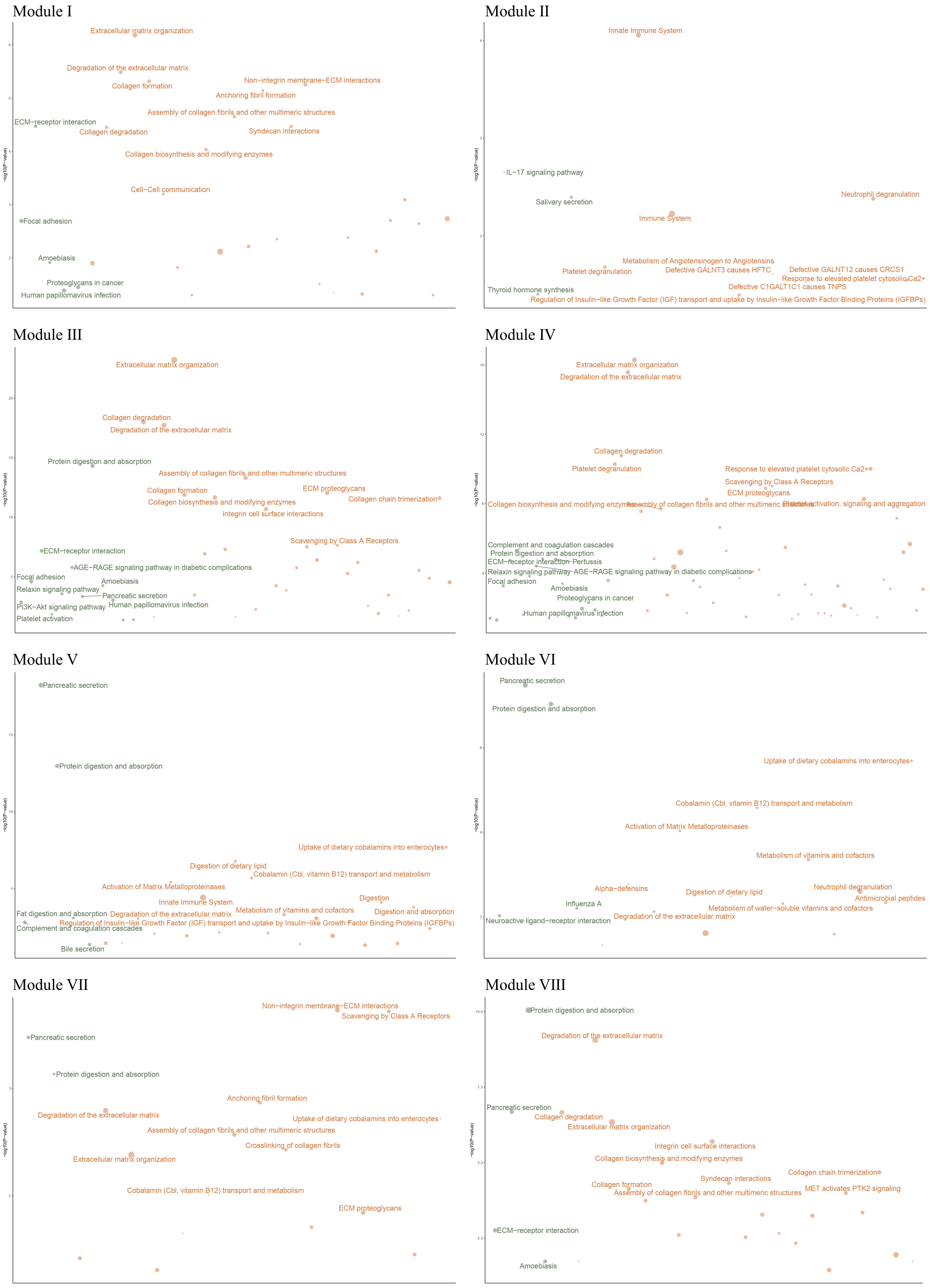

Module I
Extracellular matrix organization
Degradation of the extracellular matrix
Collagen formation
Non−integrin membrane−ECM interactions
Anchoring fibril formation
Assembly of collagen fibrils and other multimeric structures
ECM−receptor interaction
Collagen degradation
Syndecan interactions
Collagen biosynthesis and modifying enzymes
Cell−Cell communication
Focal adhesion
Amoebiasis
Proteoglycans in cancer
Human papillomavirus infection
-log10(P-value)
Module II
Innate Immune System
IL−17 signaling pathway
Salivary secretion
Neutrophil degranulation
Immune System
Metabolism of Angiotensinogen to Angiotensins
Platelet degranulation
Defective GALNT3 causes HFTC
Defective GALNT12 causes CRCS1
Response to elevated platelet cytosolic Ca2+
Defective C1GALT1C1 causes TNPS
Thyroid hormone synthesis
Regulation of Insulin−like Growth Factor (IGF) transport and uptake by Insulin−like Growth Factor Binding Proteins (IGFBPs)
-log10(P-value)
Module III
Extracellular matrix organization
Collagen degradation
Degradation of the extracellular matrix
Protein digestion and absorption
Assembly of collagen fibrils and other multimeric structures
Collagen formation
ECM proteoglycans
Collagen biosynthesis and modifying enzymes
Collagen chain trimerization
Integrin cell surface interactions
Scavenging by Class A Receptors
ECM−receptor interaction
AGE−RAGE signaling pathway in diabetic complications
Focal adhesion
Amoebiasis
Relaxin signaling pathway
Pancreatic secretion
PI3K−Akt signaling pathway
Human papillomavirus infection
Platelet activation
-log10(P-value)
Module IV
Extracellular matrix organization
Degradation of the extracellular matrix
Collagen degradation
Platelet degranulation
Response to elevated platelet cytosolic Ca2+
Scavenging by Class A Receptors
ECM proteoglycans
Collagen biosynthesis and modifying enzymes
Assembly of collagen fibrils and other multimeric structures
Platelet activation, signaling and aggregation
Complement and coagulation cascades
Protein digestion and absorption
ECM−receptor interaction
Pertussis
Relaxin signaling pathway
AGE−RAGE signaling pathway in diabetic complications
Focal adhesion
Amoebiasis
Proteoglycans in cancer
Human papillomavirus infection
-log10(P-value)
Module V
Pancreatic secretion
Protein digestion and absorption
Uptake of dietary cobalamins into enterocytes
Digestion of dietary lipid
Cobalamin (Cbl, vitamin B12) transport and metabolism
Activation of Matrix Metalloproteinases
Innate Immune System
Digestion
Metabolism of vitamins and cofactors
Fat digestion and absorption
Degradation of the extracellular matrix
Digestion and absorption
Regulation of Insulin−like Growth Factor (IGF) transport and uptake by Insulin−like Growth Factor Binding Proteins (IGFBPs)
Complement and coagulation cascades
Bile secretion
-log10(P-value)
Module VI
Pancreatic secretion
Protein digestion and absorption
Uptake of dietary cobalamins into enterocytes
Cobalamin (Cbl, vitamin B12) transport and metabolism
Activation of Matrix Metalloproteinases
Metabolism of vitamins and cofactors
Alpha−defensins
Digestion of dietary lipid
Neutrophil degranulation
Antimicrobial peptides
Influenza A
Metabolism of water−soluble vitamins and cofactors
Neuroactive ligand−receptor interaction
Degradation of the extracellular matrix
-log10(P-value)
Module VII
Non−integrin membrane−ECM interactions
Scavenging by Class A Receptors
Pancreatic secretion
Protein digestion and absorption
Anchoring fibril formation
Degradation of the extracellular matrix
Uptake of dietary cobalamins into enterocytes
Assembly of collagen fibrils and other multimeric structures
Crosslinking of collagen fibrils
Extracellular matrix organization
Cobalamin (Cbl, vitamin B12) transport and metabolism
ECM proteoglycans
-log10(P-value)
Module VIII
Protein digestion and absorption
Degradation of the extracellular matrix
Pancreatic secretion
Collagen degradation
Extracellular matrix organization
Integrin cell surface interactions
Collagen biosynthesis and modifying enzymes
Collagen chain trimerization
Syndecan interactions
Collagen formation
Assembly of collagen fibrils and other multimeric structures
MET activates PTK2 signaling
ECM−receptor interaction
Amoebiasis
-log10(P-value)

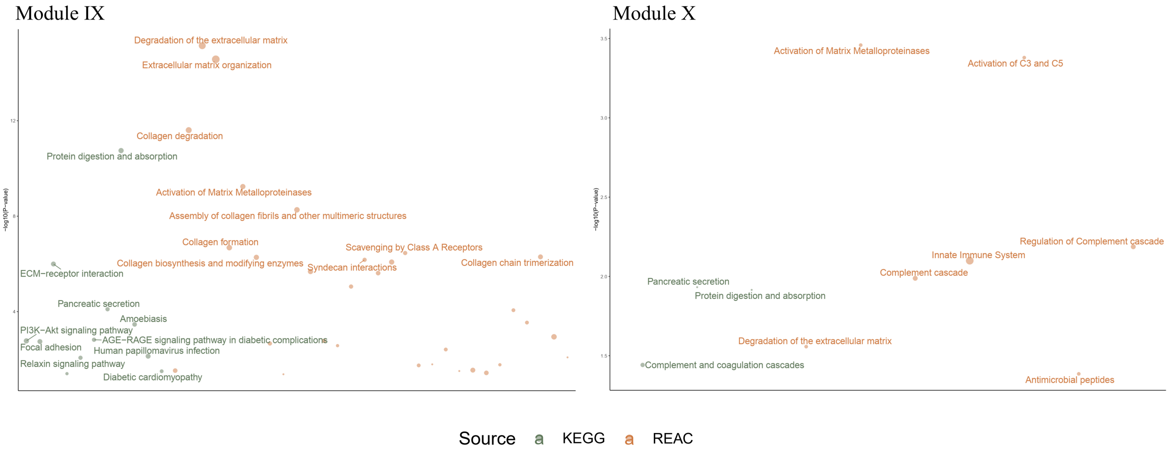

Fig. S8. Functional enrichment analysis of the tissue modules identified by STModule from PDAC-A. Y-axis represents -$\log_{10}$(P-value). Color indicates the source of pathways, including Kyoto Encyclopedia of Genes and Genomes (KEGG) and Reactome. Point size indicates the number of overlapping genes of the modules and the pathways. X-axis represents the id and type of the KEGG and Reactome terms.

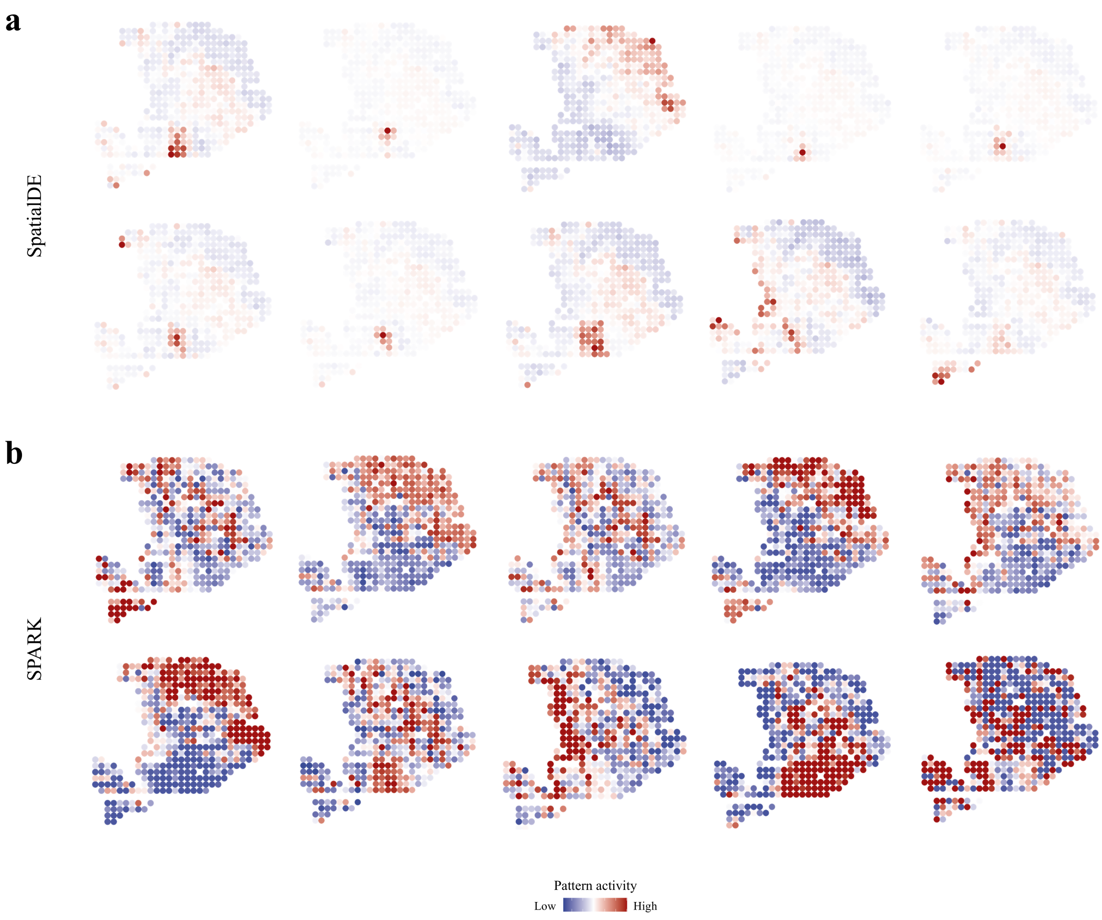

Fig. S9. Spatial patterns identified by SpatialDE (a) and SPARK (b) for PDAC-A.

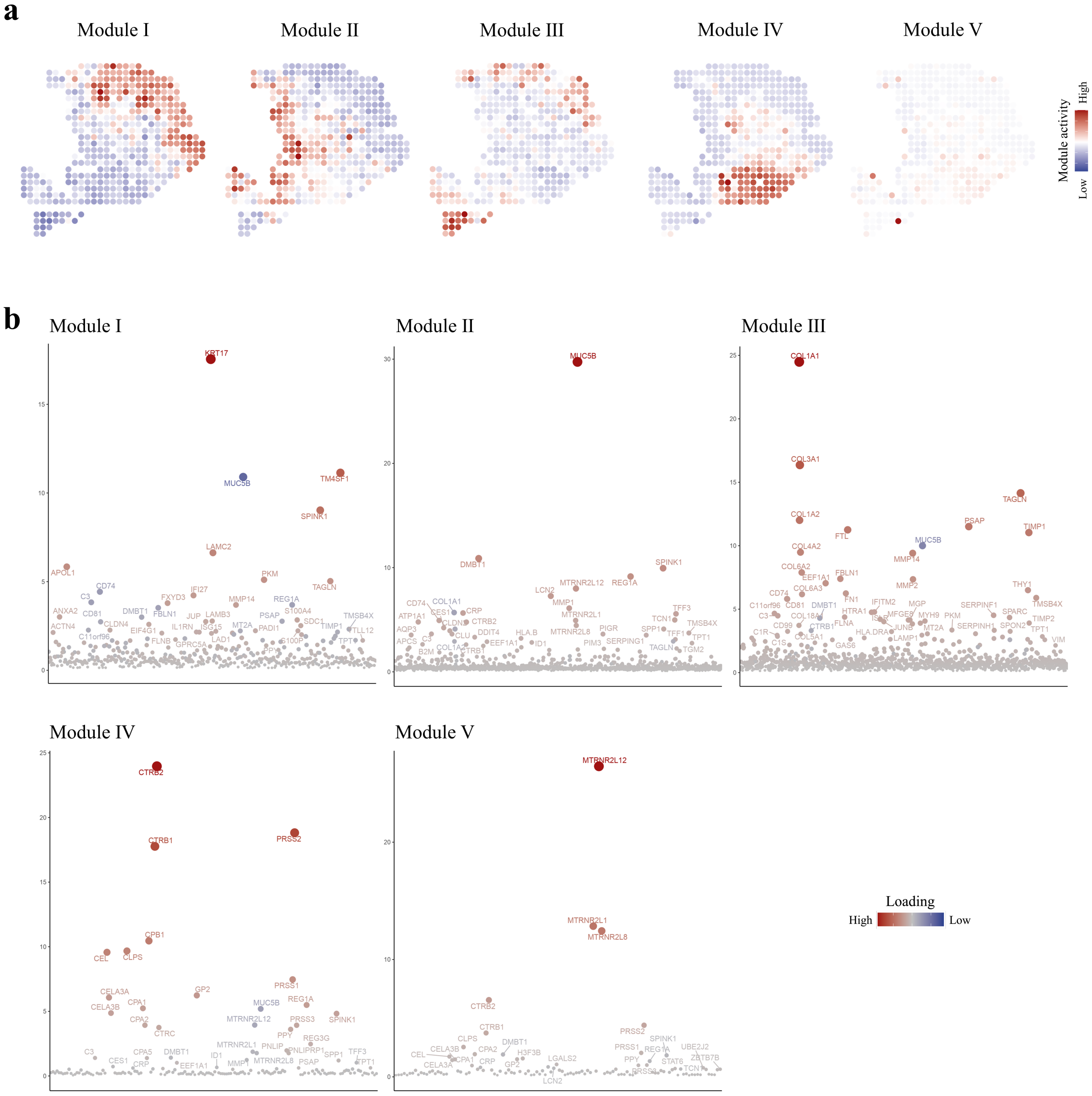

Fig. S10. Tissue modules identified by STModule from PDAC-A with 5 latent factors. a Spatial maps of the tissue modules. **b** Gene loadings of the tissue modules. Y-axis represents the absolute value of gene loading and color indicates the sign and value of loading. X-axis represents the alphabetical order of the gene names.

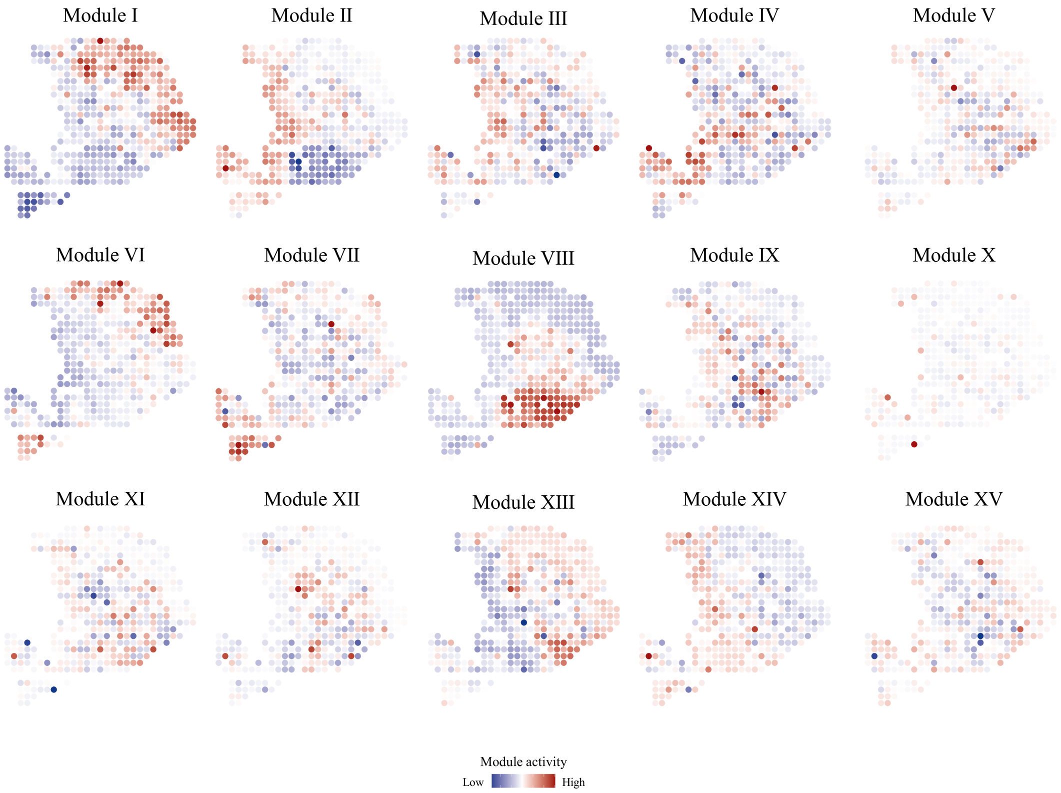

Fig. S11. Spatial maps of the tissue modules identified by STModule from PDAC-A with 15 latent factors.

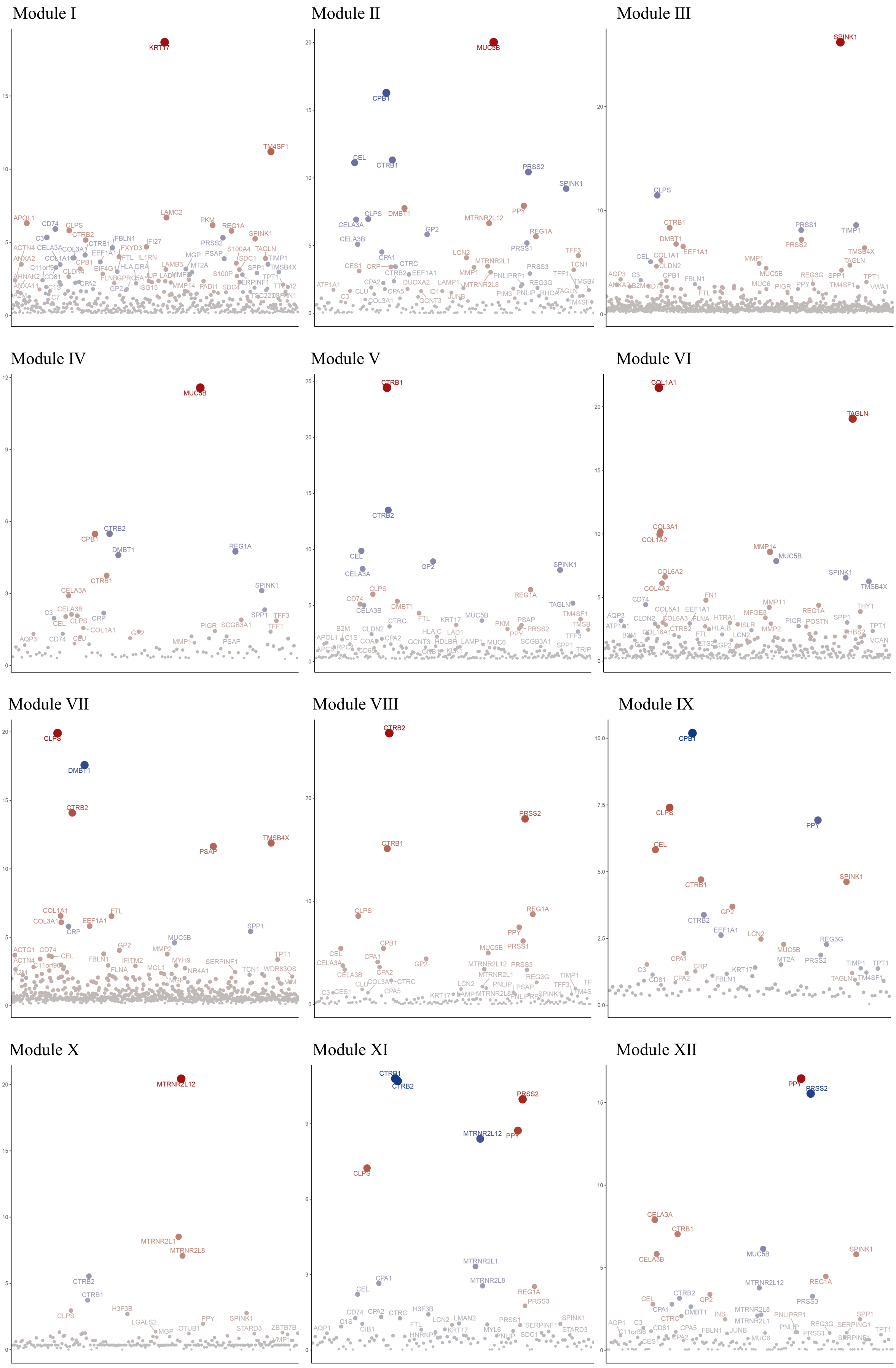
Module I
KRT17
TM4SF1
Module II
MUC5B
CPB1
CEL
CTRB1
PRSS2
SPINK1
Module III
SPINK1
CLPS
Module IV
MUC5B
CTRB2
CPB1
DMBT1
REG1A
Module V
CTRB1
CTRB2
Module VI
COL1A1
TAGLN
COL3A1
COL1A2
Module VII
CLPS
DMBT1
CTRB2
TMSB4X
PSAP
Module VIII
CTRB2
PRSS2
CTRB1
Module IX
CPB1
CLPS
PPY
CEL
Module X
MTRNR2L12
MTRNR2L1
MTRNR2L8
Module XI
CTRB1
CTRB2
PRSS2
MTRNR2L12
PPY
CLPS
Module XII
PPY
PRSS2
CELA3A
CTRB1

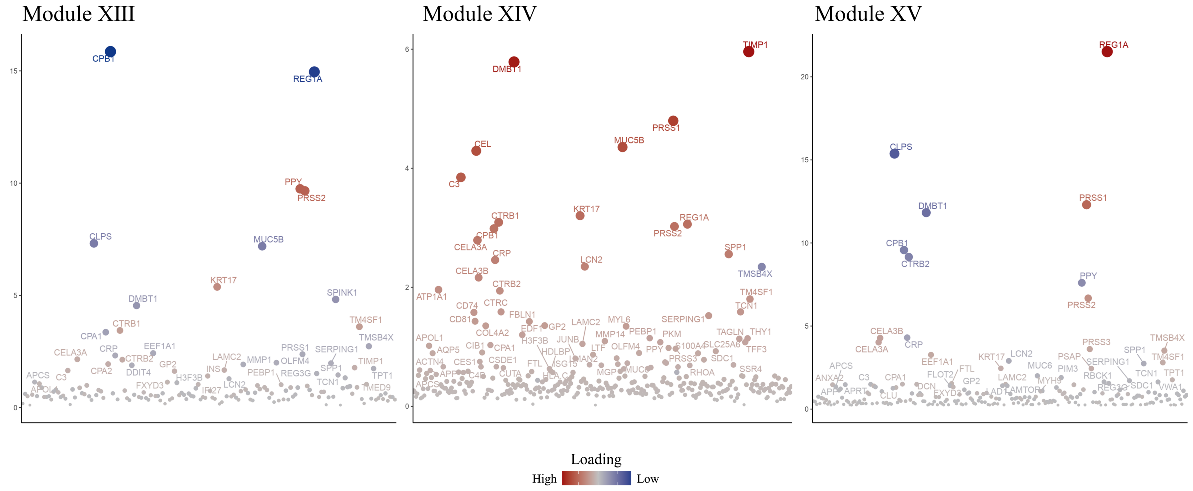

Fig. S12. Gene loadings of the tissue modules identified by STModule from PDAC-A with 15 latent factors. Y-axis represents the absolute value of gene loading and color indicates the sign and value of loading. X-axis represents the alphabetical order of the gene names.

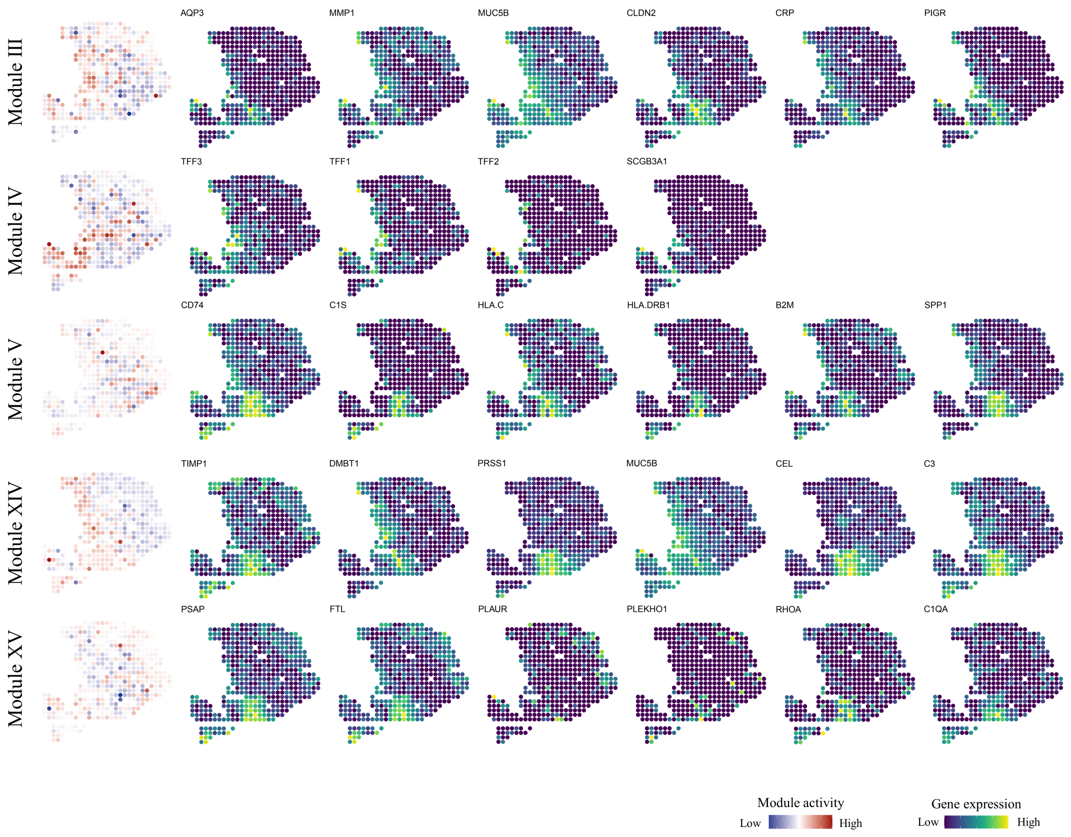

Fig. S13. Spatial expression of representative associated genes of additional tissue modules identified by STModule from PDAC-A with 15 latent factors.

a

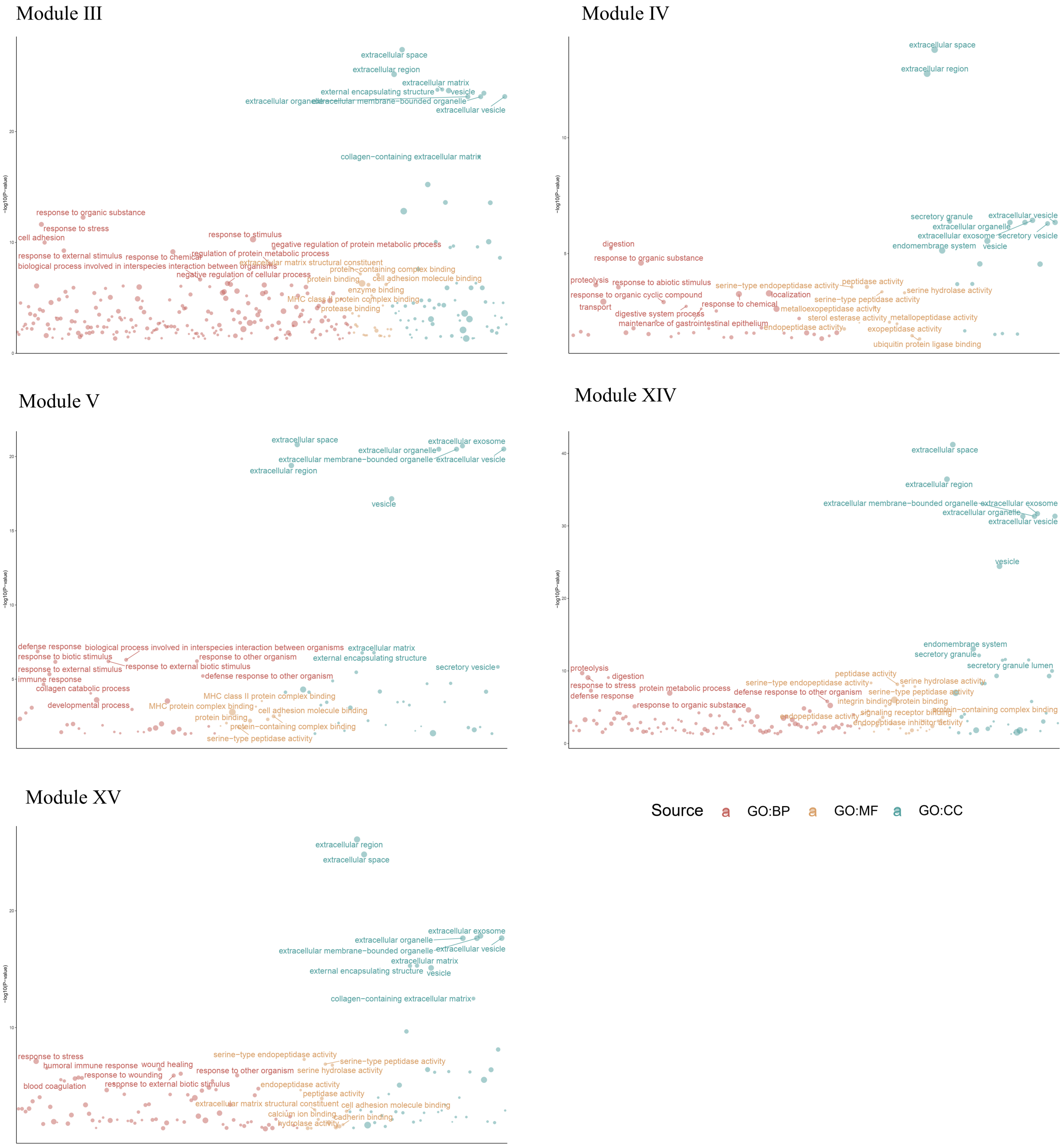
Module III
extracellular space
extracellular region
extracellular matrix
external encapsulating structure
vesicle
extracellular organelle
extracellular membrane−bounded organelle
extracellular vesicle
collagen−containing extracellular matrix
response to organic substance
response to stress
cell adhesion
response to stimulus
negative regulation of protein metabolic process
response to external stimulus
response to chemical
regulation of protein metabolic process
biological process involved in interspecies interaction between organisms
extracellular matrix structural constituent
negative regulation of cellular process
protein−containing complex binding
protein binding
cell adhesion molecule binding
enzyme binding
MHC class II protein complex binding
protease binding
-log10(P-value)
Module IV
extracellular space
extracellular region
secretory granule
extracellular vesicle
extracellular organelle
extracellular exosome
secretory vesicle
endomembrane system
vesicle
digestion
response to organic substance
proteolysis
response to abiotic stimulus
serine−type endopeptidase activity
peptidase activity
serine hydrolase activity
response to organic cyclic compound
localization
serine−type peptidase activity
response to chemical
transport
metalloexopeptidase activity
digestive system process
sterol esterase activity
metallopeptidase activity
maintenance of gastrointestinal epithelium
endopeptidase activity
exopeptidase activity
ubiquitin protein ligase binding
Module V
extracellular space
extracellular exosome
extracellular organelle
extracellular membrane−bounded organelle
extracellular vesicle
extracellular region
vesicle
defense response
biological process involved in interspecies interaction between organisms
extracellular matrix
response to biotic stimulus
response to other organism
external encapsulating structure
response to external stimulus
response to external biotic stimulus
secretory vesicle
immune response
defense response to other organism
collagen catabolic process
MHC class II protein complex binding
developmental process
MHC protein complex binding
cell adhesion molecule binding
protein binding
protein−containing complex binding
serine−type peptidase activity
Module XIV
extracellular space
extracellular region
extracellular membrane−bounded organelle
extracellular exosome
extracellular organelle
extracellular vesicle
vesicle
endomembrane system
secretory granule
secretory granule lumen
proteolysis
digestion
peptidase activity
response to stress
serine−type endopeptidase activity
serine hydrolase activity
protein metabolic process
defense response to other organism
serine−type peptidase activity
defense response
integrin binding
protein binding
response to organic substance
protein−containing complex binding
endopeptidase activity
signaling receptor binding
endopeptidase inhibitor activity
Module XV
extracellular region
extracellular space
extracellular exosome
extracellular organelle
extracellular membrane−bounded organelle
extracellular vesicle
extracellular matrix
external encapsulating structure
vesicle
collagen−containing extracellular matrix
response to stress
serine−type endopeptidase activity
humoral immune response
wound healing
serine−type peptidase activity
response to wounding
response to other organism
serine hydrolase activity
blood coagulation
response to external biotic stimulus
endopeptidase activity
peptidase activity
extracellular matrix structural constituent
cell adhesion molecule binding
calcium ion binding
cadherin binding
hydrolase activity
Source
GO:BP
GO:MF
GO:CC

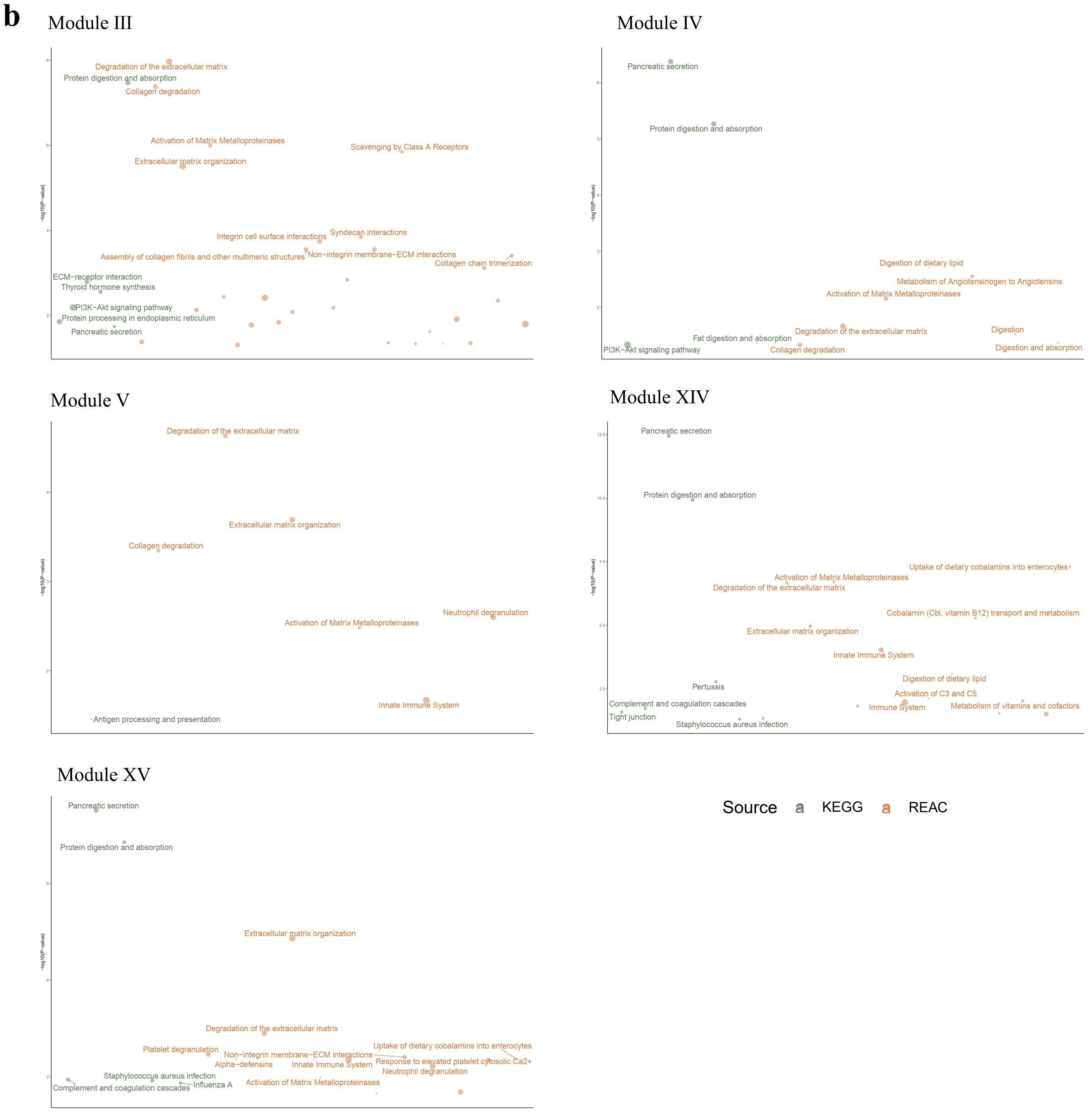

Fig. S14. Functional enrichment analysis of additional tissue modules identified by STModule from PDAC-A with 15 latent factors. a GO enrichment analysis. **b** KEGG and Reactome pathway enrichment analysis. Y-axis represents -$\log_{10}$(P-value). Color indicates the source of functional terms. Point size indicates the number of overlapping genes of the modules and the terms. X-axis represents the id and type of the terms.

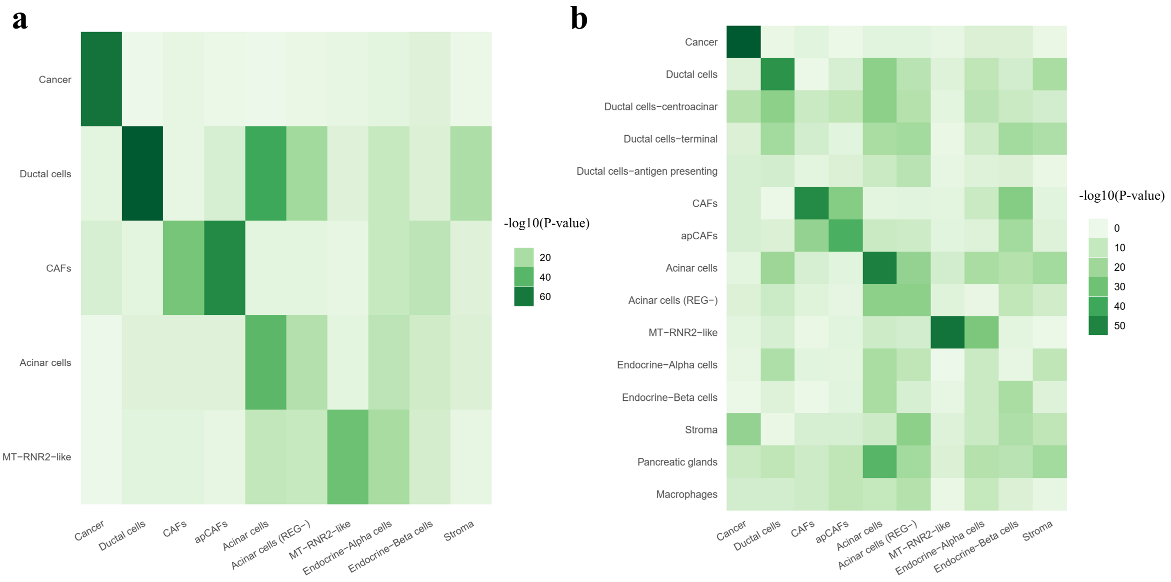

Fig. S15. Comparison of top-ranking genes in the tissue modules identified by STModule from PDAC-A with 5, 10 and 15 latent factors. a Comparison of tissue modules identified with 10 (x-axis) and 5 (y-axis) latent factors. **b** Comparison of tissue modules identified with 10 (x-axis) and 15 (y-axis) latent factors. Tissue modules are indicated by their annotations. Color indicates -$\log_{10}$(P-value) of hypergeometric test between the top 50 genes with the highest loadings of the modules.

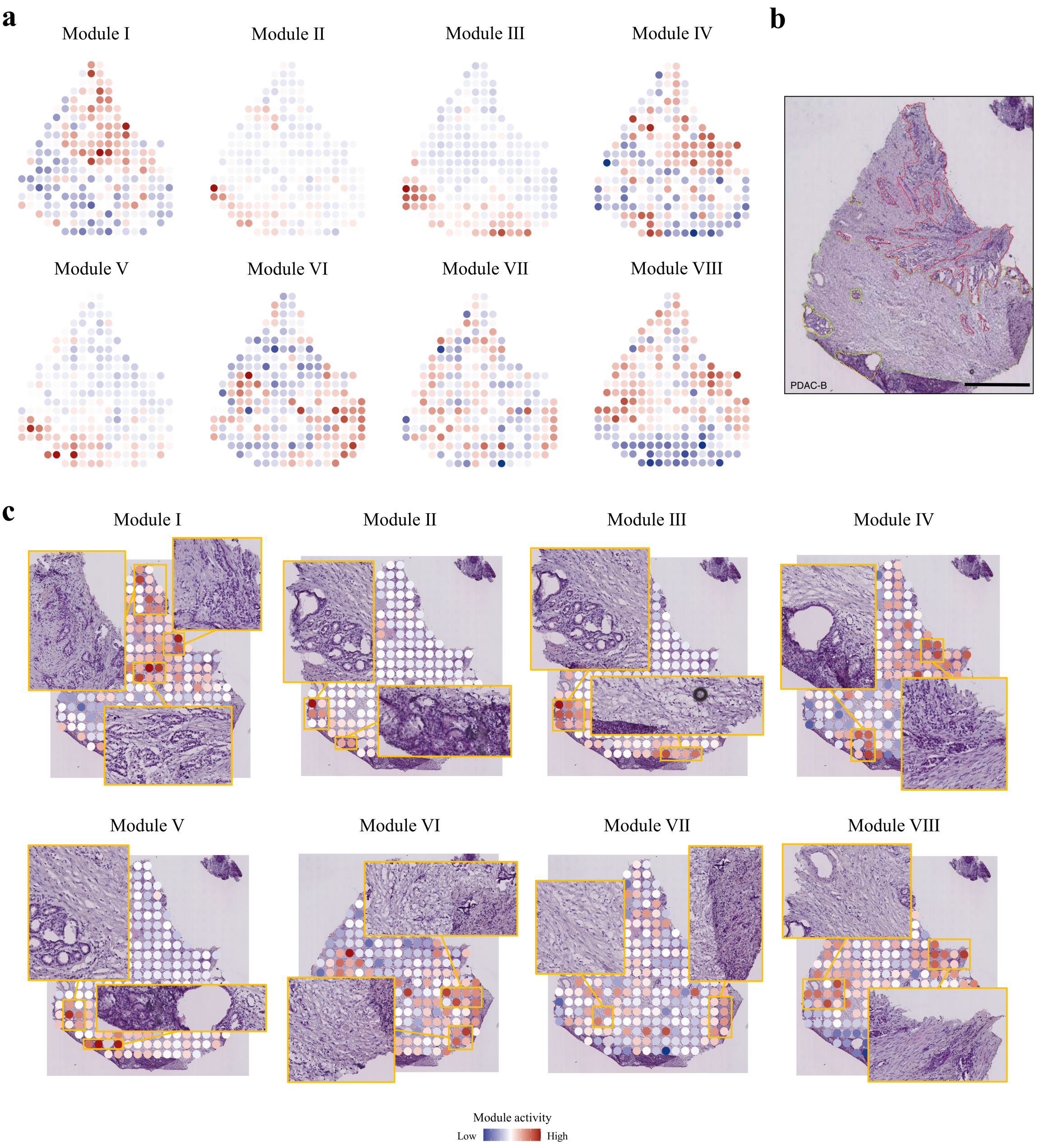

Fig. S16. Tissue modules identified by STModule from PDAC-B. a Spatial maps of the tissue modules. **b** Histological annotation of the tissue in the original study[4]. Red, cancer cells and desmoplasia. Yellow, duct epithelium. Green, interstitium. **c** Illustration of areas in the H&E-stained image of the tissue with relatively high activities of the modules. Annotations of the modules: cancer cells (I), duct epithelium (II), ductal cells-terminal (III), atypical ductal cells (IV), acinar cells (V), fibroblasts-1 (VI), fibroblasts-2 (VII), fibroblasts-3 (VIII).

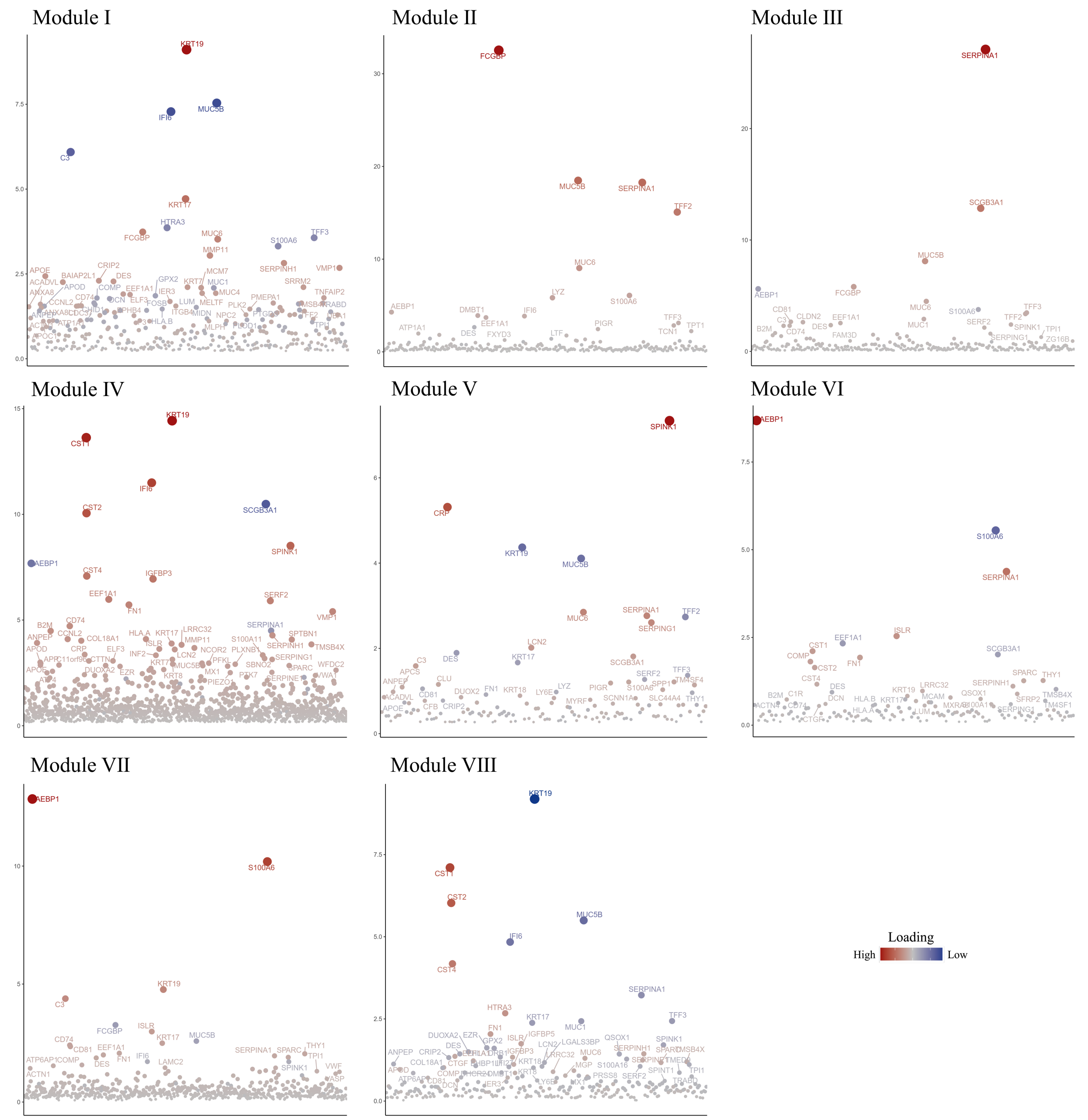

Fig. S17. Gene loadings of the tissue modules identified by STModule from PDAC-B. Y-axis represents the absolute value of gene loading and color indicates the sign and value of loading. X-axis represents the alphabetical order of the gene names.

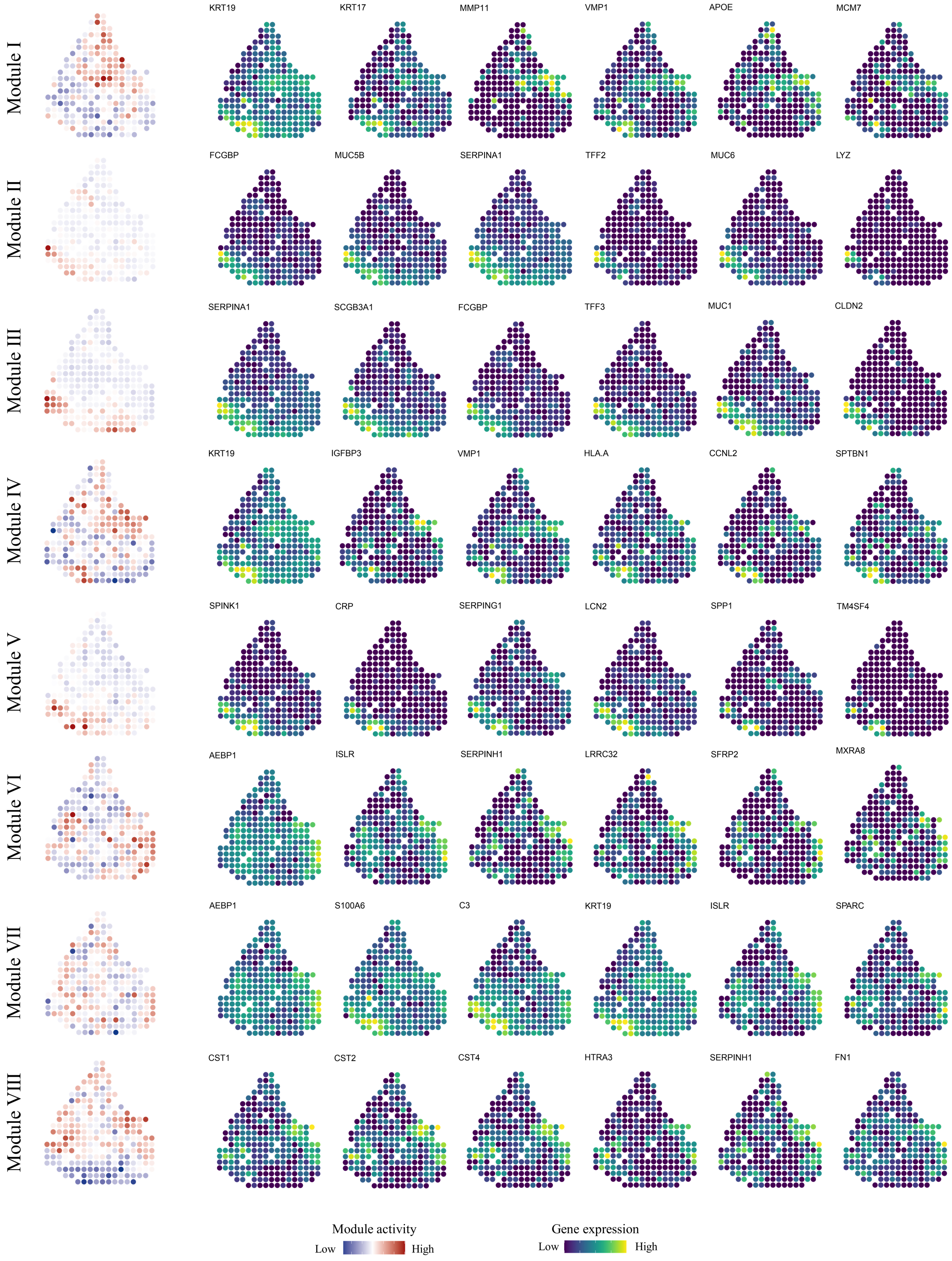

Fig. S18. Spatial expression of representative associated genes of the tissue modules identified by STModule from PDAC-B.

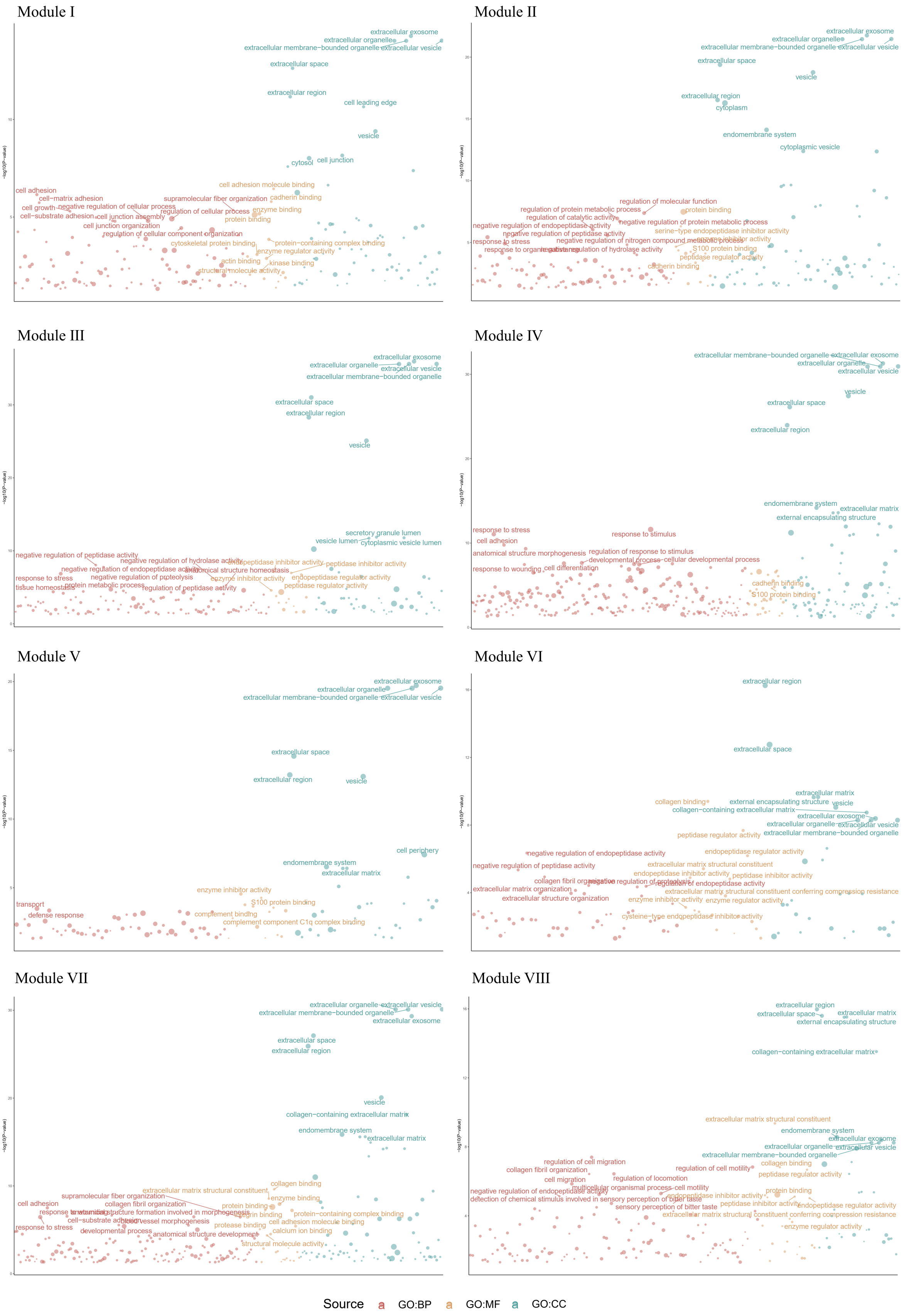

Fig. S19. GO enrichment analysis of the tissue modules identified by STModule from PDAC-B. Y-axis represents -log$_{10}$(P-value). Color indicates the source of the GO terms. Point size indicates the number of overlapping genes of the modules and the terms. X-axis represents the id and type of the terms.

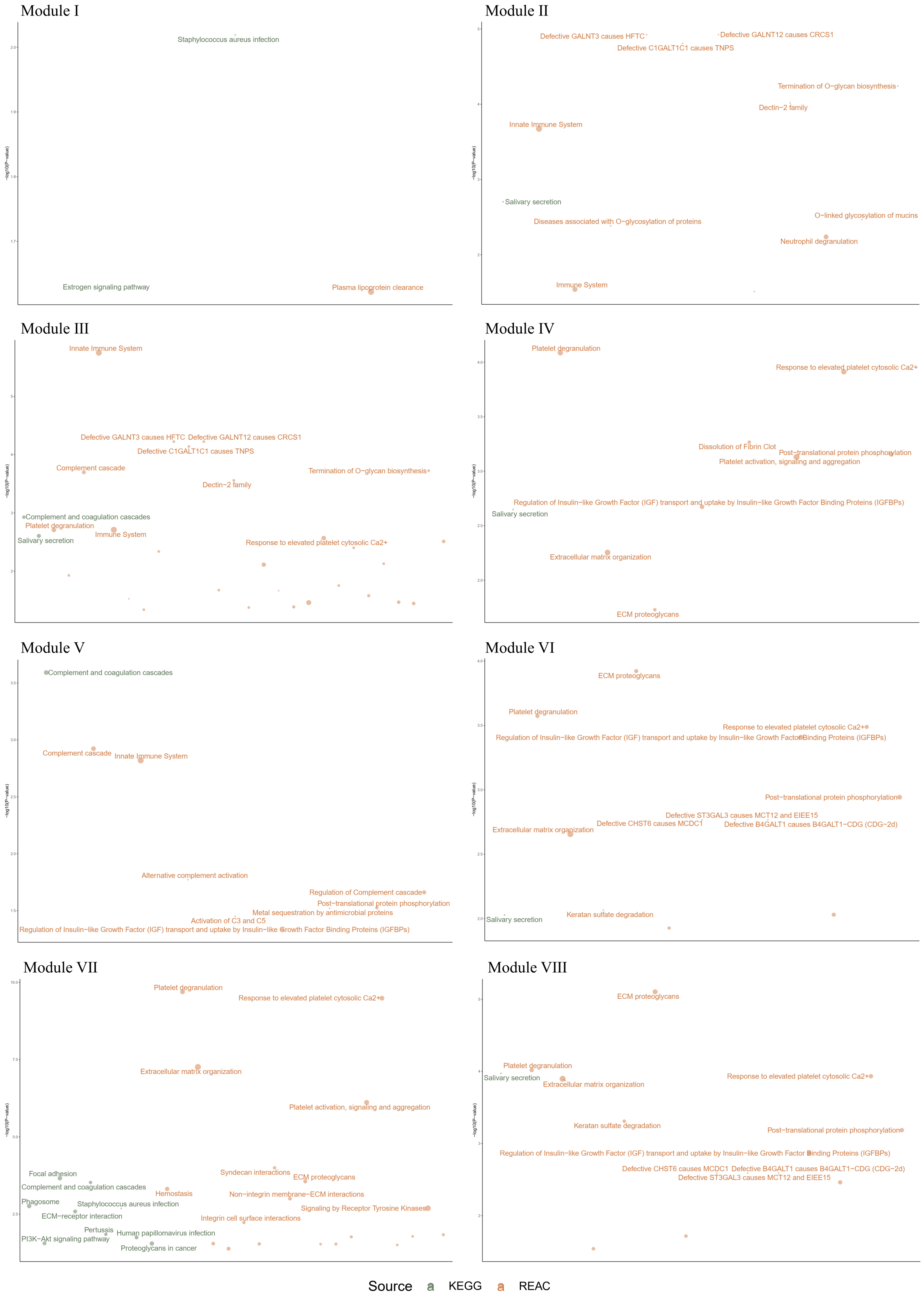

Fig. S20. Functional enrichment analysis of the tissue modules identified by STModule from PDAC-B. Y-axis represents $-\log_{10}$(P-value). Color indicates the source of the pathways. Point size indicates the number of overlapping genes of the modules and the pathways. X-axis represents the id and type of the pathways.

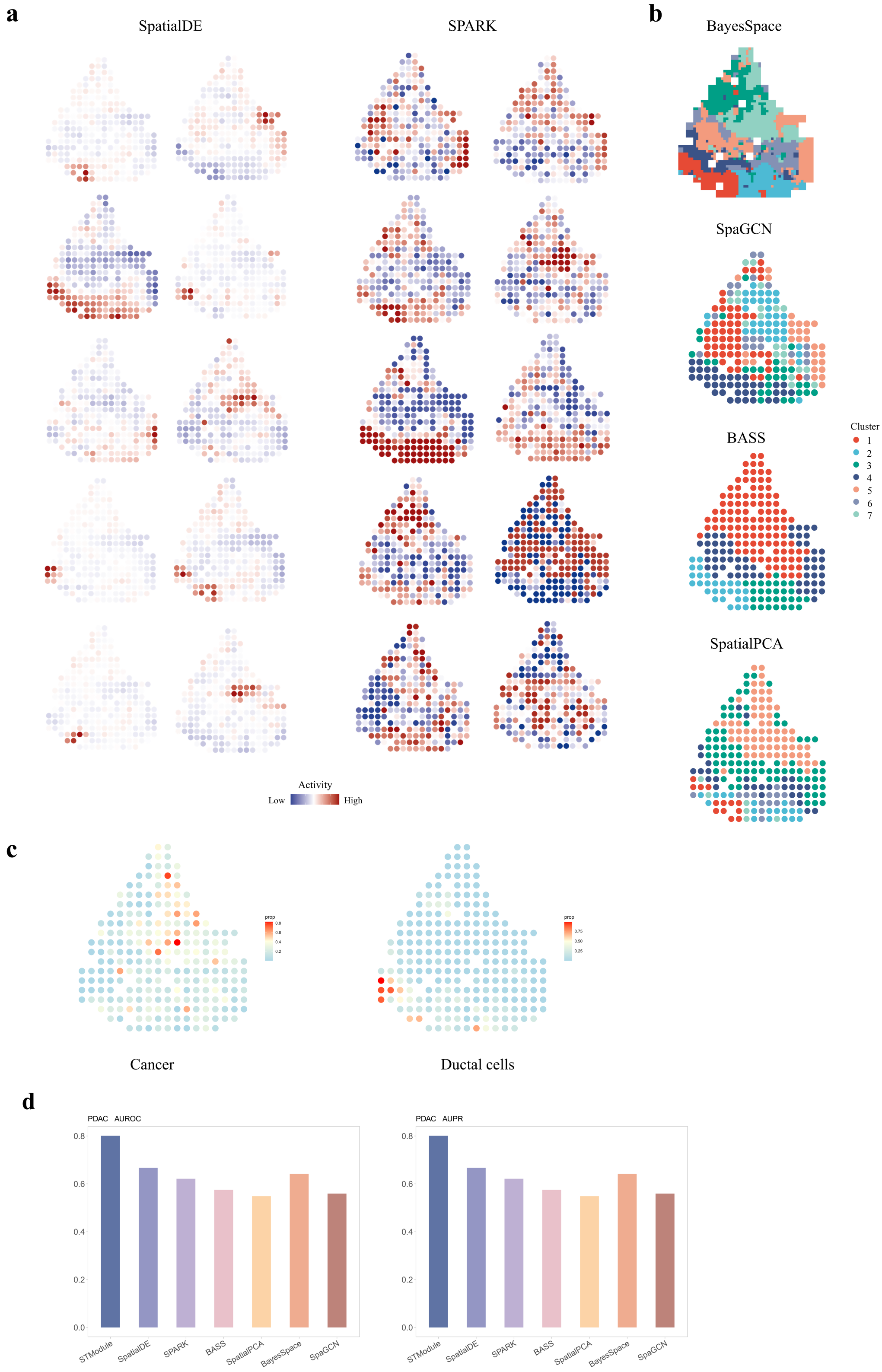

Fig. S21. Additional comparison for PDAC. a Spatial patterns identified by SpatialDE and SPARK from PDAC-B. **b** Results of clustering methods for PDAC-B. **c** Spatial distributions of cancer and ductal cells in PDAC-B estimated by CARD. **d** Comparison of AUROC and AUPR of the methods in detecting the domains of PDAC-A and PDAC-B.

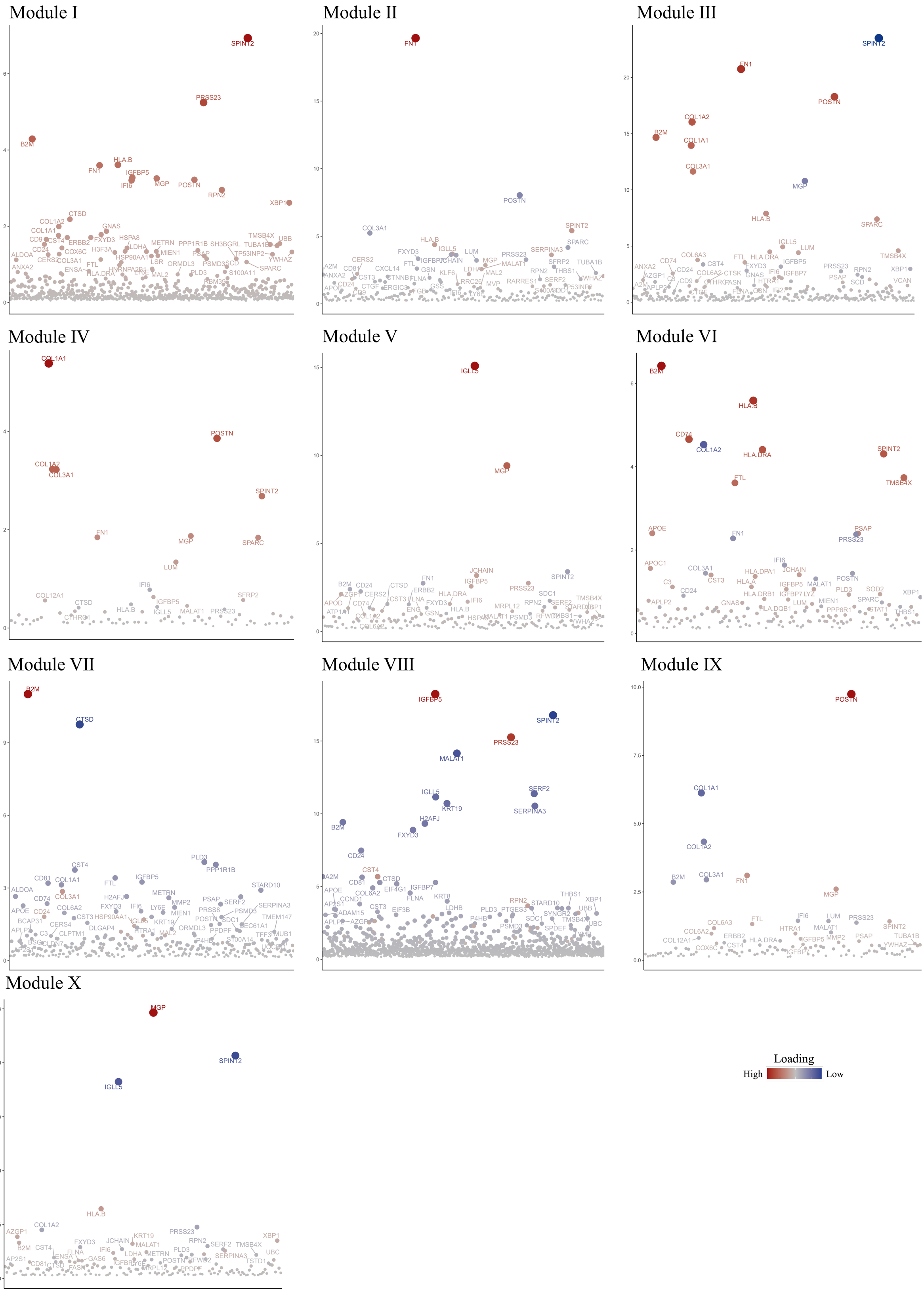

Fig. S22. Gene loadings of the tissue modules identified by STModule from BC layer 2. Y-axis represents the absolute value of gene loading and color indicates the sign and value of loading. X-axis represents the alphabetical order of the gene names.

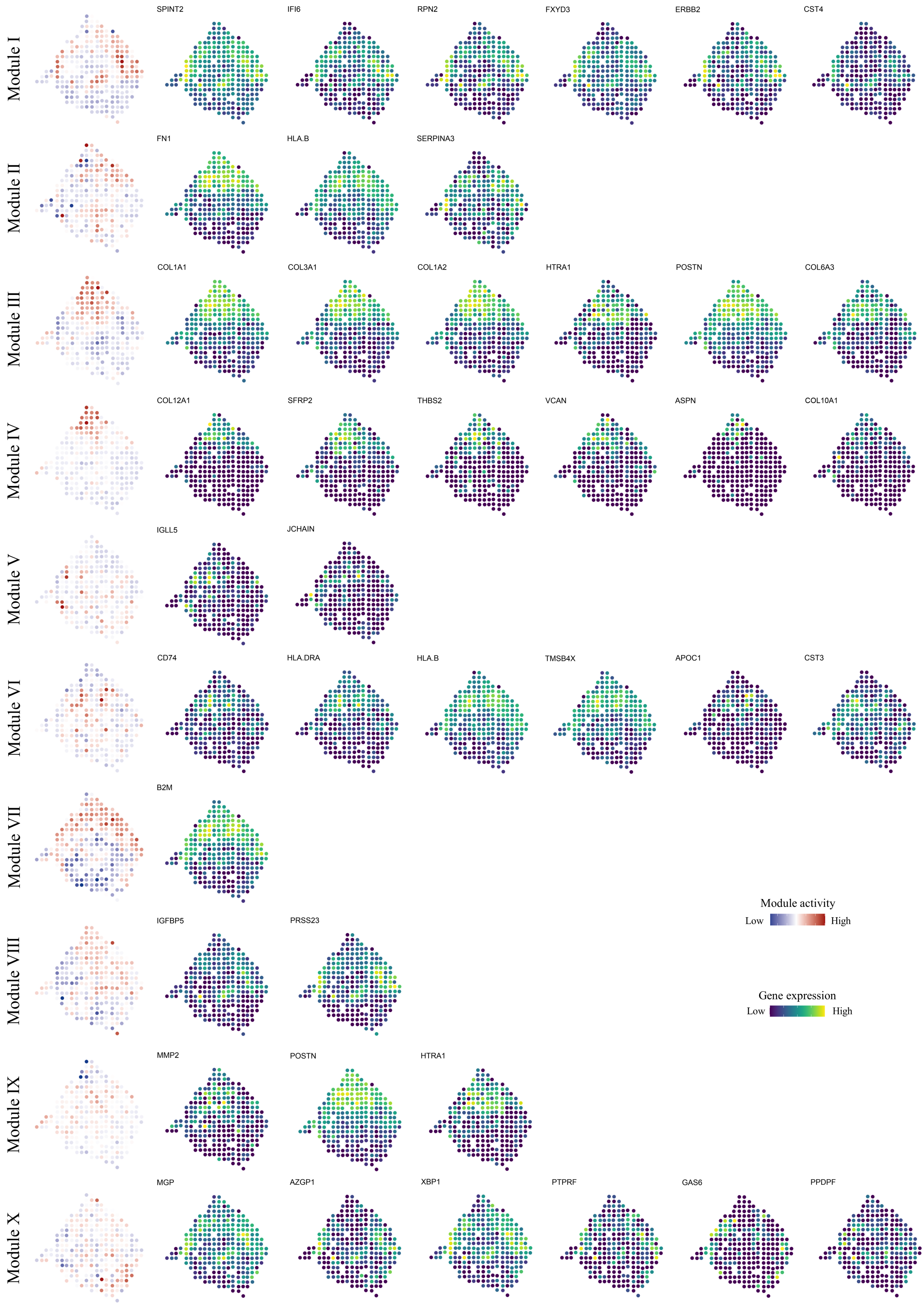

Fig. S23. Spatial expression of representative associated genes of the tissue modules identified by STModule from BC layer 2. Annotations of the modules: ductal carcinoma in situ (DCIS) (I), invasive

ductal cancer (INV) (II), CAFs (III), myCAFs (IV), immune - B cells (V), immune – myeloid cells (VI), immune – B2M (VII), perivascular cells (VIII), endothelial cells (IX), normal breast glands (X).

Module I

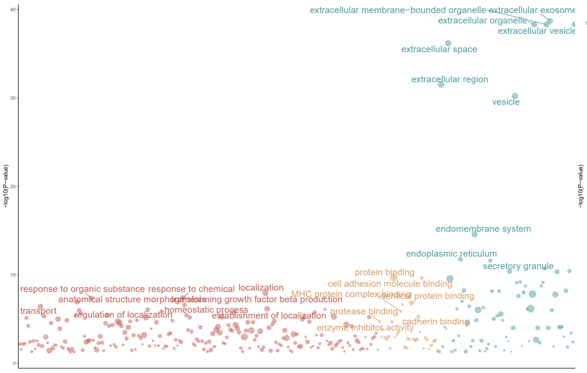

Module II

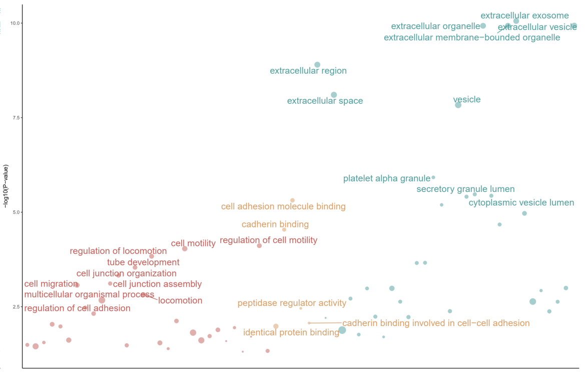

Module III

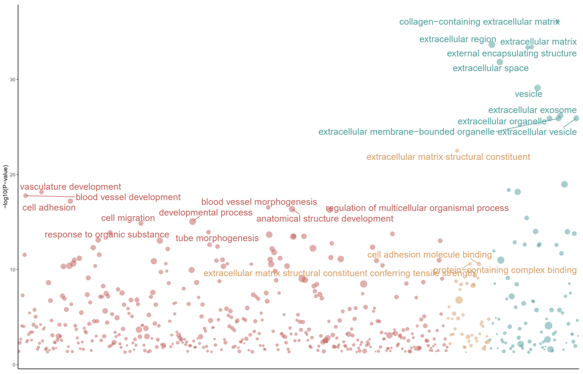

Module IV

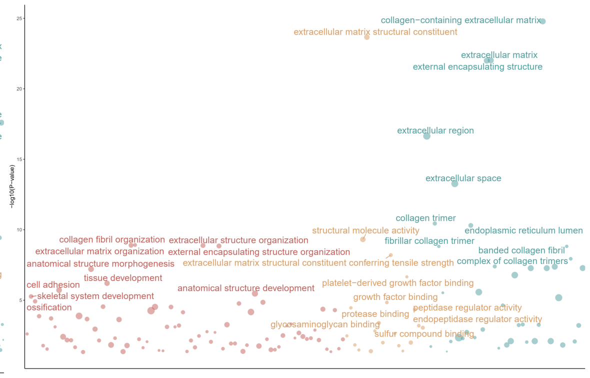

Module V

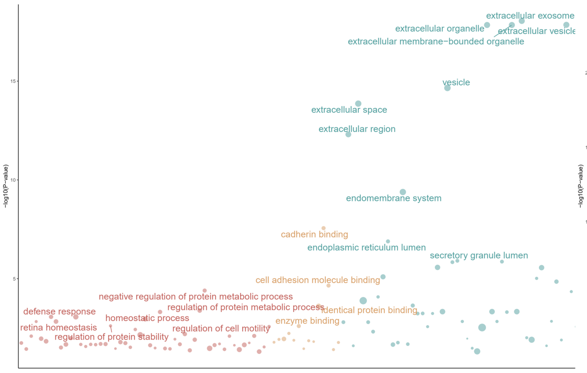

Module VI

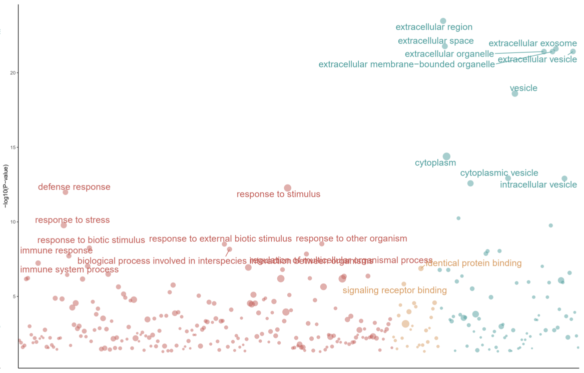

Module VII

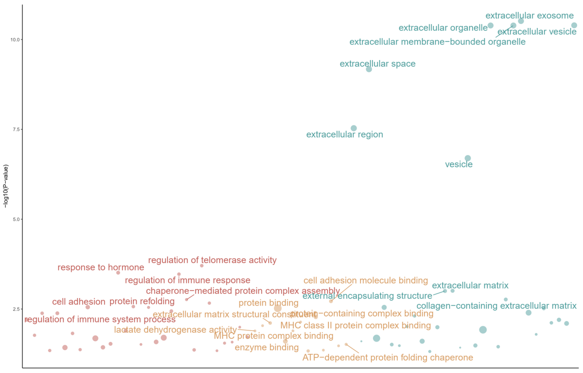

Module VIII

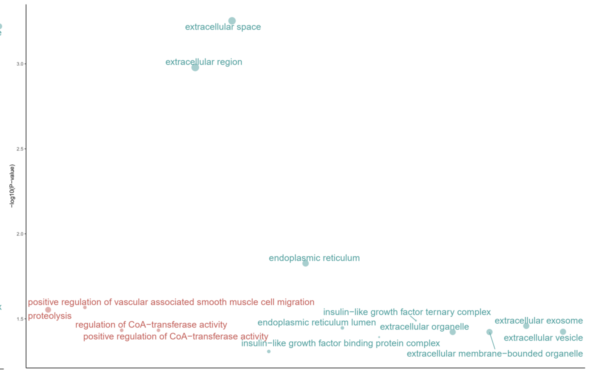

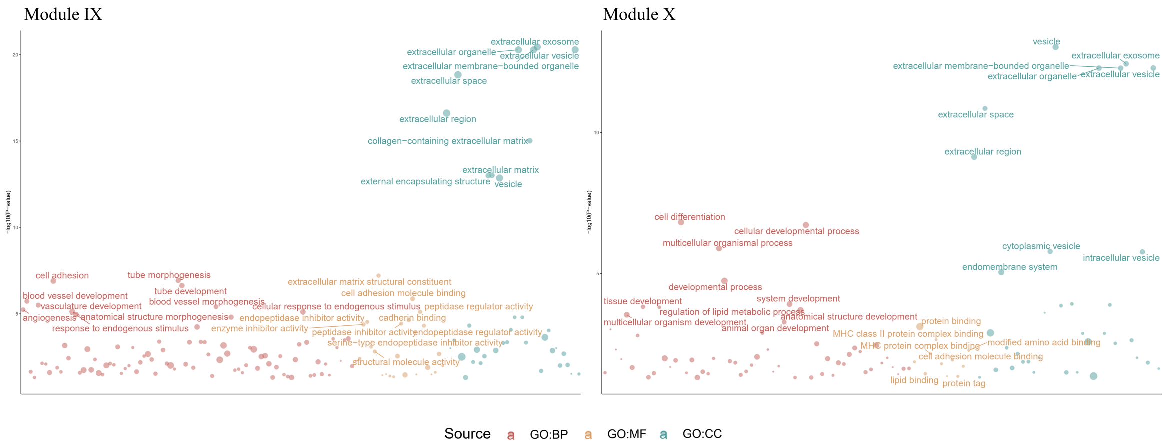

Fig. S24. GO enrichment analysis of the tissue modules identified by STModule from BC layer 2. Y-axis represents -$\log_{10}$(P-value). Color indicates the source of the GO terms. Point size indicates the number of overlapping genes of the modules and the terms. X-axis represents the id and type of the terms.

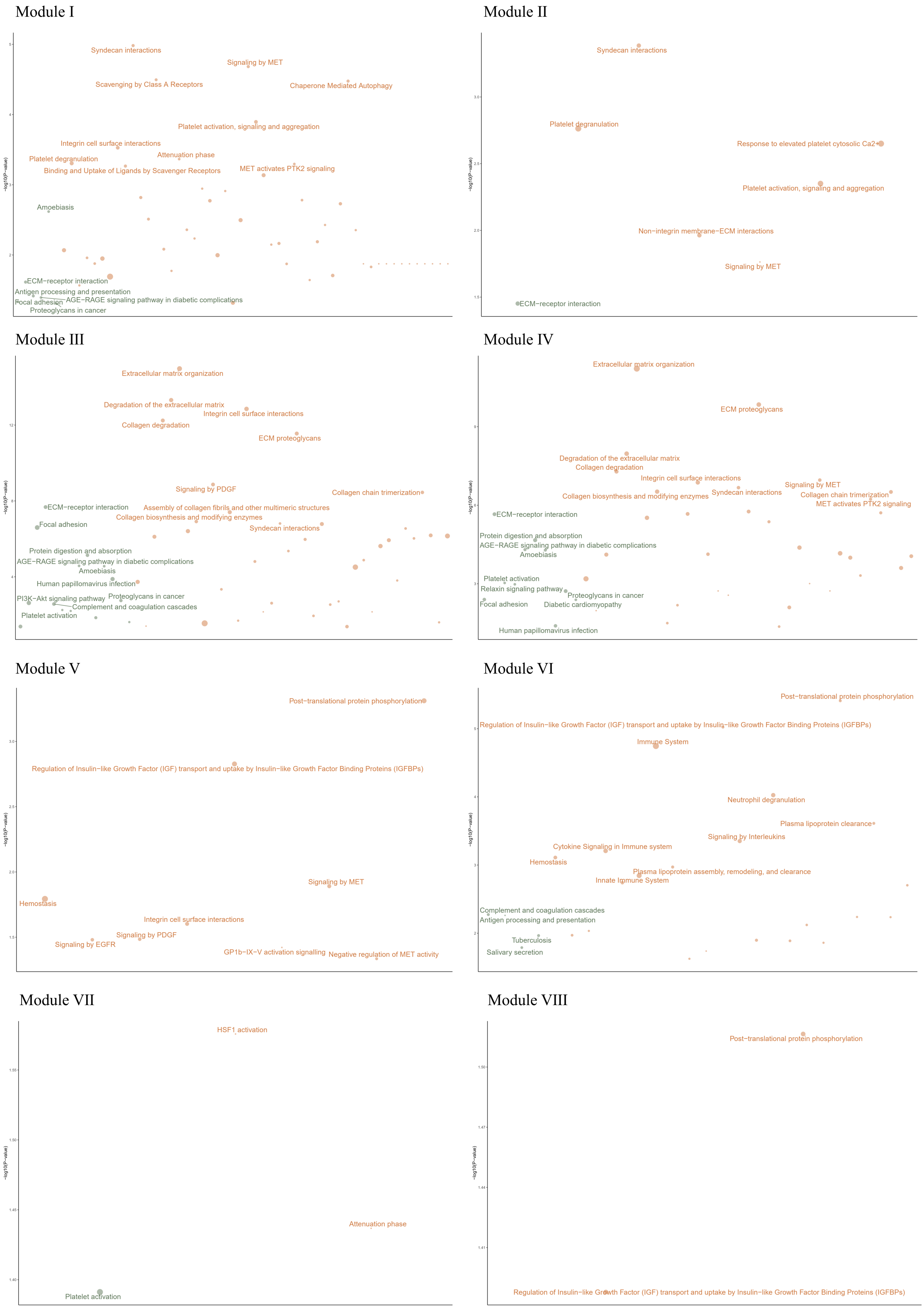
Module I
Syndecan interactions
Signaling by MET
Scavenging by Class A Receptors
Chaperone Mediated Autophagy
Platelet activation, signaling and aggregation
Integrin cell surface interactions
Attenuation phase
Platelet degranulation
Binding and Uptake of Ligands by Scavenger Receptors
MET activates PTK2 signaling
Amoebiasis
ECM−receptor interaction
Antigen processing and presentation
Focal adhesion
AGE−RAGE signaling pathway in diabetic complications
Proteoglycans in cancer
−log10(P−value)
Module II
Syndecan interactions
Platelet degranulation
Response to elevated platelet cytosolic Ca2+
Platelet activation, signaling and aggregation
Non−integrin membrane−ECM interactions
Signaling by MET
ECM−receptor interaction
−log10(P−value)
Module III
Extracellular matrix organization
Degradation of the extracellular matrix
Integrin cell surface interactions
Collagen degradation
ECM proteoglycans
Signaling by PDGF
Collagen chain trimerization
ECM−receptor interaction
Assembly of collagen fibrils and other multimeric structures
Collagen biosynthesis and modifying enzymes
Focal adhesion
Syndecan interactions
Protein digestion and absorption
AGE−RAGE signaling pathway in diabetic complications
Amoebiasis
Human papillomavirus infection
PI3K−Akt signaling pathway
Proteoglycans in cancer
Complement and coagulation cascades
Platelet activation
−log10(P−value)
Module IV
Extracellular matrix organization
ECM proteoglycans
Degradation of the extracellular matrix
Collagen degradation
Integrin cell surface interactions
Signaling by MET
Collagen biosynthesis and modifying enzymes
Syndecan interactions
Collagen chain trimerization
MET activates PTK2 signaling
ECM−receptor interaction
Protein digestion and absorption
AGE−RAGE signaling pathway in diabetic complications
Amoebiasis
Platelet activation
Relaxin signaling pathway
Proteoglycans in cancer
Focal adhesion
Dilated cardiomyopathy
Human papillomavirus infection
−log10(P−value)
Module V
Post−translational protein phosphorylation
Regulation of Insulin−like Growth Factor (IGF) transport and uptake by Insulin−like Growth Factor Binding Proteins (IGFBPs)
Signaling by MET
Hemostasis
Integrin cell surface interactions
Signaling by PDGF
Signaling by EGFR
GP1b−IX−V activation signalling
Negative regulation of MET activity
−log10(P−value)
Module VI
Post−translational protein phosphorylation
Regulation of Insulin−like Growth Factor (IGF) transport and uptake by Insulin−like Growth Factor Binding Proteins (IGFBPs)
Immune System
Neutrophil degranulation
Plasma lipoprotein clearance
Signaling by Interleukins
Cytokine Signaling in Immune system
Hemostasis
Plasma lipoprotein assembly, remodeling, and clearance
Innate Immune System
Complement and coagulation cascades
Antigen processing and presentation
Tuberculosis
Salivary secretion
−log10(P−value)
Module VII
HSF1 activation
Attenuation phase
Platelet activation
−log10(P−value)
Module VIII
Post−translational protein phosphorylation
Regulation of Insulin−like Growth Factor (IGF) transport and uptake by Insulin−like Growth Factor Binding Proteins (IGFBPs)
−log10(P−value)

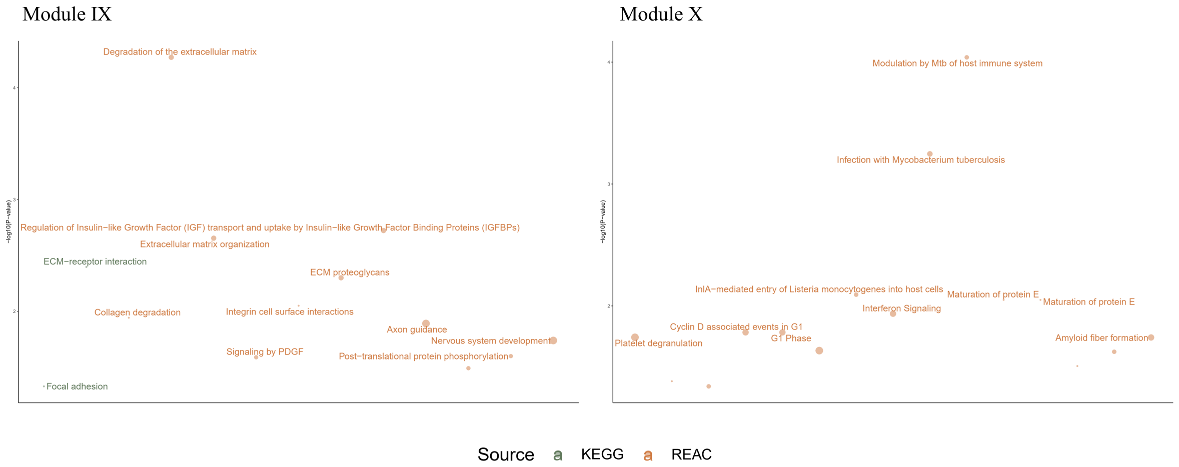

Fig. S25. Functional enrichment analysis of the tissue modules identified by STModule from BC layer 2. Y-axis represents $-\log_{10}$(P-value). Color indicates the source of the pathways. Point size indicates the number of overlapping genes of the modules and the pathways. X-axis represents the id and type of the pathways.

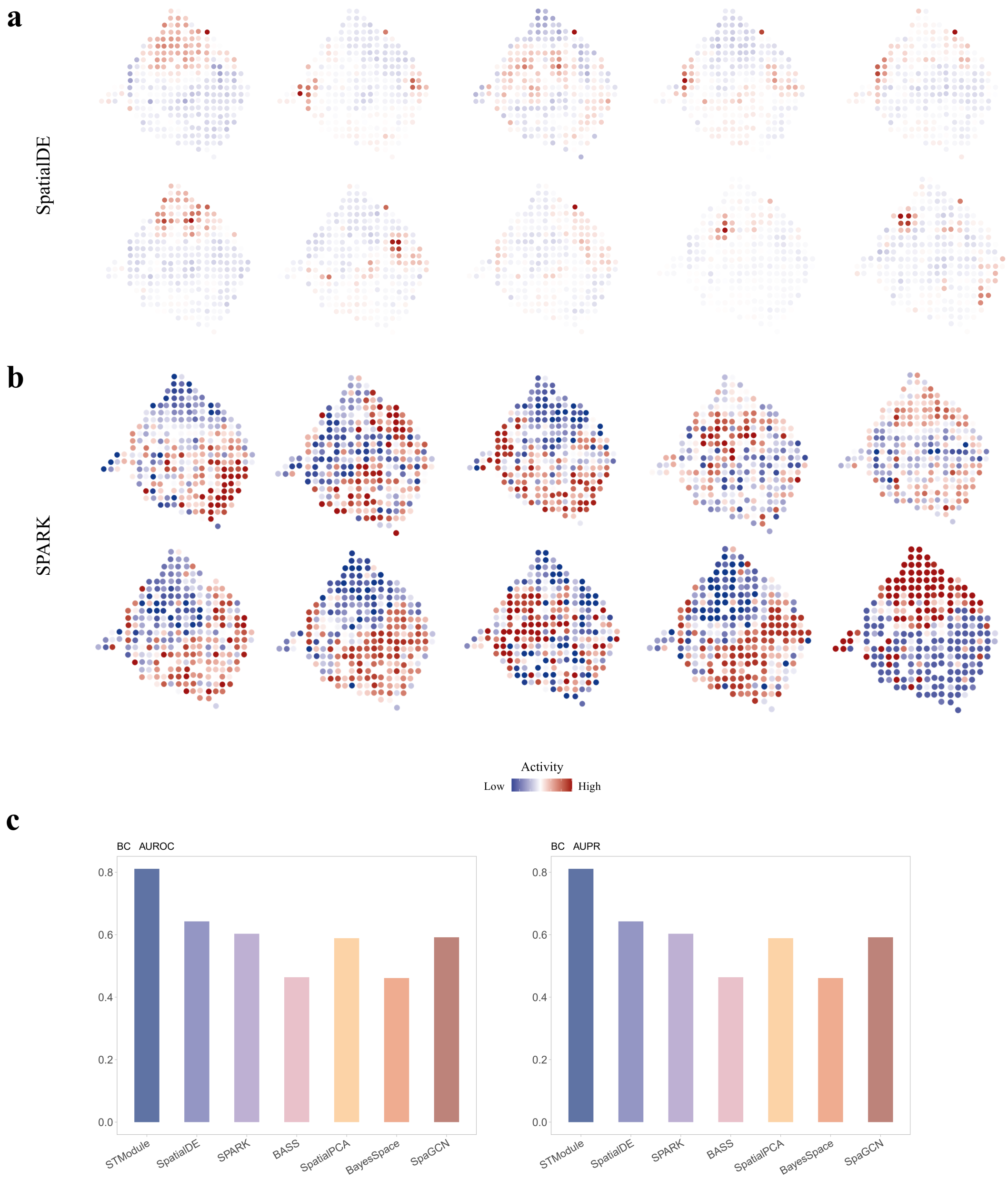

Fig. S26. Additional comparison for BC layer 2. a, b Spatial patterns identified by SpatialDE (**a**) and SPARK (**b**). **c** Comparison of AUROC and AUPR of the methods in detecting the cancer and CAF domains.

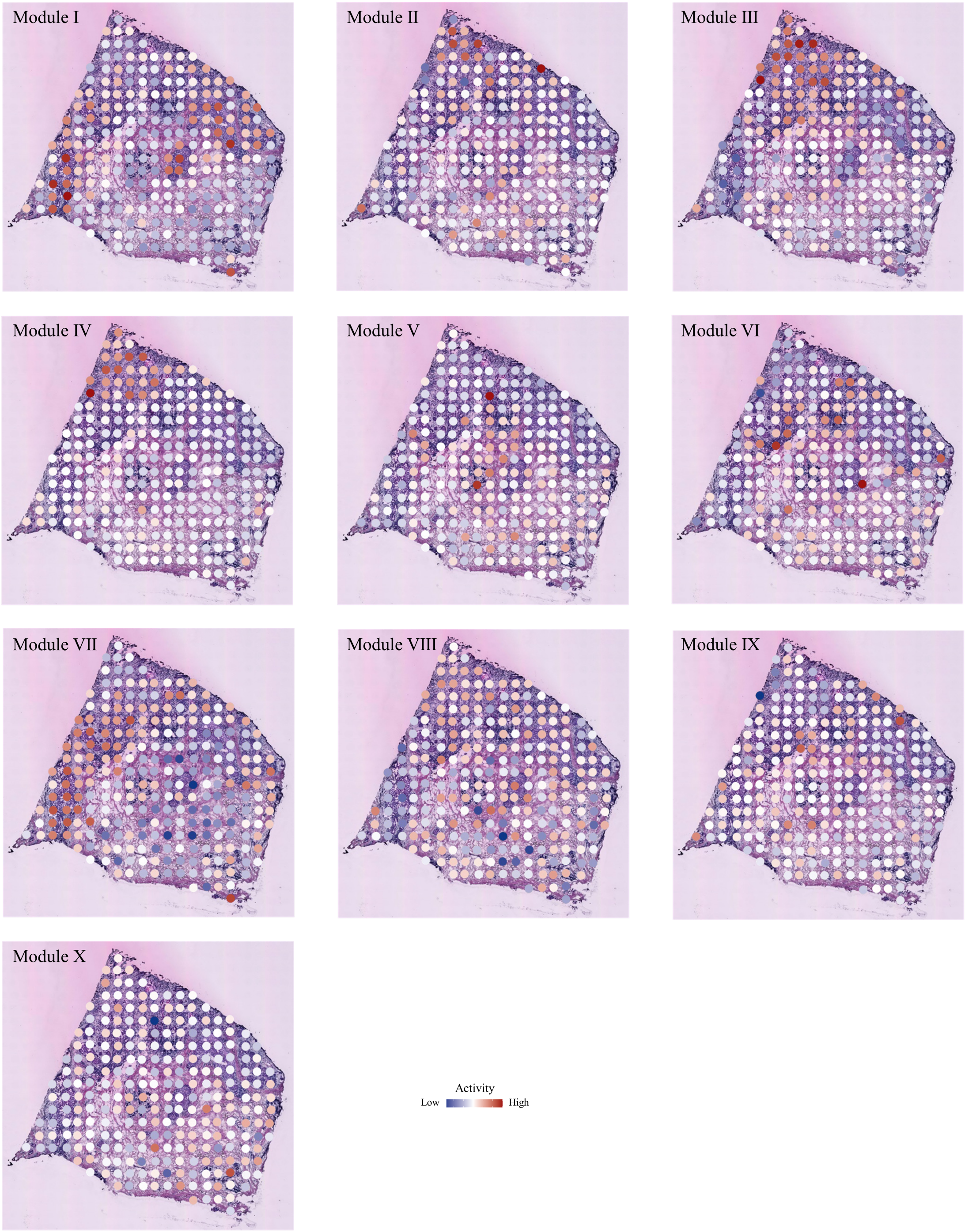

Fig. S27. Inferred spatial maps of the tissue modules for BC layer 1. The tissue modules are identified by STModule from BC layer 2. Spatial maps are manually aligned with the H&E-stained image.

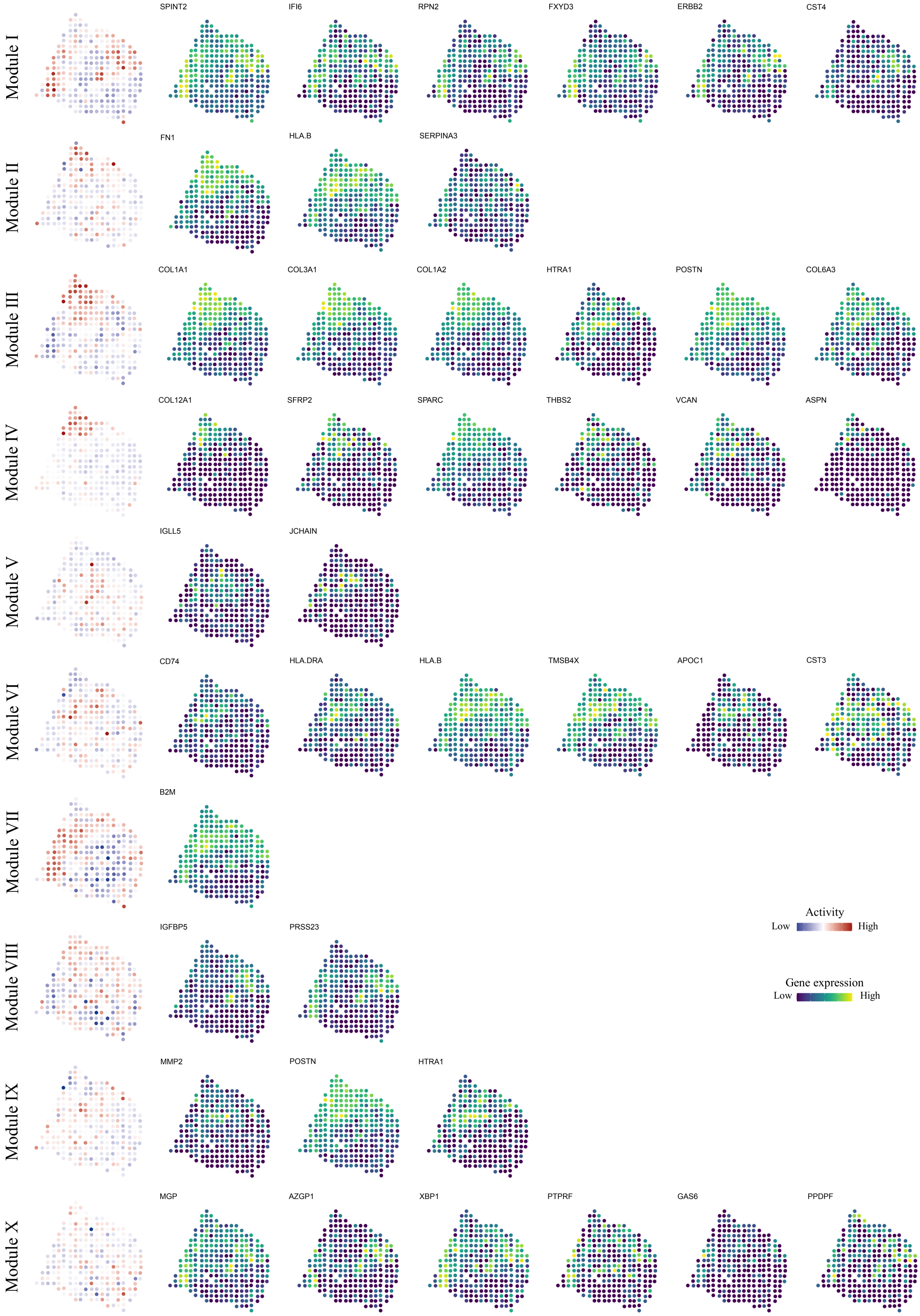

Fig. S28. Spatial expression of representative associated genes of the tissue modules in BC layer 1.

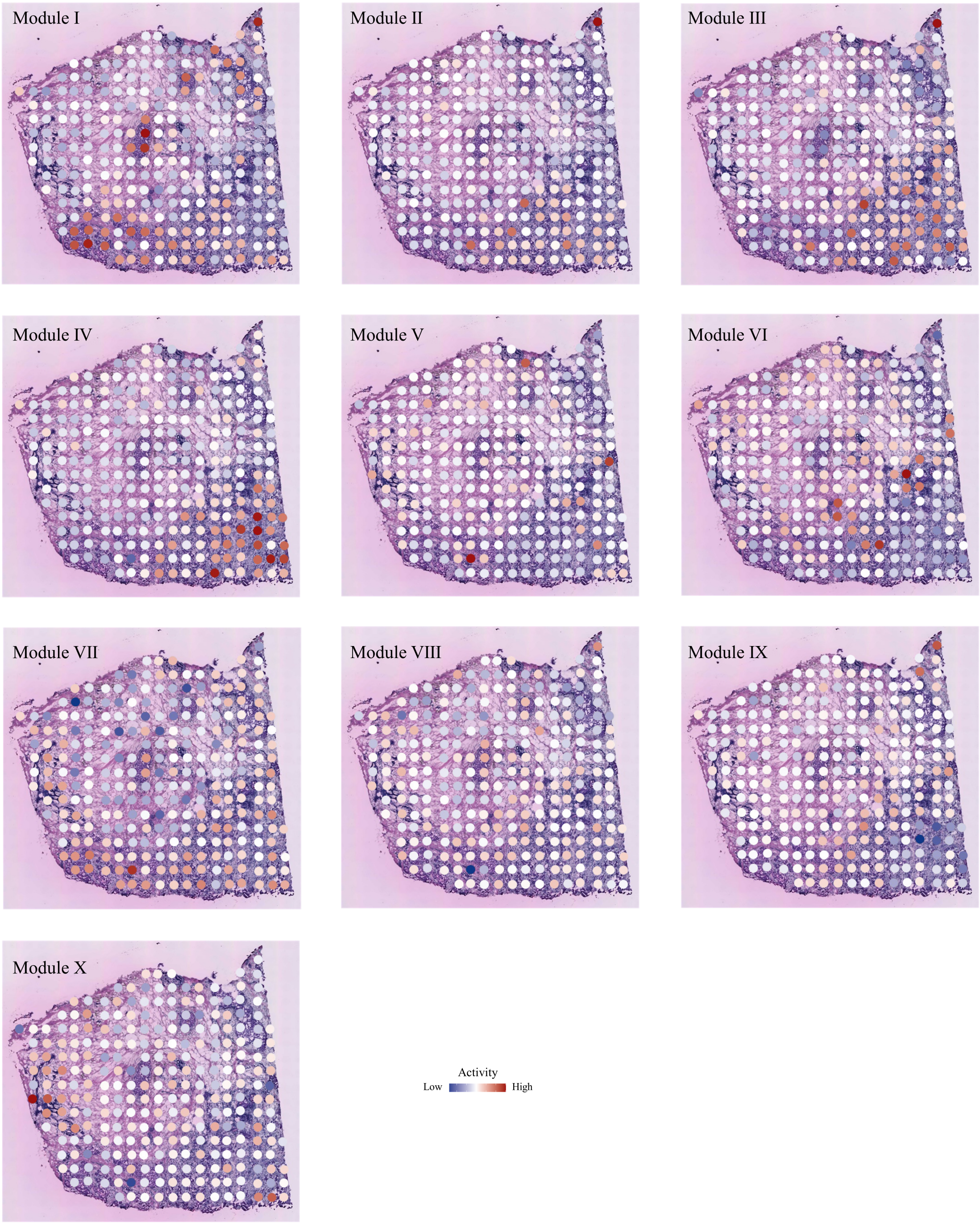

Fig. S29. Inferred spatial maps of the tissue modules for BC layer 3. The tissue modules are identified by STModule from BC layer 2. Spatial maps are manually aligned with the H&E-stained image.

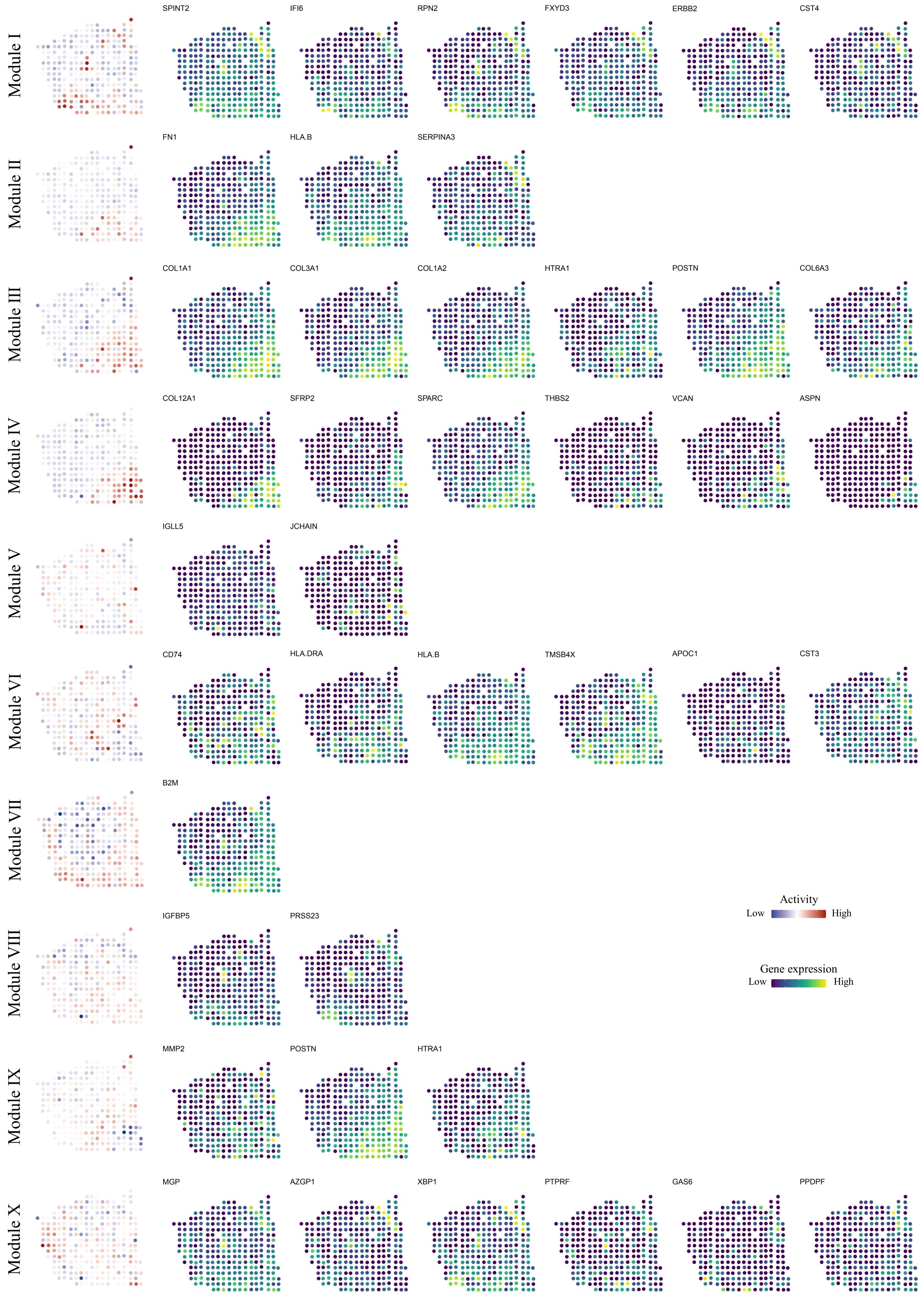

Fig. S30. Spatial expression of representative associated genes of the tissue modules in BC layer 3.

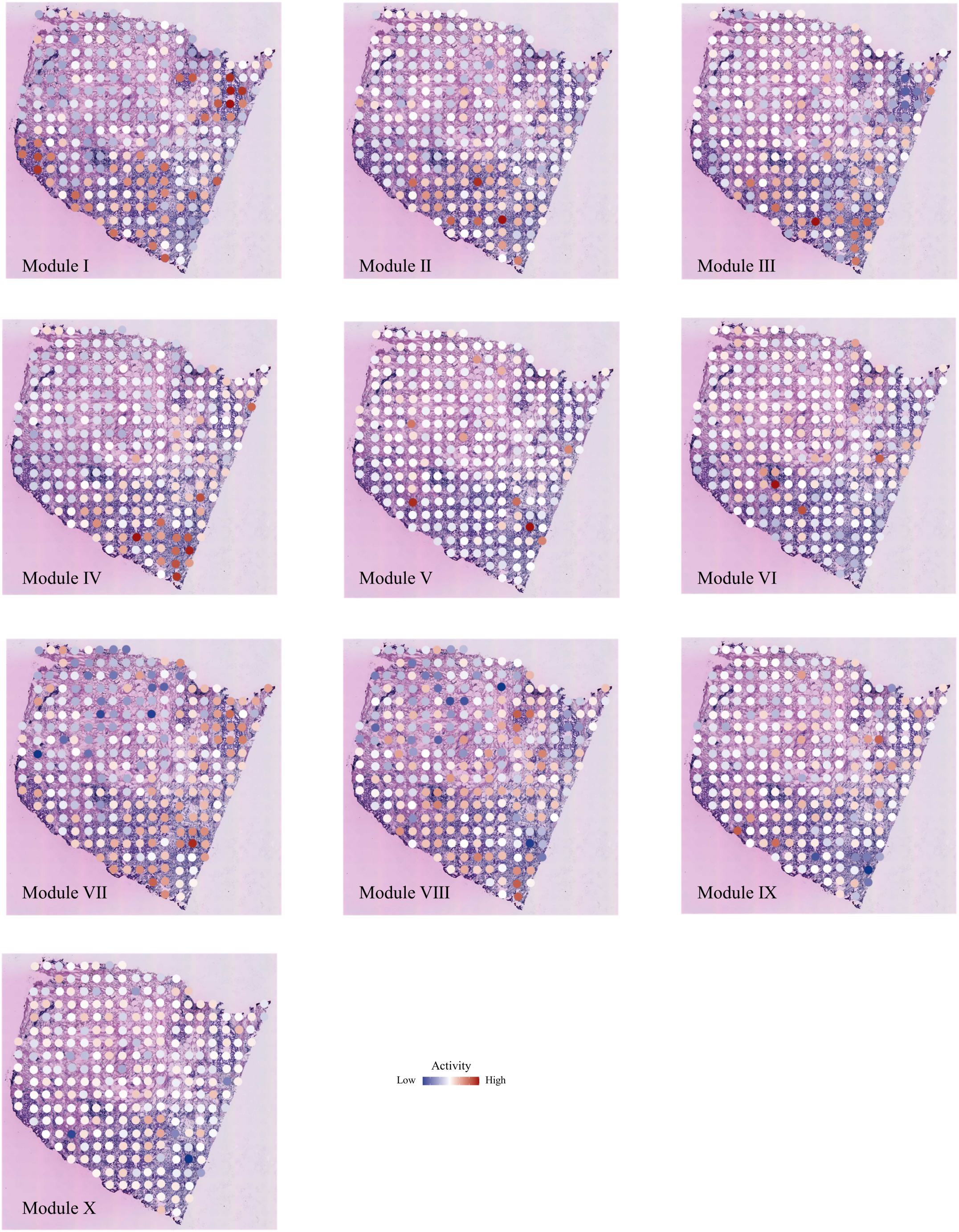

Fig. S31. Inferred spatial maps of the tissue modules for BC layer 4. The tissue modules are identified by STModule from BC layer 2. Spatial maps are manually aligned with the H&E-stained image.

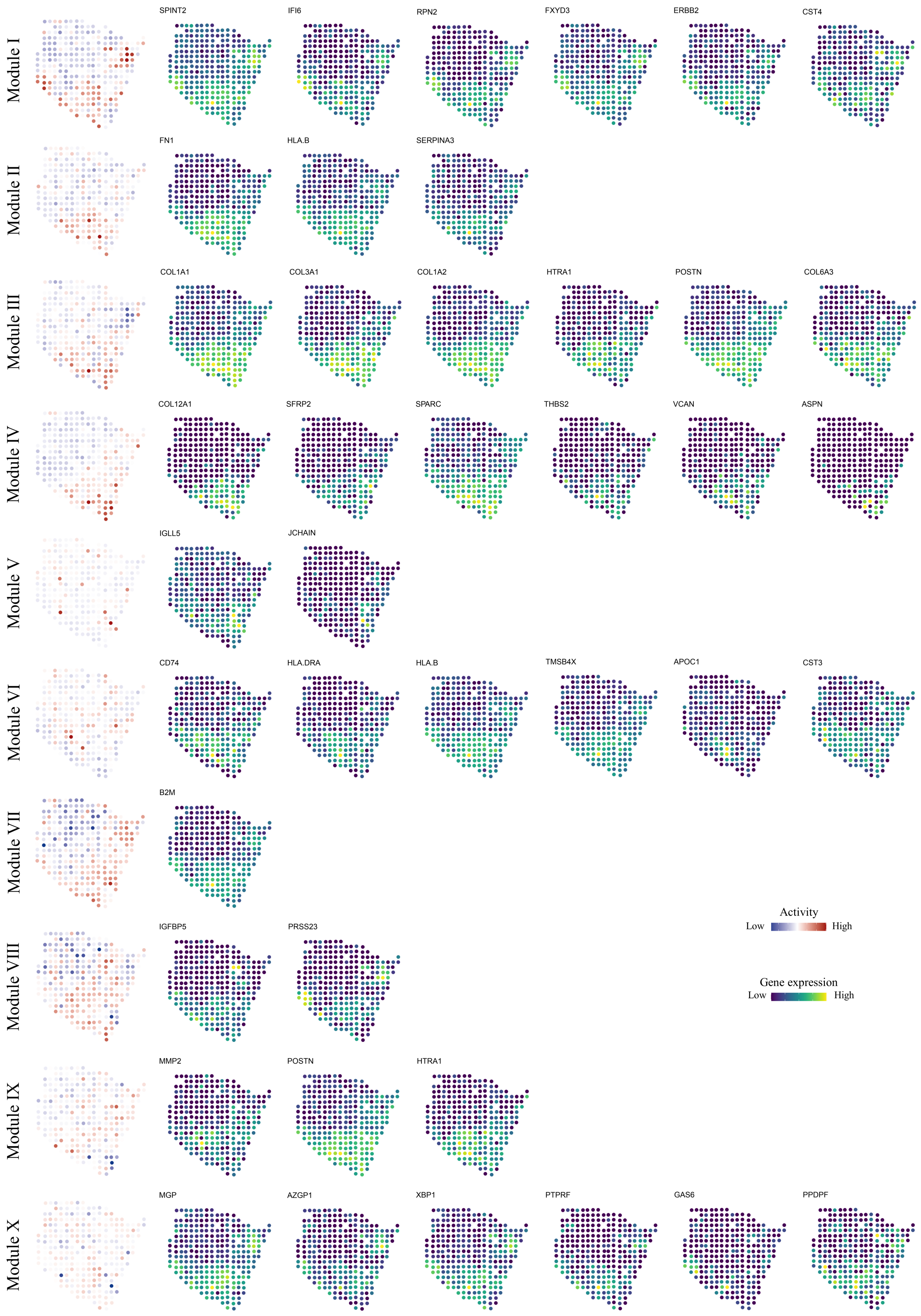

Fig. S32. Spatial expression of representative associated genes of the tissue modules in BC layer 4.

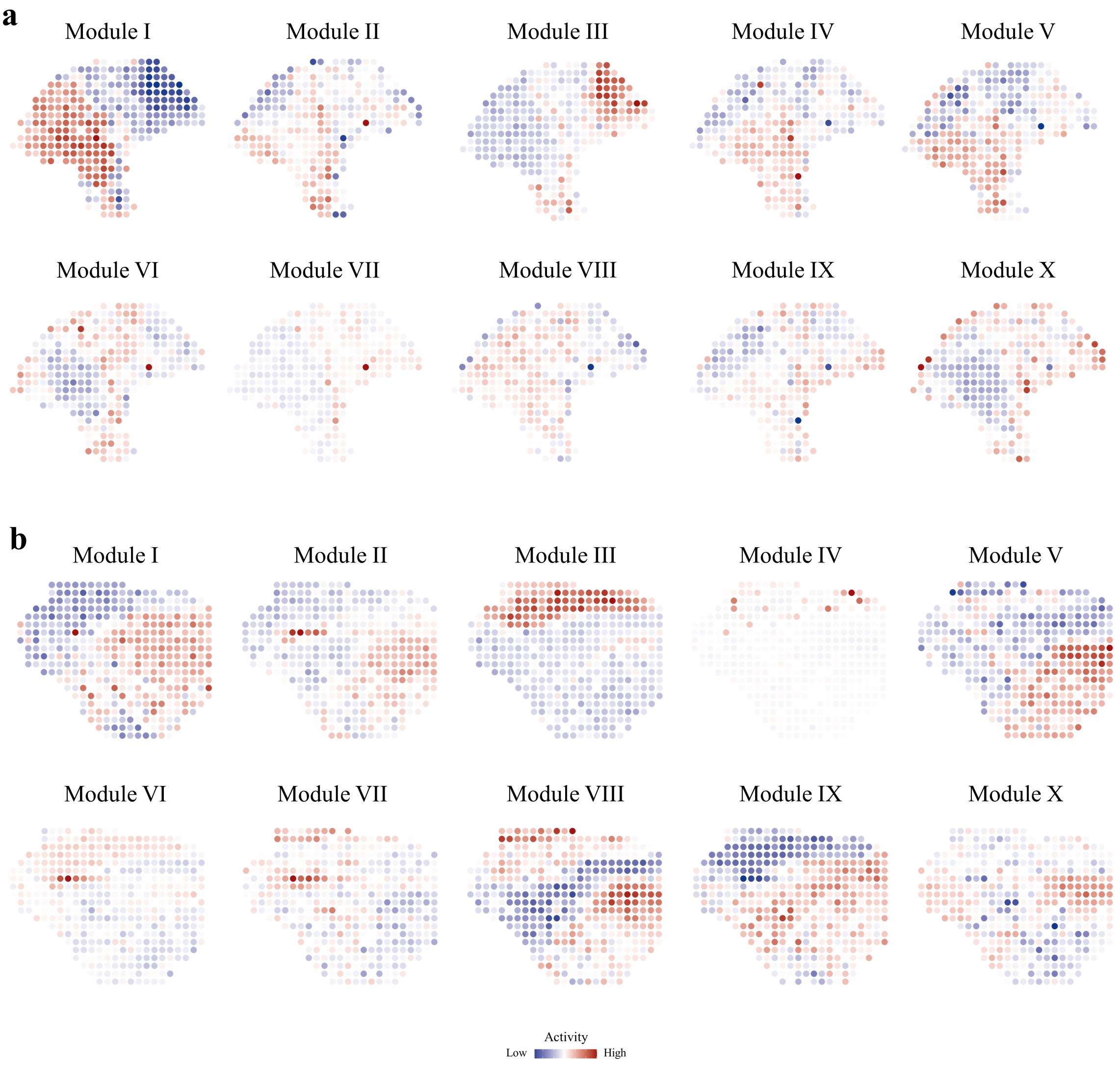

Fig. S33. Spatial maps of the tissue modules identified by STModule from the melanoma dataset. **a** Spatial maps for sample 1. Annotations of the tissue modules: cancer cells (I), transition area (II), lymphoid tissue (III), B cells (IV), expression of B2M (V), macrophages (VI), CAFs (VII), melanocytes (VIII), smooth muscle (IX), expression of CH507-513H4.5 (X). **b** Spatial maps for sample 2. Annotations of the tissue modules: cancer cells (I), transition area (II), lymphoid tissue (III), B cells (IV), expression of B2M (V), macrophages (VI), CAFs-1 (VII), CAFs-2 (VIII), melanocytes (IX), interferon-induced (X).

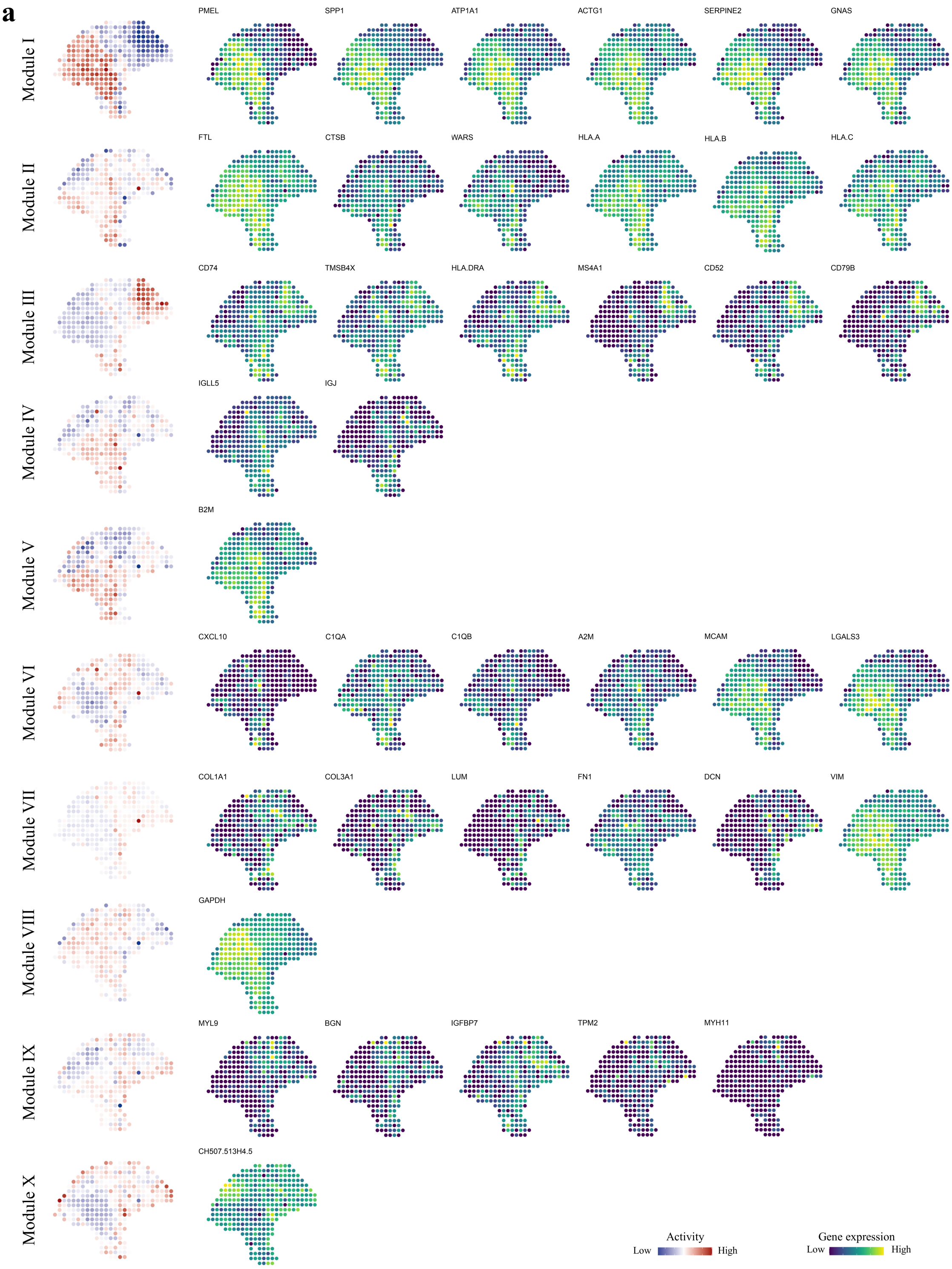
a
Module I
PMEL
SPP1
ATP1A1
ACTG1
SERPINE2
GNAS
Module II
FTL
CTSB
WARS
HLA.A
HLA.B
HLA.C
Module III
CD74
TMSB4X
HLA.DRA
MS4A1
CD52
CD79B
Module IV
IGLL5
IGJ
Module V
B2M
Module VI
CXCL10
C1QA
C1QB
A2M
MCAM
LGALS3
Module VII
COL1A1
COL3A1
LUM
FN1
DCN
VIM
Module VIII
GAPDH
Module IX
MYL9
BGN
IGFBP7
TPM2
MYH11
Module X
CH507.513H4.5
Activity
Low
High
Gene expression
Low
High

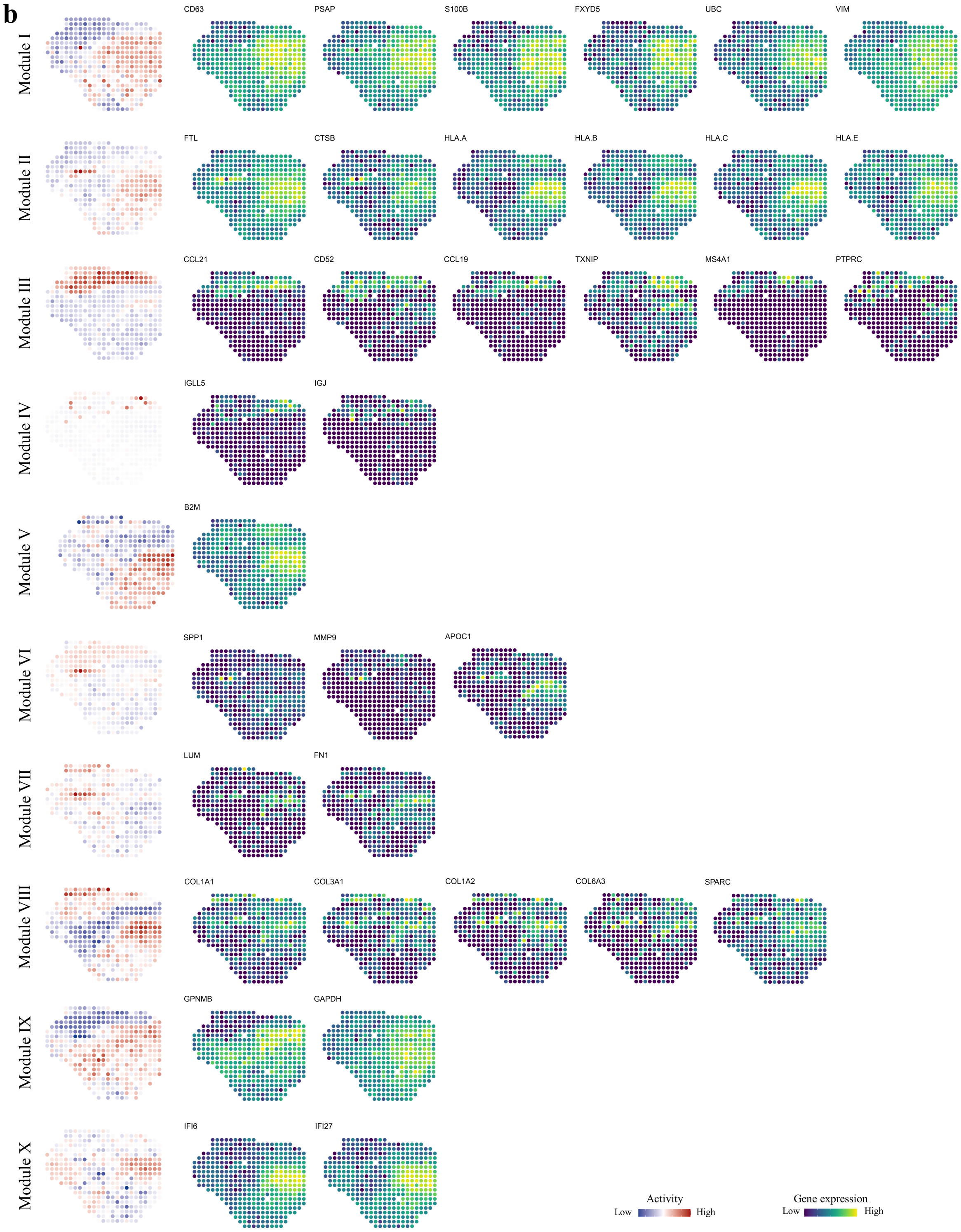

Fig. S34. Spatial expression of representative associated genes of the tissue modules identified by STModule from the melanoma dataset. a Results for sample 1. **b** Results for sample 2.

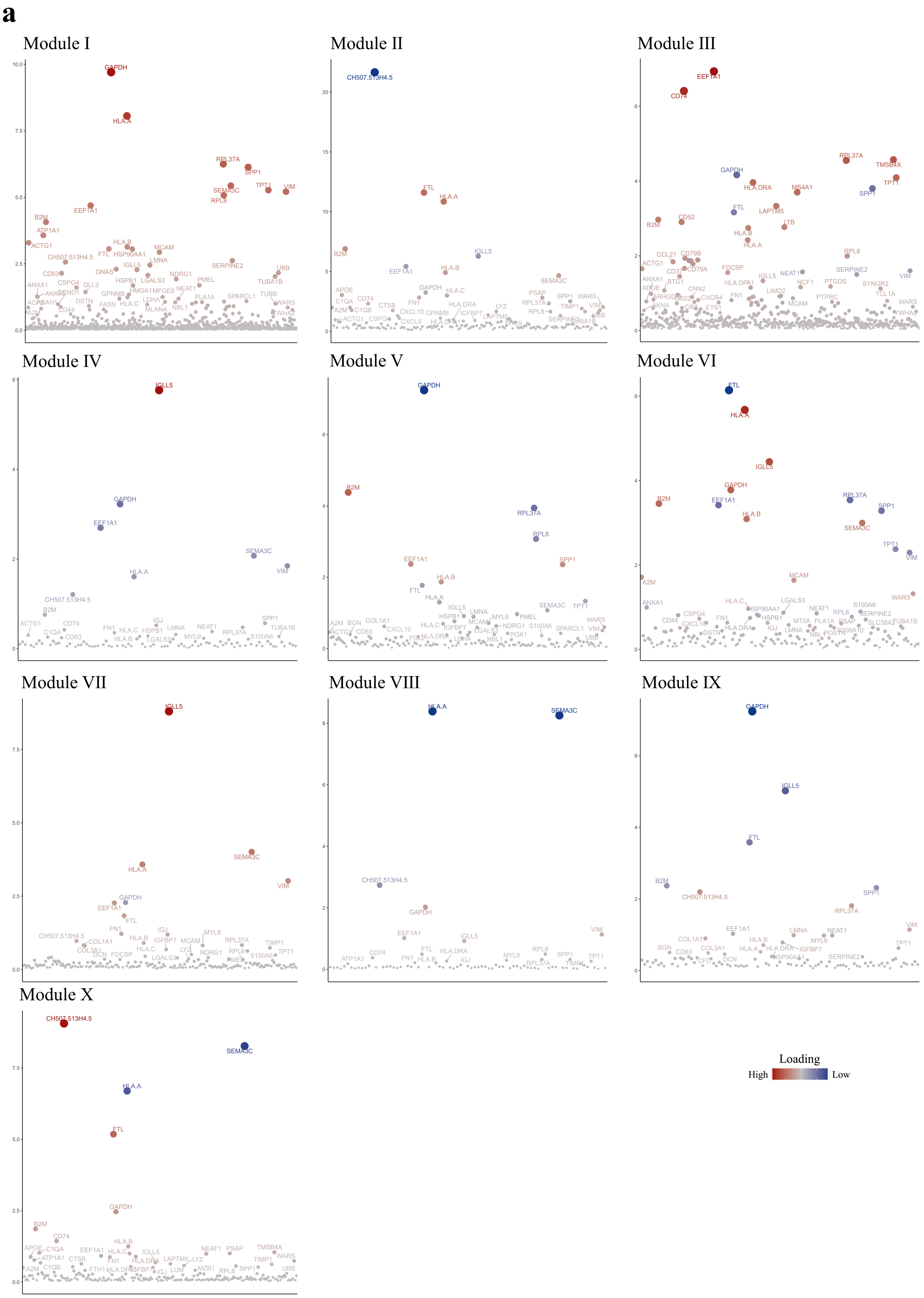
a
Module I
Module II
Module III
Module IV
Module V
Module VI
Module VII
Module VIII
Module IX
Module X
Loading
High
Low

b

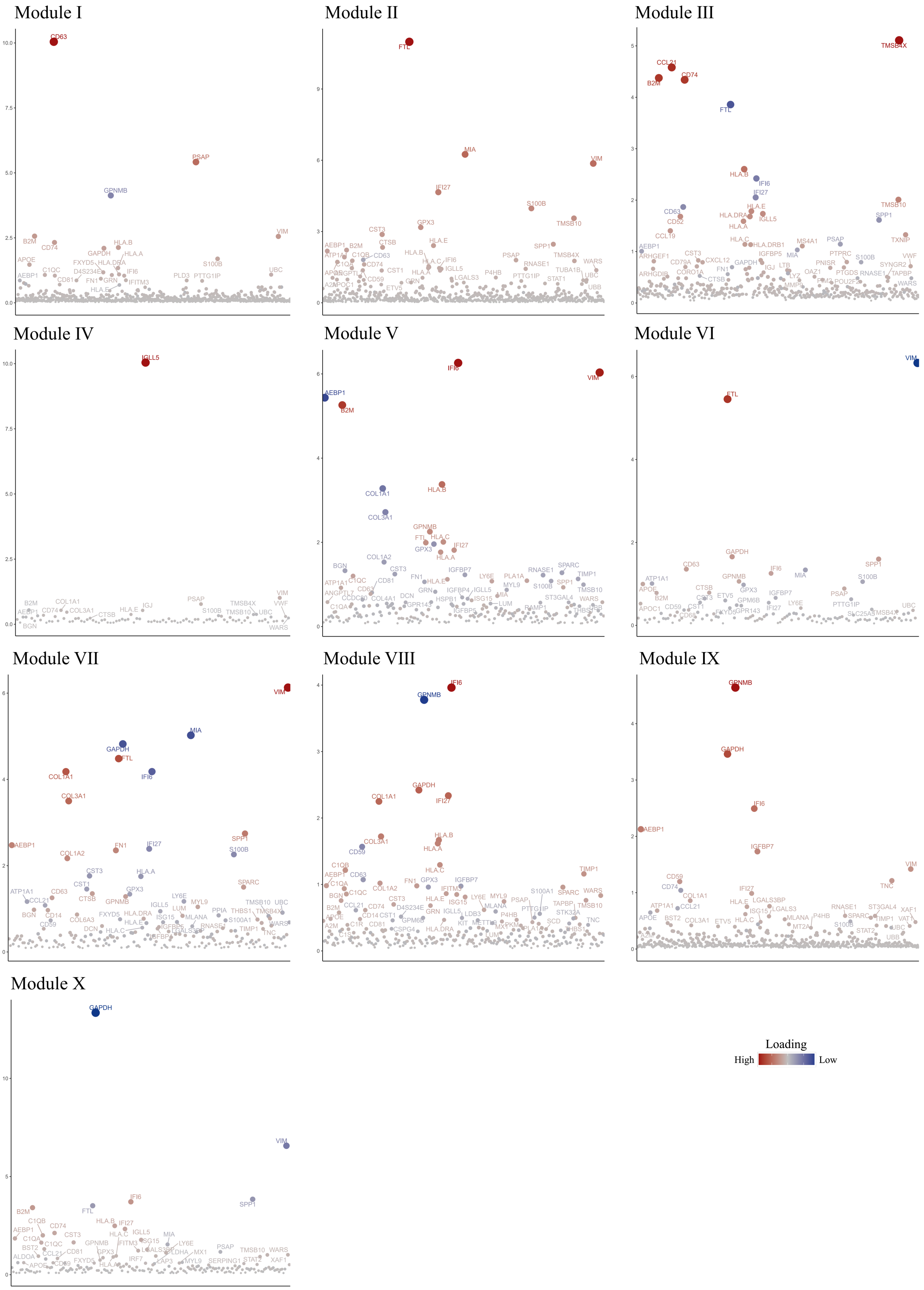

Fig. S35. Gene loadings of the tissue modules identified by STModule from the melanoma dataset. **a** Results for sample 1. **b** Results for sample 2. Y-axis represents the absolute value of gene loading and color indicates the sign and value of loading. X-axis represents the alphabetical order of the gene names.

Module I

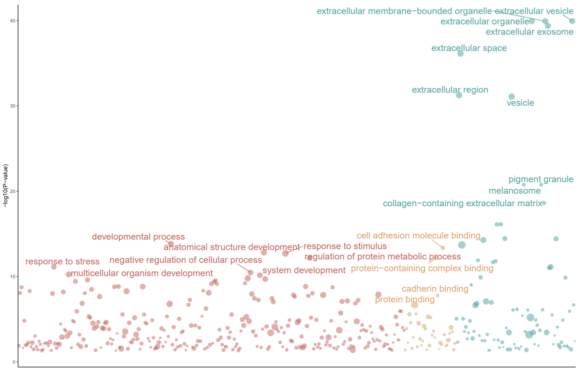

Module II

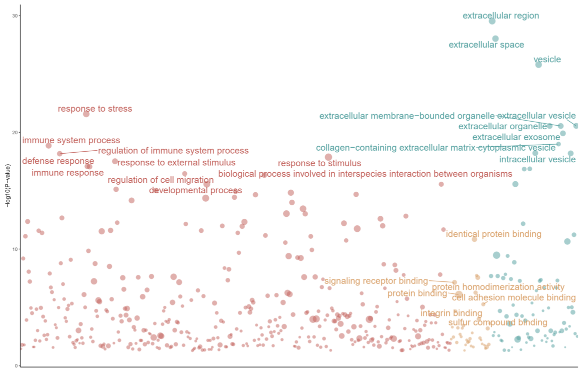

Module III

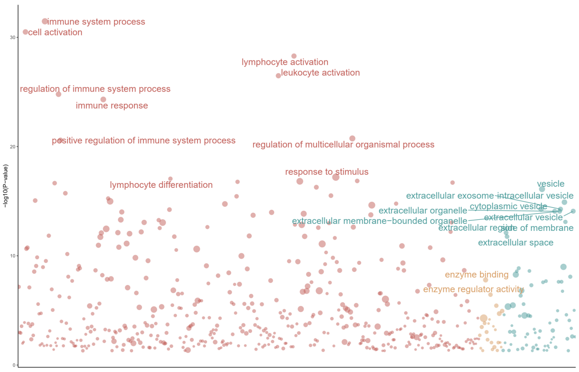

Module IV

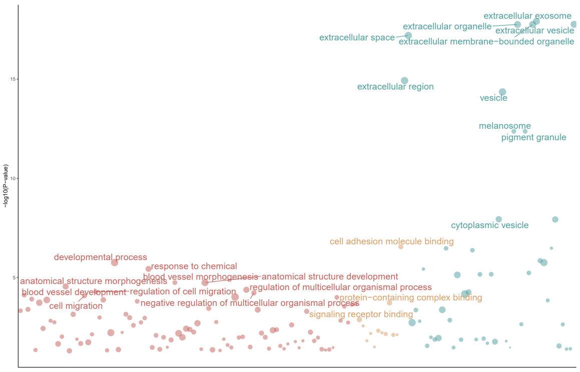

Module V

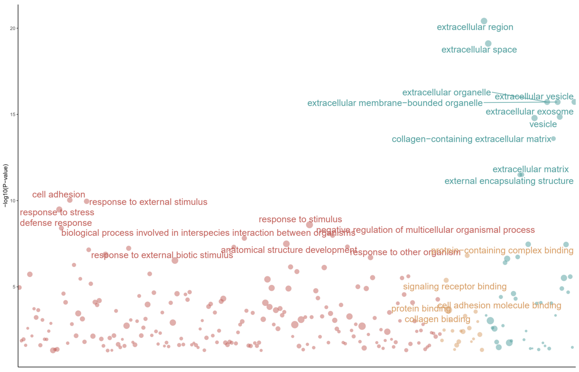

Module VI

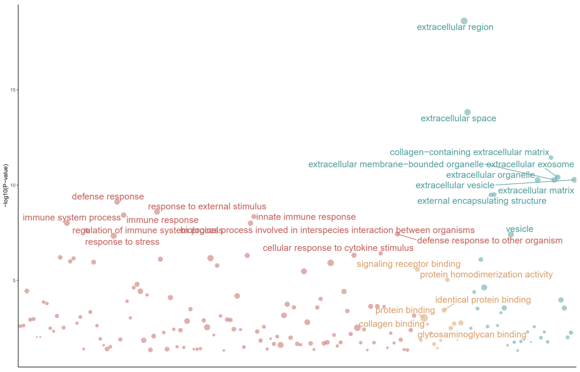

Module VII

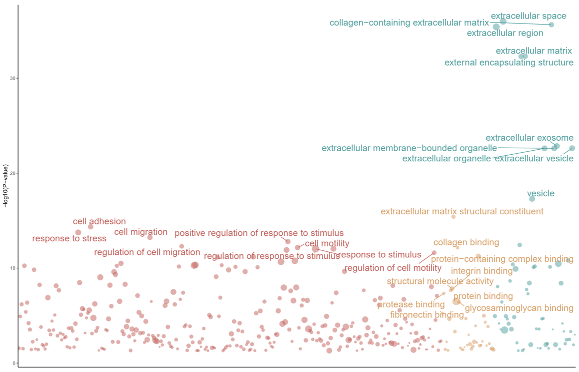

Module VIII

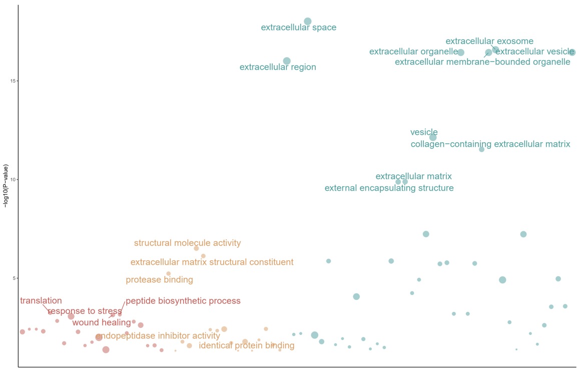

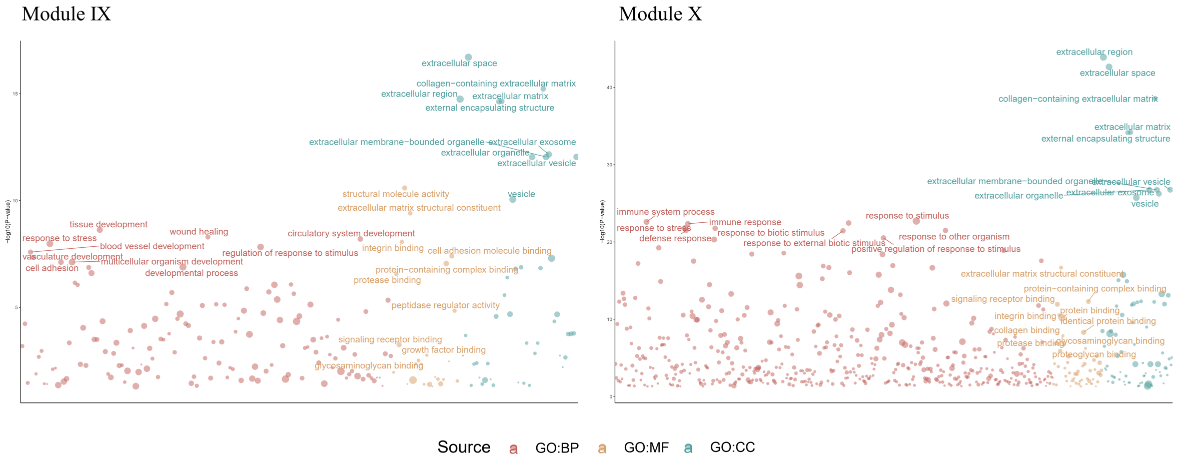

Fig. S36. GO enrichment analysis of the tissue modules identified by STModule from melanoma sample 1. Y-axis represents -$\log_{10}$(P-value). Color indicates the source of the GO terms. Point size indicates the number of overlapping genes of the modules and the terms. X-axis represents the id and type of the terms.

Module I

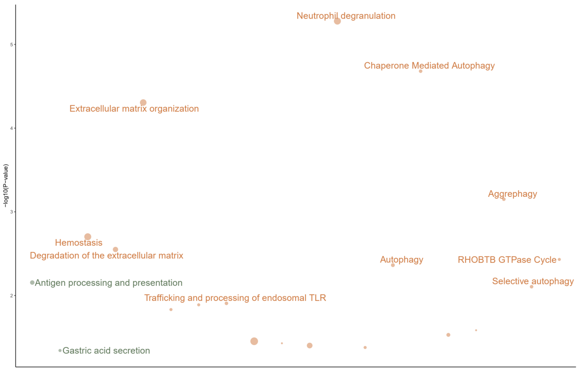

Module II

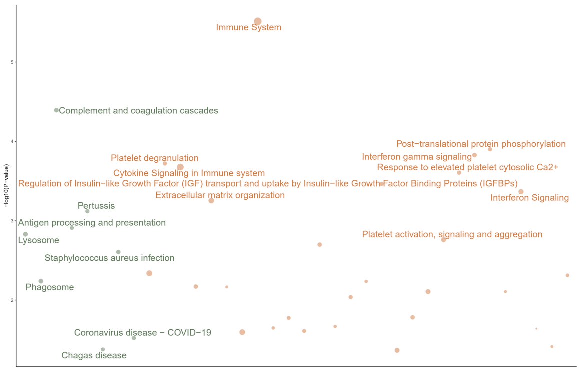

Module III

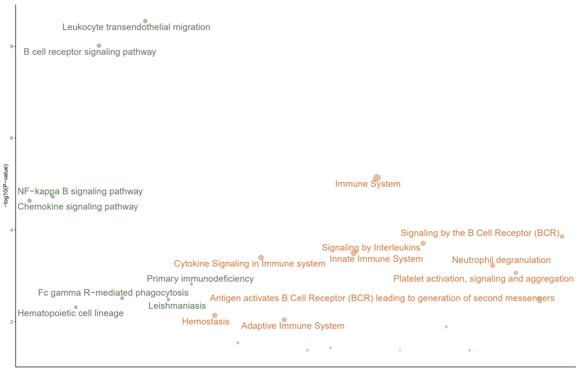

Module IV

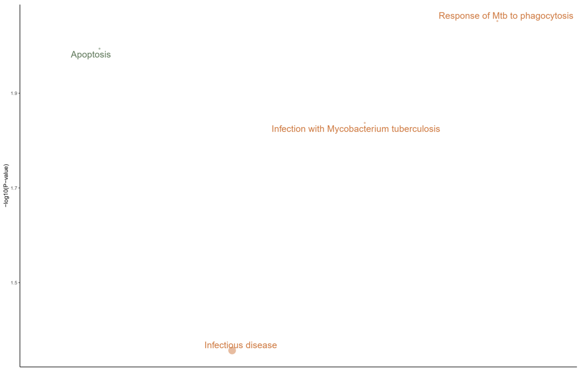

Module V

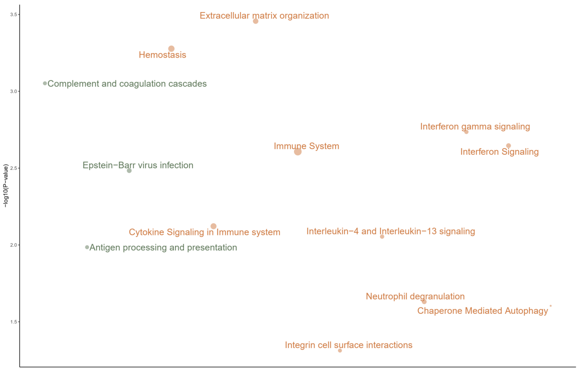

Module VI

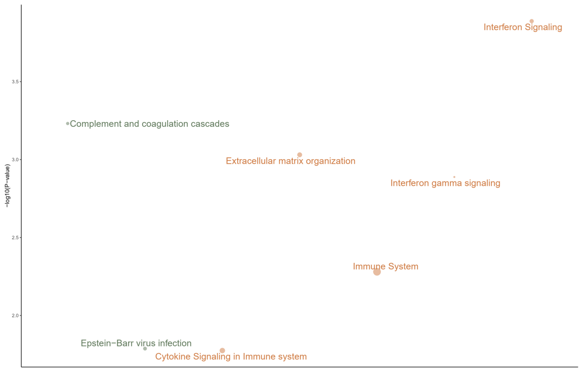

Module VII

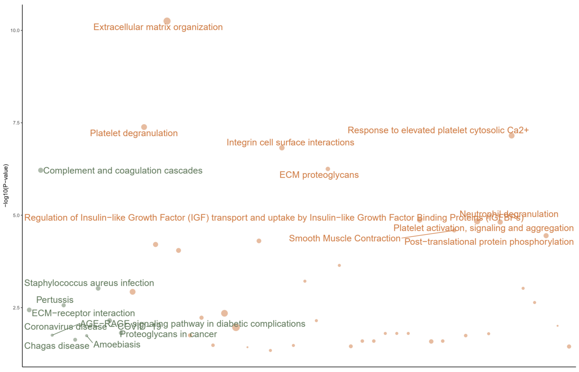

Module VIII

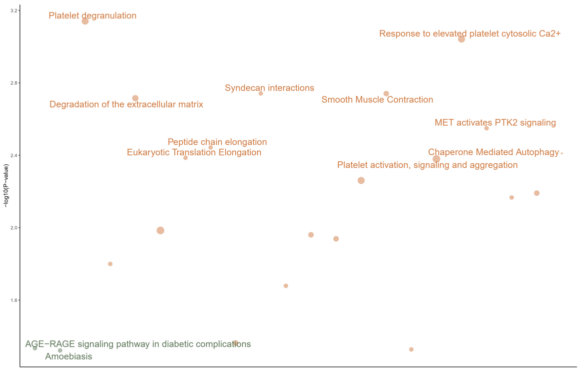

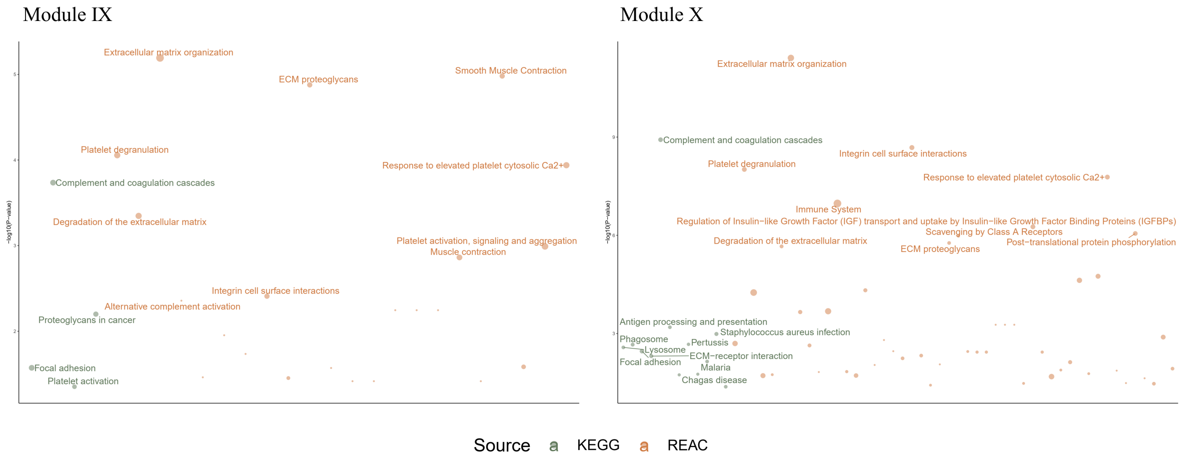

Fig. S37. Functional enrichment analysis of the tissue modules identified by STModule from melanoma sample 1. Y-axis represents -$\log_{10}$(P-value). Color indicates the source of the pathways. Point size indicates the number of overlapping genes of the modules and the pathways. X-axis represents the id and type of the pathways.

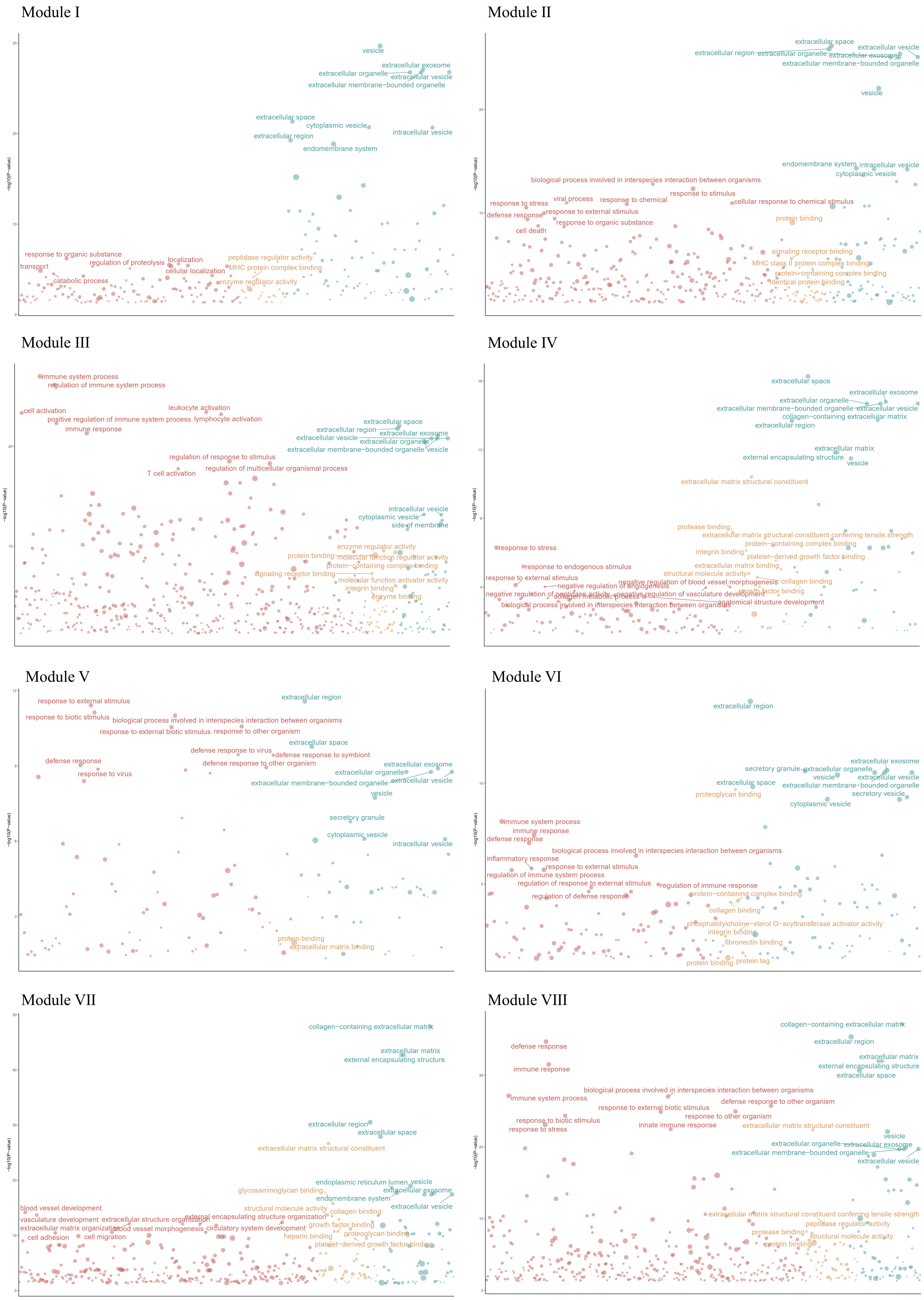
Module I
vesicle
extracellular exosome
extracellular organelle
extracellular vesicle
extracellular membrane−bounded organelle
extracellular space
cytoplasmic vesicle
extracellular region
intracellular vesicle
endomembrane system
response to organic substance
transport
regulation of proteolysis
localization
cellular localization
catabolic process
peptidase regulator activity
MHC protein complex binding
enzyme regulator activity
-log10(P−value)
Module II
extracellular space
extracellular vesicle
extracellular region
extracellular organelle
extracellular exosome
extracellular membrane−bounded organelle
vesicle
endomembrane system
intracellular vesicle
cytoplasmic vesicle
biological process involved in interspecies interaction between organisms
response to stress
viral process
response to chemical
response to stimulus
cellular response to chemical stimulus
defense response
response to external stimulus
response to organic substance
cell death
protein binding
signaling receptor binding
MHC class II protein complex binding
protein−containing complex binding
identical protein binding
Module III
immune system process
regulation of immune system process
cell activation
leukocyte activation
positive regulation of immune system process
lymphocyte activation
immune response
extracellular space
extracellular region
extracellular exosome
extracellular vesicle
extracellular organelle
extracellular membrane−bounded organelle
vesicle
regulation of response to stimulus
regulation of multicellular organismal process
T cell activation
intracellular vesicle
cytoplasmic vesicle
side of membrane
enzyme regulator activity
protein binding
molecular function regulator activity
protein−containing complex binding
signaling receptor binding
molecular function activator activity
integrin binding
enzyme binding
Module IV
extracellular space
extracellular exosome
extracellular organelle
extracellular membrane−bounded organelle
extracellular vesicle
collagen−containing extracellular matrix
extracellular region
extracellular matrix
external encapsulating structure
vesicle
extracellular matrix structural constituent
protease binding
extracellular matrix structural constituent conferring tensile strength
response to stress
protein−containing complex binding
integrin binding
platelet−derived growth factor binding
response to endogenous stimulus
extracellular matrix binding
structural molecule activity
response to external stimulus
negative regulation of blood vessel morphogenesis
collagen binding
negative regulation of angiogenesis
growth factor binding
negative regulation of peptidase activity
collagen metabolic process
negative regulation of vasculature development
anatomical structure development
biological process involved in interspecies interaction between organisms
Module V
extracellular region
response to external stimulus
response to biotic stimulus
biological process involved in interspecies interaction between organisms
response to external biotic stimulus
response to other organism
extracellular space
defense response to virus
defense response to symbiont
defense response
defense response to other organism
response to virus
extracellular exosome
extracellular organelle
extracellular membrane−bounded organelle
extracellular vesicle
vesicle
secretory granule
cytoplasmic vesicle
intracellular vesicle
protein binding
extracellular matrix binding
Module VI
extracellular region
extracellular exosome
secretory granule
extracellular organelle
vesicle
extracellular vesicle
extracellular space
extracellular membrane−bounded organelle
proteoglycan binding
secretory vesicle
cytoplasmic vesicle
immune system process
immune response
defense response
biological process involved in interspecies interaction between organisms
inflammatory response
response to external stimulus
regulation of immune system process
regulation of response to external stimulus
regulation of immune response
regulation of defense response
protein−containing complex binding
collagen binding
phosphatidylcholine−sterol O−acyltransferase activator activity
integrin binding
fibronectin binding
protein binding
protein tag
Module VII
collagen−containing extracellular matrix
extracellular matrix
external encapsulating structure
extracellular region
extracellular space
extracellular matrix structural constituent
endoplasmic reticulum lumen
vesicle
glycosaminoglycan binding
extracellular exosome
endomembrane system
structural molecule activity
extracellular vesicle
blood vessel development
collagen binding
vasculature development
extracellular structure organization
external encapsulating structure organization
growth factor binding
extracellular matrix organization
blood vessel morphogenesis
circulatory system development
cell adhesion
cell migration
heparin binding
proteoglycan binding
platelet−derived growth factor binding
Module VIII
collagen−containing extracellular matrix
defense response
extracellular region
extracellular matrix
immune response
external encapsulating structure
extracellular space
biological process involved in interspecies interaction between organisms
immune system process
defense response to other organism
response to external biotic stimulus
response to other organism
response to biotic stimulus
innate immune response
extracellular matrix structural constituent
response to stress
vesicle
extracellular organelle
extracellular exosome
extracellular membrane−bounded organelle
extracellular vesicle
extracellular matrix structural constituent conferring tensile strength
peptidase regulator activity
protease binding
structural molecule activity
protein binding

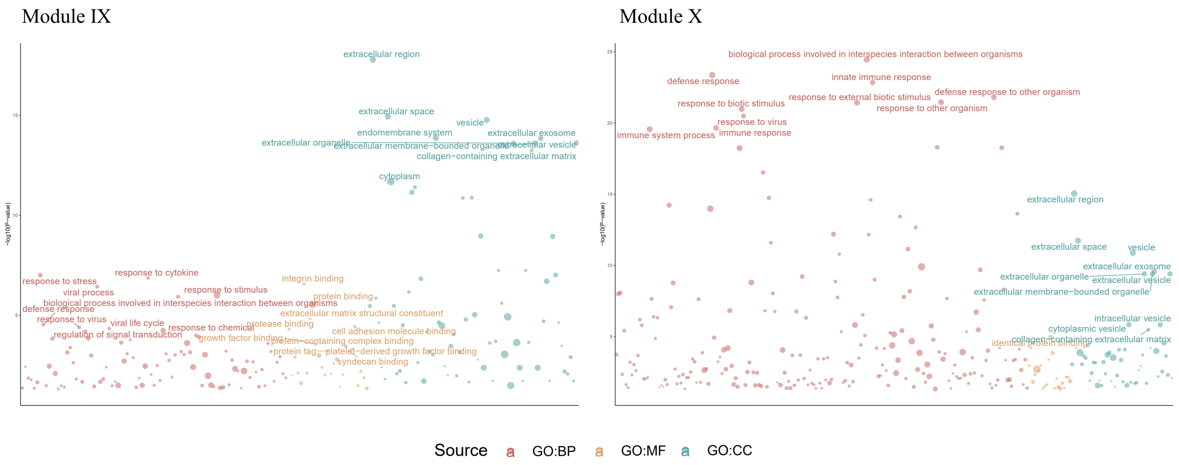

Fig. S38. GO enrichment analysis of the tissue modules identified by STModule from melanoma sample 2. Y-axis represents -log$_{10}$(P-value). Color indicates the source of the GO terms. Point size indicates the number of overlapping genes of the modules and the terms. X-axis represents the id and type of the terms.

Module I

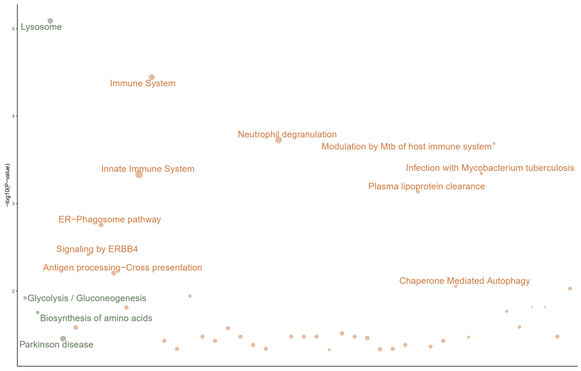

Module II

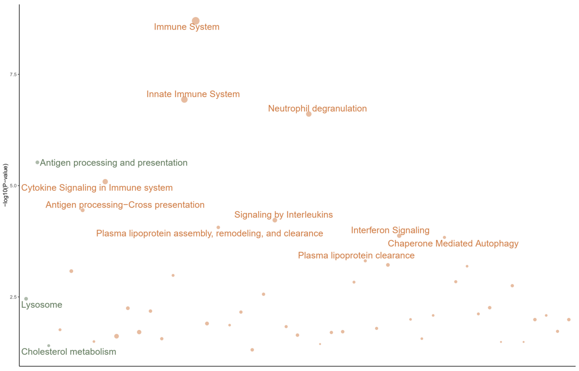

Module III

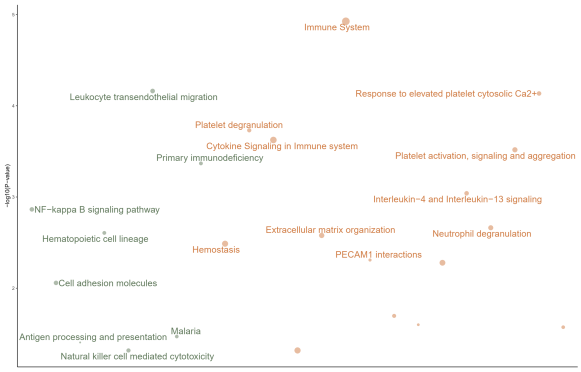

Module IV

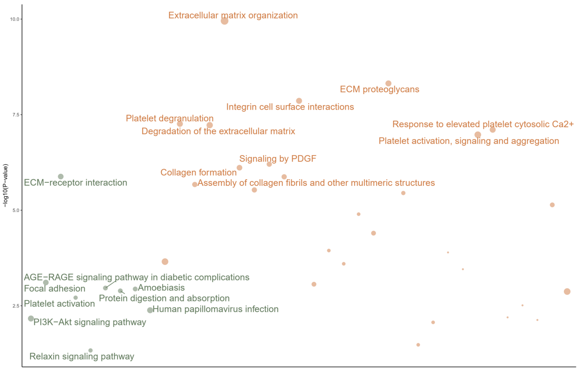

Module V

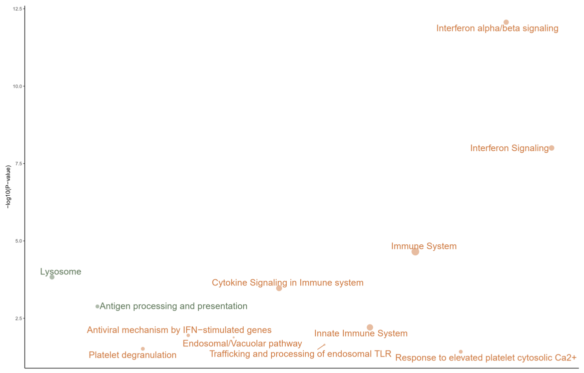

Module VI

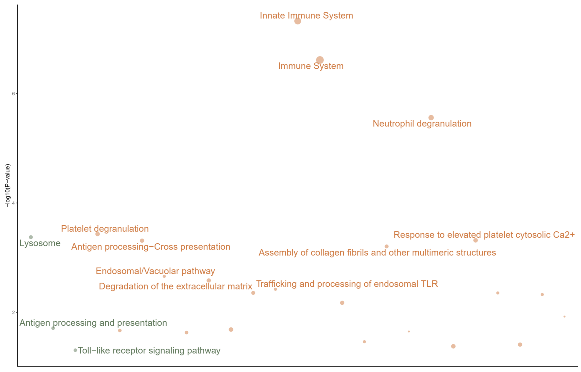

Module VII

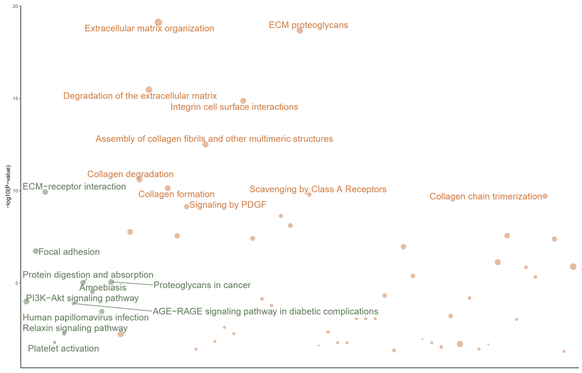

Module VIII

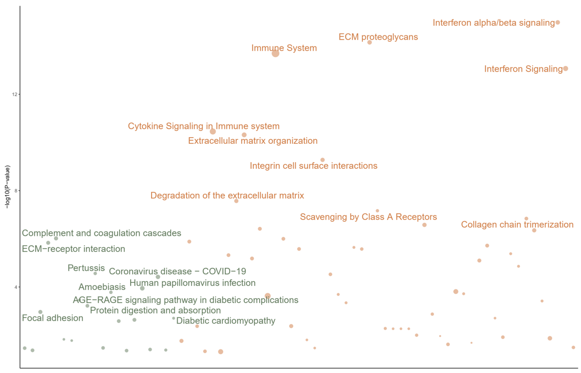

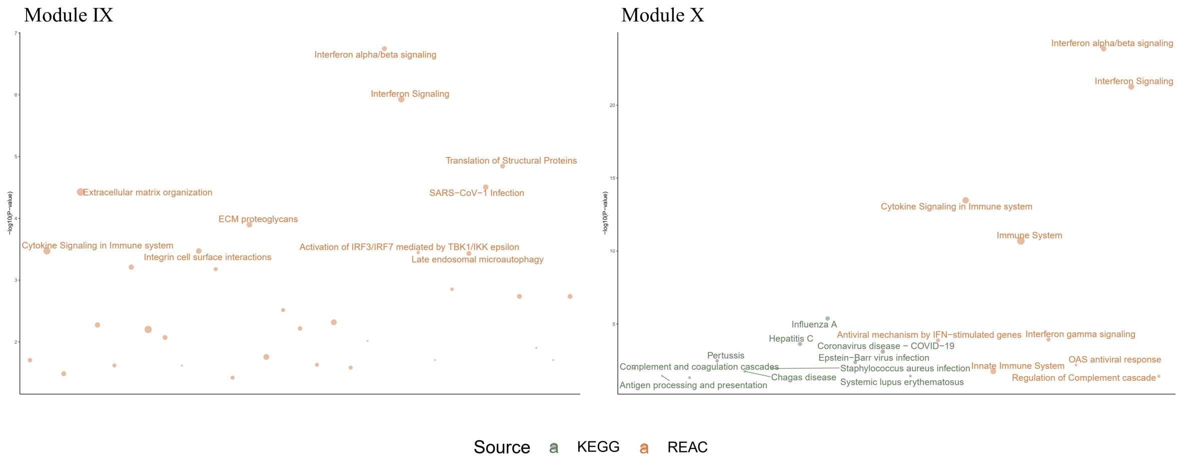

Fig. S39. Functional enrichment analysis of the tissue modules identified by STModule from melanoma sample 2. Y-axis represents -$\log_{10}$(P-value). Color indicates the source of the pathways. Point size indicates the number of overlapping genes of the modules and the pathways. X-axis represents the id and type of the pathways.

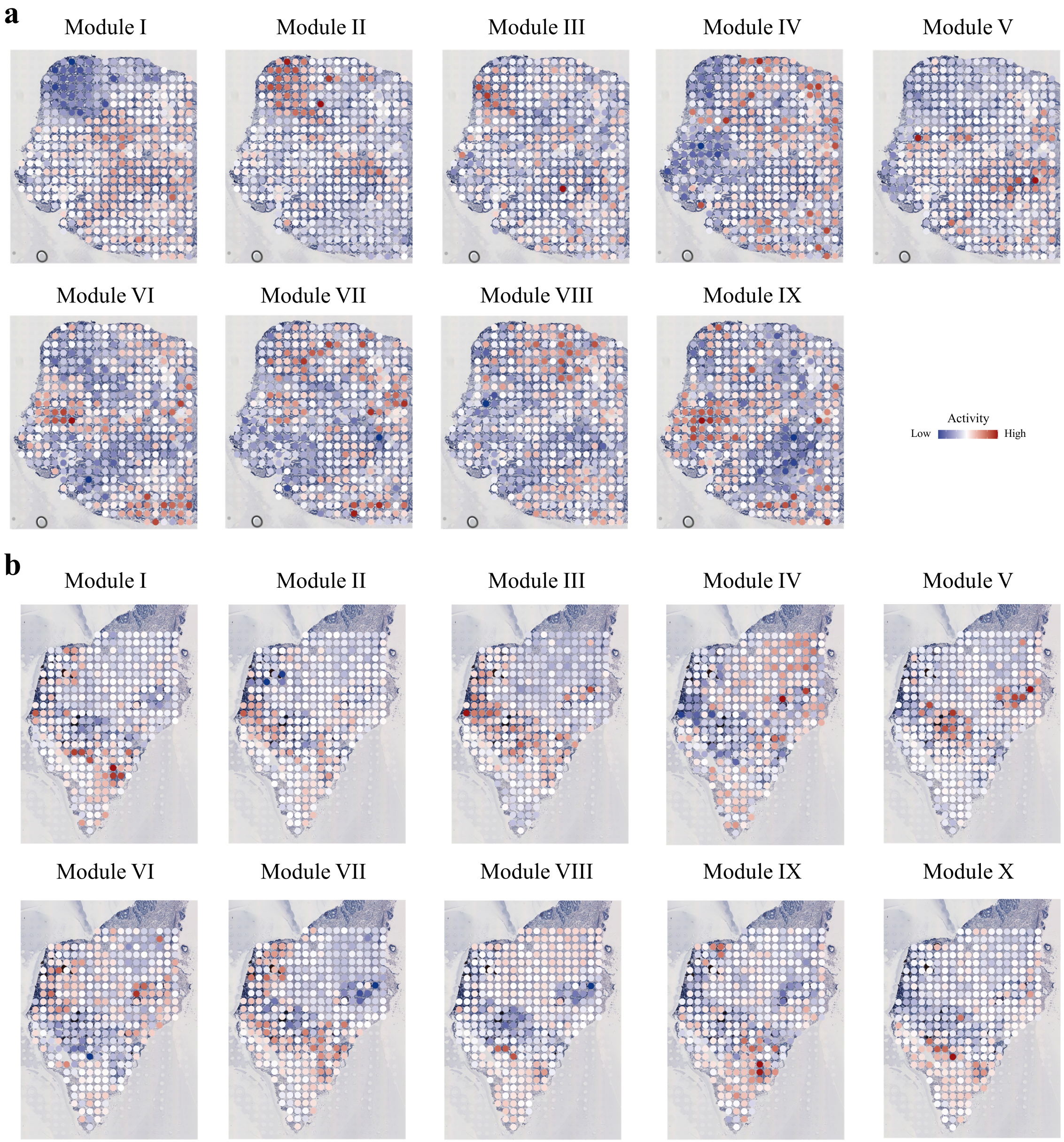

Fig. S40. Spatial maps of the tissue modules identified by STModule from the prostate cancer dataset. a Spatial maps for sample P1.2. Annotations of the tissue modules: PIN vs. tumor (I), shared feature of cancer and PIN (II), center of tumor (III), smooth muscle (IV), luminal epithelial cells (V), basal epithelial cells (VI), club epithelial cells (VII), fibroblast (VIII), immune & stress (IX). **b** Spatial maps for sample P4.2. Annotations of the tissue modules: inflammation (I), ATF3 (II), prostatic gland (III), smooth muscle (IV), luminal epithelial cell-1 (V), luminal epithelial cell-2 (VI), basal epithelial cells (VII), Hillock cells (VIII), Club cells (IX), B cells (X).

a

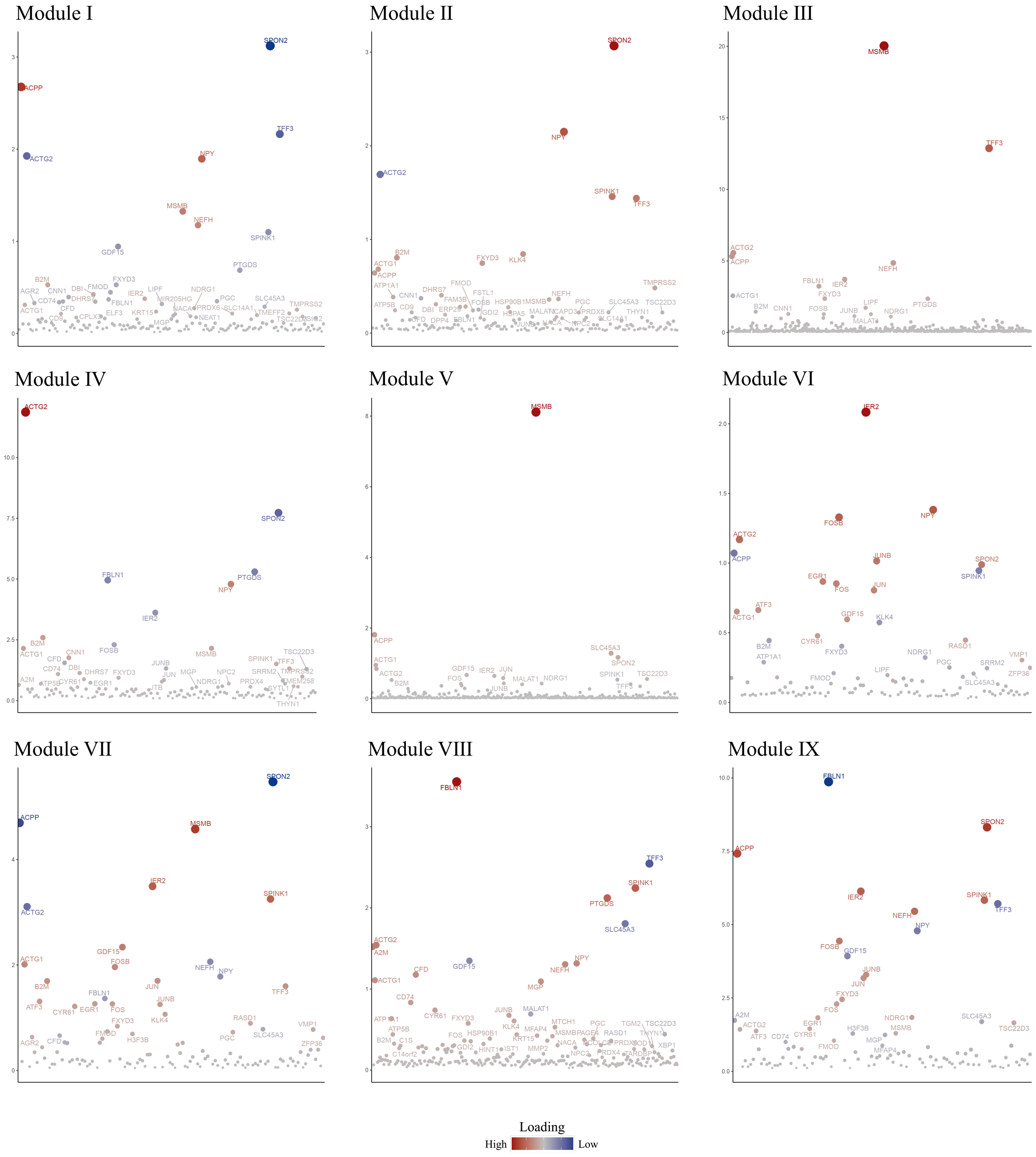
Module I
SPON2
ACPP
TFF3
ACTG2
NPY
MSMB
NEFH
SPINK1
GDF15
PTGDS
Module II
SPON2
NPY
ACTG2
SPINK1
TFF3
Module III
MSMB
TFF3
Module IV
ACTG2
SPON2
FBLN1
PTGDS
NPY
IER2
Module V
MSMB
Module VI
IER2
NPY
FOSB
ACTG2
ACPP
JUNB
SPON2
SPINK1
Module VII
SPON2
ACPP
MSMB
ACTG2
IER2
SPINK1
Module VIII
FBLN1
TFF3
SPINK1
PTGDS
SLC45A3
Module IX
FBLN1
SPON2
ACPP
IER2
SPINK1
TFF3
NEFH
NPY
Loading
High
Low

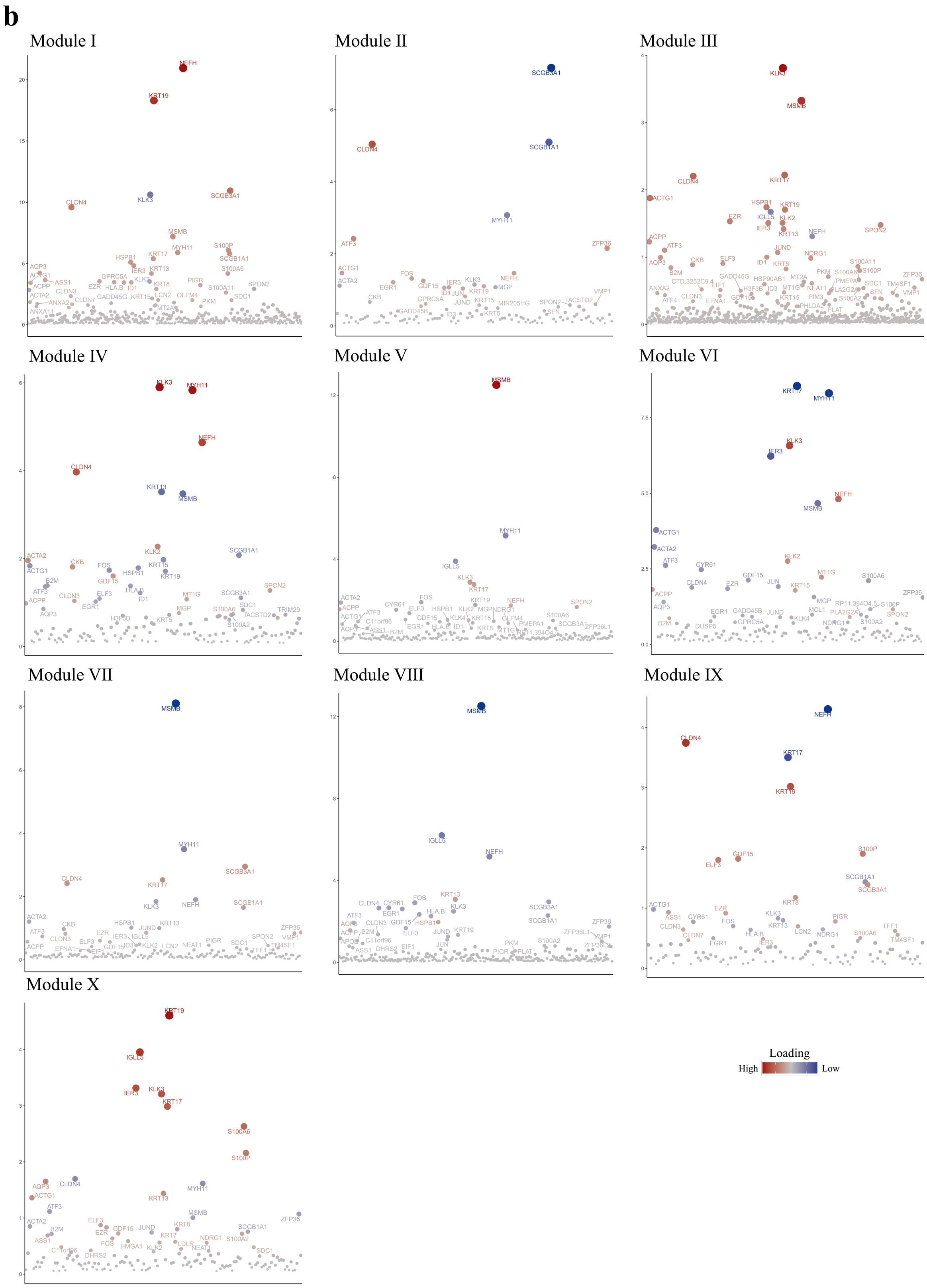

Fig. S41. Gene loadings of the tissue modules identified by STModule from the prostate cancer dataset. a Results for sample P1.2. **b** Results for sample P4.2. Y-axis represents the absolute value of gene loading and color indicates the sign and value of loading. X-axis represents the alphabetical order of the gene names.

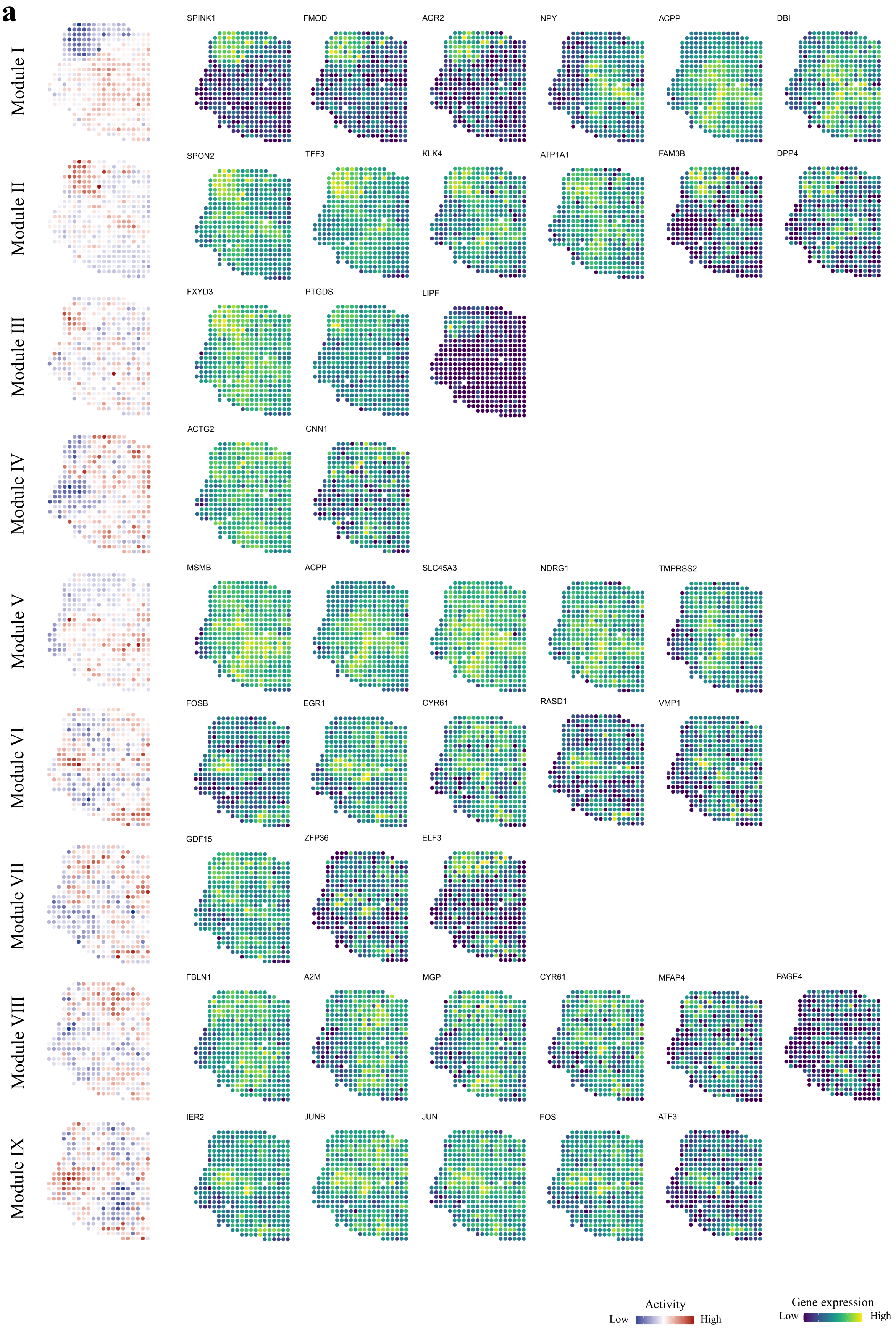

a
Module I
SPINK1
FMOD
AGR2
NPY
ACPP
DBI
Module II
SPON2
TFF3
KLK4
ATP1A1
FAM3B
DPP4
Module III
FXYD3
PTGDS
LIPF
Module IV
ACTG2
CNN1
Module V
MSMB
ACPP
SLC45A3
NDRG1
TMPRSS2
Module VI
FOSB
EGR1
CYR61
RASD1
VMP1
Module VII
GDF15
ZFP36
ELF3
Module VIII
FBLN1
A2M
MGP
CYR61
MFAP4
PAGE4
Module IX
IER2
JUNB
JUN
FOS
ATF3
Activity
Low
High
Gene expression
Low
High

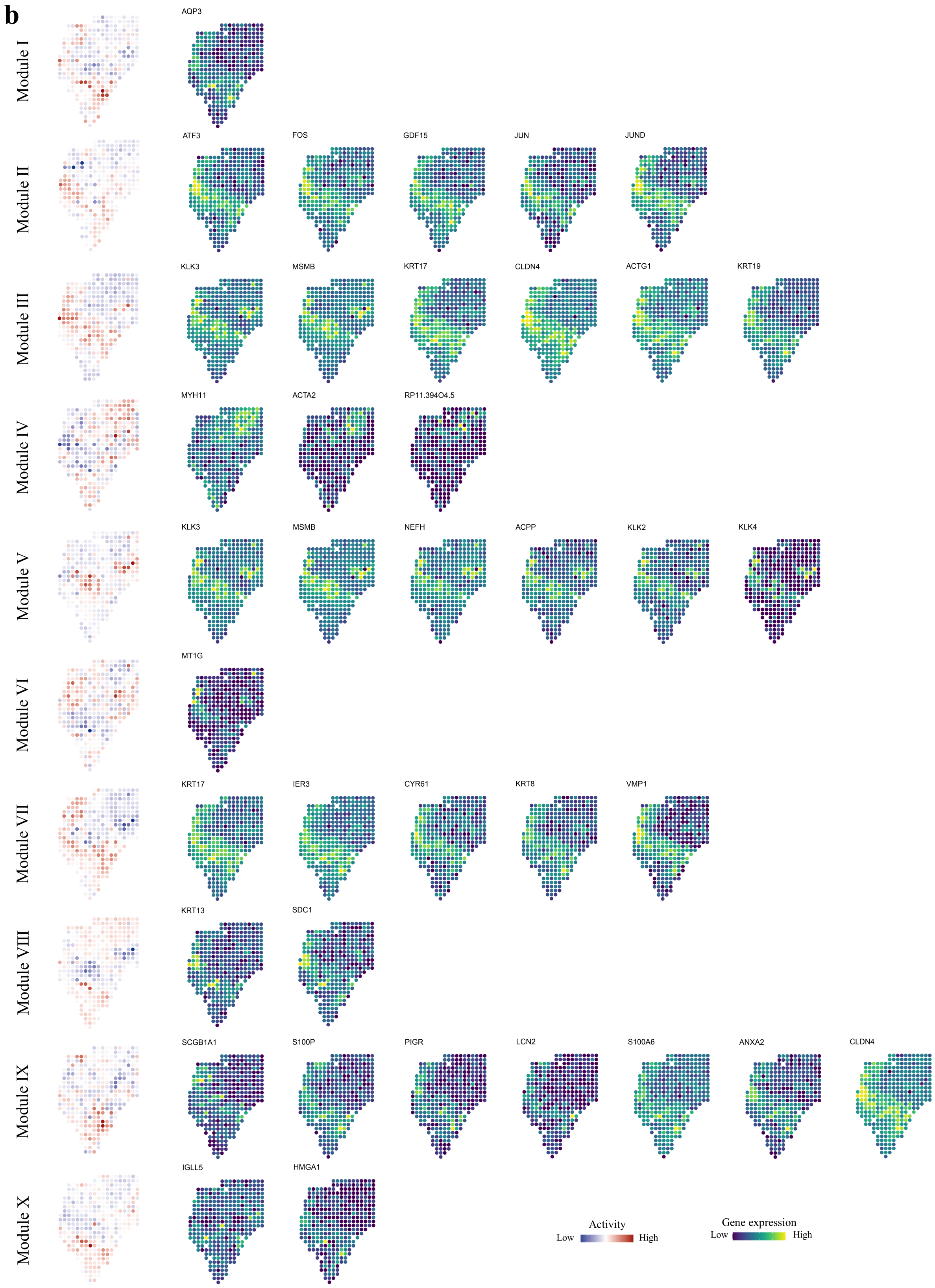

Fig. S42. Spatial expression of representative associated genes of the tissue modules identified by STModule from the prostate cancer dataset. a Results for sample P1.2. **b** Results for sample P4.2.

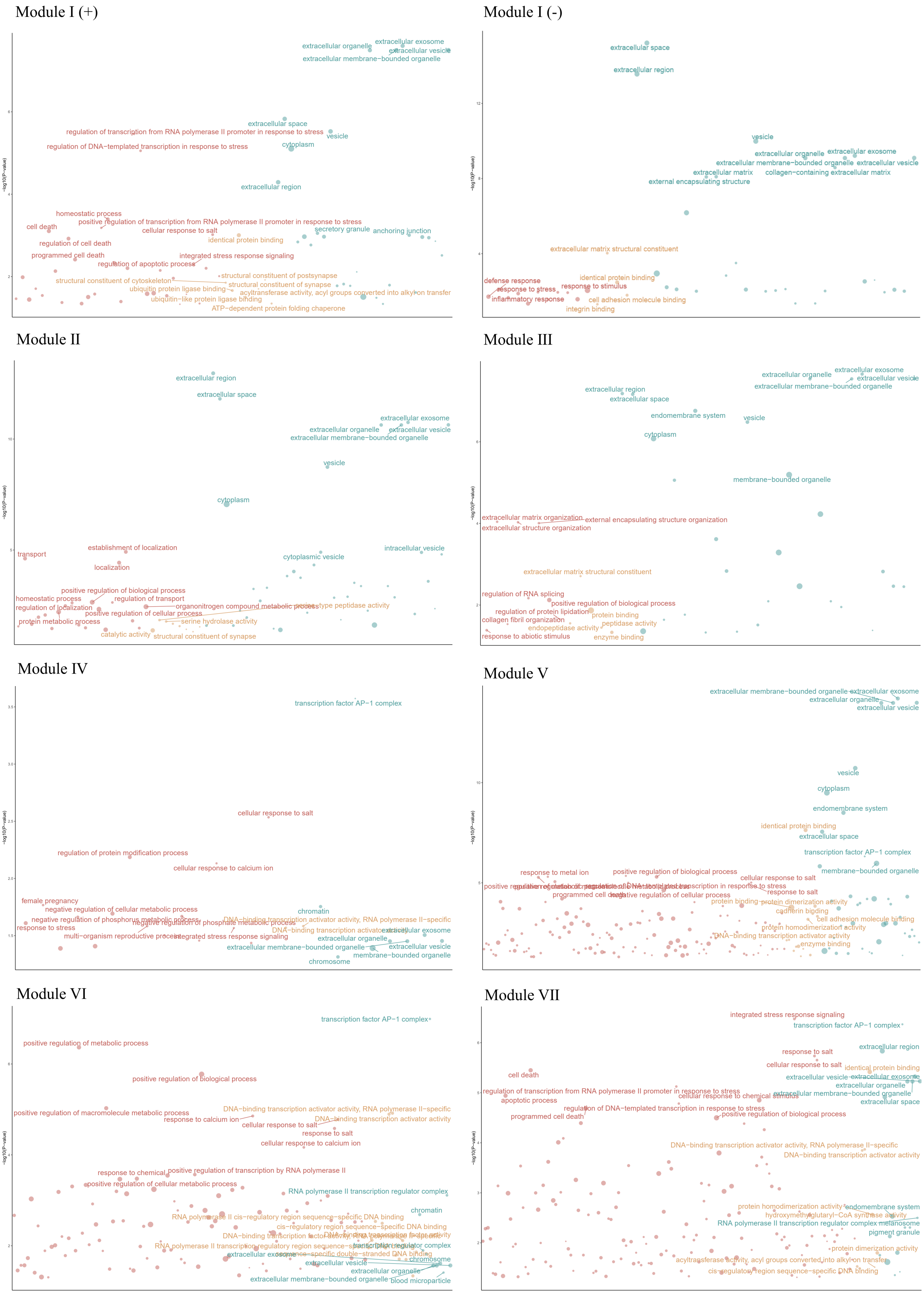
Module I (+)
extracellular exosome
extracellular organelle
extracellular vesicle
extracellular membrane−bounded organelle
extracellular space
regulation of transcription from RNA polymerase II promoter in response to stress
vesicle
regulation of DNA−templated transcription in response to stress
cytoplasm
extracellular region
homeostatic process
positive regulation of transcription from RNA polymerase II promoter in response to stress
cell death
cellular response to salt
secretory granule
anchoring junction
regulation of cell death
identical protein binding
programmed cell death
integrated stress response signaling
regulation of apoptotic process
structural constituent of postsynapse
structural constituent of cytoskeleton
structural constituent of synapse
ubiquitin protein ligase binding
acyltransferase activity, acyl groups converted into alkyl on transfer
ubiquitin−like protein ligase binding
ATP−dependent protein folding chaperone
-log10(P-value)
Module I (-)
extracellular space
extracellular region
vesicle
extracellular organelle
extracellular exosome
extracellular membrane−bounded organelle
extracellular vesicle
extracellular matrix
collagen−containing extracellular matrix
external encapsulating structure
extracellular matrix structural constituent
defense response
identical protein binding
response to stress
response to stimulus
inflammatory response
cell adhesion molecule binding
integrin binding
Module II
extracellular region
extracellular space
extracellular exosome
extracellular organelle
extracellular vesicle
extracellular membrane−bounded organelle
vesicle
cytoplasm
establishment of localization
intracellular vesicle
transport
cytoplasmic vesicle
localization
positive regulation of biological process
homeostatic process
regulation of transport
regulation of localization
organonitrogen compound metabolic process
serine−type peptidase activity
positive regulation of cellular process
protein metabolic process
serine hydrolase activity
catalytic activity
structural constituent of synapse
Module III
extracellular exosome
extracellular organelle
extracellular vesicle
extracellular region
extracellular membrane−bounded organelle
extracellular space
endomembrane system
vesicle
cytoplasm
membrane−bounded organelle
extracellular matrix organization
external encapsulating structure organization
extracellular structure organization
extracellular matrix structural constituent
regulation of RNA splicing
positive regulation of biological process
regulation of protein lipidation
protein binding
collagen fibril organization
peptidase activity
endopeptidase activity
response to abiotic stimulus
enzyme binding
Module IV
transcription factor AP−1 complex
cellular response to salt
regulation of protein modification process
cellular response to calcium ion
female pregnancy
chromatin
negative regulation of cellular metabolic process
negative regulation of phosphorus metabolic process
DNA−binding transcription activator activity, RNA polymerase II−specific
negative regulation of phosphate metabolic process
response to stress
DNA−binding transcription activator activity
extracellular exosome
multi−organism reproductive process
integrated stress response signaling
extracellular organelle
extracellular membrane−bounded organelle
extracellular vesicle
membrane−bounded organelle
chromosome
Module V
extracellular membrane−bounded organelle
extracellular exosome
extracellular organelle
extracellular vesicle
vesicle
cytoplasm
endomembrane system
identical protein binding
extracellular space
transcription factor AP−1 complex
membrane−bounded organelle
response to metal ion
positive regulation of biological process
cellular response to salt
response to salt
programmed cell death
negative regulation of cellular process
protein binding
protein dimerization activity
cadherin binding
cell adhesion molecule binding
protein homodimerization activity
DNA−binding transcription activator activity
enzyme binding
Module VI
transcription factor AP−1 complex
positive regulation of metabolic process
positive regulation of biological process
DNA−binding transcription activator activity, RNA polymerase II−specific
positive regulation of macromolecule metabolic process
response to calcium ion
DNA−binding transcription activator activity
cellular response to salt
response to salt
cellular response to calcium ion
response to chemical
positive regulation of transcription by RNA polymerase II
positive regulation of cellular metabolic process
RNA polymerase II transcription regulator complex
chromatin
RNA polymerase II cis−regulatory region sequence−specific DNA binding
cis−regulatory region sequence−specific DNA binding
DNA−binding transcription factor activity, RNA polymerase II−specific
RNA polymerase II transcription regulatory region sequence−specific DNA binding
extracellular exosome
sequence−specific double−stranded DNA binding
chromosome
extracellular vesicle
extracellular organelle
extracellular membrane−bounded organelle
blood microparticle
Module VII
integrated stress response signaling
transcription factor AP−1 complex
extracellular region
response to salt
cell death
cellular response to salt
identical protein binding
extracellular vesicle
extracellular exosome
extracellular organelle
regulation of transcription from RNA polymerase II promoter in response to stress
cellular response to chemical stimulus
extracellular membrane−bounded organelle
apoptotic process
extracellular space
regulation of DNA−templated transcription in response to stress
programmed cell death
positive regulation of biological process
DNA−binding transcription activator activity, RNA polymerase II−specific
DNA−binding transcription activator activity
protein homodimerization activity
endomembrane system
hydroxymethylglutaryl−CoA synthase activity
RNA polymerase II transcription regulator complex
melanosome
pigment granule
protein dimerization activity
acyltransferase activity, acyl groups converted into alkyl on transfer
cis−regulatory region sequence−specific DNA binding

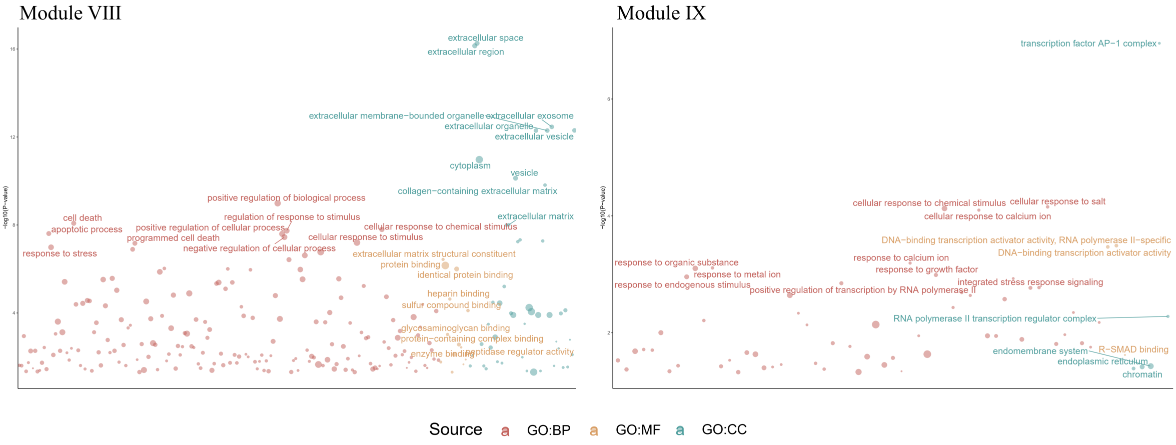

Fig. S43. GO enrichment analysis of the tissue modules identified by STModule from prostate sample P1.2. Y-axis represents -$\log_{10}$(P-value). Color indicates the source of the GO terms. Point size indicates the number of overlapping genes of the modules and the terms. X-axis represents the id and type of the terms.

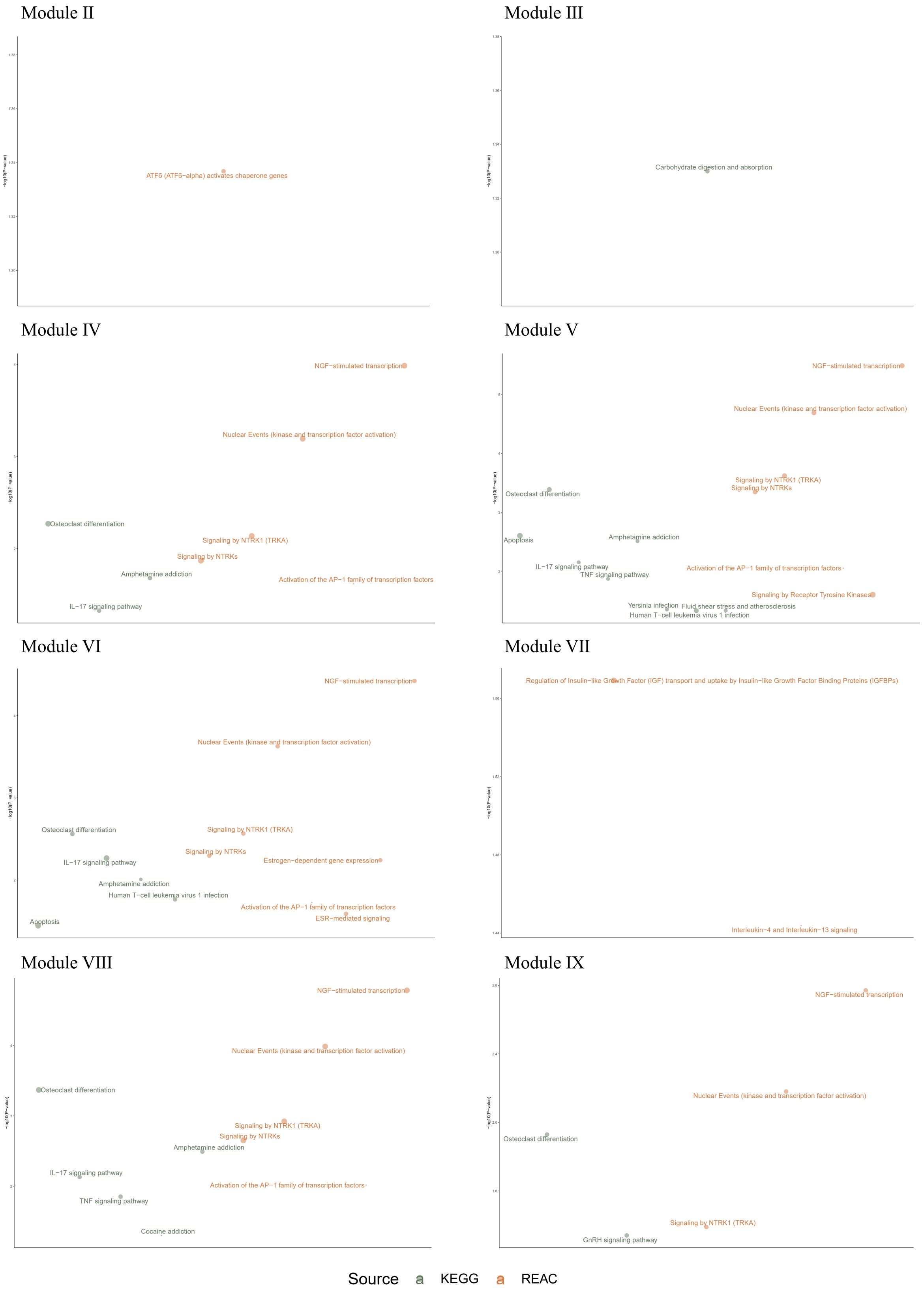

Fig. S44. Functional enrichment analysis of the tissue modules identified by STModule from prostate sample P1.2. Y-axis represents -log$_{10}$(P-value). Color indicates the source of the pathways. Point size indicates the number of overlapping genes of the modules and the pathways. X-axis represents the id and type of the pathways.

Module I

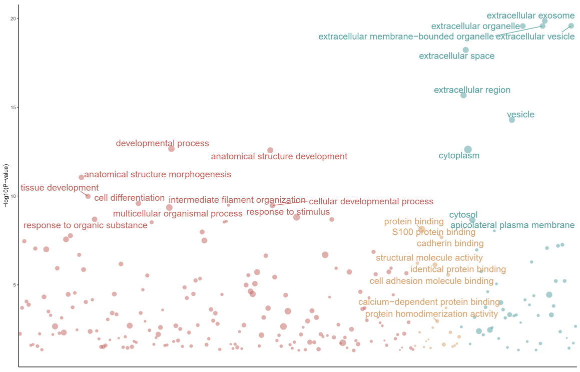

Module II

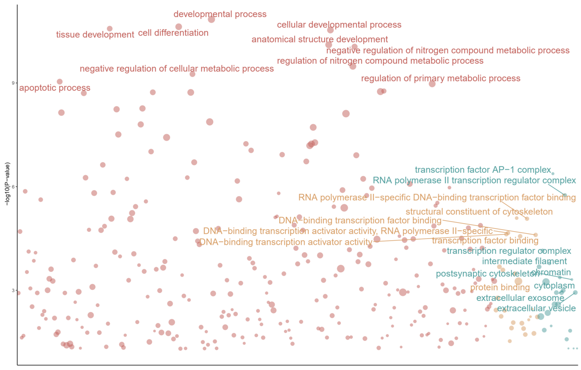

Module III

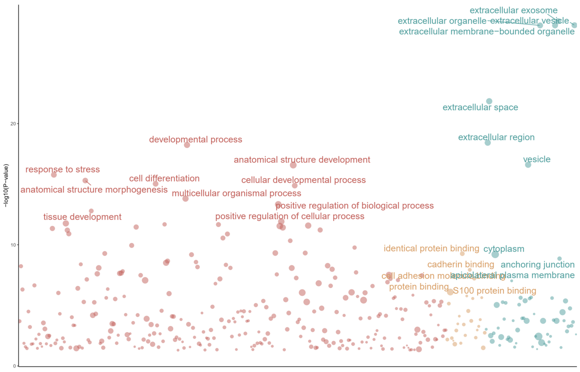

Module IV

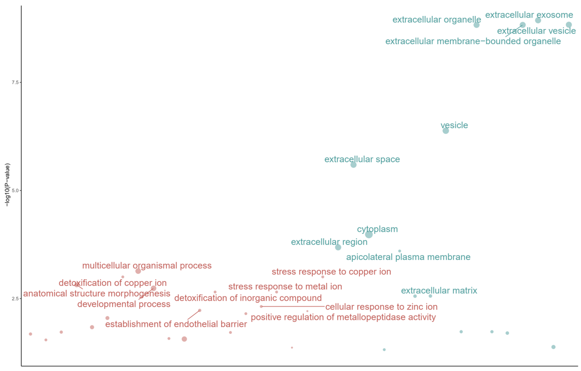

Module V

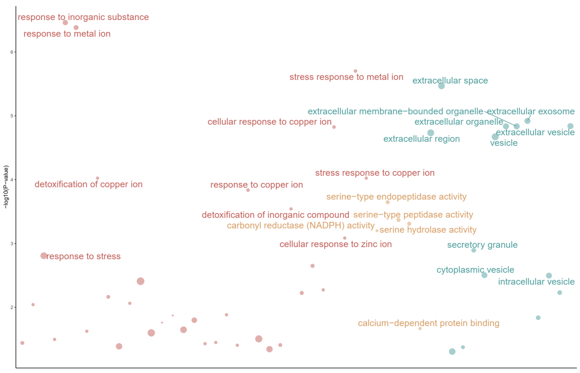

Module VI

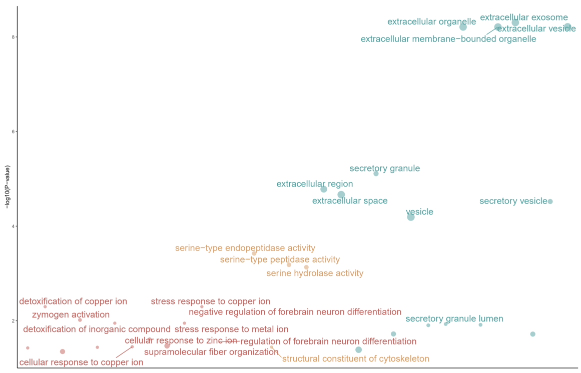

Module VII

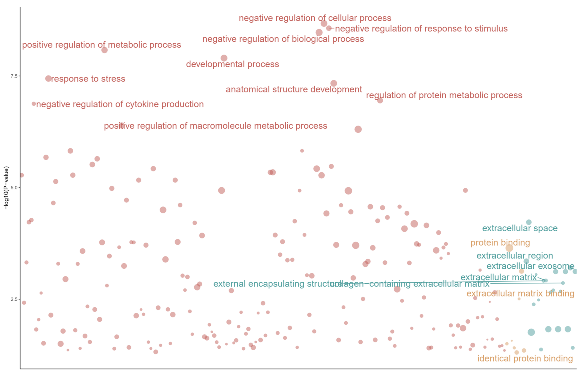

Module VIII

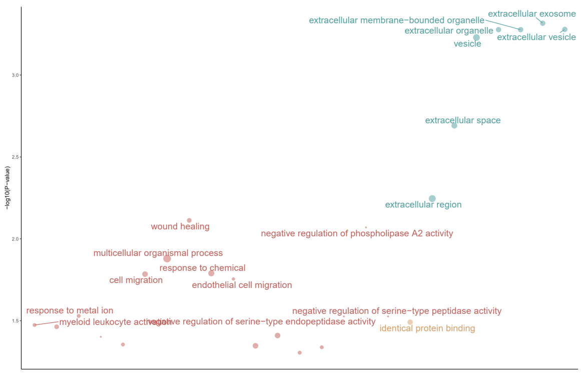

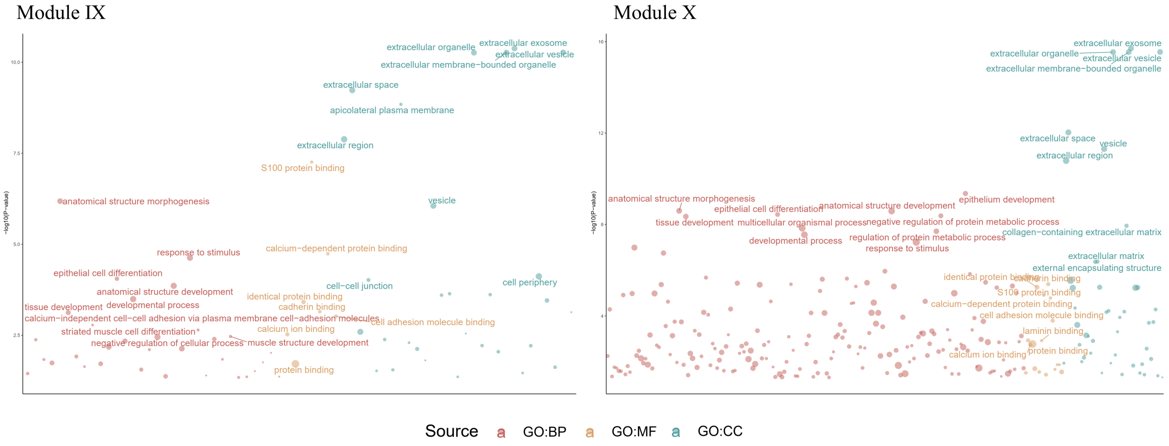

Fig. S45. GO enrichment analysis of the tissue modules identified by STModule from prostate sample P4.2. Y-axis represents -$\log_{10}$(P-value). Color indicates the source of the GO terms. Point size indicates the number of overlapping genes of the modules and the terms. X-axis represents the id and type of the terms.

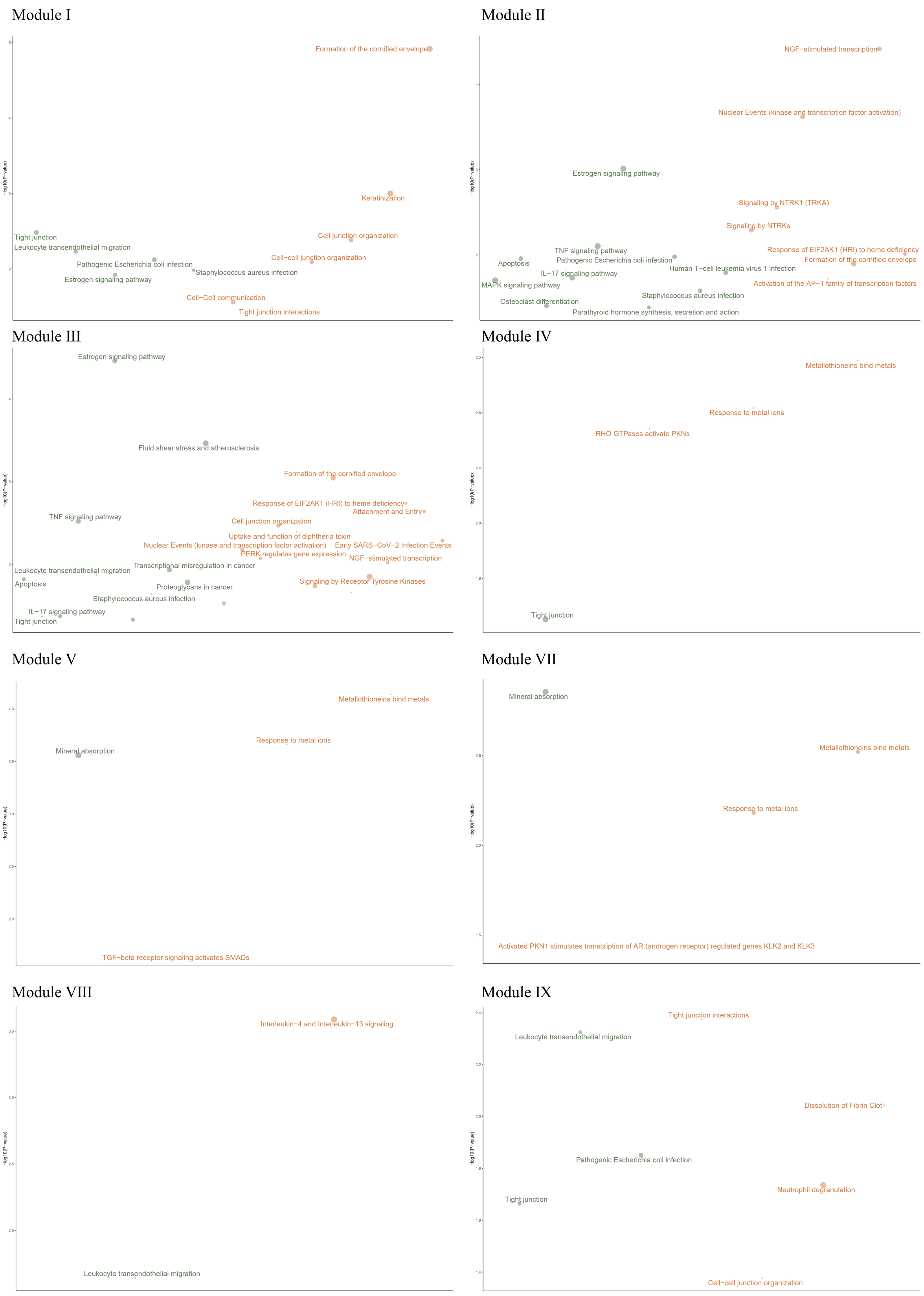
Module I
Formation of the cornified envelope
Keratinization
Tight junction
Leukocyte transendothelial migration
Cell junction organization
Cell−cell junction organization
Pathogenic Escherichia coli infection
Staphylococcus aureus infection
Estrogen signaling pathway
Cell−Cell communication
Tight junction interactions
−log10(P−value)
Module II
NGF−stimulated transcription
Nuclear Events (kinase and transcription factor activation)
Estrogen signaling pathway
Signaling by NTRK1 (TRKA)
Signaling by NTRKs
Response of EIF2AK1 (HRI) to heme deficiency
TNF signaling pathway
Pathogenic Escherichia coli infection
Formation of the cornified envelope
Apoptosis
Human T−cell leukemia virus 1 infection
IL−17 signaling pathway
Activation of the AP−1 family of transcription factors
MAPK signaling pathway
Staphylococcus aureus infection
Osteoclast differentiation
Parathyroid hormone synthesis, secretion and action
Module III
Estrogen signaling pathway
Fluid shear stress and atherosclerosis
Formation of the cornified envelope
Response of EIF2AK1 (HRI) to heme deficiency
Attachment and Entry
TNF signaling pathway
Cell junction organization
Uptake and function of diphtheria toxin
Nuclear Events (kinase and transcription factor activation)
Early SARS−CoV−2 Infection Events
PERK regulates gene expression
NGF−stimulated transcription
Leukocyte transendothelial migration
Transcriptional misregulation in cancer
Apoptosis
Signaling by Receptor Tyrosine Kinases
Proteoglycans in cancer
Staphylococcus aureus infection
IL−17 signaling pathway
Tight junction
Module IV
Metallothioneins bind metals
Response to metal ions
RHO GTPases activate PKNs
Tight junction
Module V
Metallothioneins bind metals
Response to metal ions
Mineral absorption
TGF−beta receptor signaling activates SMADs
Module VII
Mineral absorption
Metallothioneins bind metals
Response to metal ions
Activated PKN1 stimulates transcription of AR (androgen receptor) regulated genes KLK2 and KLK3
Module VIII
Interleukin−4 and Interleukin−13 signaling
Leukocyte transendothelial migration
Module IX
Tight junction interactions
Leukocyte transendothelial migration
Dissolution of Fibrin Clot
Pathogenic Escherichia coli infection
Neutrophil degranulation
Tight junction
Cell−cell junction organization

Module X

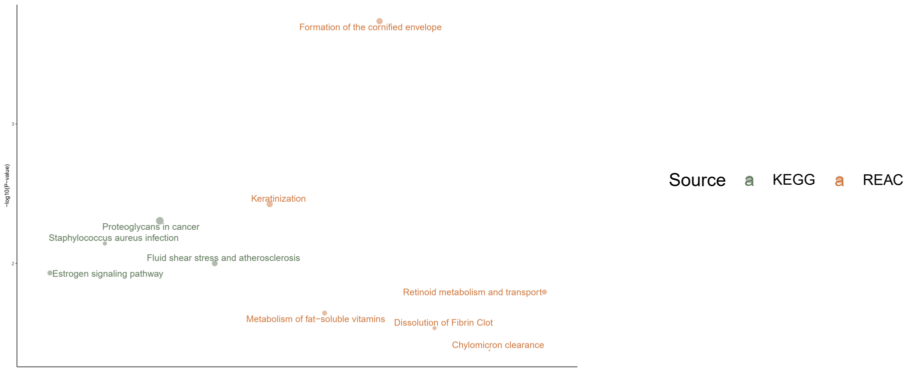

Fig. S46. Functional enrichment analysis of the tissue modules identified by STModule from prostate sample P4.2. Y-axis represents -$\log_{10}$(P-value). Color indicates the source of the pathways. Point size indicates the number of overlapping genes of the modules and the pathways. X-axis represents the id and type of the pathways.

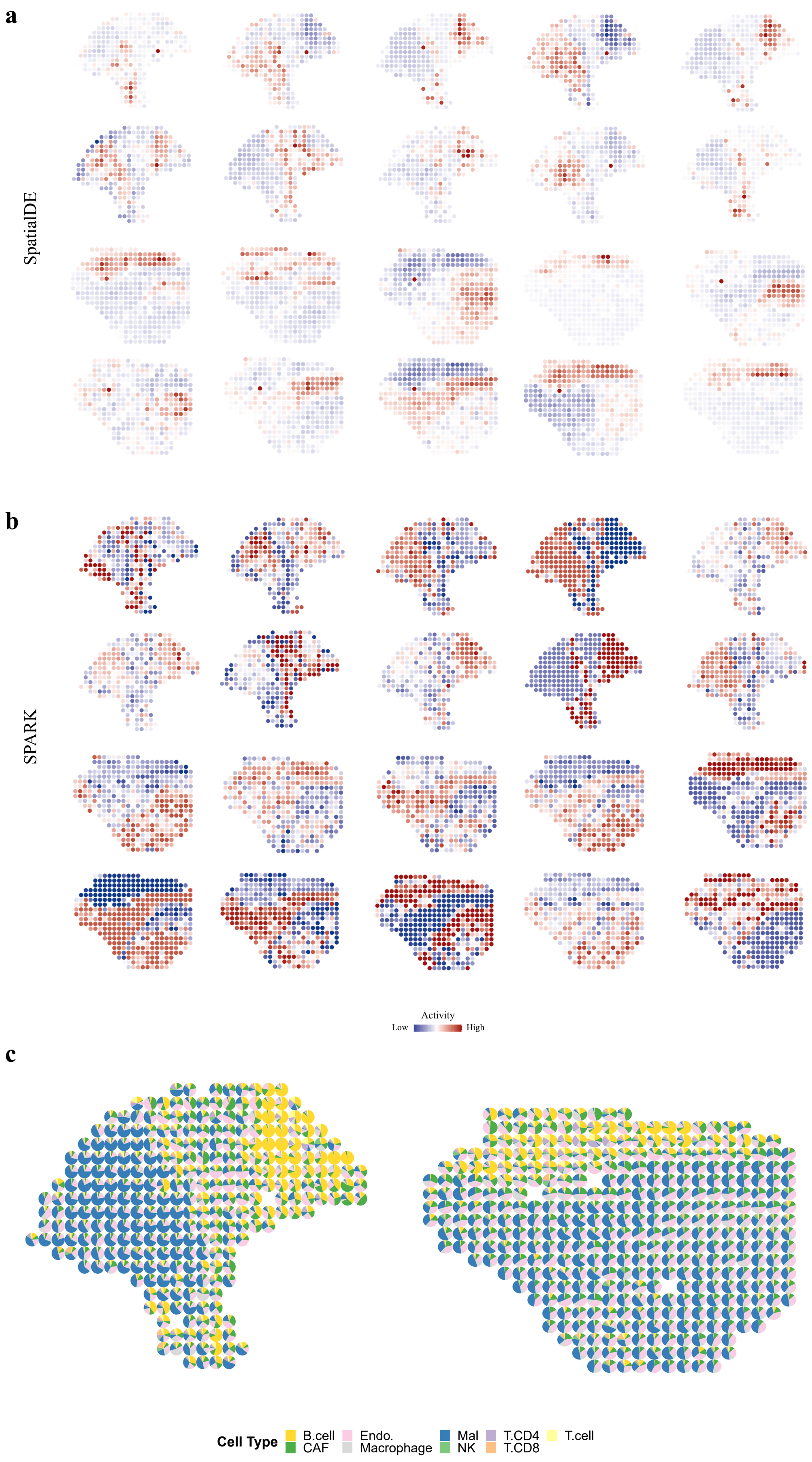
a
SpatialDE
b
SPARK
Activity
Low
High
c
Cell Type
B.cell
CAF
Endo.
Macrophage
Mal
NK
T.CD4
T.CD8
T.cell

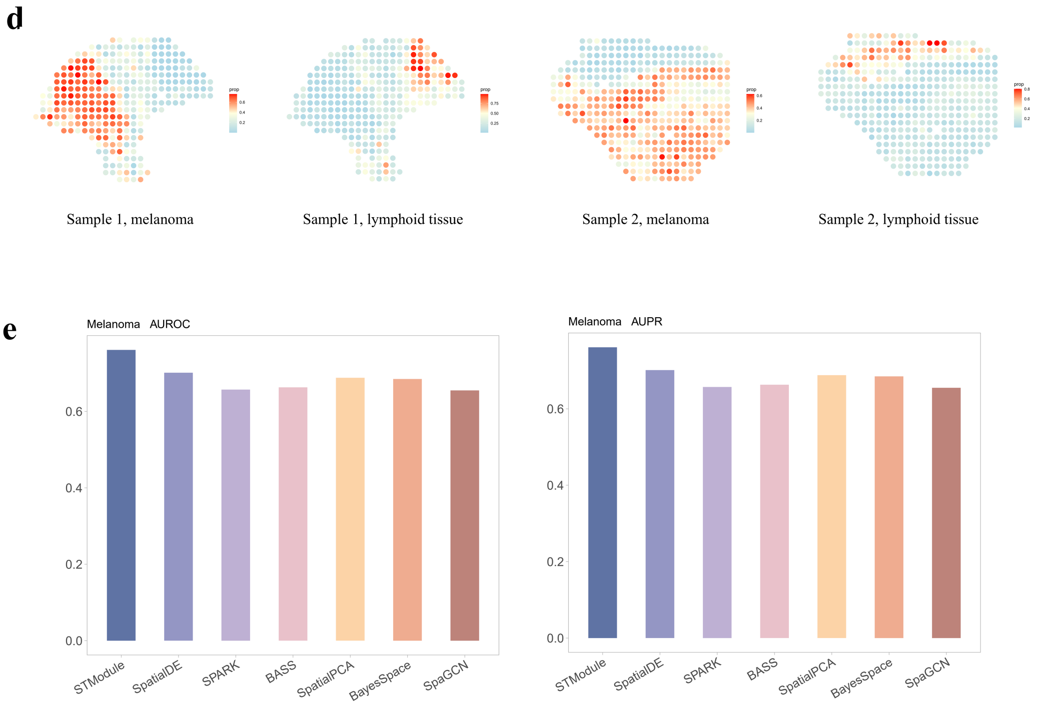

Fig. S47. Additional comparison for the melanoma dataset. a, b Spatial patterns identified by SpatialDE (**a**) and SPARK (**b**) from the melanoma samples. **c** Spatial distribution of major cell types estimated by CARD. **d** Spatial distribution of melanoma and lymphoid domains estimated by CARD. **e** Comparison of AUROC and AUPR of the methods in detecting the melanoma and lymphoid domains.

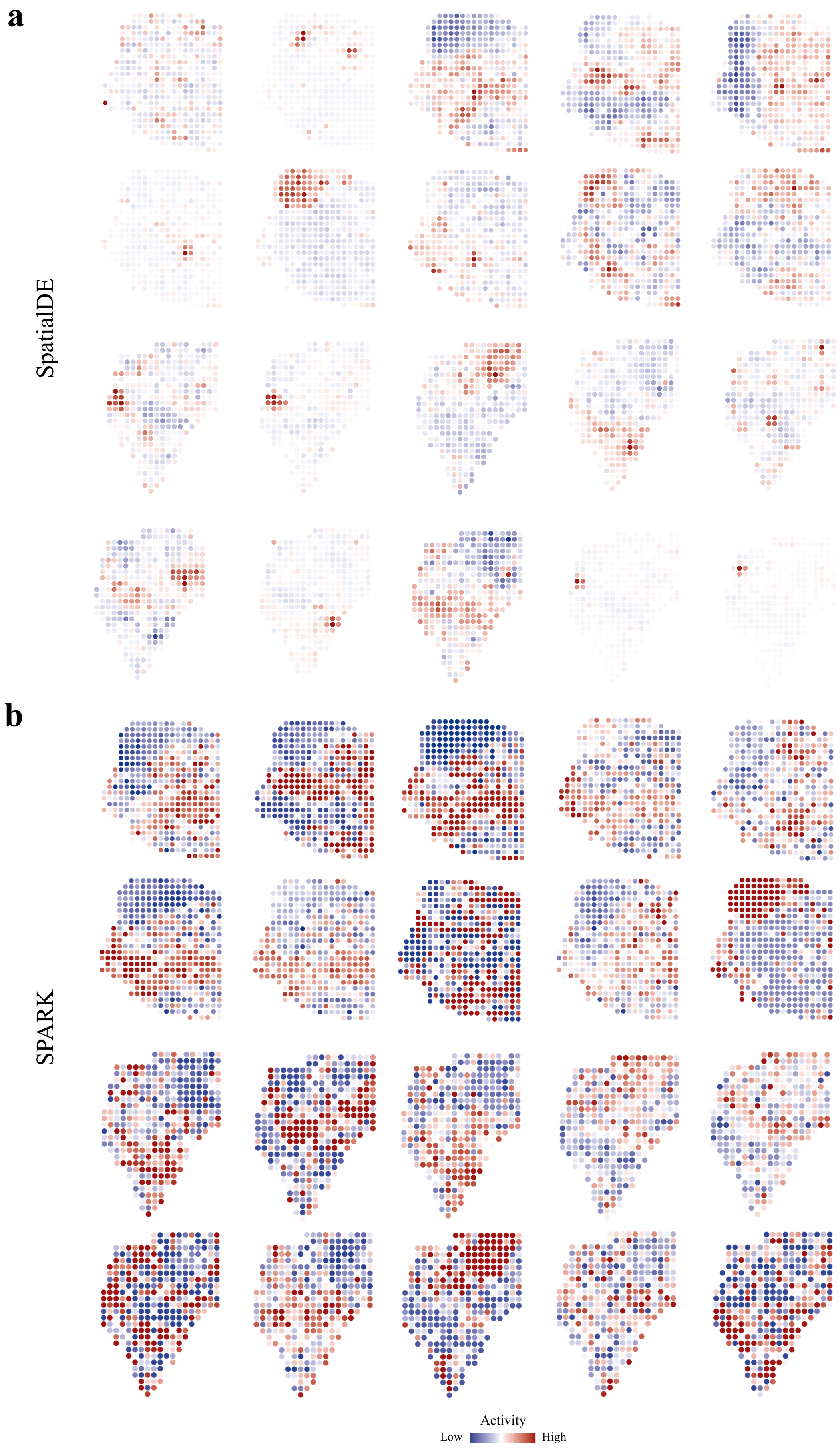
a
SpatialDE
b
SPARK
Activity
Low
High

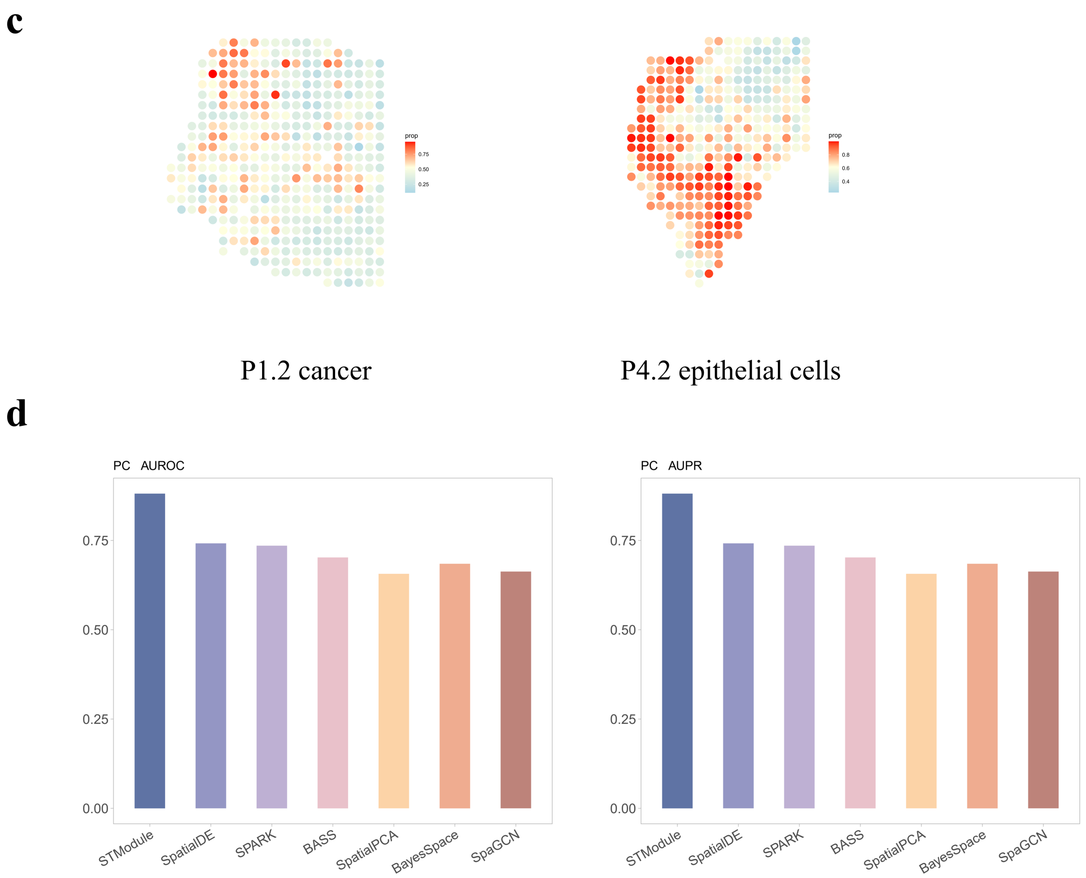

Fig. S48. Additional comparison for the prostate dataset. a, b Spatial patterns identified by SpatialDE (**a**) and SPARK (**b**) from the prostate samples. **c** Spatial distribution of cancer and epithelial cells estimated by CARD. **d** Comparison of AUROC and AUPR of the methods in detecting the cancer and epithelial domains.

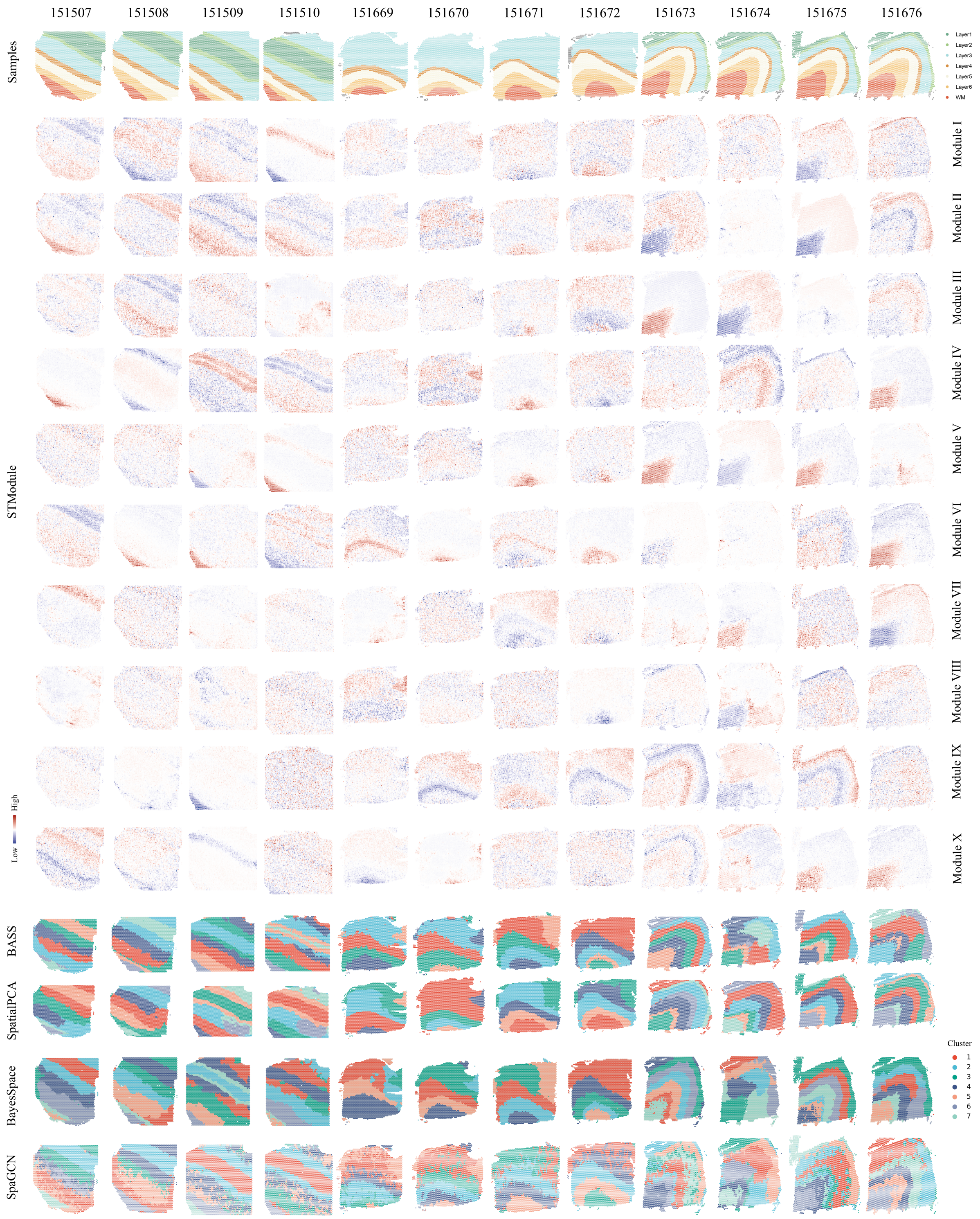

Fig. S49. Results for all twelve samples in the DLPFC dataset. Each column demonstrates the results of a sample, including the annotated structure of the sample, the spatial maps of the tissue modules identified by STModule, and the clustering results of BASS, SpatialPCA, BayesSpace and SpaGCN.

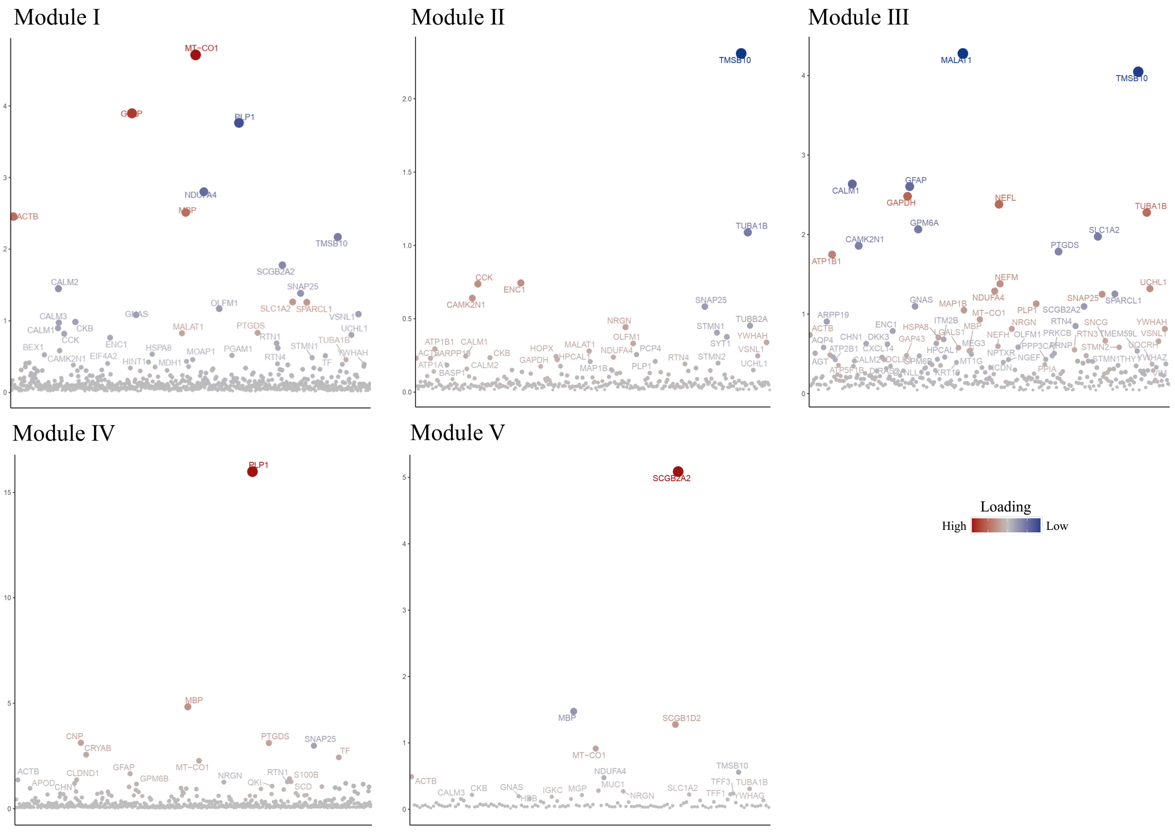

Fig. S50. Gene loadings of the tissue modules identified by STModule from DLPFC sample 151676. Y-axis represents the absolute value of gene loading and color indicates the sign and value of loading. X-axis represents the alphabetical order of the gene names.

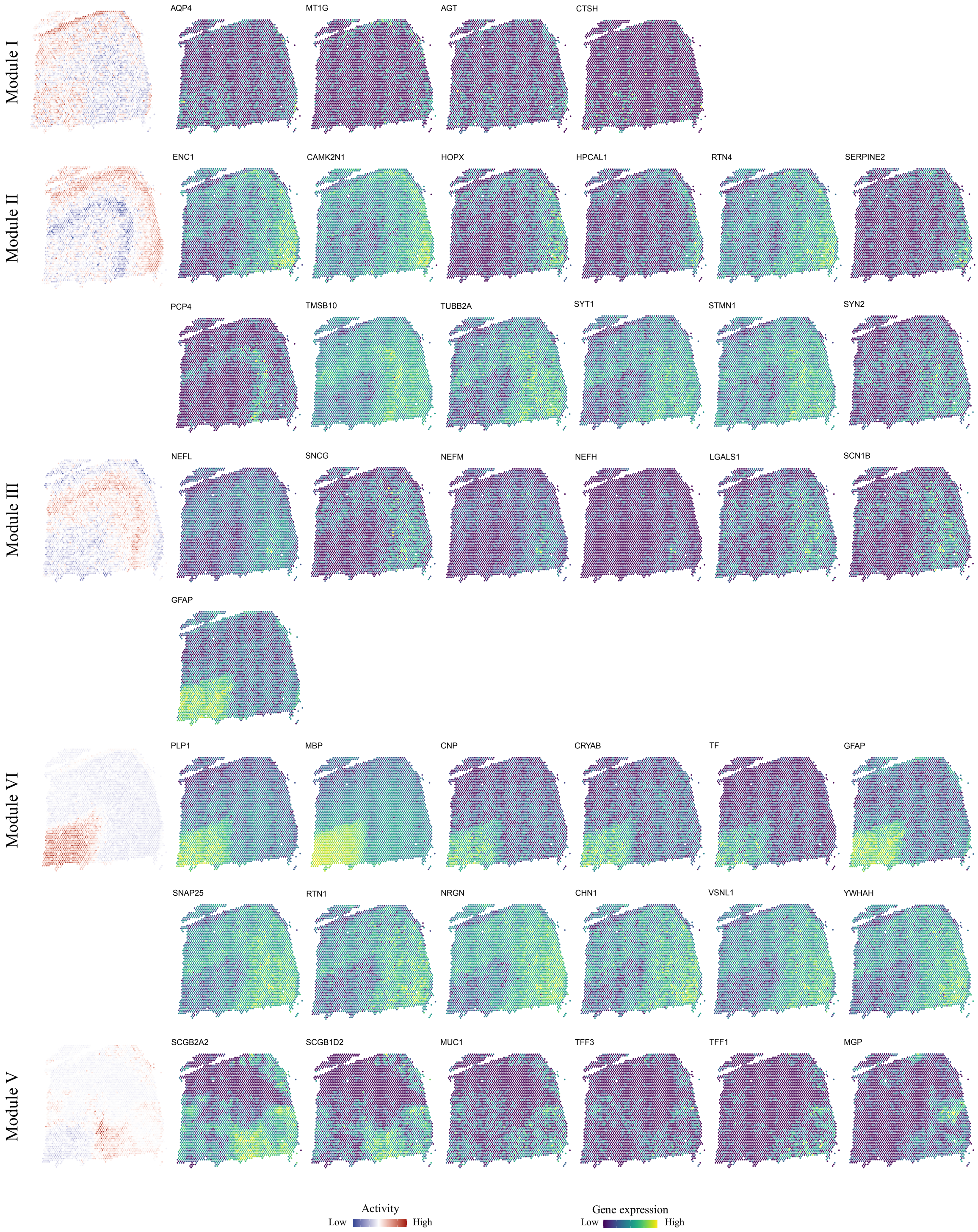

Fig. S51. Spatial expression of representative associated genes of the tissue modules identified by STModule from DLPFC sample 151676.

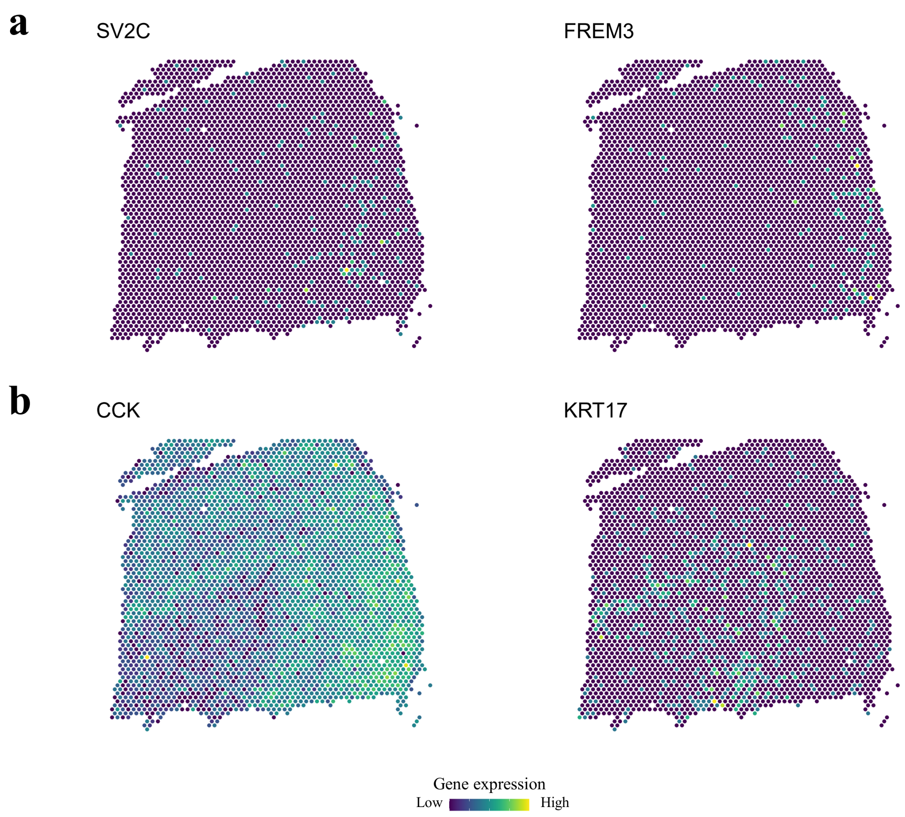

Fig. S52. Spatial expression of marker genes of layer 3 (a) and layer 6 (b) identified in previous studies in DLPFC sample 151676.

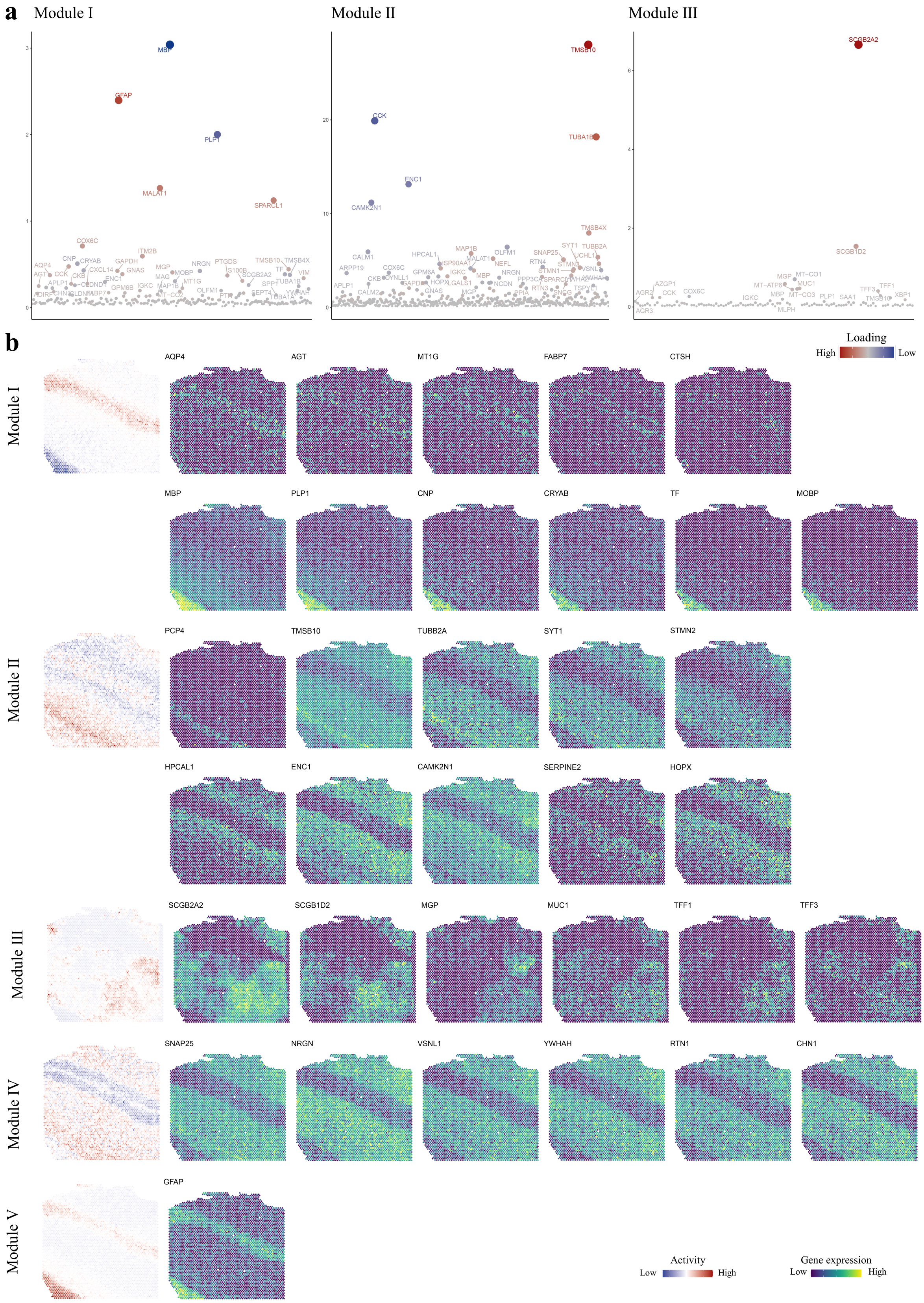

Fig. S53. Additional results of the tissue modules identified by STModule from DLPFC sample 151510. a Gene loadings of the tissue modules. Y-axis represents the absolute value of gene loading and color indicates the sign and value of loading. X-axis represents the alphabetical order of the gene names. **b** Spatial expression of representative associated genes of the tissue modules.

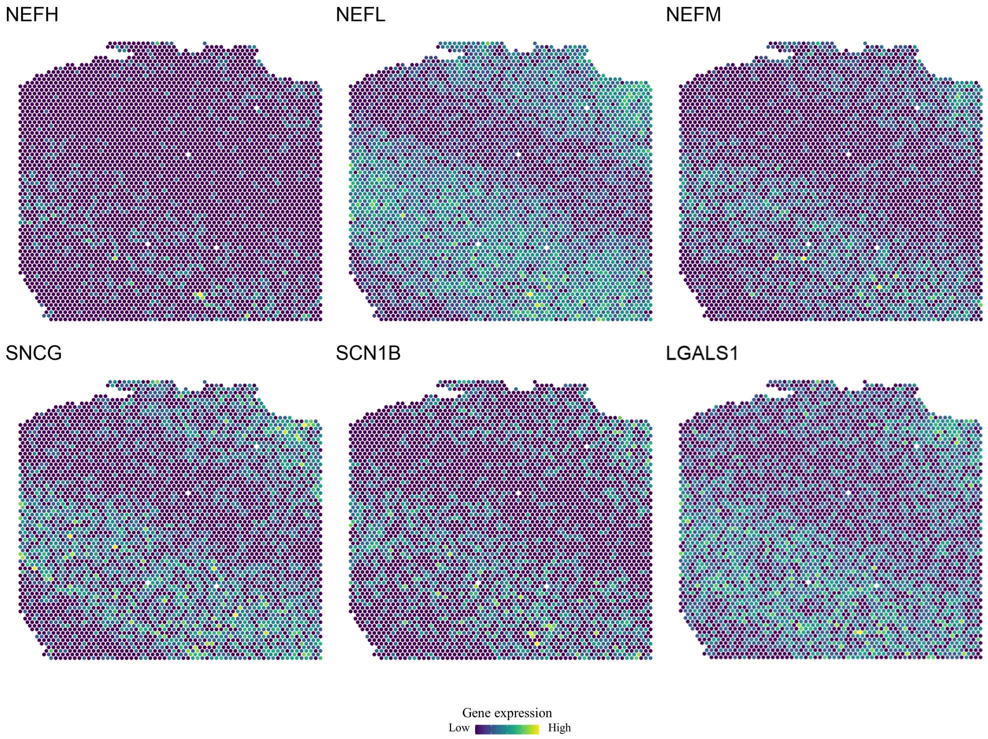

Fig. S54. Spatial expression of genes in DLPFC sample 151510, which are highly expressed in layer 4 of DLPFC sample 151676.

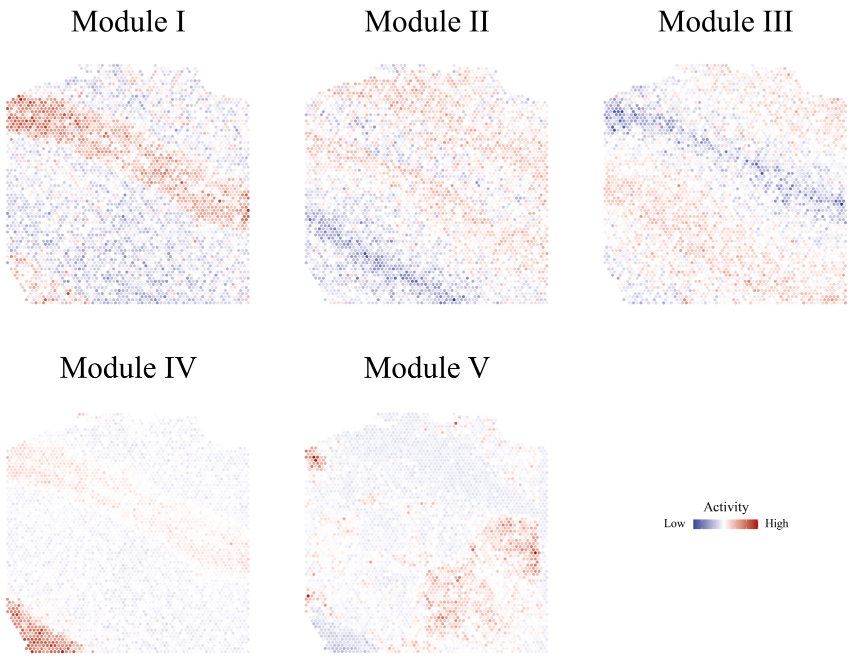

Fig. S55. Application of tissue modules identified from DLPFC sample 151676 to sample 151510.

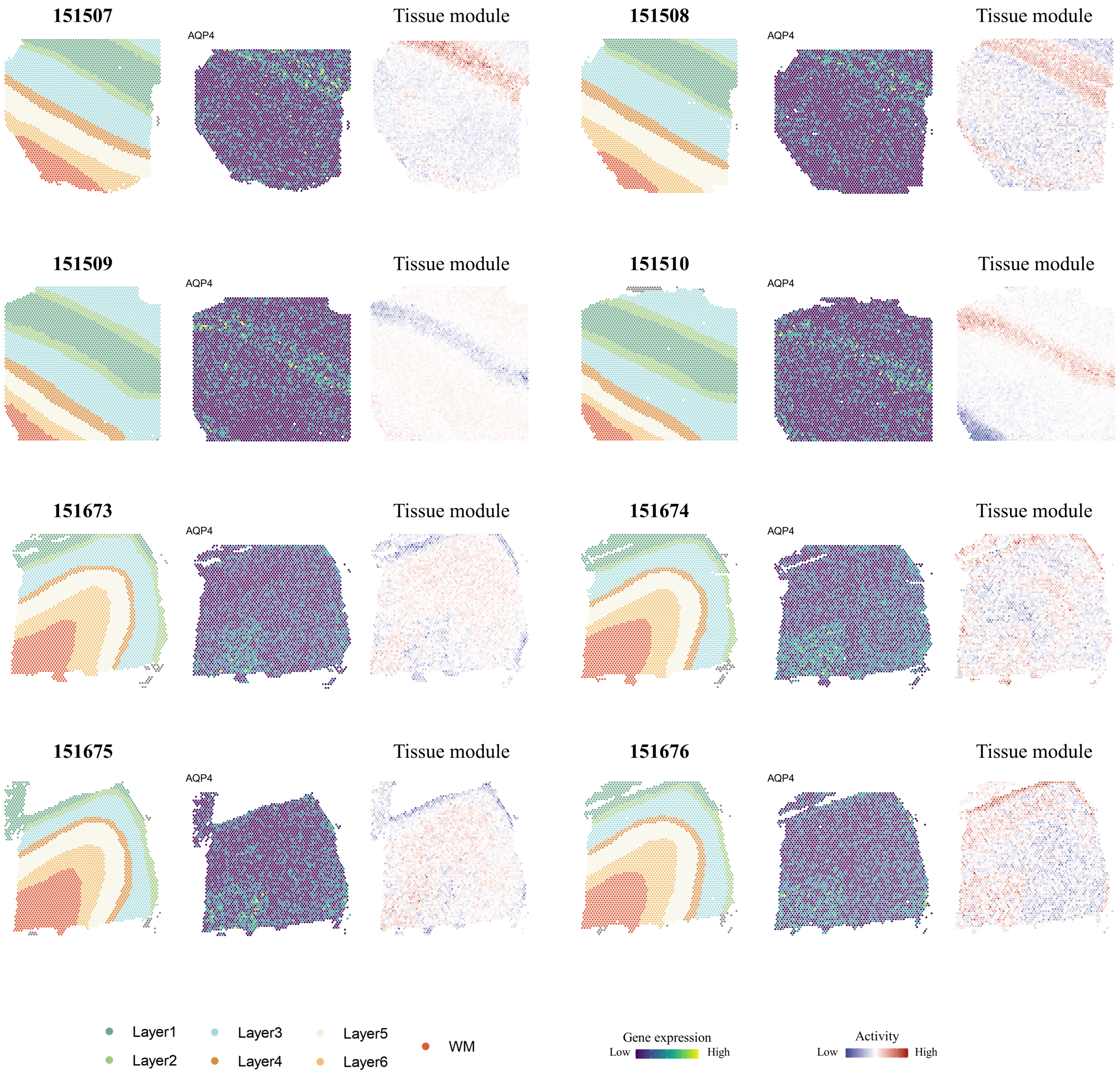

Fig. S56. Spatial expression of AQP4 in the DLPFC samples. Structure of the samples and the corresponding tissue modules representing layer 1 are also illustrated.

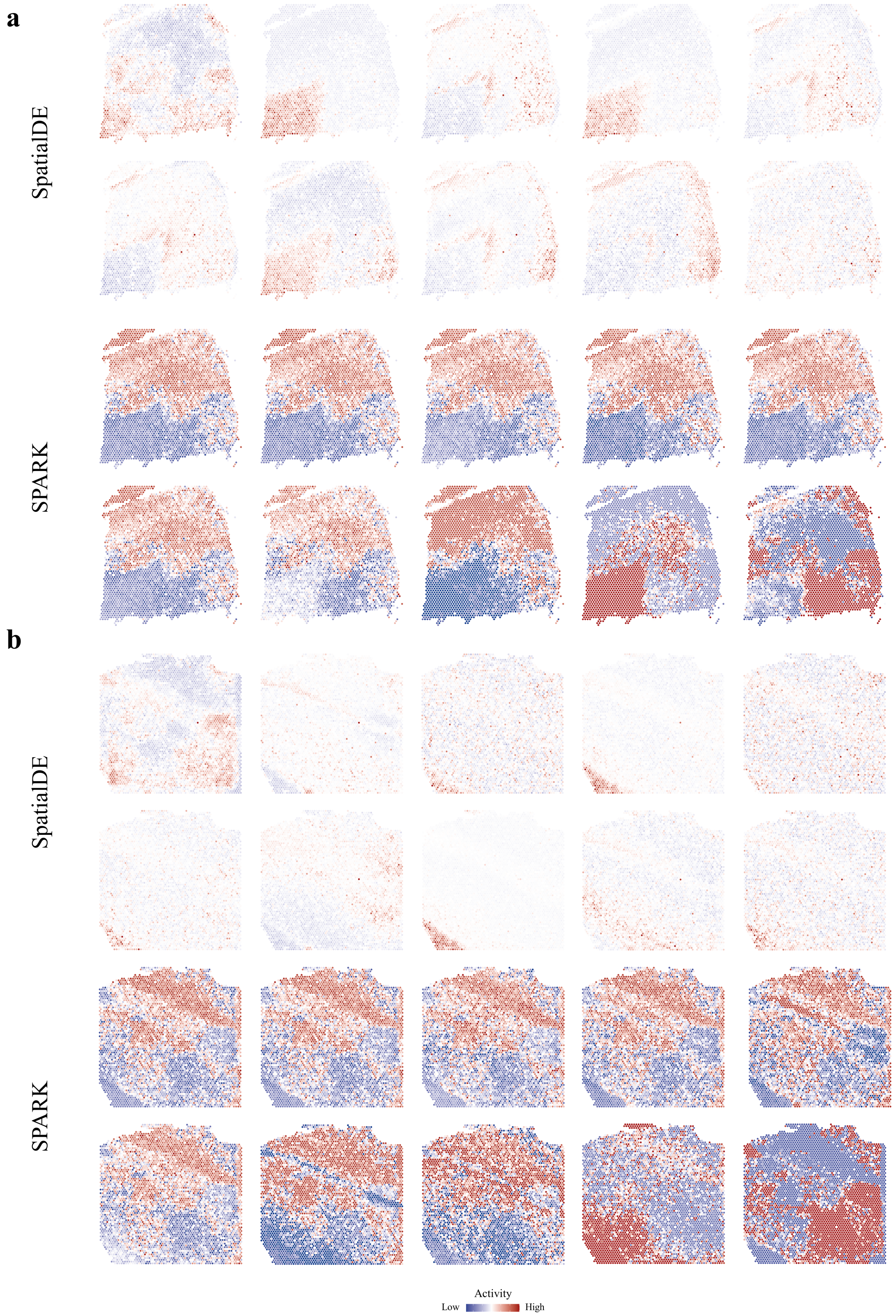

Fig. S57. Spatial patterns identified by SpatialDE and SPARK from DLPFC sample 151676 (a) and sample 151510 (b).

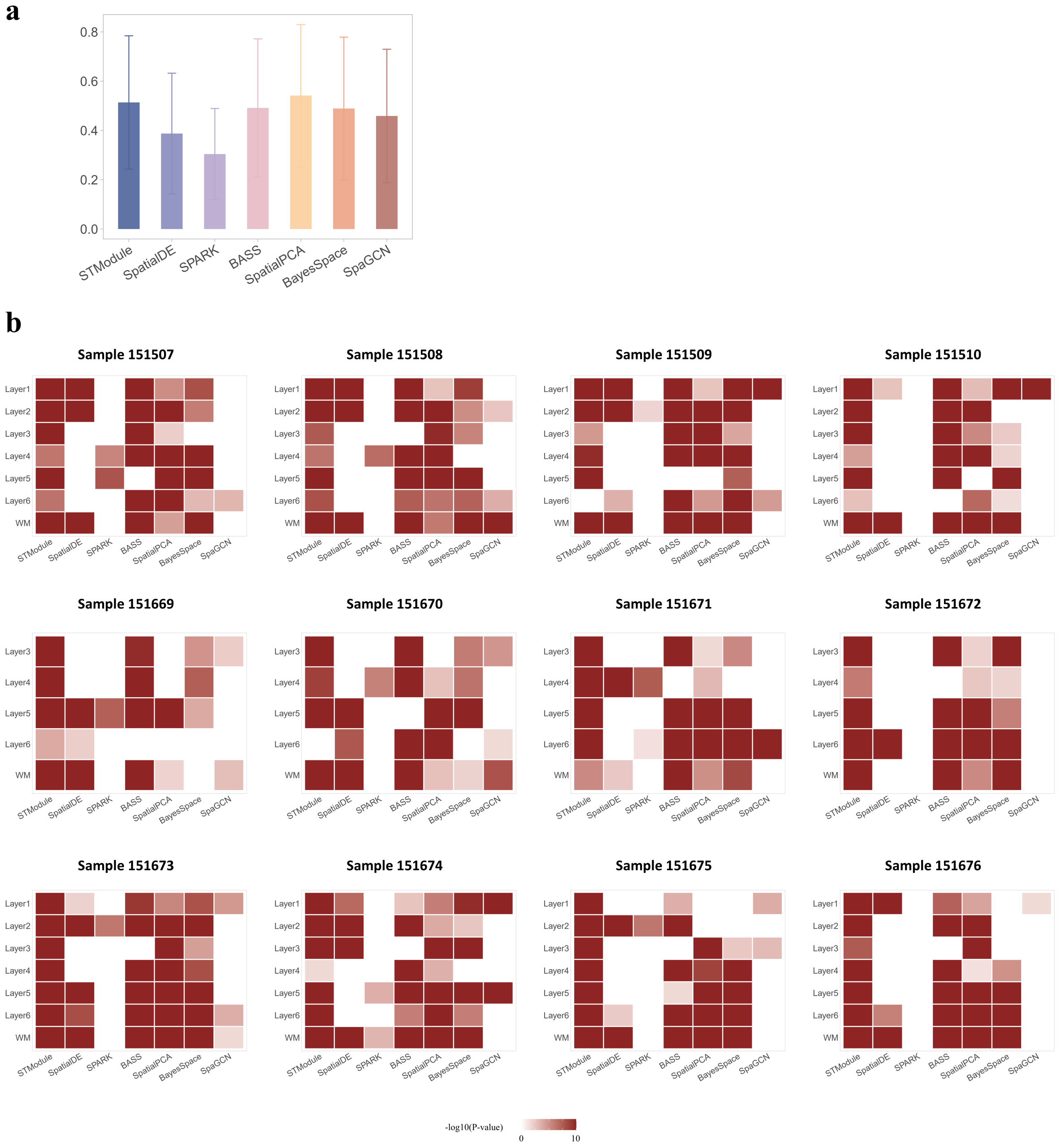

Fig. S58. Additional comparison for the DLPFC dataset. a Comparison of average AUPR of the methods in identifying the layers of all samples. **b** Gene set enrichment analysis of the differential or associated genes detected by the methods for all samples. Color indicates the adjusted P-value. White color indicates adjusted P-value ≥ 0.05.

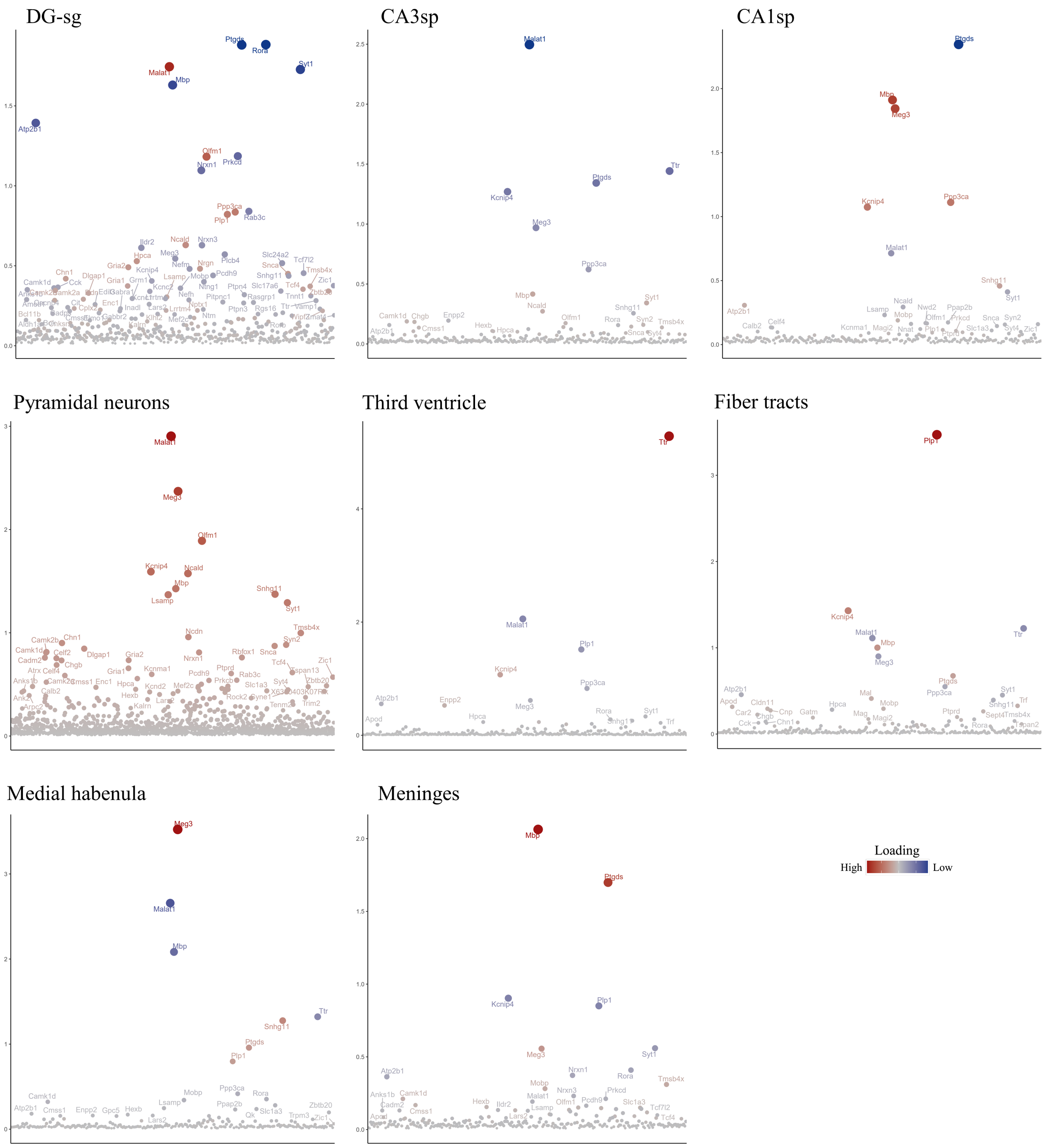

Fig. S59. Gene loadings of the tissue modules identified by STModule from mouse hippocampus. Y-axis represents the absolute value of gene loading and color indicates the sign and value of loading. X-axis represents the alphabetical order of the gene names. DG-sg, granule cell layer of dentate gyrus. CA3sp, pyramidal layer of cornu ammonis 3 (CA3). CA1sp, pyramidal layer of CA1.

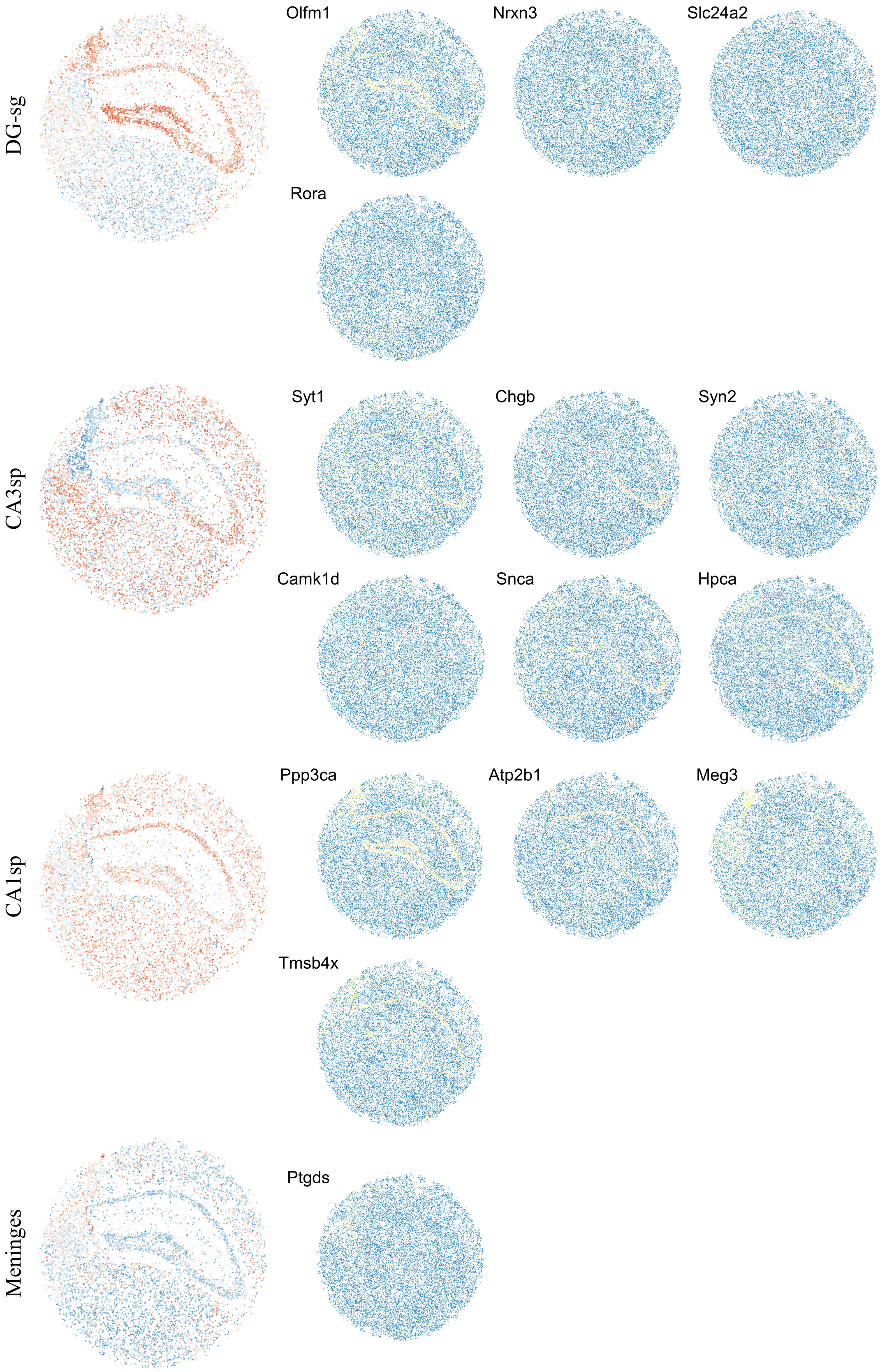
DG-sg
Olfm1
Nrxn3
Slc24a2
Rora
CA3sp
Syt1
Chgb
Syn2
Camk1d
Snca
Hpca
CA1sp
Ppp3ca
Atp2b1
Meg3
Tmsb4x
Meninges
Ptgds

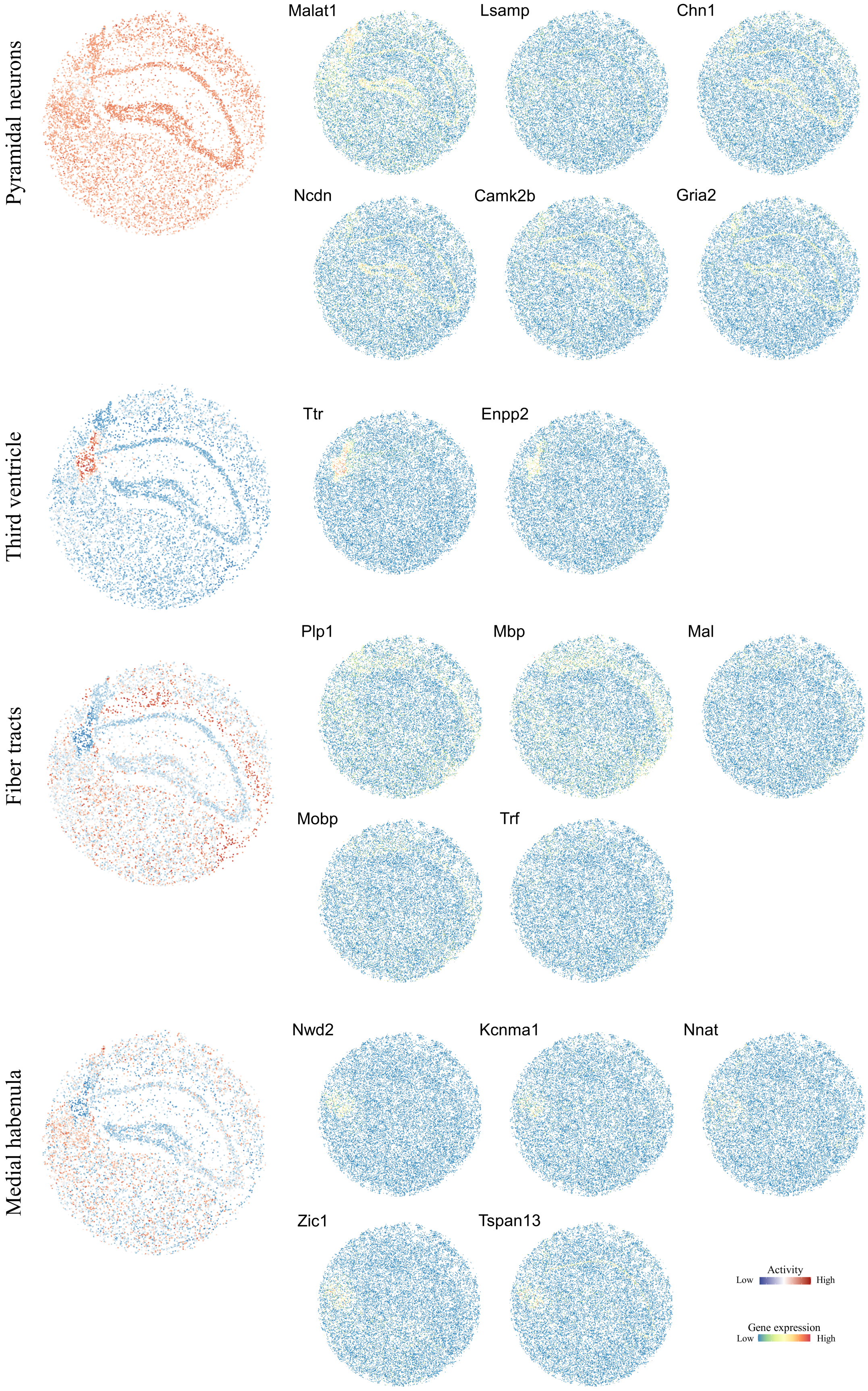

Fig. S60. Spatial expression of representative associated genes of the tissue modules identified by STModule from mouse hippocampus.

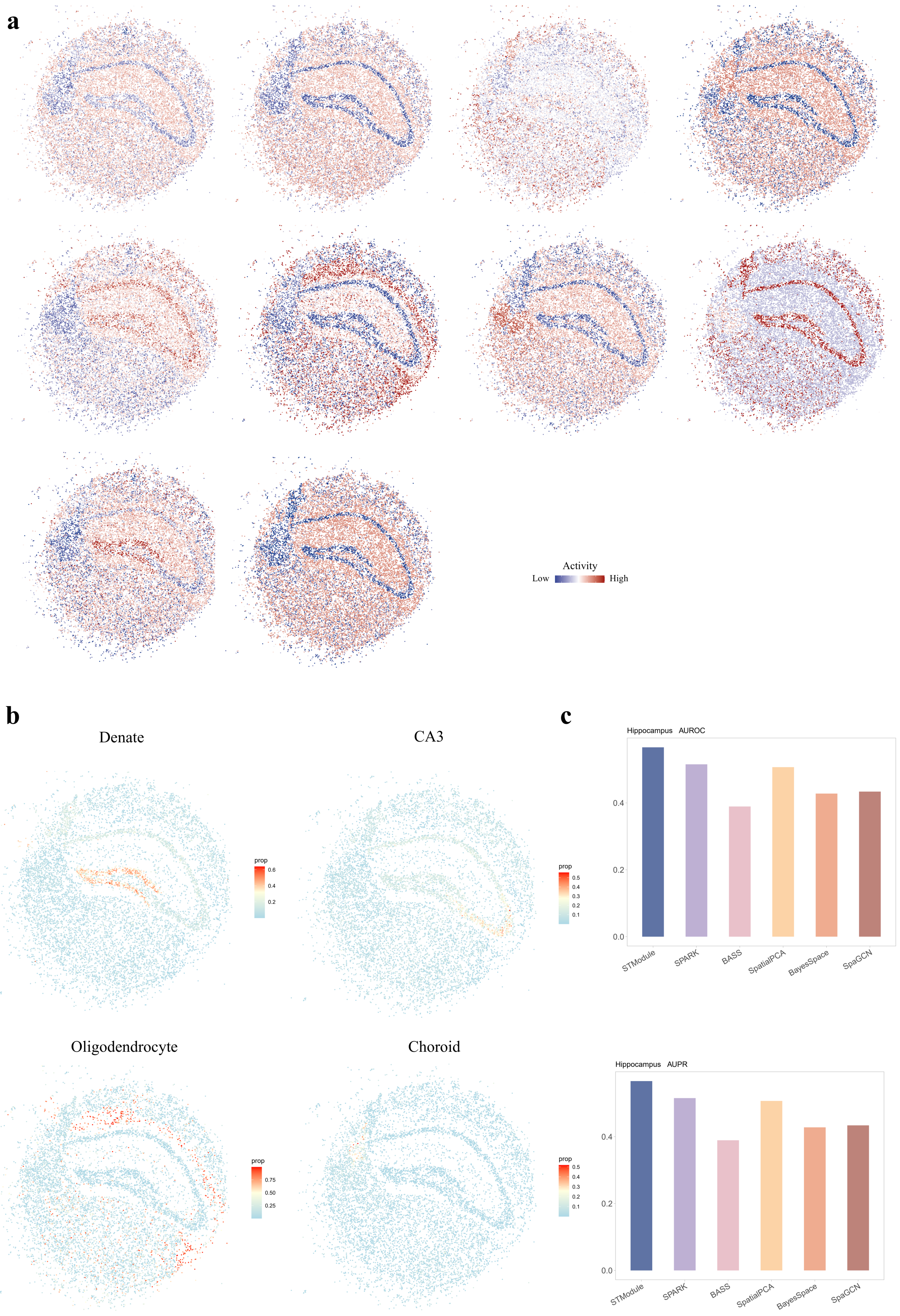

Fig. S61. Additional comparison for the hippocampus dataset. a Spatial patterns identified by SPARK-X. **b** Spatial distribution of major components estimated by CARD. **c** Comparison of average AUROC and AUPR of the methods in detecting the domains in **b**.

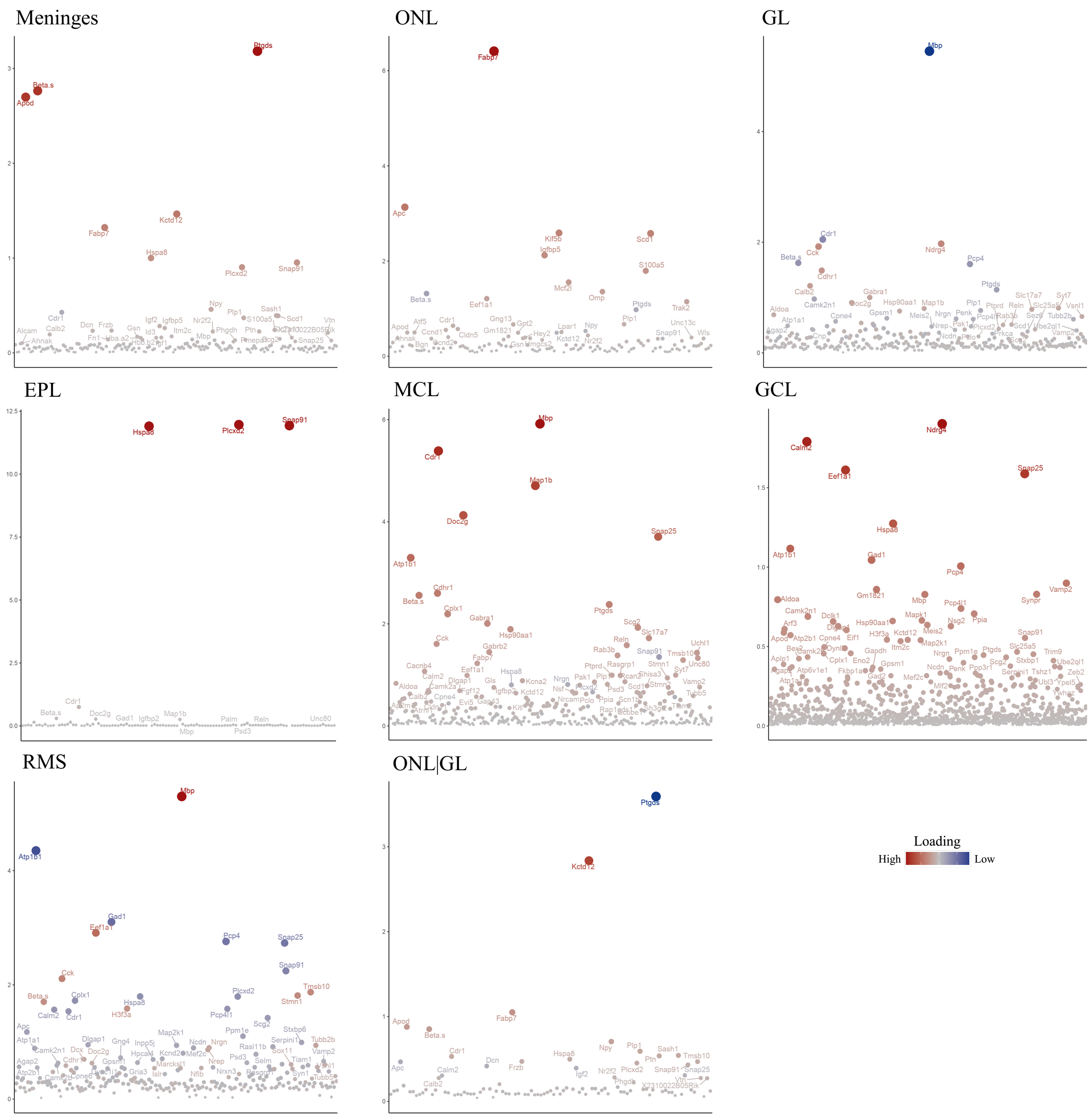

Fig. S62. Gene loadings of the tissue modules identified by STModule from the ST MOB data. Y-axis represents the absolute value of gene loading and color indicates the sign and value of loading. X-axis represents the alphabetical order of the gene names. ONL, olfactory nerve layer. GL, glomerular layer. EPL, external plexiform layer. MCL, mitral cell layer. IPL, internal plexiform layer. GCL, granule cell layer. RMS, rostral migratory stream. ONL|GL, interface between ONL and GL.

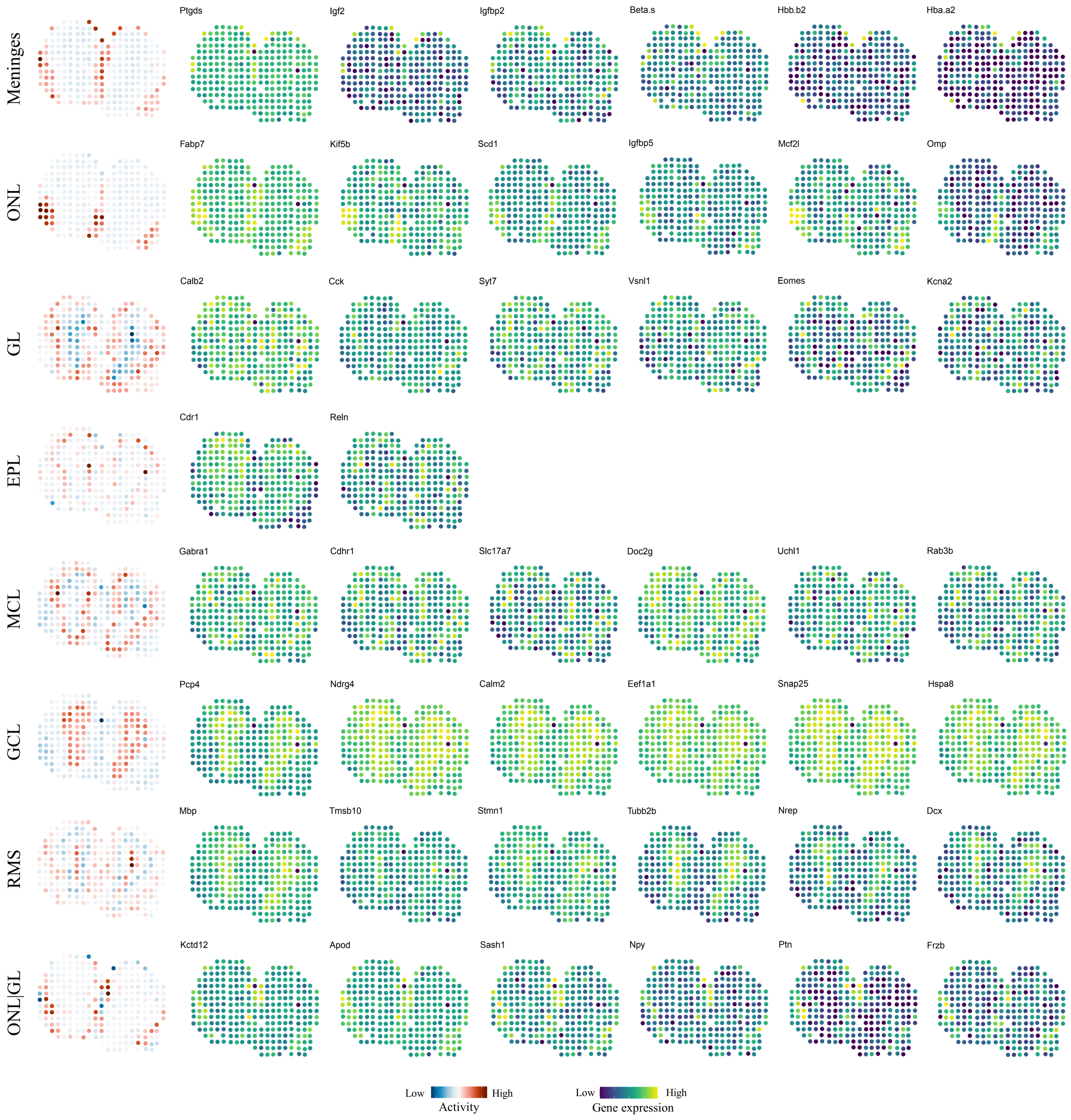

Fig. S63. Spatial expression of representative associated genes of the tissue modules identified by STModule from the ST MOB data.

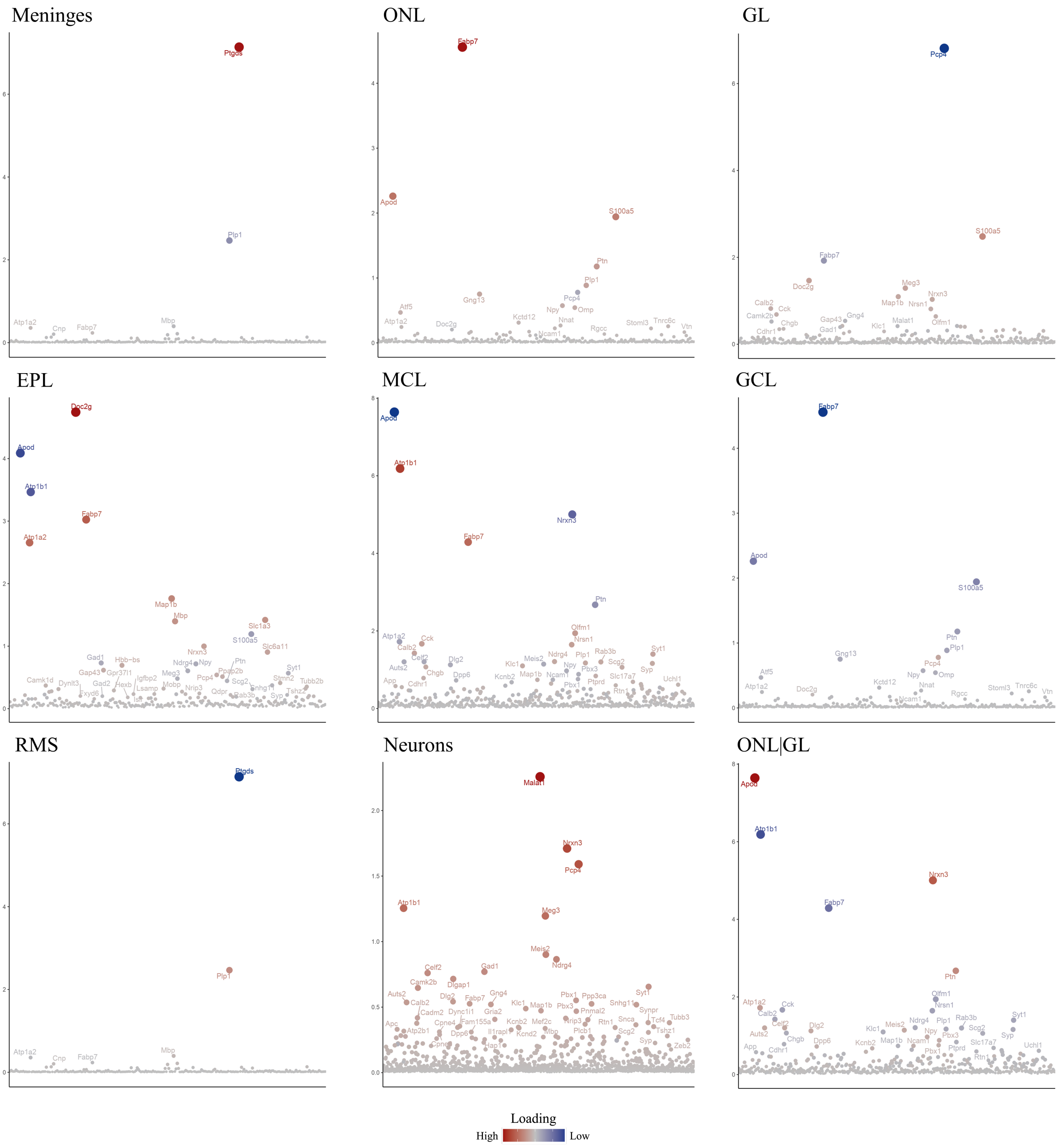

Fig. S64. Gene loadings of the tissue modules identified by STModule from the Slide-seqV2 MOB data. Y-axis represents the absolute value of gene loading and color indicates the sign and value of loading. X-axis represents the alphabetical order of the gene names.

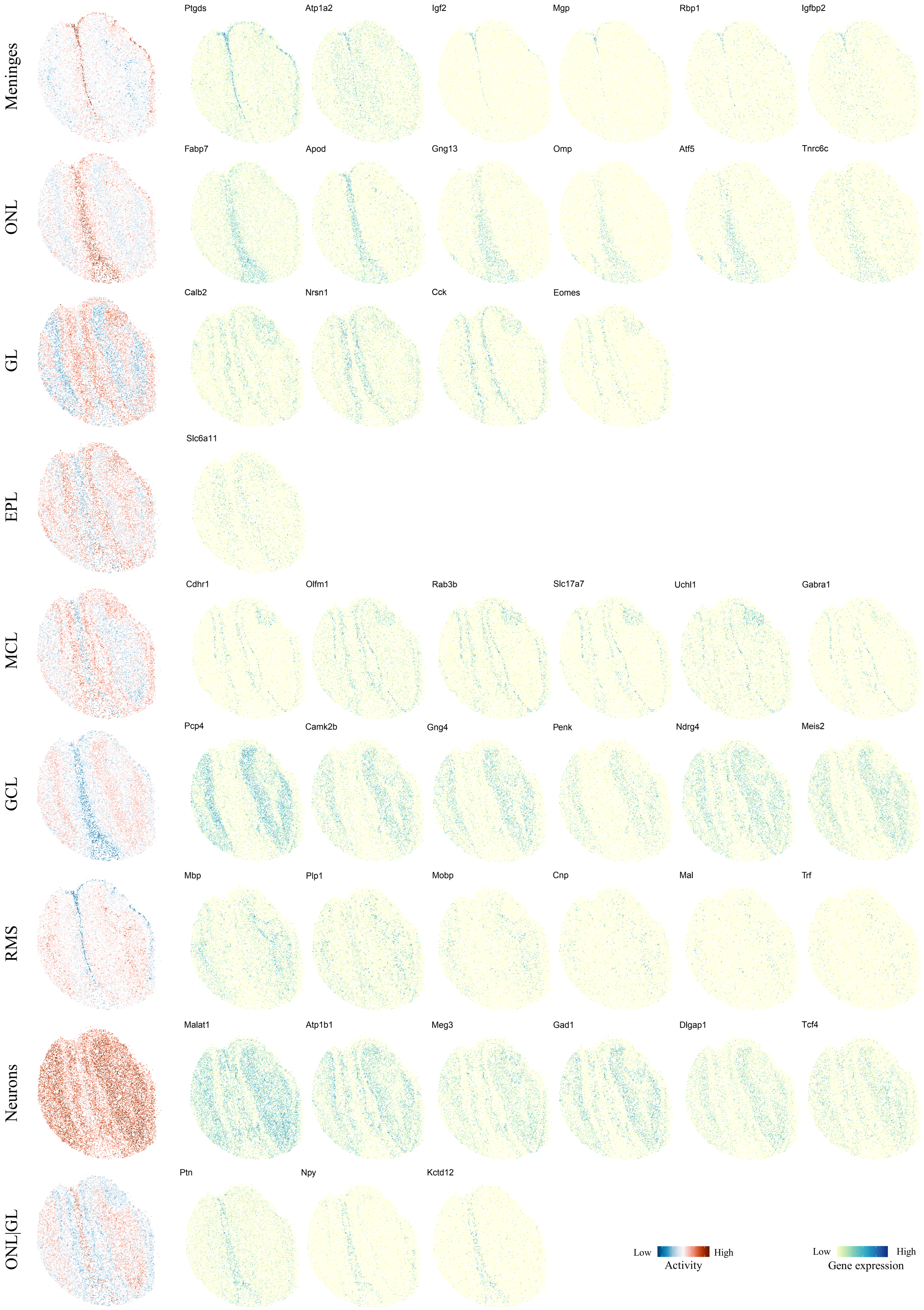

Fig. S65. Spatial expression of representative associated genes of the tissue modules identified by STModule from the Slide-seqV2 MOB data.

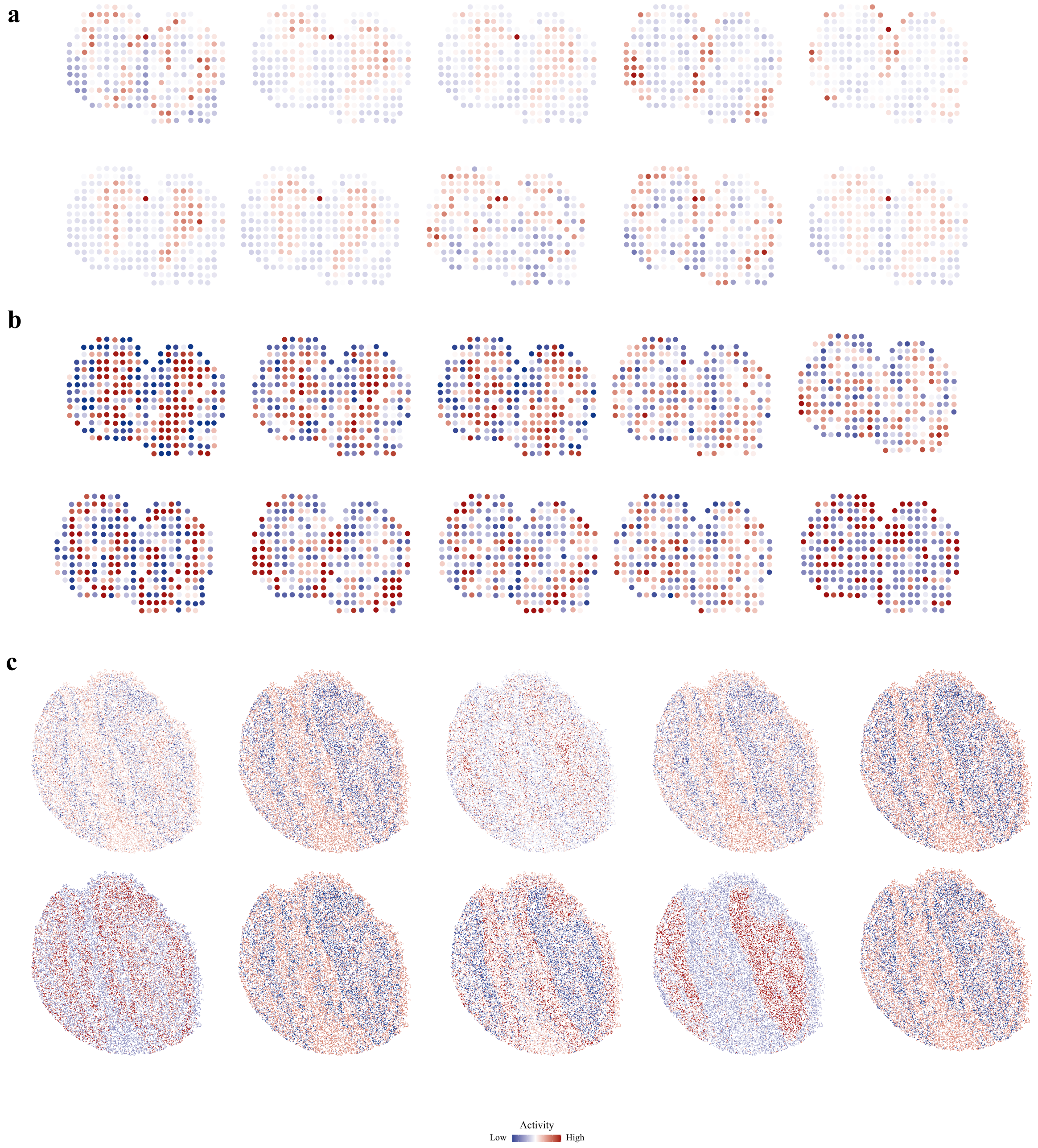

Fig. S66. Spatial patterns identified by SpatialDE and SPARK from the ST and Slide-seqV2 MOB datasets. **a** Spatial patterns identified by SpatialDE from the ST MOB data. **b, c** Spatial patterns identified by SPARK from the ST (**b**) and Slide-seqV2 (**c**) MOB datasets.

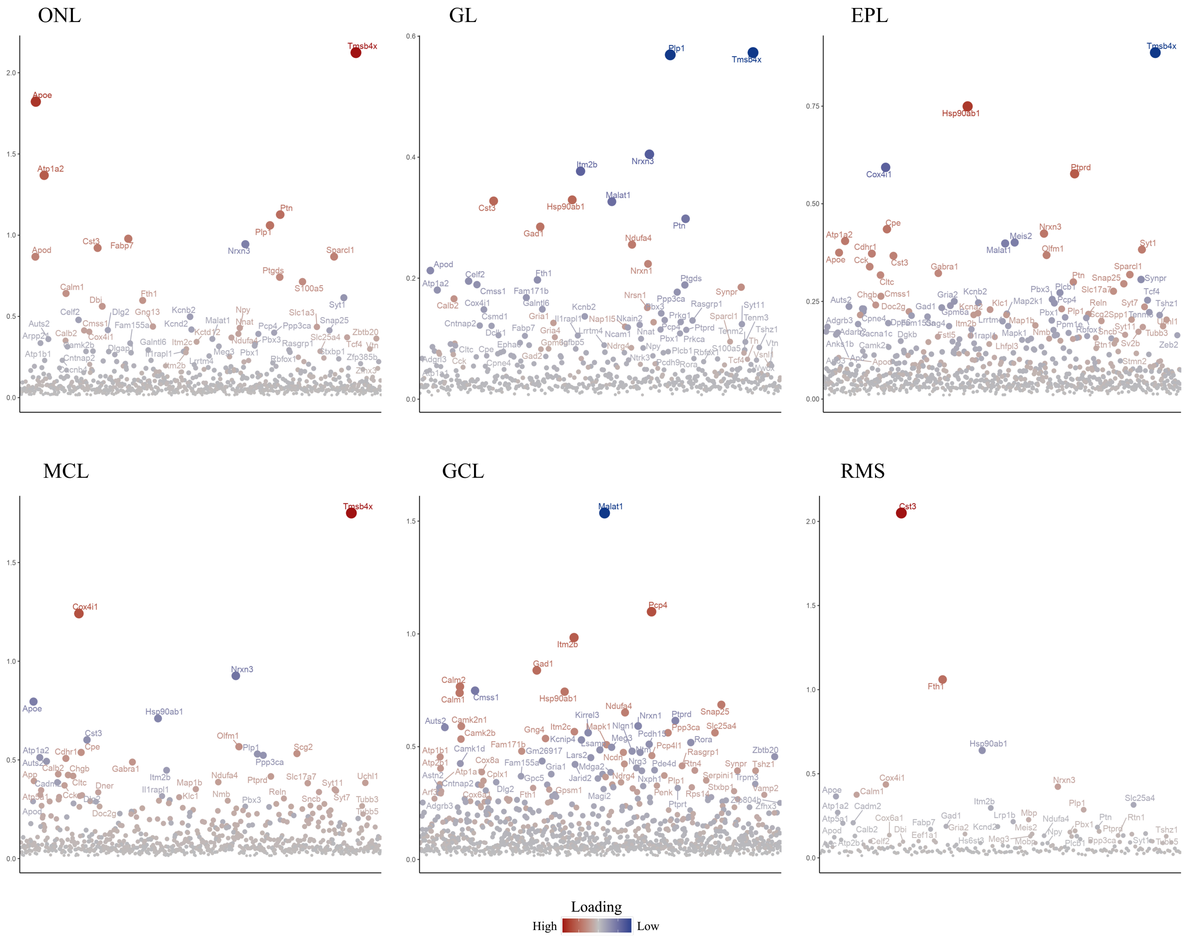

Fig. S67. Gene loadings of the tissue modules identified by STModule from the Stereo-seq MOB data. Y-axis represents the absolute value of gene loading and color indicates the sign and value of loading. X-axis represents the alphabetical order of the gene names.

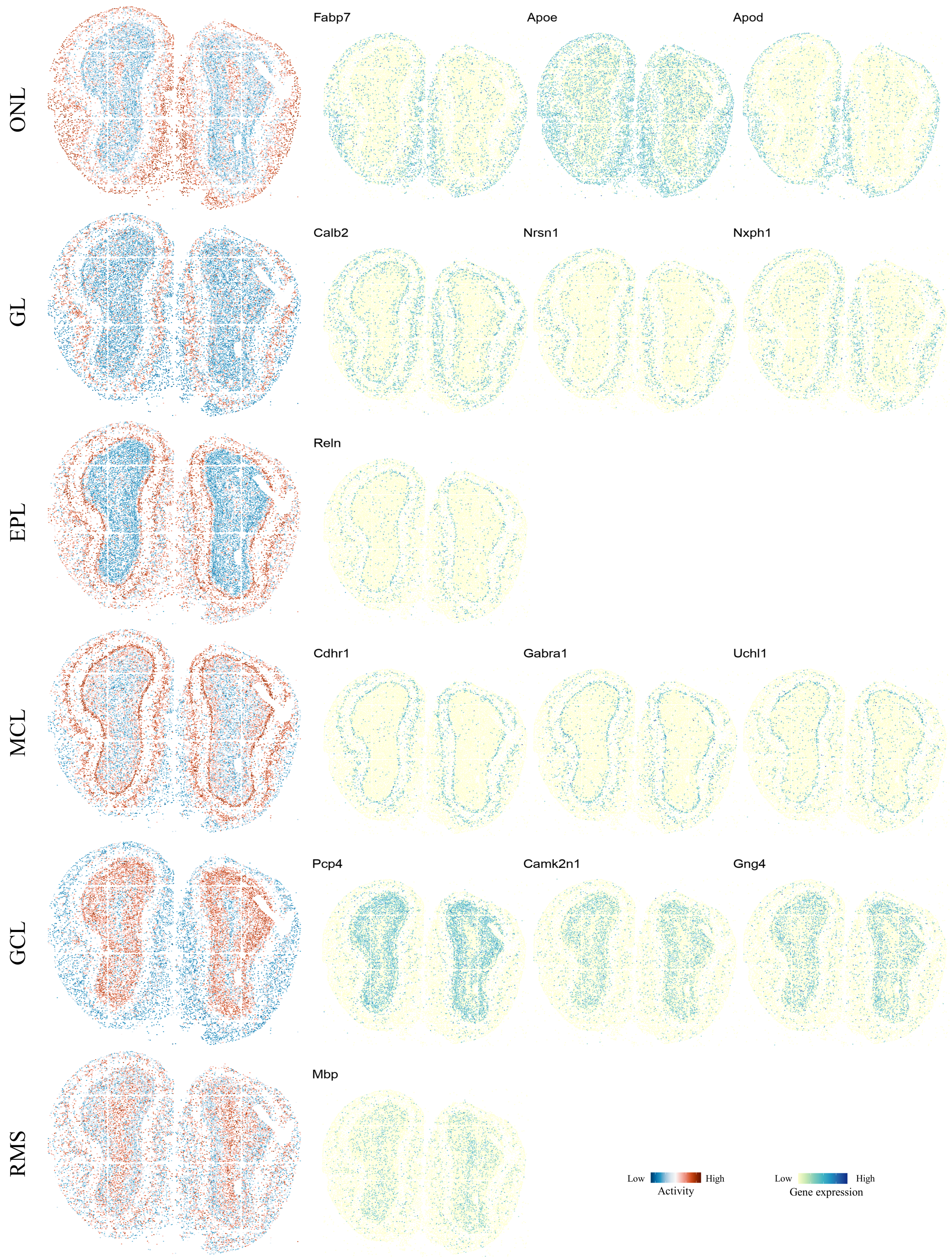

Fig. S68. Spatial expression of representative associated genes of the tissue modules identified by STModule from the Stereo-seq MOB data.

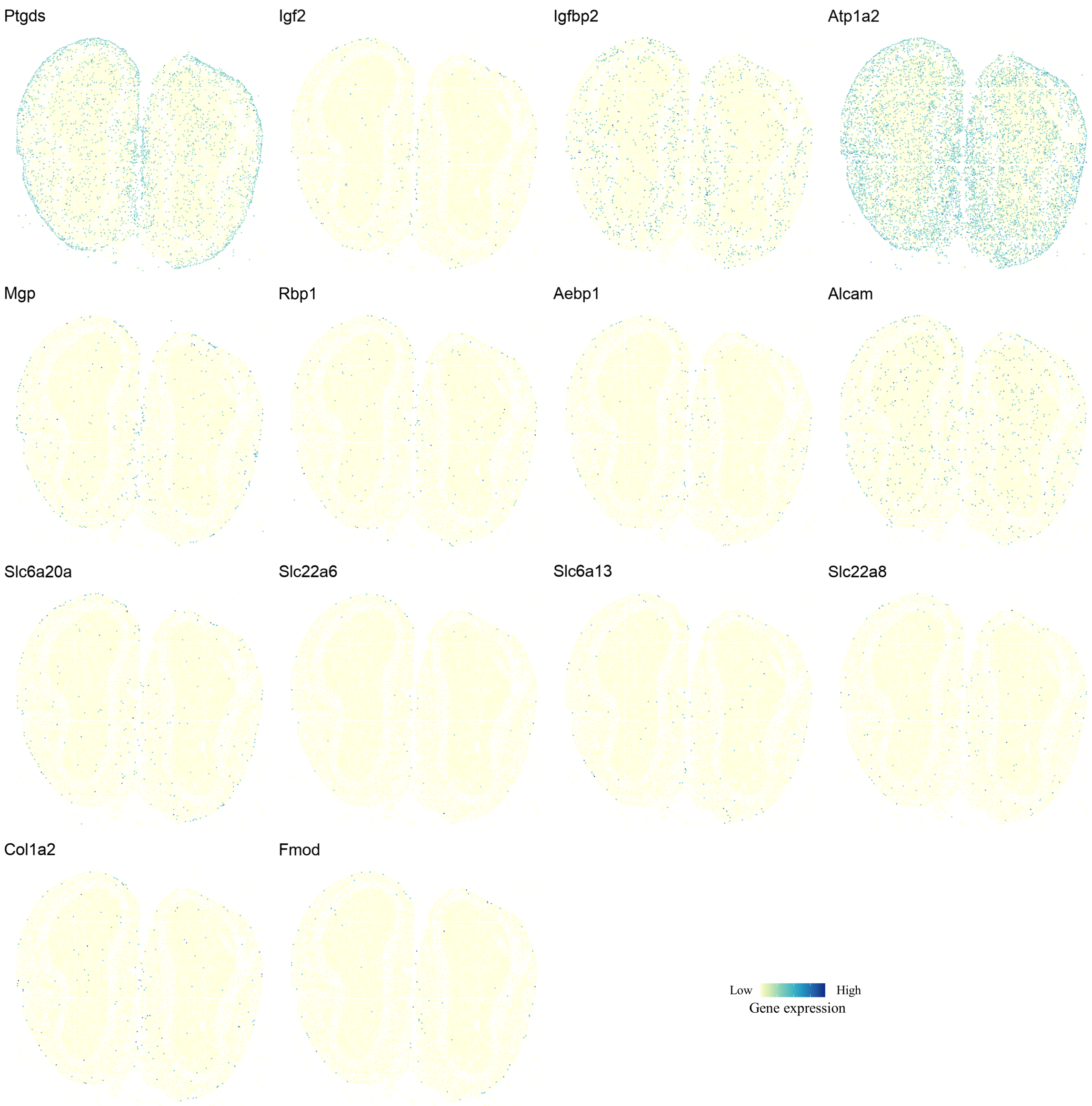

Fig. S69. Spatial expression of representative associated genes of meninges in Stereo-seq MOB, identified from ST and Slide-seqV2 MOB.

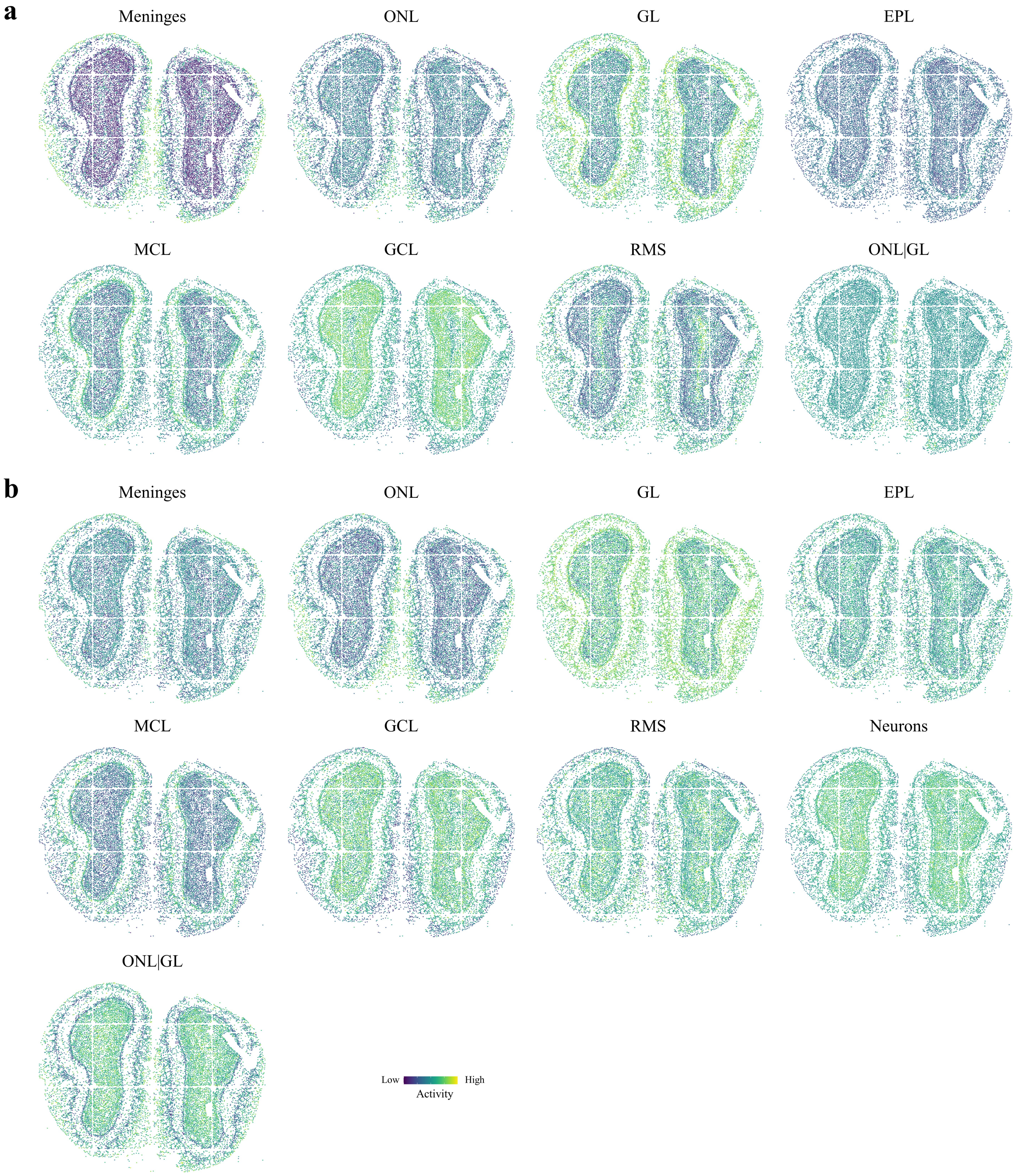

Fig. S70. Application of the tissue modules identified from the ST (a) and Slide-seqV2 (b) MOB data to Stereo-seq MOB data.

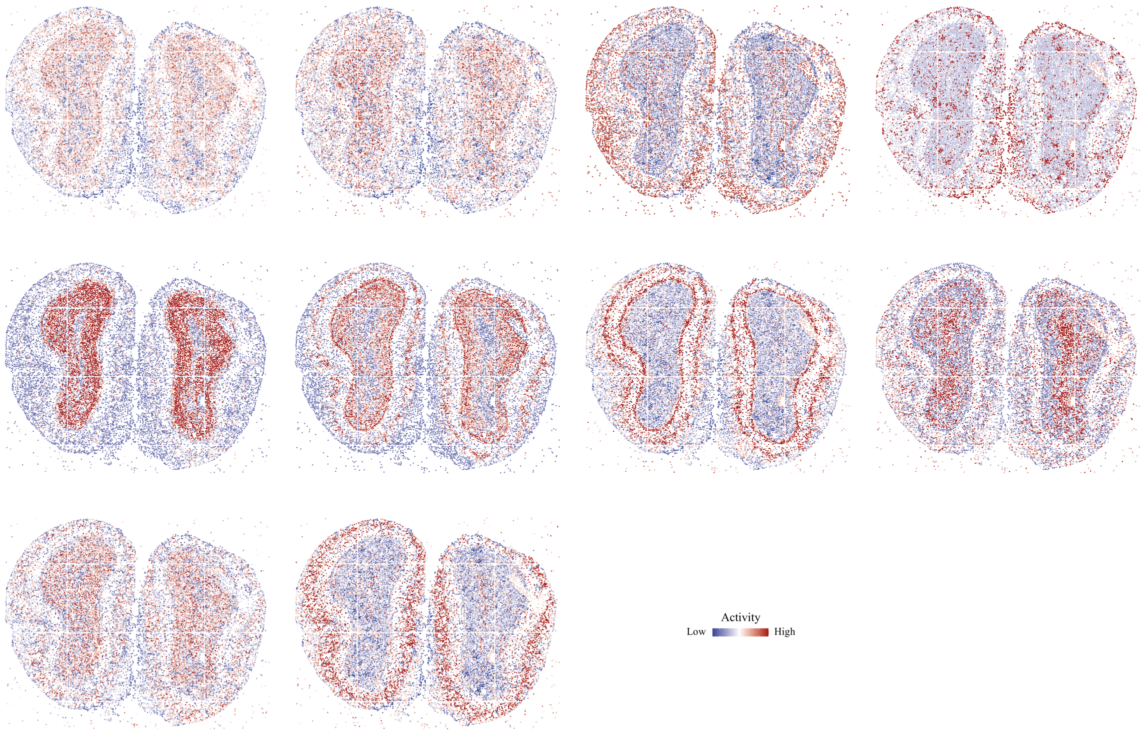

Fig. S71. Spatial patterns identified by SPARK from the Stereo-seq MOB datasets.

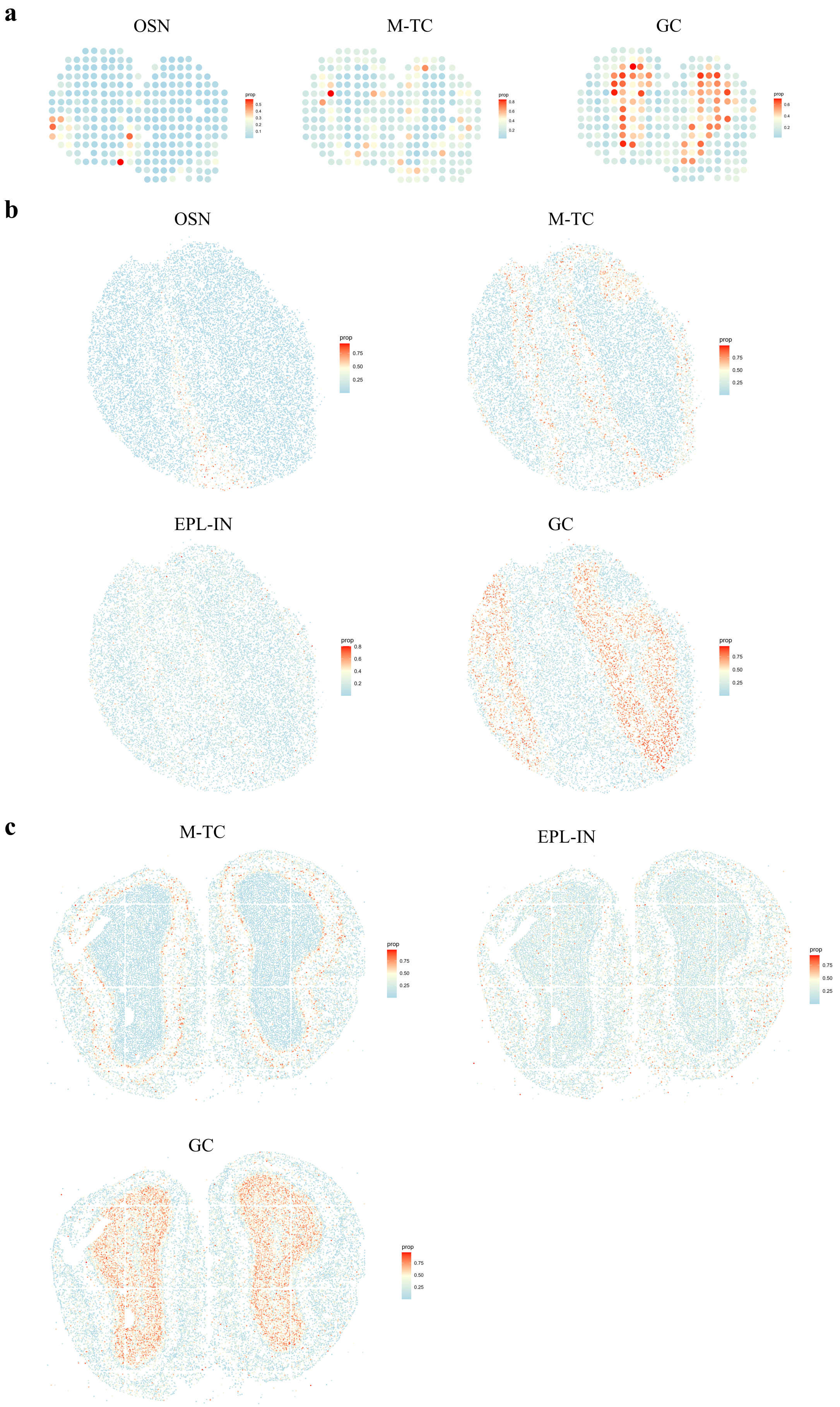
a
OSN
M-TC
GC
prop
b
OSN
M-TC
EPL-IN
GC
prop
0.75
0.50
0.25
0.8
0.6
0.4
0.2
c
M-TC
EPL-IN
GC
prop
0.75
0.50
0.25

d

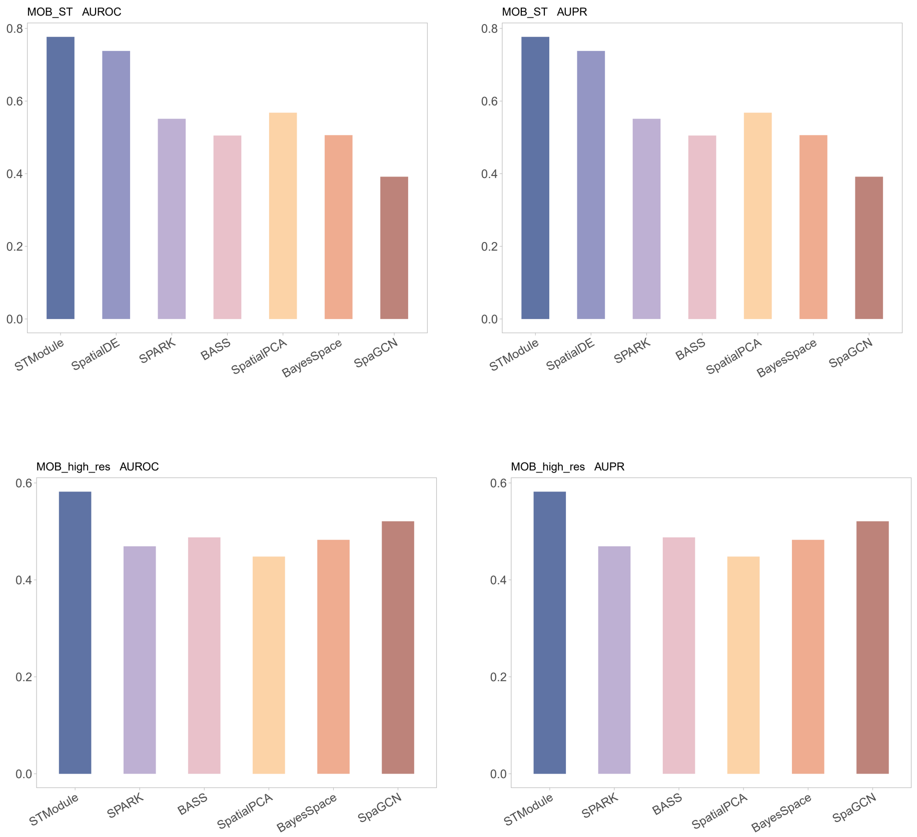

Fig. S72. Additional comparison for the MOB datasets. a-c Spatial distribution of major MOB components estimated by CARD for the ST data (**a**), Slide-seqV2 data (**b**) and Stereo-seq data (**c**). **d** Comparison of average AUROC and AUPR of the methods in detecting the domains in **a-c**. Above, results for the ST dataset. Bottom, results for the Slide-seqV2 and Stereo-seq datasets.

a

b

c

d

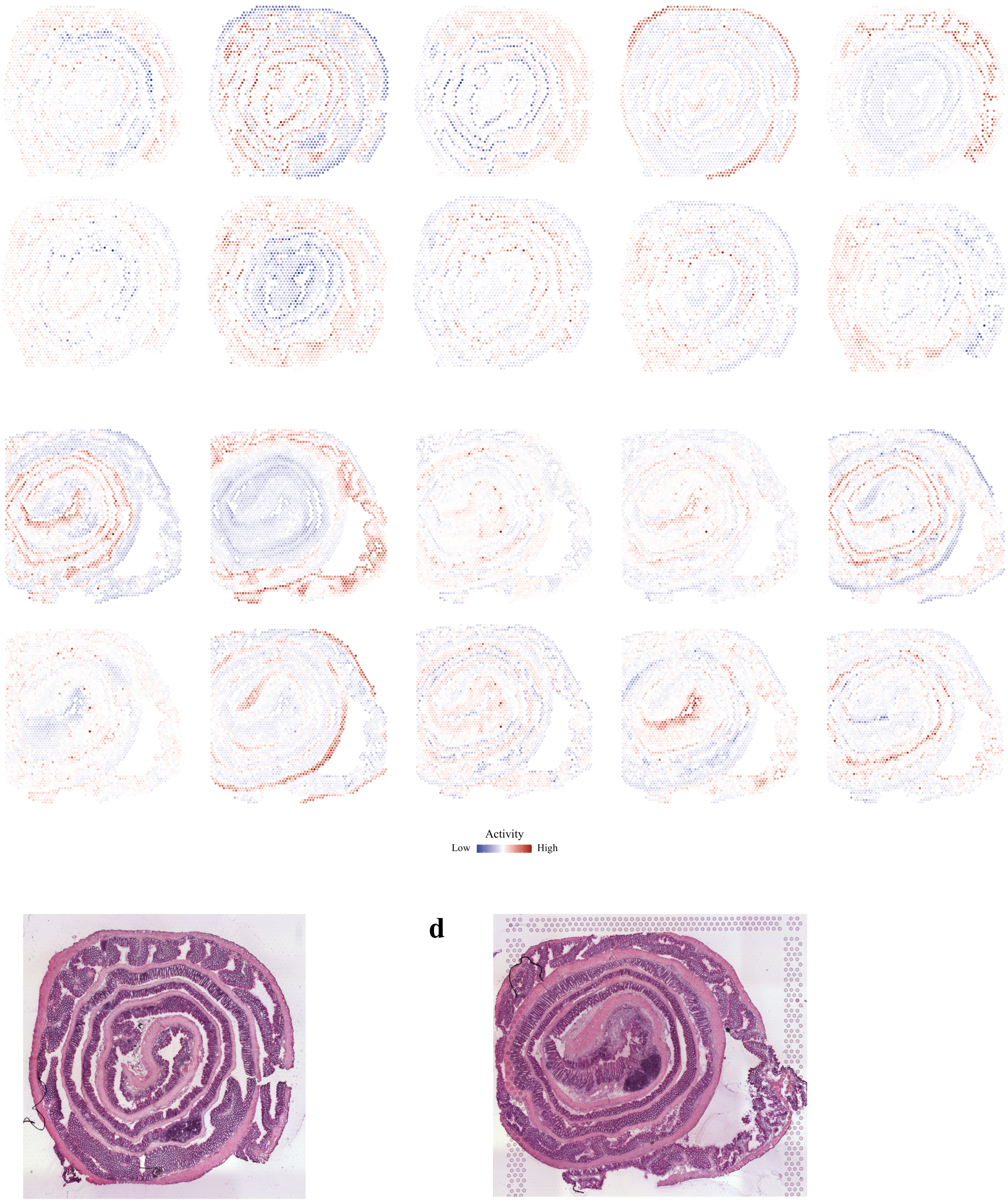

Fig. S73. Application of STModule to a mouse colon dataset profiled by 10x Visium. a, b Spatial maps of tissue modules identified by STModule for sample d0 of steady state (**a**) and sample d14 of healing state (**b**). **c, d** The H&E images of sample d0 (**c**) and sample d14 (**d**).

a

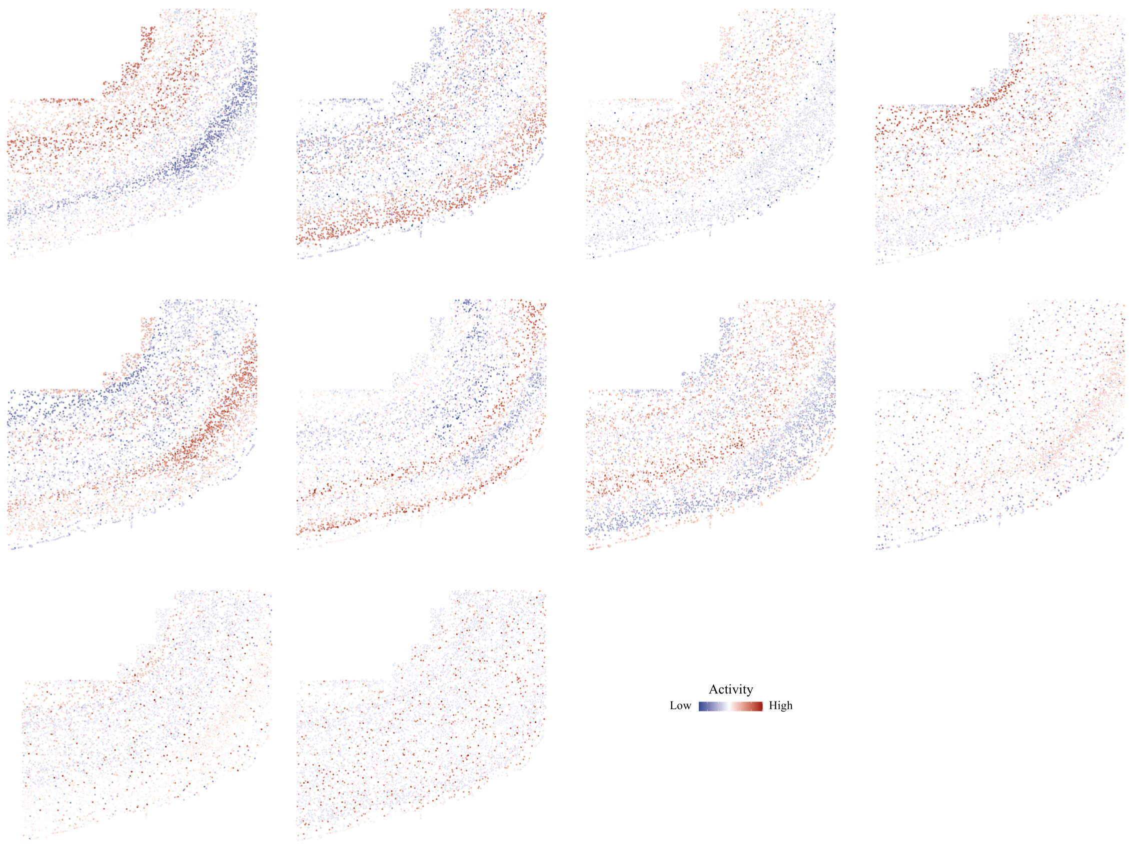

b

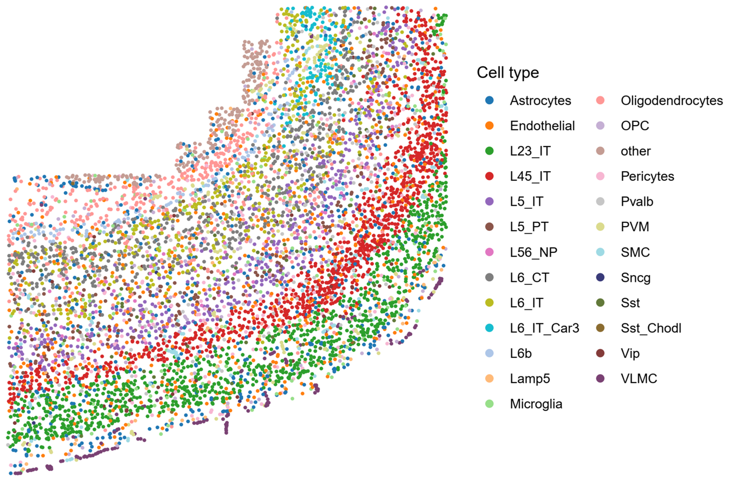

Fig. S74. Application of STModule to a mouse primary motor cortex dataset profiled by MERFISH. a Spatial maps of tissue modules identified by STModule. **b** Spatial distribution of different cell types in the tissue section. Colors indicate the cell types.

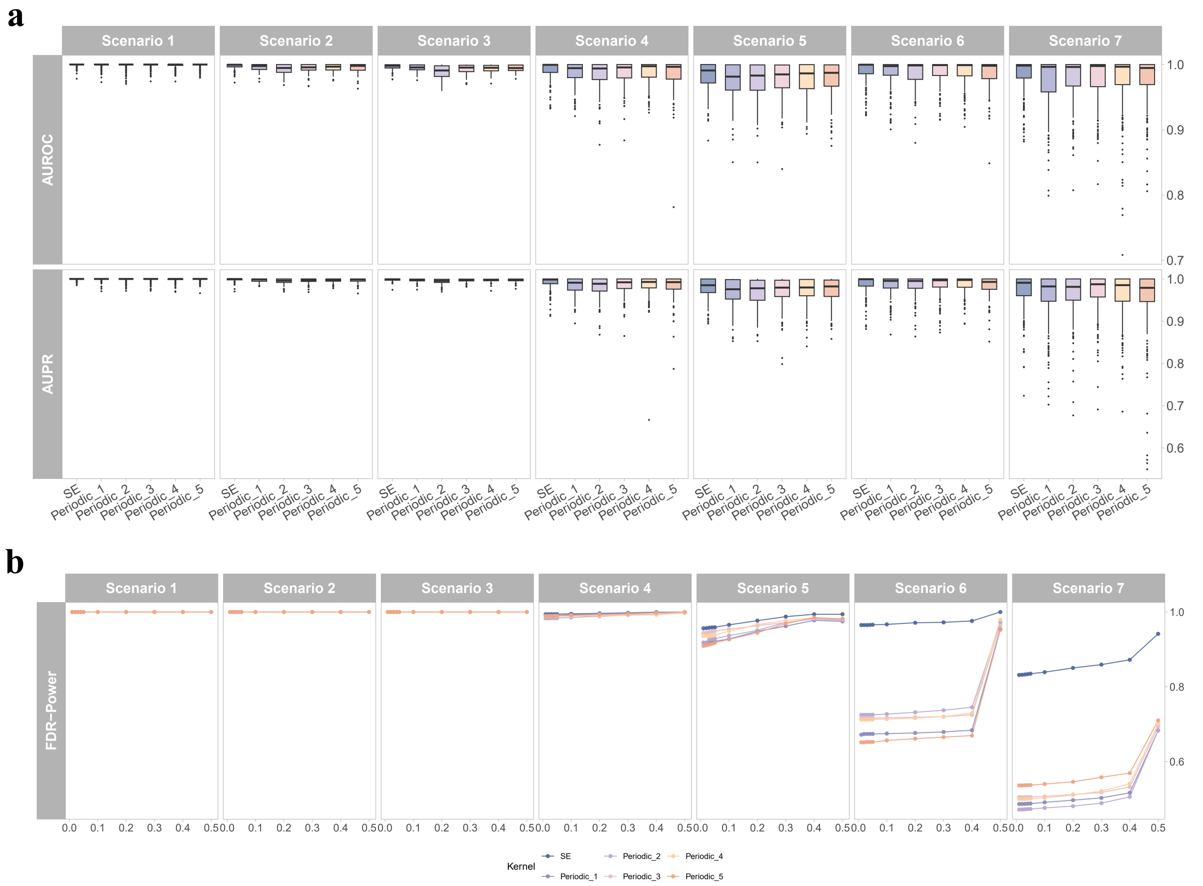

Fig. S75. Performance comparison of STModule using different kernels in the first set of simulations. SE, squared exponential kernel. Periodic 1-5, periodic kernels with different values for the parameter p.
